# Genomic and Phenotypic Bases of Salt Tolerance in *Sinorhizobium meliloti* : Candidate Traits for Bioinoculant Development Addressing Saline Soils

**DOI:** 10.1111/1751-7915.70304

**Published:** 2026-01-29

**Authors:** Agnese Bellabarba, Camilla Fagorzi, Giovanni Bacci, Francesca Decorosi, Alice Checcucci, Gaio Cesare Pacini, Abdelkader Bekki, Amina El Hadj Mimoune, Khalid Azim, Majida Hafidi, Alessio Mengoni, Francesco Pini, Carlo Viti

**Affiliations:** ^1^ Department of Agricultural, Food, Environmental and Forestry Sciences (DAGRI) University of Florence Firenze Italy; ^2^ Genexpress Laboratory, Department of Agronomy, Food, Environmental and Forestry Sciences (DAGRI) University of Florence Sesto Fiorentino Italy; ^3^ Department of Biology University of Florence Sesto Fiorentino Italy; ^4^ Laboratory of Rhizobia Biotechnology and Plant Breeding University Oran1 Es Senia Algeria; ^5^ National Institute of Agricultural Research (INRA), Regional Center of Agadir, Inzegane Principale Agadir Morocco; ^6^ Department of Biology, Faculty of Science Moulay Ismail University Zitoune Meknés Morocco; ^7^ Department of Biosciences, Biotechnology and Environment (DBBA) University of Bari ‐ Aldo Moro Bari Italy

**Keywords:** GWAS, legumes, phenotype microarray, rhizobia, salt tolerance, *Sinorhizobium meliloti*

## Abstract

Soil salinity poses a major challenge to the legume‐rhizobia symbiosis development, thereby affecting sustainable agriculture. Selecting NaCl‐tolerant strains and enhancing the native strains' fitness under salt stress are essential steps for the restoration of marginal areas. In this work, 49 *Sinorhizobium meliloti* strains, the rhizobial species forming symbiotic nitrogen‐fixing associations with alfalfa—including 21 de novo‐sequenced field isolates—were subjected to a thorough in vitro screening for salt tolerance at progressively higher NaCl concentrations. Field isolates showed genome‐based geographical clustering but contrasting salt tolerance abilities. Indeed, genome‐wide association (GWA) analysis on the strains' whole‐genome sequencing data indicated several loci associated with the variability in salt tolerance. Candidate genes were involved in various processes including cell wall organisation, LPS biosynthesis, quorum sensing, and carbohydrate transport and metabolism. The relationship with carbohydrate metabolism was further confirmed by Phenotype Microarray analysis which indicated salt‐tolerant strains having enhanced capacity in carbon source usage. These findings reveal synergistic pathways underlying salt tolerance and suggest candidate traits (e.g., quorum sensing, carbohydrate synthesis and modification) for developing bioinoculants to enhance legume performance in saline soils.

## Graphical abstract

Genome‐wide association analysis on 49 *Sinorhizobium meliloti* strains (21 de novo sequenced) reveals extensive phenotypic heterogeneity in salt tolerance, closely associated with quorum sensing, cell surface stability and adaptation. Salt‐tolerant strains exhibit a wider metabolic capacity, especially in carbon sources exploitation, underscoring their potential for adaptability in harsh conditions.

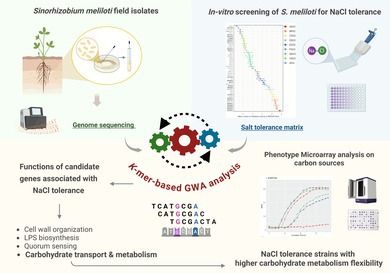

## 1 Introduction

Degradation of soil quality due to desiccation and salinity is a major constraint on agricultural productivity in arid and semi‐arid regions (Singh 2021). Evaporation induced by high temperature, low precipitation and irrigation with salt‐rich water due to scarce availability of fresh water are among the main drivers that increase salinity in agricultural soils (Tomaz et al. 2020). Globally, almost 20% of the world's agricultural land is affected by salinity, and projections suggested that by 2050, up to half of the world's croplands may be affected by salinisation (Nachshon 2018). Salinisation occurs mainly due to the accumulation of sodium (Na^+^) and chloride (Cl^−^) ions such that NaCl can reach 50%–80% of the total soluble salts in most saline soils (Rengasamy 2010).

Saline stress modifies bacterial community composition both in bulk (Rath et al. 2019; Zhang et al. 2019; Li et al. 2021) and rhizospheric soil (Szymańska et al. 2018; Yuan et al. 2019; Yukun et al. 2021; Islam et al. 2022) and inhibits key nitrogen metabolism driven by bacteria (e.g., ammonification, nitrogen fixation, nitrification and denitrification processes) (Li et al. 2021). This has important implications for sustainable agriculture as salinity negatively impacts rhizobia, bacteria that fix nitrogen in a symbiotic interaction with legumes. Rhizobia induce the formation of nodules on the plant roots where they fix atmospheric nitrogen and provide ${\mathrm{NH}}_{4}^{+}$ to their legume partner, while the plant provides nutrients to rhizobia in the form of carbohydrates (Haag et al. 2012). Efficient symbiosis under salt stress requires both partners to cope with osmotic and ionic constraints (Zahran 1999; Chakraborty and Harris 2022; Hawkins and Oresnik 2022). Host plants are generally more sensitive to salinity than their rhizobial partner (Mohammad et al. 1991), and it makes the choice of saline tolerant cultivars a key factor for legume productivity in soils affected by salinity (Nadeem et al. 2019). Rhizobial strains greatly vary in growth and survival under salt stress (Roumiantseva and Muntyan 2015), and the application of tolerant rhizobia improves the establishment of an effective rhizobium‐legume symbiosis under salinity (Elsheikh and Wood 1995; Zou et al. 1995; Hashem et al. 1998; Nogales et al. 2002; Bertrand et al. 2015; Roumiantseva and Muntyan 2015).

Thus, investigation of the genetic basis of salt tolerance in rhizobia is expected to contribute to the selection of symbiotic systems with adaptative potential to saline soils. The current knowledge on salt tolerance mechanisms in rhizobia comes mainly from studies of direct/reverse genetic (Chien et al. 1992; Wei et al. 2004; Boscari et al. 2006; Miller‐Williams et al. 2006; Vriezen et al. 2013) and on gene/protein expression studies based on the analysis of the potential of single strains (Domínguez‐Ferreras et al. 2006; Shamseldin et al. 2006; Liu et al. 2014; López‐Leal et al. 2014; Maximiano et al. 2021). Studies conducted to date have revealed a complex, multilevel salt tolerance mechanism in rhizobia, identifying the presence of osmoprotectans, such as glycine betaine (Boncompagni et al. 1999) and trehalose (Reina‐Bueno et al. 2012), which strongly contribute to stress tolerance. The uptake of K^+^ and the de novo synthesis of compatible solutes (Vriezen et al. 2007) have been recently linked to osmo‐tolerance together with the modification of the long‐chain exopolysaccharides (EPS) (Primo et al. 2020) and lipopolysaccharides (LPS) (Lloret et al. 1995; Vriezen et al. 2007). Moreover, other mechanisms, including regulation of central metabolism, DNA replication and cell division (elongation factors, DNA ligase, chaperones and cell division proteins) have been shown to play a role in osmotic tolerance (Wei et al. 2004; Miller‐Williams et al. 2006). Collectively, these studies revealed that osmotolerance in rhizobia is a key example of a quantitative phenotype influenced by multiple genetic determinants that act as direct contributors and modulators. However, the relative contribution of different genes or metabolic processes to the variability of salt tolerance in natural populations remains unclear, as does an overall view of salt tolerance mechanisms.

Genome‐wide association studies (GWAS) have emerged as powerful tools for linking genetic variation to complex phenotypic traits (Uffelmann et al. 2021). Though GWAS are typically used in studies involving eukaryotic organisms, they have been more recently adapted to predict the multigenic basis of phenotypes in several bacterial species (Power et al. 2016; Andras et al. 2020). Similar to the use of GWAS and quantitative trait loci (QTLs) in plants and animals for breeding new improved varieties and races, in bacteria GWAS results could be combined with synthetic biology approaches to develop improved strains using genetic determinants from the pangenome of the species (Yang et al. 2025). Indeed, this approach has been employed to identify genetic determinants of antibiotic resistance, biofilm production, and virulence factors in several pathogens (Holt et al. 2015; Lees et al. 2016; Farhat et al. 2019; Hwang et al. 2021).

Alfalfa ( *Medicago sativa* ) is a globally significant legume forage crop valued for its high protein content, contributing over a global market of ~$25–30 billion (Fortune Business Insights 2025). However, salinity and drought stresses significantly reduce global alfalfa yields, with estimated yield losses ranging from 20% to 50% under severe conditions, resulting in billions of dollars in economic losses worldwide (Qadir et al. 2014; Daud et al. 2025). These abiotic stresses not only diminish biomass production but also degrade forage quality, thereby threatening livestock productivity and the agricultural economy in arid and semi‐arid regions. As with other legume crops, alfalfa productivity strongly relies on the nitrogen‐fixing symbiotic partnership with its symbiotic rhizobium ( *Sinorhizobium meliloti* ), which represents a well‐established model for the study of symbiosis and nitrogen fixation (Jones et al. 2007). Strains of this species display a divided genome structure, including a large megaplasmid harbouring most of the functions for symbiotic nitrogen fixation and a classical open pangenome, with a large dispensable gene pool, and extensive transcriptomic differences (Galardini et al. 2015; Fagorzi et al. 2021; Bellabarba et al. 2010; diCenzo and Finan 2017; Riley et al. 2023). Such genomic and transcriptomic diversity reflects into a pattern of quantitative variation in the symbiotic phenotype with alfalfa, as well as in metabolic phenotypes (Biondi et al. 2009; Bellabarba et al. 2023). The divided genome structure has allowed for the construction of synthetic hybrid strains with replacement of ca. 25%–30% of the genes (Checcucci et al. 2018), indicating this species is a good model for bacterial strain amelioration of complex, multigenic traits (Geddes et al. 2021; Wagner et al. 2024; Kearsley et al. 2025).

In this study, GWAS analysis was applied to a pool of natural *S. meliloti* strains showing different levels of tolerance to NaCl, aiming to identify the panel of genetic determinants and functions associated with tolerance to NaCl for the development of future synthetic hybrid strains with increased salt tolerance and symbiotic nitrogen fixation with alfalfa as tailored microbial inoculants to saline agroecosystems.

## 2 Methods

### 2.1 In Vitro NaCl Tolerance of *S. meliloti* Strains

Strains of *S. meliloti* were grown on (TY) agar medium (5 g l^−1^ tryptone, 3 g l^−1^ yeast extract, and 0.4 g l^−1^ CaCl_2_, 16 g l^−1^ bacteriological agar) (Beringer 1974) and incubated at 30°C for 48 h. Then, individual colonies were picked up with a sterile cotton swab and suspended in 15 mL of TY liquid medium. Cell density was adjusted to OD_600_ = 0.1. To carry out the experiment, 125 μL of the bacterial suspension was dispensed in each well of an empty 96 well microplate. Then, each well was added with 5 μL of 50× Dye Mix A (Biolog, Hayward, CA, USA) and 125 μL of 2× NaCl solutions (0, 0.8, 1.2, 1.6, 1.8, 2 M). The final composition of the medium in each well was 2.5 g l^−1^ tryptone, 1.5 g l^−1^ yeast extract, 0.2 g l^−1^ CaCl_2_, dye MixA 1× (Biolog, Hayward, CA, USA), and NaCl at different concentrations (0 mM, 400 mM, 600 mM, 800 mM, 900 mM, 1 M). The initial cell density in the wells was OD600 = 0.05. Plates were incubated at 30°C in an OmniLog Reader (Biolog) for 96 h in static conditions. Readings were recorded for 96 h every 15 min, and data were analysed using the OmniLog‐PM software (Biolog). The metabolic activities of strains were recorded as Arbitrary OmniLog Units (AOU), which represents the area under the kinetic curves. Each condition was performed in triplicate. For each replicate, the AOU normalised value was calculated as the ratio between the AOU detected for each NaCl concentration divided by the AOU value of the control NaCl concentration at 0 mM. Data were expressed as the average of normalised values for each condition.

### 2.2 Genome Sequencing and Annotation

All strains were grown to stationary phase at 30°C in TY medium. Total genomic DNA was extracted using cetyltrimethylammonium bromide (CTAB) method (Perrin et al. 2015). Short‐read sequencing was performed at IGATech (Udine, Italy) using an Illumina NovaSeqTM 6000 System instrument with 150‐bp paired‐end reads. Reads were trimmed with using Trimmomatic v0.39 (Bolger et al. 2014) and then assembled into scaffolds using Unicycler v.0.5.0 with default parameters (Wick et al. 2017). To evaluate the genome assemblies QUAST tool v.5.0.2 were used (Mikheenko et al. 2018). Genomes were annotated with Prokka 1.14.6 (Seemann 2014). To construct an unrooted, core gene phylogeny, the pangenome of the 21 *S. meliloti* strains was calculated using Roary 3.11.0 with a percent identify threshold of 95%. File S1 reports the presence and absence matrix genes list of *S. meliloti* pangenome (Page et al. 2015). As part of Roary analysis, the nucleotide sequences of the 2859 core genes identified (those found in at least 99% of the genomes) were aligned and concatenated. The concatenated alignment was used to construct a maximum likelihood phylogeny (the bootstrap best tree following 100 bootstrap replicates, as determined by the extended majority‐rule consensus tree criterion) using RAxML 8.2.4 with the GTRCAT model (Stamatakis 2014). All phylogenies prepared in this study were visualised with the online iTOL webserver (Letunic and Bork 2016). Phylogenomic analysis was performed using the Genome Blast Distance Phylogeny (GBDP) on Type (Strain) Genome Server (TYGS) (Meier‐Kolthoff and Göker 2019). Briefly, related type strains were identified via the MASH algorithm and 16S rDNA comparisons (Ondov et al. 2016). Pairwise GBDP distances (algorithm ‘trimming’, formula d5) were used to calculate dDDH values and infer a balanced minimum evolution tree via FASTME 2.1.6.1 (Lefort et al. 2015) with 100 bootstrap replicates, finally visualised using PhyD3 (Kreft et al. 2017). To identify the correspondence among the scaffolds with putative gene hits and the chromosome, pSymA and pSymB of the reference *S. meliloti* 2011 genome, multiple alignments between draft genomes of MO56(1) or BO21CC and the genome of the reference strain *S. meliloti* 2011 (RefSeq assembly GCF_000346065.1) were performed with Mauve 2.4.0 with default parameters (Darling et al. 2004).

### 2.3 Statistical Analysis

All statistical data analysis was conducted using the RStudio software (R Core Team 2022). Before each statistical analysis, the Shapiro test was applied to assess the data distribution and One‐way ANOVA on normalised metabolic activities obtained for the strains used in this study under the different concentrations tested (200 mM, 400 mM, 600 mM, 700 mM, 800 mM, 900 mM and 1 M) was carried out with the *agricolae* package. The Scott‐Knott clustering analysis was performed on normalised metabolic activities of strains grown at 400 and 600 mM NaCl with the library *ScottKnott*.

### 2.4 PhenotypeSeeker Analysis and Mapping Procedure

The normalised mean values of metabolic activities of strains assessed in the Biolog experiment at 400 mM and 600 mM NaCl were used as continuous matrices, with values between 0 and 1. The FASTA genome sequences of the 49 *S. meliloti* strains and the obtained matrices were used as input to count all *k*‐mers (set to 13 nucleotides) for each dataset (i.e., for each NaCl concentration). In the filtering step, *k*‐mers found in fewer than two samples or absent from fewer than two samples (‘–min 2–max 2’; default) were discarded. A correction for clonal population structure was applied. For each dataset, the *k*‐mers were then tested for associations with the salt tolerance phenotypes using the weighted Welch two‐sample *t*‐test. *K*‐mers with a *p* value higher than 0.05 were automatically discarded. The genome position of 808 *k*‐mers (*p* value = 1.07e^−14^, 1.11e^−14^ and 1.01e^−13^) obtained at 600 mM NaCl was detected using the R package Biostrings (version 2.54; Pagès et al. 2017). In line with PhenotypeSeeker, only *k*‐mers aligned without mismatches or gaps on the positive or negative strand of the genome reference were considered. Four rules were followed to transform the absolute positions of *k*‐mers into relative ones, based on genomic annotations, described in detail in Bellabarba et al. (2021). The relative positions of *k*‐mers were used to determine the coordinates of predicted protein‐coding sequences (CDS) and to identify regulatory regions associated with these *k*‐mers. Specifically, *k*‐mers with a relative position of 0 were used to extract CDS coordinates, while those with relative positions between −600 and 0 were used to map regulatory regions (Galardini et al. 2011).

### 2.5 Phenotype Microarray Analysis

The *S. meliloti* BO21CC, MO56(1) and RU11/001 strains were inoculated into PM1 and PM2 plates to test growth on different carbon sources, at both 0 mM NaCl and 300 mM NaCl. The complete list of assayed compounds is reported here: https://www.biolog.com/products/metabolic‐characterization‐microplates/microbial‐phenotype/#table. Bacterial strains were grown on TY agar medium (Beringer 1974) and incubated at 30°C for 48 h. Then, colonies were picked up with a sterile cotton swab and suspended in 12 mL of M9 (6 g l^−1^ Na_2_HPO_4_, 3 g l^−1^ KH_2_PO_4_, 0.5 g l^−1^ NaCl, 1 g l^−1^ NH_4_Cl, 4.1 μM biotin, 42 nM CoCl2, 1 mM MgSO_4_, 0.25 mM CaCl_2_, 38 μM FeCl_3_, 5 μM thiamine‐HCl). Cell density was adjusted to OD_600_ = 0.1. M9 media to test the condition of salt‐stress were obtained with the addition of NaCl to a final concentration of 300 mM. Inoculation fluid for each plate and for each condition tested (PM1 and PM2; 0 mM and 300 mM) was obtained by diluting the cellular suspension for each strain 10 times in M9 or M9 supplemented with 300 mM NaCl and adding 1× Dye Mix A (Biolog). Finally, the inoculation fluid was dispensed into PM plates (100 μL/well). In each PM plate, a negative control with no carbon source was inoculated. PM plates were sealed with Breath‐easy gas permeable membrane (Sigma‐Aldrich) to avoid fluid essiccation and incubated without shaking in the Omnilog Reader (Biolog). The OmniLog System recorded colorimetric changes in each well over time, resulting from tetrazolium dye reduction, and generated time‐course curves that reflected the ability of the tested strains to utilise each specific substrate. Readings were taken over 96 h at 30°C every 15‐min intervals.

The data generated by the Omnilog‐PM software (version OM_PM_109M) were filtered, and the areas under the kinetic curves were extracted as indicators of metabolic activity. Based on the maximum values obtained from the negative controls across all plates and conditions tested, carbon sources with an area below 13,000 were considered inactive and excluded from further analyses. Since the Phenotype Microarray analysis is well‐known as a very precise and highly repeatable technique, no technical replicates were carried out (Johnson et al. 2008; Dunkley et al. 2019; Fagorzi et al. 2020).

## 3 Results

### 3.1 Genomic Diversity and Phylogenetic Clustering of *Sinorhizobium meliloti* Strains Indicate a Regional Genomic Distinction of Field Isolates

Twenty‐one *S. meliloti* strains, previously reported (Bellabarba et al. 2023), were characterised by genome sequencing (Table S1). Most of these strains were isolated from the Fleuris site (W. Oran), characterised by a moderately saline soil. Exceptions include strains SO14(2) and SO9(2), which were isolated from the saline soil of Oued Sebbah (W. d'Ain Témouchent), and the strain SS19(1), isolated from the moderately saline soil of Es Senia (Oran). The assemblies showed an average coverage of 209×, with genome size ranging from 6.454.551 bp (MO53(2)) to 6.818.496 bp (MO19) (mean size: of 6.631.753 bp) and an average GC content of 62.2% (range: 62.11% in MO25(1) to 62.31% (MO53(2)) (Table S2)). In addition, 28 well‐characterised reference *S. meliloti* strains from established collections, and whose genomes were available, were included to expand the North Africa field‐isolate panel for subsequent genomic and phenotypic analysis (Table S3).

Digital DNA:DNA hybridization (dDDH) was performed using 16 type strains of closely related *Sinorhizobium/Ensifer* species (Fagorzi et al. 2020). Clustering yielded 16 species groups, with all the query strains assigned to the *S. meliloti* cluster (File S1). Genome Blast Distance Phylogeny (GBDP) confirmed that all the isolates belong to the *S. meliloti* species (Figure S1, File S1) (Fagorzi et al. 2020).

**FIGURE 1:**
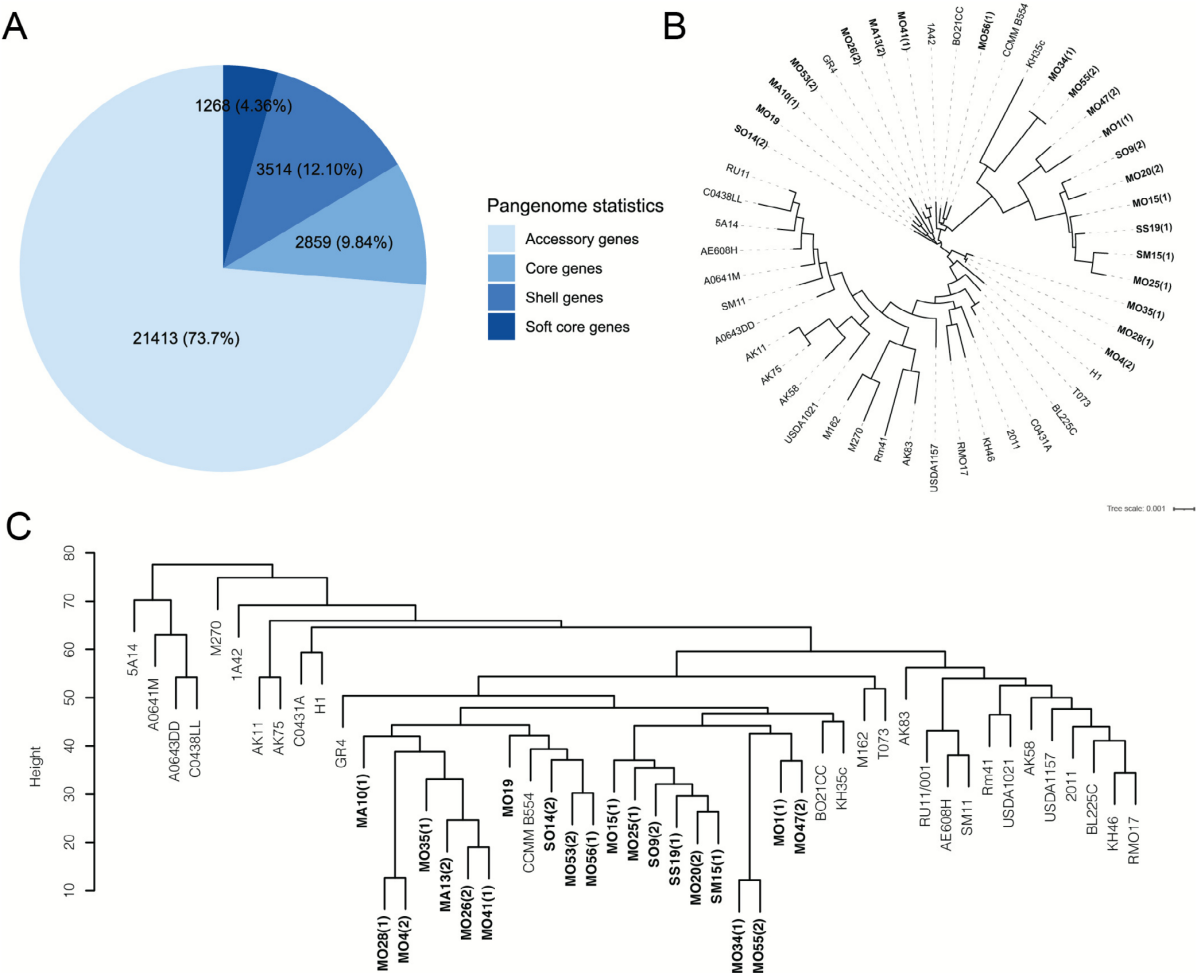
Pangenomic analysis and phylogeny of the *Sinorhizobium meliloti* strains used in this work. (A) Pangenome pie chart showing the number of the core, soft core, shell and accessory genes. (B) Unrooted core gene phylogeny obtained using a concatemer of 2859 genes. (C) Clustering of the dispensable genome based on 26,195 genes. The Algerian field strains were highlighted in bold.

A pangenome analysis of the 49 *S. meliloti* strains identified 29,054 total genes: 2859 core genes (present in all genomes), 1268 soft‐core genes (present in 95%–99% of genomes), 3514 shell genes (present in 15%–94% of genomes), and 21,413 accessory genes (present in < 15% of genomes) (Figure 1A and File S2). An unrooted core gene phylogeny tree of the 49 *S. meliloti* strains and a hierarchical clustering of the 26,195 dispensable genes (accessory, shell and soft‐core) revealed consistent clustering across methods (Figure 1B,C and Figure S1).

A clear distinction between the *S. meliloti* field isolates and most of the other strains was observed; out of 28 *S. meliloti* strains from established collections only 6 (KH35c, GR4, CCMM B554, BO21CC, H1 and 1A42) appear in clusters together with the field isolates, as shown in the phylogram based on GBDP analysis (Figure S1), where the field strains were subdivided in at least two main clusters. Similarly, in the phylogenetic tree based on core genes the number of *S. meliloti* strains from collection closely related to the field ones was reduced to five collection strains (all except H1; Figure 1B); while the field strains were subdivided in four clusters. In the accessory genes hierarchical clustering, the *S. meliloti* field isolates were more compacted in two main clusters with only three collection strains (CCMM B554, BO21CC and KH35c; Figure 1C).

Comparing the strains SM15(1), SO14(2) and SO9(2), originating from the same *M. sativa* cultivar (Seriver) but from a different region (Wileya d'Ain Témouchent), no co‐clustering was observed between SM15(1) and SO14(2) in any of the phylogenomic reconstructions. However, a greater phylogenomic proximity between SM15(1) and S09(2) was observed.

### 3.2 *Sinorhizobium meliloti* Strains Showed a High Phenotypic Variability in Sodium Chloride Tolerance

**FIGURE 2:**
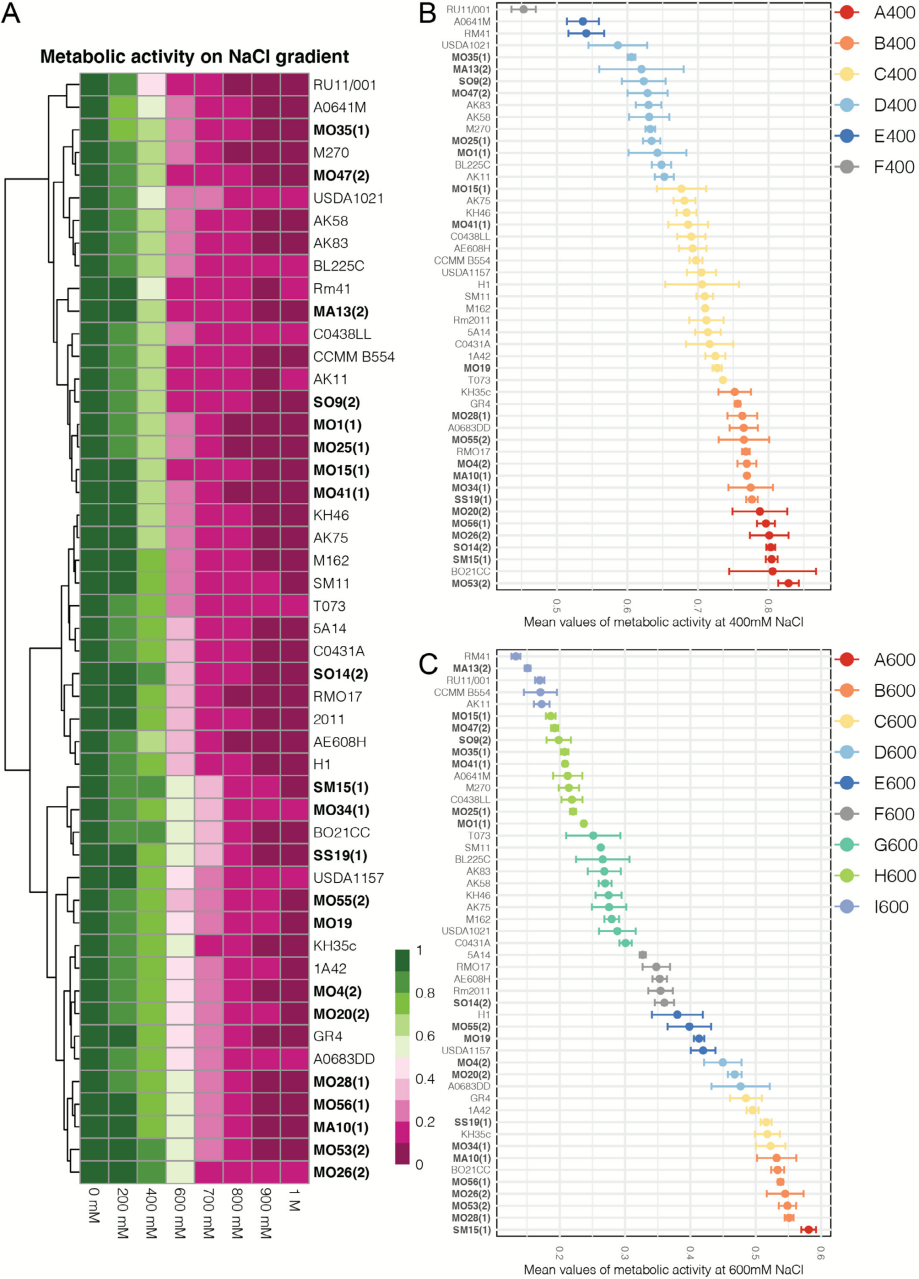
*Sinorhizobium meliloti* strains metabolic activity on increasing concentration of NaCl (0 mM, 200 mM, 400 mM, 600 mM, 700 mM, 800 mM, 900 mM and 1 M). (A) In the Heatmap, the reported data were normalised mean values for each condition. Each condition was performed in triplicate. For each replicate, normalised value was calculated as of the ratio of the AOU on the average value of AOU detected at the concentration of 0 mM of the related strain. Hierarchical clustering with complete linkage of *S. meliloti* strains was reported. Statistical difference among *S. meliloti* strains for salt resistance phenotype at (B) 400 mM NaCl and (C) 600 mM NaCl. Different bars colours and letters indicate statistically significant clustering based on the Scott Knott tests (*p* value < 0.05). For each condition, the dots indicate the mean values, while the vertical lines indicate the standard deviation. For each strain, the dots indicate mean values of normalised metabolic activity, and the vertical lines indicate the standard deviations.

The same panel of 49 *S. meliloti* strains (Tables S1 and S3) was screened for their metabolic activity on seven different concentrations of NaCl (200 mM, 400 mM, 600 mM, 700 mM, 800 mM, 900 mM and 1 M) with respect to a salt‐free control (Figure 2A). Significant differences among strains in metabolic activities were observed at all tested concentrations (Table S4).

All the strains tested thrived well at a concentration of 200 mM NaCl with a Metabolic Activity Reduction (MAR) ranging from 3.7% (MA13(2)) to 21.7% (A0641M). At 800 mM, 900 mM and 1 M NaCl, a great reduction of metabolic activity (more than 80%) was observed for all the strains. The NaCl concentrations of 400 mM, 600 mM and 700 mM showed a more scaled distribution of the strains tested, ranging from 17.2% (MO53(2)) to 54.7% (RU11/001) at 400 mM, from 41.9% (SM15(1)) to 86.7% (Rm41) at 600 mM, and from 62.6% (SM15(1)) to 89.1% (RMO17). The MAR at 400 and 600 mM was distributed in a range between 37.5% and 44.8%, respectively.

Scott‐Knott clustering analysis further resolved these differences. Six groups were identified at 400 mM (Figure 2B). The better performing groups (A400 and B400) with less than 25% MAR comprised 17 strains, 12 of them were field isolates. At 600 mM the best performing groups (MAR < 50%) were A600, B600 and C600 accounting for 12 strains, eight of which were field strains. Differently, at 400 mM NaCl, nine out of 15 low‐performing strains were collection strains, while at 600 mM low performing strains were more equally distributed (7 out of 15) among the collection and field groups. Strains that showed the lowest performance in the presence of NaCl are both collection strains: Rm41 and RU11/001.

Notably, most of the strains showing a higher metabolic activity in NaCl presence are mostly the same at 400 and 600 mM (Figure S2A,B, in orange). Among these, *S. meliloti* SS19(1) was the sole strain isolated from a non‐saline soil (Table S1). All the other high‐performing field strains were isolated from moderately saline soils, while the two strains SO14(2) and SO9(2) isolated from saline soil showed intermediate and low‐performing phenotypes, respectively. Moreover, these two strains were isolated from a different *Medicago sativa* cultivar (Seriver), from which the high‐tolerant strain SM15(1) was obtained.

Considering the distribution of high and low tolerant strains, no direct correlation emerged with their phylogenetic distribution or their accessory genome composition (Figures 1, 2 and Figure S2A,B). Field strains were present in high and low‐performing groups, whereas collection strains were evenly distributed with a predominance for intermediate and low tolerance. Collection strains with the best performance (BO21CC, KH35c and GR4, Figure S2A) were those phylogenetically closer to field strains (Figure 1B,C and Figure S1) when considering both the dispensable and core genome. Conversely, CCMM B554, despite its phylogenetic proximity to high performing strains (e.g., MO56(1) and BO21CC; Figures 1B and Figure S1) and clustering with them in accessory genome space (Figure 1C), exhibited only intermediate or low tolerance at 400 and 600 mM NaCl (Figure 2).

### 3.3 Genome‐Wide Association Highlights Candidate Genes and Regulatory Regions Linked to Salt Tolerance

To identify genes that might be responsible for salt tolerance in the 49 different *S. meliloti* strains, we performed a *k*‐mer‐based GWAS analysis using the tool PhenotypeSeeker (Aun et al. 2018; Bellabarba et al. 2021). As input, we used the genomic sequences of the *S. meliloti* strains used in this study and the phenotypic matrices, gained with the in vitro salt screening. The normalised mean values of metabolic activities detected at 400 and 600 mM NaCl were employed, since these concentrations showed the widest phenotypic variation (Figure 2, Figure S2, and File S3).

The GWAS identified *k*‐mers significantly associated with salt tolerance for both the phenotypic matrices used (i.e., 400 and 600 mM NaCl) (*p* < 0.05, Table S5). Notably, a larger amount of total *k*‐mers significantly associated with salt tolerance (*p* value < 0.05) was obtained for phenotypes measured at 600 mM NaCl (for consistency) (Table S5). Considering the large variability responses to salt stress shown by strains at 600 mM (range of mean metabolic activities = 0.58–0.13), and the larger number of clustering groups gained with the Scott Knott test (Figure 2C, File S3), the dataset of *k*‐mers obtained with the salt tolerance phenotype at 600 mM NaCl was then chosen for the following genomic mapping analysis.

Among the most significant *k*‐mers identified in 600 mM NaCl dataset (*p* value < 5.00e^−08^, File S4), 808 k‐mers (*p* value = 1.07e^−14^, 1.11e^−14^ and 1.01e^−13^, File S4 in bold) were exclusively found in genomes of five strains—MO28(1), MO26(2), MO53(2), MO56(1) and BO21CC—which showed a reduction of metabolic activity lower than 50% at 600 mM and that belonged to Scott Knott cluster B (Figure 2C, File S4 in bold). Top *k*‐mers mapped on the genomes of MO28(1), MO26(2), MO53(2), MO56(1) and BO21CC identified a total of 219 predicted protein‐coding sequences (CDSs), of which 71 orthologous genes (File S5).

The distribution of the candidate function of these orthologous gene hits was uneven among five strains (Figure S3A,C). Most of the gene loci were tracked in *S. meliloti* MO56(1) and BO21CC genomes (isolated from Algeria and Italy, respectively) (Figure S3A,C). A strong enrichment for COG category G (Carbohydrate transport and metabolism) was observed (Figure S3A, Table 1 and Table S5). Additional enriched COG groups were: C (Energy production and conversion), H (Coenzyme transport and metabolism), and K (Transcription) (Figure S3A, Table 1, Tables S5 and S6).

**TABLE 1:** List of functions putatively involved in defining salt resistance at 600 mM NaCl.

Class	COG ID	Annotation	Biological process
C	COG0280	Phosphate acetyltransferase Pta	Acetyl‐CoA biosynthesis
	COG0546	Phosphoglycolate phosphatase Gph	Carbohydrate metabolism (Glycolate biosynthesis)
	COG0644	Electron transfer flavoprotein‐ubiquinone oxidoreductase	Oxidation–reduction process (Electron transport chain)
	COG1012	2‐formylbenzoate dehydrogenase	Aromatic hydrocarbons catabolism
	COG1902	N‐ethylmaleimide reductase NemA	Xenobiotic metabolic process
	COG2055	Putative oxidoreductase	Oxidation–reduction process (Galacturonate utilisation)
E	COG0757	3‐dehydroquinate dehydratase AroQ	Aromatic amino acid biosynthesis
	COG3842	Spermidine/putrescine import ATP‐binding protein PotA	Spermidine ‐ Putrescine transmembrane transport
EGPR	COG0477	Anhydromuropeptide permease	Cell wall organisation, peptidoglycan transport
EH	COG0028	Acetolactate synthase isozyme 3 large subunit	Branched‐chain amino acid biosynthesis
F	COG0105	Nucleoside diphosphate kinase Ndk	Nucleoside triphosphates biosynthesis
	COG0516	Inosine‐5′‐monophosphate dehydrogenase	Purine metabolism
G	COG0296	1,4‐alpha‐glucan branching enzyme GlgB	Glycogen biosynthesis
	COG0366	Trehalose synthase/amylase TreS	Trehalose metabolic process (Glycogen metabolism)
	COG0395	L‐arabinose transport system permease protein	Carbohydrate transmembrane transport (Uptake of arabinooligosaccharides)
	COG1129/COG1172/COG1879	Autoinducer 2 import system ATP‐binding protein LsrA, LsrB and LsrD	AI‐2 transports system (Quorum sensing)
	COG1175	Inner membrane ABC transporter permease protein	Multiple sugar transmembrane transport
	COG1653/COG3839	glycerol‐3‐phosphate‐binding periplasmic protein UgpB and UgpC	Glycerol‐3‐phosphate transmembrane transport
	COG1682	Polysialic acid transport protein	Polysialic acid transmembrane transport
	COG0406/COG2971/COG3957/COG3459	Acid phosphatase Gpm2 (or Fructose‐1,6‐bisphosphatase)/Glucosamine kinase GspK/Xylulose‐5‐phosphate phosphoketolase XpkA/Cyclic beta‐(1,2)‐glucan synthase NdvB	Carbohydrate metabolism
H	COG0001	Glutamate‐1‐semialdehyde 2,1‐aminomutase HemL	Porphyrin‐containing compound metabolism
	COG0161	Putrescine ‐ pyruvate aminotransferase SpuC	Putrescine catabolic process
	COG2227	Ubiquinone biosynthesis O‐methyltransferase UbiG	Ubiquinone biosynthesis (Hyperosmotic salinity response in *E. coli* )
HR	COG0596	3‐oxoadipate enol‐lactonase 2	Aromatic hydrocarbons catabolism (Beta‐ketoadipate pathway)
I	COG0183	Acetyl‐CoA acetyltransferase	Fatty acid degradation
	COG0331/COG1028	Malonyl CoA‐acyl carrier protein transacylase FabD and FabG	Lipid metabolism, fatty acid biosynthesis
	COG0446	Hydrogen cyanide synthase subunit HcnB	Biosynthesis of secondary metabolites/Glycine dehydrogenase (cyanide‐forming) activity
IR	COG2303	Fructose dehydrogenase large subunit	Fructose metabolism
J	COG0024	Methionine aminopeptidase	Proteolysis
	COG0088	50S ribosomal protein L4 RplD	rRNA binding in 50S assembly
	COG0684	4‐carboxy‐4‐hydroxy‐2‐oxoadipate aldolase	Aromatic hydrocarbons catabolism
K	COG0583/COG1737/COG2188	HTH‐type transcriptional regulator PgrR, MurR and NagR	Regulation of peptidoglycan degradation/N‐acetylmuramic acid catabolic process/N‐acetylglucosamine transport and utilisation
	COG1508	RNA polymerase sigma‐54 factor 2 RpoN2	DNA‐binding transcriptional initiation (Nitrogen fixation)/Nitrogen metabolism
L	COG1793	Multifunctional non‐homologous end joining protein LigD	DNA recombination and repair
	COG4973	Tyrosine recombinase XerC	Cell cycle and division/Chromosome partition/DNA integration and recombination
M	COG0438/COG0677	D‐inositol‐3‐phosphate glycosyltransferase/UDP‐N‐acetyl‐D‐glucosamine 6‐dehydrogenase	Cell wall organisation/Lipopolysaccharide biosynthesis
	COG1088	UDP‐glucose 4‐epimerase	Carbohydrate metabolism (Galactose metabolic process)
O	COG0492	Thioredoxin reductase	Cell redox homeostasis (Removal of superoxide radicals)
	COG0526	Thiol‐disulfide oxidoreductase ResA	Cytochrome c maturation
P	COG3158	Low affinity potassium transport system protein Kup	Potassium ion transport
Q	COG1228	Uncharacterized putative protein	Nucleotide metabolism
	COG3321	Narbonolide/10‐deoxymethynolide synthase PikA2, modules 3 and 4	Fatty acid biosynthesis
R	COG0673	Glucose–fructose oxidoreductase Gfo	Carbohydrate metabolism (Sorbitol biosynthesis)
T	COG1409	3′,5′‐cyclic adenosine monophosphate phosphodiesterase CpdA	Cell wall modification
U	COG4789	Invasion protein	Bacterial secretion system
X	COG2826	IS30 family transposase ISRle2	Transposition

*Note:* COG description of gene hits identified by 670 top *k*‐mers (*p* value = 1.07e^−14^, *p* value = 1.11e^−14^ and *p* value = 1.01e^−13^) in the most salt‐resistant strains at concentration of 600 mM (MO28(1), MO26(2), MO53(2), MO56(1) and BO21CC). Function and annotation are reported according to the annotation performed with Prokka in this work.

The most represented orthologous gene groups were related to cell wall organisation and lipopolysaccharide biosynthesis, identifying a putative D‐inositol‐3‐phosphate glycosyltransferase (COG0438; *mshA*) and UDP‐N‐acetyl‐D‐glucosamine 6‐dehydrogenase (COG0677; *wbpA*), but also Carbohydrate and Galactose metabolism as hinted by a putative UDP‐glucose 4‐epimerase (COG10881; *galE1*) (Figure S3A, Table 1). Also, a putative autoinducer 2 import system LsrABD, involved in quorum sensing and AI‐2 transport system, was frequently pinpointed (COG1129, COG1172, COG1879) (Figure S3A, Table 1). Further, orthologous genes codifying for ATP‐dependent DNA ligase LigD (COG1793), engaged in DNA recombination and repair, were repeatedly found in strains MO26(2), MO53(2) and MO56(1) (Figure S3A, Table 1).

**FIGURE 3:**
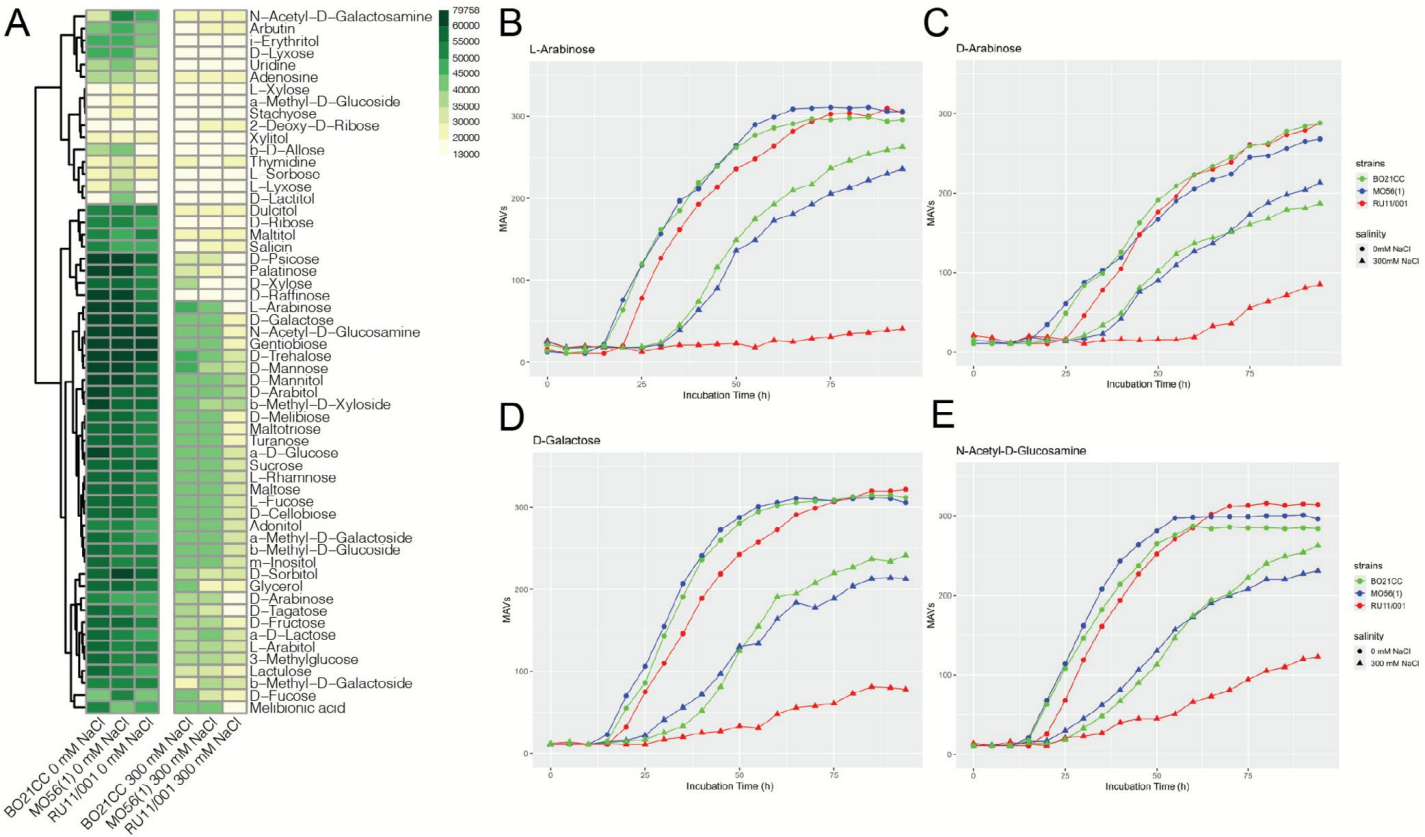
Metabolic activities on carbon sources of *S. meliloti* NaCl‐*R* ^+^ strains (BO21CC and MO56(1)) and NaCl‐R^−^ (RU11/001) strains at 0 mM and 300 mM NaCl. (A) The metabolic activities were expressed and reported as the area of the kinetic curves inferred for each condition tested. Hierarchical clustering with complete linkage of *S. meliloti* strains was reported. Kinetic curves of NaCl‐R^+^ (MO56(1) and BO21CC) and NaCl‐R^−^ (RU11/001) strains on (B) L‐Arabinose, (C) D‐Arabinose, (D) D‐Galactose and (E) N‐Acetyl‐D‐Glucosamine at 0 and 300 mM NaCl. Metabolic activity values are reported on the *y*‐axis, incubation times on the *x*‐axis.

The genes common to MO56(1) and BO21CC were those of the *lsr* operon, *galE1* and *mshA* (Figure S3A and Table 1). However, most of the candidate functions were exclusively identified in one of the two strains (Figure S3A and Table 1). In BO21CC genome, candidate functions possibly involved in salt tolerance were related to carbohydrate metabolism (COG0406, COG3957), specifically, peptidoglycan transport and degradation (COG0477, COG0583), sorbitol biosynthesis (COG0673), glycogen biosynthesis (COG0296) and gluconeogenesis (COG0406) (Figure 3A, Table 1). Interestingly, orthologous genes encoding for trehalose synthase‐amylase TreS involved in trehalose metabolic process (COG0366) and cyclic glucan synthase NdvB involved in cyclic‐glucan synthesis enhanced under hypo‐osmotic conditions were also detected (COG3459) (Figure 3A, Table 1) (Griffitts et al. 2008). Other predicted functions of some of the gene hits identified were related to lipid metabolism and fatty acid biosynthesis (COG0183, COG033, COG1028, COG3321), aromatic hydrocarbons catabolism (COG0684, COG1012, COG0596) oxidation–reduction process (COG2055, COG0644), potassium ion transport (COG3158), purine metabolism (COG0516), aromatic amino acid biosynthesis (COG0757) and Acetyl‐CoA biosynthesis (COG0280) (Figure 3A, Table 1).

In the strain MO56(1) genome, some orthologous gene hits codify functions related to arabinose transport system (COG0395), glycolate biosynthesis (COG0546), fructose metabolism (COG2303), and in general multiple sugar transport (COG1175) were found (Figure 3A, Table 1). Moreover, a putative ABC‐type glycerol‐3‐phosphate transport system UgpBC (COG1653, COG3839) involved in glycerol transmembrane transport was identified (Figure 3A, Table 1). Interestingly, a putative putrescine–pyruvate aminotransferase (COG0161) and an ABC‐type spermidine‐putrescine transport system (COG3842), respectively engaged in putrescine catabolic process and in spermidine–putrescine transmembrane transport, were also tagged in MO56(1) (Figure 3A, Table 1). Other presumed functions of some gene hits identified were related to cell redox homeostasis (COG0492), proteolysis (COG0024), cell wall modification (COG1409), transcriptional regulation of genes involved in N‐acetylglucosamine transport and utilisation (COG2188) and N‐acetylmuramic acid catabolic process (COG1737), nucleoside triphosphate biosynthesis (COG0105) and ubiquinone biosynthesis (COG2227) (Figure 3A, Table 1). Among the CDSs tagged by the best *k*‐mers, predicted protein‐coding sequences (CDSs) with no assigned function were also identified (File S5).

Several gene clusters with the same functional annotation of gene hits were found in the pangenome across strains. Matching the presence‐absence matrix of gene clusters of pangenome analysis (File S2) with *k*‐mers tagged gene hits (File S5), we observed that each *k*‐mers‐tagged gene hit identified in our GWAS belonged to single gene clusters. Gene hits more frequently tagged by *k*‐mers were common to several strains of our populations (Table S8). These gene loci were different allelic forms carrying several SNPs tagged by *k*‐mers that might be responsible for different salt resistance phenotypes. Other gene hits found in our analysis were genes uniquely found in MO56(1) or BO21CC (Table S9), as presence‐absence within our population. Indeed, it is well known that *k*‐mers allow to capture of a large set of genetic variants in a population, as single nucleotide polymorphisms (SNPs) and insertions/deletions (indels) (Lees et al. 2016).

To explore regulatory elements, we considered as promoter sequence the hits mapping within 600 nucleotides upstream of the CDS start (Galardini et al. 2011; Bellabarba et al. 2021). A total of 146 *best k*‐mers pinpointed 71 regulatory regions (File S6) in BO21CC and MO56(1). Most of the regulatory region hits (52) were associated with CDSs with no assigned function (File S6). Other were associated with genes encoding proteins whose functions were related to carbohydrate and galactose metabolism (COG3957, COG0406, COG0546, COG1088), putrescine catabolism (COG0161) DNA recombination and repair (COG1793), cell wall organisation (COG0381), lipopolysaccharide biosynthesis (COG0677) and aromatic hydrocarbons catabolism (COG0757, COG0596, COG0446) (Figure S3B; Tables S5 and S7).

### 3.4 Replicon‐Level Distribution of *k*‐Mer–Tagged Loci Associated With Salt Tolerance

To uncover the distribution of *k*‐mers along replicons and their contribution in salt tolerance (i.e., replicons where *k‐mers*‐tagged gene hits and regulatory regions were located) multiple alignment was performed with the draft genomes of MO56(1) and BO21CC to the genome of the reference strain *Sinorhizobium meliloti* 2011.

In both strains, most of the genes associated with the salt tolerance mechanism were found on scaffolds corresponding to the 2011 chromosome replicon (Figure S4A,B). In MO56(1), 62.5% of genes were located on scaffolds that corresponded to the 2011 chromosome replicon and pSymB megaplasmid (31.25% each, Figure S4A), 18.75% of genes on pSymA, while the remaining 18.75% on scaffolds showing no clear correspondence (Figure S4A). In BO21CC, 34.78% of gene loci were pinpointed on scaffolds corresponding to the chromosome, while only 27.75% and 8.70% of loci were found on scaffolds corresponding respectively to pSymA and pSymB (Figure S4B). Concerning the regulatory region, scaffolds with a higher number of gene loci for salt tolerance were related to the corresponding chromosome in MO56(1) (33.3%) and BO21CC (42.9%) (Figure S4C,D).

### 3.5 Salt‐Tolerant Strains Showed Broader Abilities in Carbon Sources Exploitation

To ascertain the involvement of carbohydrates in salt‐stress tolerance mechanisms, the metabolic activities of salt‐tolerant and salt‐sensitive strains were recorded on different carbon sources both in presence and absence of salt (0 mM NaCl and 300 mM NaCl). To avoid excessive stress to cells, the salt stress condition was tested at the concentration of 300 mM NaCl since the Phenotype Microarray analysis on PM1 was conducted in M9 minimal medium without the addition of carbon sources (Checcucci et al. 2018). The strain BO21CC and the strain MO56(1) were selected as NaCl resistant strains (NaCl‐R^+^) (belonging to groups A400 and B600; Figure 2B,C, Figure S2 and File S4), while RU11/001 was chosen as NaCl‐*R* ^−^ strain (groups F400 and I600; Figure 2B,C, Figure S2 and File S4).

The salt tolerance threshold was considered as the maximum area under the kinetic curves of the negative controls across all tested conditions (Table S10). Carbon sources with an area below 13,000 AOU were rejected on the basis that such responses reflected a lack of metabolic activity by the strains on those carbon sources (File S7).

Overall, NaCl‐*R* ^+^ strains MO56(1) and BO21CC showed broader catabolic capacities than the NaCl‐*R* ^−^ strain RU11/001 (Table S11). In salt‐free conditions, out of 190 sources tested, the strains MO56(1) and BO21CC utilised 108 and 94 carbon sources respectively, while RU11/001 only utilised 86 (Table S11). In the presence of 300 mM NaCl, the spectrum of used carbon sources decreased for all strains: 61 and 58 sources for MO56(1) and BO21CC, and 53 sources for RU11/001 (about 57%, 62% and 62% of sources normally catabolised, respectively) (Table S11). According to the different classes, in the presence and absence of NaCl, most sources used as carbon sources by all three strains were carbohydrates, followed by carboxylic acids and amino acids (Table S11, Figure 3A and Figure S5). Instead, all three strains do not utilise amine and amide as carbon sources (Table S11, Figure S5).

Focusing on carbohydrate metabolism, at 0 mM NaCl the NaCl‐*R* ^+^ strains showed higher metabolic activities than the NaCl‐*R* ^−^ strain and were able to grow on a wider variety of carbohydrates (Figure 3A). Despite the general reduction of metabolic activities in all strains at 300 mM NaCl, differences in growth abilities in presence of salt were more pronounced between the NaCl‐*R* ^+^ and NaCl‐R^−^ strains for most of the carbohydrate sources (Figure 3A). Considering common carbon sources (48) in tolerant and non‐tolerant strains we observed that for half of them (25 compounds) the salt‐tolerant strains retain a high metabolic activity in presence of 300 mM NaCl (Figure 3A). Specifically, these compounds were L‐arabinose, D‐galactose, N‐acetil‐D‐glucosamine, Gentibiose, D‐thehalose, D‐mannose, D‐mannitol, D‐arabitol, β‐methyl‐D‐xyloside, D‐melibiose, Maltotriose, Turanose, α‐D‐glucose, Sucrose, L‐rhamnose, Maltose, L‐fucose, D‐cellobiose, Adonitol, α‐methyl‐D‐galactoside, β‐methyl‐D‐glucoside, m‐inositol, D‐Arabinose, D‐tagatose, Melibionic acid.

Under salt stress, the extent of decrease in metabolic activities varied across the different carbohydrates tested (Figure 3). In most cases, although all the strains showed comparable kinetic curves at 0 mM NaCl, when grown at 300 mM, the growth kinetics decreased for the NaCl‐*R* ^−^ strain. This indicated that salt stress affected the catabolic capacities of NaCl‐*R* ^−^ for those sources (as for α‐D‐glucose, Sucrose, D‐cellobiose, Glycerol, D‐trehalose and Turanose, and others reported in Supporting Informations—File S8). This trend is particularly evident in growth kinetics for L‐arabinose, D‐arabinose, D‐galactose and N‐acetyl‐D‐Glucosamine (Figure 3B–E) and D‐tagatose (File S8), where under salt stress the NaCl‐*R* ^−^ greatly lost its metabolic activities compared to NaCl‐R^+^, suggesting that the catabolism of these sources was probably implicated/involved in salt tolerance.

## 4 Discussion

Climate change is driving significant shifts in global agriculture, particularly in regions affected by increasing salinity in soils. In saline soils, rhizobia can be greatly affected in terms of survival and efficiency in establishing the symbiosis (Bellabarba et al. 2019). Therefore, the major challenge for future agricultural applications is to improve their capacities to adapt to harsh conditions (Viti et al. 2021). *S. meliloti* is agriculturally important and serves as a model for dissecting rhizobial diversity and nitrogen fixation. This species displays a high level of genomic diversity, with various strains demonstrating unique stress tolerance traits that can impact their interaction with the host plant (Bellabarba et al. 2019). Here we elucidated the putative genetic and metabolic bases of salt tolerance in *S. meliloti*, obtaining findings that highlighted a complex interplay between genetic diversity and adaptive features, underscoring the multifactorial nature of salt tolerance in *S. meliloti* .

Our comparative genomic analyses revealed that the field strains formed a distinct cluster, both in core‐gene phylogeny and accessory genome composition. This pattern indicates a clear geography‐related genetic divergence, which appears to have shaped *S. meliloti* populations through environmental pressures and evolutionary events, as previously reported by Toro et al. (2017).

A few collection strains, however, clustered with the field isolates, notably CCMM‐B554, BO21CC and KH35c, originating from Morocco, Italy, and France, respectively (Galardini et al. 2013; Sugawara et al. 2013; Kazmierczak et al. 2017). Despite the genetic divergence, which may reflect regional adaptation or historical separation among these bacterial populations, the phenotypic analysis of salt tolerance did not mirror the genomic clustering. As a result, the geographically related pattern appeared less pronounced. Although most strains tolerant of high salt stress belonged to the field isolates group, the latter showed a heterogeneous distribution, with several strains exhibiting an intermediate or weak phenotype, as well as the collection strains. Otherwise, all the other high‐performing strains were isolated from moderately saline soils, including SO14(2) and SO9(2), those showing the respectively intermediate or low‐performing phenotype. This implies that salt tolerance is not solely determined by the phylogenetic lineage of the strains but rather by the presence of specific adaptive mechanisms. It is probable that distinct adaptation mechanisms have developed in different *S. meliloti* populations to impart salt tolerance in response to the wide range of soil conditions (with differing salinity levels) inhabited by these bacteria. The lack of association between phylogenetic distance and salt tolerance underlines the flexibility of rhizobia adaptation to environmental stress, possibly due to horizontal gene transfer events, but also to convergent evolution (Cardoso et al. 2015). Our findings confirm and further support previous reports indicating that salt‐tolerant strains are not necessarily derived from saline soils (Talebi et al. 2008; Thrall et al. 2009).

Phenotypic assays indicated that the metabolic activity of the *S. meliloti* strains tested remained high up to 200 mM NaCl, but a significant decline was observed at concentrations ranging above 800 mM. The optimal tolerance range of the strains was determined among 400 and 700 mM NaCl, where distinct variations were evident. Similar findings have been previously reported: *S. meliloti* and *S. medicae* isolates from marginal soils affected by salinity and drought in arid and semi‐arid regions of Morocco exhibited a broad tolerance range, from 171 to 1711 mM NaCl (Elboutahiri et al. 2010).

Considering this heterogenous pattern of NaCl tolerance and the lack of complete coincidence between genome‐wide relatedness, geographical origin and salt tolerance, we applied GWAS to pinpoint the genetic determinants associated with salt tolerance features. The analysis at 600 mM identified numerous *k*‐mers significantly related to salt tolerance. Previous studies suggested that bacterial adaptation to environmental stressors, such as salinity, could be driven by horizontal gene transfer (HGT), regulatory changes, and polygenic interactions (Cardoso et al. 2015).

Most of the *k*‐mers generated in GWA analysis pinpointed gene loci located on the two strains BO21CC and MO56(1). The reduced number of *k*‐mers‐tagged genes on other strains (MO28(1), MO26(2), MO53(2)) or even the lack of identifying *k*‐mers‐tagged genes on other salt‐resistant strains is possibly due to the cutoff used for the *k*‐mers selection, suggesting that more genes could be linked to this phenotype. Moreover, we observed that most of the gene loci identified in our analysis were genes allelic versions enriched in SNPs of orthologous genes common to other strains with different salt tolerance phenotypes. This evidence might indicate that our GWAS association was mainly driven by SNPs tagged by *k‐*mers, and that these SNPs are possibly differentiating salt‐tolerant and sensitive strains.

Many significant *k*‐mers were associated with the *lsr* operon that is possibly associated to an AI‐2 quorum sensing (QS) system, the *galE1* gene which is encoding for a UDP‐glucose 4‐epimerase and *mshA* encoding for a D‐inositol‐3‐phosphate glycosyl transferase. The coexistence of multiple orthologous genes for *galE1*, *mshA* and *lsr* within our population and multiple genic allelic variants within each orthologous group suggests that some of them may have evolved strain‐specific functions through SNPs diversification. However, considering their original function these loci are associated to mechanisms possibly relevant to salt adaptation. The function of the AI‐2 QS system in bacterial communication, biofilm formation, and stress adaptation is well known (Zhang et al. 2022). QS‐mediated control may help coordinate gene expression in response to osmotic stress, by establishing a protective milieu that lessens direct exposure to osmotic stress (Zhu et al. 2021). In *E. coli*, AI‐2‐promotes cells auto aggregation which in turn increases stress tolerance (Laganenka et al. 2016) Moreover, biofilm development has been demonstrated to improve bacterial viability in high‐salinity environments (Haque et al. 2022). Notable is also the role of GalE: it contributes to exopolysaccharides (EPS) and lipopolysaccharides (LPS) biosynthesis, which support cell surface integrity and resilience to osmotic stress (Culligan et al. 2012). Exopolysaccharides are particularly important for preserving cell hydration and creating protective biofilms that can lessen the impact of elevated salt concentrations. Modifications to the bacterial cell envelope and/or extracellular matrix composition may be significant adaptive mechanisms in saline conditions. GalE is connected to the interconversion of UDP‐galactose and UDP‐glucose, besides its role in membrane modification UDP‐glucose is also a key player in salt stress tolerance, as it is a precursor of the osmoprotectant trehalose (Giaever et al. 1988). Interesting queries concerning the regulatory networks controlling stress responses in *S. meliloti* are brought up by the possible synergy between AI‐2 QS and *galE1* in salt adaptation. There may be a connection between quorum sensing and the increase of *galE1*‐mediated polysaccharide synthesis, as AI‐2 QS has been linked to the regulation of polysaccharide biosynthesis and biofilm formation in other bacterial species (Zhang et al. 2022).

Our GWAS also identified hits in genes associated with biological process as ‘cell wall organization, lipopolysaccharide biosynthesis’ and ‘peptidoglycan transport’ consistent with prior evidence that cell wall structure influences survival under osmotic stress (Vriezen et al. 2007). The survival of rhizobia strains during desiccation and rewetting is influenced by cell wall fragility, with weak points identified at flagellar emergence sites (Bushby and Marshall 1977). Structural alterations in the cell wall have been observed under osmotic and salt stress, including the downregulation of the *murACG* operon (Domínguez‐Ferreras et al. 2006) and the involvement of genes associated with cell wall in survival during desiccation, such as a putative D‐Ala–D‐Ala ligase (Vriezen et al. 2006). The salt‐tolerant *Sinorhizobium* sp. BL3 strain exhibited a constant expression of the protein synthesis‐related genes ortholog to *SMc00868* (ATP synthase subunit B) and *SMc02942* (peptidoglycan‐associated lipoprotein precursor) under high salinity conditions compared to normal conditions (Tanthanuch et al. 2010). Under salt stress, predominant changes were observed in LPS I compared to LPS II in both the wild‐type *Rhizobium* sp. ST1, a fast‐growing, salt‐sensitive strain with a narrow host range isolated from *Cajanus cajan* nodules, and its *Tn5* mutant, which is exopolysaccharide‐deficient (*exo* ^*−*^) (Unni and Rao 2001).

Furthermore, the accumulation of compatible solutes, such as certain carbohydrates, is a well‐known and widespread strategy to counter osmotic stress (da Costa et al. 1998). Early reports on disaccharides as osmoprotectants and glucan biosynthesis in *S. meliloti* are present (Ingram‐Smith and Miller 1998; Gouffi et al. 1999). Glucan biosynthesis is stimulated by neutral solutes, such as trehalose and glycine betaine (Ingram‐Smith and Miller 1998). The production of cyclic glucans by *ndvA* and *ndvB* genes decreases with increasing osmolarity, and the expression of those genes is normally downregulated when osmolarity increases, suggesting a role in NaCl‐mediated survival during desiccation (Vriezen et al. 2007). Normally, trehalose accumulates in stressed bacteria, particularly in osmo‐stressed rhizobia, where it helps protect against desiccation by preserving membrane integrity during both drying and rehydration. Its presence may account for the enhanced desiccation survival observed during the stationary phase and when cells are exposed to NaCl (Vriezen et al. 2007). Actually, several *k*‐mers were associated with genes linked to trehalose: a transport system permease in MO56(1) and a synthase in BO21CC. On the contrary, no hits on the *bet* system, involved in glycine betaine transport and biosynthesis, known to contribute to osmotic tolerance in *S. meliloti* , were found (Boscari et al. 2006). Concerning the *bet* system, the genes of this system have been found to show variability in a collection of salt‐tolerant strains of *S. meliloti* from the north Aral Sea region (Muntyan and Roumiantseva 2022), including the presence of additional copies of some of them on the pSymA megaplasmid. However, *bet* genes are involved not only in osmotolerance but are also required for symbiotic interactions in bacteroid osmoprotection (Boscari et al. 2006). It is not surprising that no hits were detected in our GWAS since their variability could also be linked to different host plant genotypes or neutral variation. Another gene common to both BO21CC and MO56(1) was *mshA*, which catalyses the transfer of an N‐acetyl‐glucosamine moiety to 1D‐myo‐inositol 3‐phosphate in the pathway of mycothiol biosynthesis. It has been shown that mycothiol is crucial for the maintenance of intracellular redox balance in *Mycobacterium smegmatis* (Miller et al. 2024); however, other genes for mycothiol biosynthesis do not seem to be present in these two strains. Therefore, it is reasonable to take into account that this gene may have a different and still unknown function in *S. meliloti* .

Plants secrete a wide array of different carbon compounds in the rhizosphere (amino acids, sugars, organic acids, and some secondary metabolites), releasing up to 20%–40% of their photosynthetic fixed C through root exudates which play different roles in bacteria metabolisms (Badri et al. 2009; Pini et al. 2017). Many genes' hits were associated with COG categories related to carbohydrate metabolism. Indeed, it is well known that under growth‐limiting conditions, carbon sources are stored as glycogen, which may help restore cell volume following osmotic shock. This is further supported by the observation that genes involved in glycogen metabolism, such as *glgA2, glgB2* and *glgX*, are expressed at higher levels when exposed to osmotic stress, suggesting that glycogen accumulates during such stress (Vriezen et al. 2007). As previously discussed, under saline stress, cells preferentially accumulate compatible solutes, such as carbohydrates (e.g., sucrose, trehalose, maltose) and amino acids (e.g., glutamate, proline). While some solutes are absorbed from the environment, making transmembrane transport systems essential, others, such as sucrose and trehalose, are primarily synthesised de novo and may be accumulated not just as osmotic stabilisers but also to prevent starvation (Vriezen et al. 2007).

The role of carbon sources in *Sinorhizobium* salt tolerance was then further investigated using Phenotype Microarray. Salt‐tolerant strains showed broader capabilities in exploiting the number of possible carbon sources. We observed that the salt‐tolerant strains retain a high metabolic activity in the presence of 300 mM NaCl for half of the carbon sources, including L‐arabinose, D‐galactose, sucrose, trehalose and sugar alcohols—many of which are abundant in *M. sativa* root exudates (Koo et al. 2005; Ramachandran et al. 2011; Wang et al. 2019). These sugars not only sustain bacterial growth under stress but also feed into EPS production and biofilm formation, further enhancing survival. However, salinity affects the growth of tolerant and sensitive strains to different extents. Therefore, it remains difficult to determine whether the limited differences observed in sugar utilisation truly reflect a role in saline tolerance, or instead it's merely a consequence of differential growth inhibition under osmotic stress. Furthermore, a number of exogenous sugars, especially disaccharides, function as osmoprotectants by promoting the build‐up of glutamate and other suitable solutes without being catabolised. The interpretation of Phenotype Microarray data may be complicated by strain‐specific osmoprotective mechanisms that affect metabolic profiles under salt.

In summary, our study reveals a high genetic and phenotypic variation of *S. meliloti* with respect to salt stress. GWAS highlighted that multiple pathways (possibly synergistic), such as QS‐AI2 mediated regulation and cell surface modifications (*galE1*), may then contribute to salt tolerance in *S. meliloti* . These discoveries not only advance our knowledge of microbial adaptability to osmotic stress, but they may also have applications in enhancing the robustness of the symbiotic relationship between legumes and rhizobia in saline soils.

These insights provide a framework for selecting or engineering rhizobial strains with enhanced salt tolerance (linked to carbohydrate metabolism, cell wall modification and QS‐regulated stress responses) as tailor‐suited bioinoculants for saline environments.

## Author Contributions

**Agnese Bellabarba:** conceptualization, data curation, formal analysis, investigation, methodology, validation, visualisation, writing – original draft, writing – review and editing. **Camilla Fagorzi:** data curation, formal analysis, investigation, methodology, visualisation, writing – original draft, writing – review and editing. **Giovanni Bacci:** data curation, formal analysis, investigation, methodology, software, writing – review and editing. **Francesca Decorosi:** data curation, formal analysis, investigation, methodology, writing – original draft, writing – review and editing. **Alice Checcucci:** writing – original draft, writing – review and editing. **Gaio Cesare Pacini:** funding acquisition, writing – review and editing. **Abdelkader Bekki:** funding acquisition, writing – review and editing. **Amina El Hadj Mimoune:** investigation, writing – review and editing. **Khalid Azim:** funding acquisition, writing – review and editing. **Majida Hafidi:** funding acquisition, writing – review and editing. **Alessio Mengoni:** conceptualization, funding acquisition, resources, supervision, writing – review and editing. **Francesco Pini:** conceptualization, supervision, validation, visualisation, writing – original draft, writing – review and editing. **Carlo Viti:** conceptualization, funding acquisition, project administration, resources, supervision, writing – review and editing.

## Funding

This work was supported by Partnership for Research and Innovation in the Mediterranean Area.

## Conflicts of Interest

The authors declare no conflicts of interest.

## Supporting information


**Data S1:** mbt270304‐sup‐0001‐DataS1.xlsx.

**Appendix S1:** mbt270304‐sup‐0002‐supinfo.docx.

**Figure S1:** ‘Phylogram’: Tree inferred with FastME from GBDP distances calculated from genome sequences. The branch lengths were scaled in terms of GBDP distance formula *d*_*5*_. The numbers above branches are GBDP pseudo‐bootstrap support values > 60% from 100 replications, with an average branch support of 28.2%. Algerian strains were highlighted in bold.

**Figure S2:** mbt270304‐sup‐0004‐FigureS2.png. *Sinorhizobium meliloti* strains with high salt resistance at 400 and 600 mM NaCl. (A) Venn diagram between the groups characterised by a higher AOU at 400 mM (groups A and B) and 600 mM NaCl (Groups A, B and C). (B) Venn diagram with the top AOU groups at 400 mM (groups A) and 600 mM NaCl (Groups A and B). Algerian strains were highlighted in bold.

**Figure S3:** Functions associated with the salt‐resistant phenotype. Frequency of candidate functions of gene hits (A) and regulatory regions (B) identified by *best k*‐mers in the most salt‐resistant strains. The frequency of candidate functions reported as COG annotations (rows) in each strain (columns) is represented by grayscale shades. In the upset plots, (C) the number of shared functions of gene hits and (D) shared functions of regulatory regions, in each and different combinations of strains are reported.

**Figure S4:** Distribution of scaffolds hit by *best k*‐mers among different replicons in *Sinorhizobium meliloti* MO56(1) and BO21CC through the alignment with the genome of *S. meliloti* 2011. Distribution of *k*‐mers‐cointaining scaffolds of MO56(1) (A&C) and BO21CC (B & D) mapped on the genome of 2011 (RefSeq assembly GCF_000346065.1) referring to both gene hits (A, B) and regulatory region hits (C, D). Data are reported as percentage on the total number of considered scaffolds in each dataset. Scaffold reported as Unknown could not be aligned on the reference genome.

**Figure S5:** Metabolic activities on carboxylic acid, amino acid and other sources of *S. meliloti* strains BO21CC, MO56(1) and RU11/001 at 0 and 300 mM NaCl. Carboxylic acid (A), amino acid (C) and others (B) were used as carbon sources. Other sources refer to alcohols, ester & fatty acids, polymers, amine & amide. The metabolic activities were expressed and reported as area of the kinetic curves for each condition. Hierarchical clustering with complete linkage of *S. meliloti* strains was reported.

**File S1:** DDH table.
**File S2:** The gene presence and absence matrix of *S. meliloti* pangenome.

**File S3:** Grouping of mean values of normalized metabolic activity for each *S. meliloti* strain at 200 mM, 400 mM, 600 mM, 700 mM, 800 mM, 900 mM and 1 M NaCl. Different letters indicate statistically significant groupings based on the Scott Knott tests (*p* < 0.05).

**File S4:** List of top *k*‐mers for (a) 400 mM (b) 600 mM.

**File S5:** Genes hits identified by the best *k*‐mers at 600 mM dataset.

**File S6:** Regulatory region hits identified by the best *k*‐mers for 600 mM dataset.

**File S7:** Metabolic activity values of strains NaCl‐R+ (BO21CC and RU11/001) and NaCl‐R‐ (RU11/001) in presence and absence of NaCl (0 mM and 300 mM NaCl) on unused sources with an area smaller than 13000 AOU on PM1 and PM2 plates.

**File S8:** Metabolic activity values of strains NaCl‐R+ (BO21CC and RU11/001) and NaCl‐R‐ (RU11/001) in presence and absence of NaCl (0 mM and 300 mM NaCl) on some carbon sources putatively involved in salt resistance.

## Data Availability

Genomic sequences of this project have been deposited at GenBank under the BioProject ID PRJNA853716.

## References

[mbt270304-bib-0001] Andras, J. P. , P. D. Fields , L. D. Pasquier , M. Fredericksen , and D. Ebert . 2020. “Genome‐Wide Association Analysis Identifies a Genetic Basis of Infectivity in a Model Bacterial Pathogen.” Molecular Biology and Evolution 37: 3439–3452. 32658956 10.1093/molbev/msaa173 PMC7743900

[mbt270304-bib-0002] Aun, E. , A. Brauer , V. Kisand , T. Tenson , and M. Remm . 2018. “A k‐Mer‐Based Method for the Identification of Phenotype‐Associated Genomic Biomarkers and Predicting Phenotypes of Sequenced Bacteria.” PLoS Computational Biology 14: e1006434. 30346947 10.1371/journal.pcbi.1006434 PMC6211763

[mbt270304-bib-0003] Badri, D. V. , T. L. Weir , D. van der Lelie , and J. M. Vivanco . 2009. “Rhizosphere Chemical Dialogues: Plant‐Microbe Interactions.” Current Opinion in Biotechnology 20: 642–650. 19875278 10.1016/j.copbio.2009.09.014

[mbt270304-bib-0004] Bellabarba, A. , G. Bacci , F. Decorosi , et al. 2021. “Competitiveness for Nodule Colonization in *Sinorhizobium meliloti*: Combined In Vitro—Tagged Strain Competition and Genome‐Wide Association Analysis.” mSystems 6: e0055021. 34313466 10.1128/mSystems.00550-21 PMC8407117

[mbt270304-bib-0005] Bellabarba, A. , F. Decorosi , C. Fagorzi , et al. 2023. “Salt Stress Highlights the Relevance of Genotype × Genotype Interaction in the Nitrogen‐Fixing Symbiosis Between *Sinorhizobium meliloti* and Alfalfa.” Soil Systems 7: 112.

[mbt270304-bib-0006] Bellabarba, A. , C. Fagorzi , G. C. DiCenzo , F. Pini , C. Viti , and A. Checcucci . 2019. “Deciphering the Symbiotic Plant Microbiome: Translating the Most Recent Discoveries on Rhizobia for the Improvement of Agricultural Practices in Metal‐Contaminated and High Saline Lands.” Agronomy 9: 529.

[mbt270304-bib-0007] Beringer, J. E. 1974. “R Factor Transfer in *Rhizobium leguminosarum* .” Journal of General Microbiology 84: 188–198. 4612098 10.1099/00221287-84-1-188

[mbt270304-bib-0008] Bertrand, A. , C. Dhont , M. Bipfubusa , F. P. Chalifour , P. Drouin , and C. J. Beauchamp . 2015. “Improving Salt Stress Responses of the Symbiosis in Alfalfa Using Salt‐Tolerant Cultivar and Rhizobial Strain.” Applied Soil Ecology 87: 108–117.

[mbt270304-bib-0009] Biondi, E. G. , E. Tatti , D. Comparini , et al. 2009. “Metabolic Capacity of *Sinorhizobium* (*Ensifer*) *meliloti* Strains as Determined by Phenotype MicroArray Analysis.” Applied and Environmental Microbiology 75: 5396–5404. 19561177 10.1128/AEM.00196-09 PMC2725449

[mbt270304-bib-0011] Bolger, A. M. , M. Lohse , and B. Usadel . 2014. “Trimmomatic: A Flexible Trimmer for Illumina Sequence Data.” Bioinformatics 30: 2114–2120. 24695404 10.1093/bioinformatics/btu170 PMC4103590

[mbt270304-bib-0012] Boncompagni, E. , M. Østerås , M.‐C. Poggi , and D. le Rudulier . 1999. “Occurrence of Choline and Glycine Betaine Uptake and Metabolism in the Family Rhizobiaceae and Their Roles in Osmoprotection.” Applied and Environmental Microbiology 65: 2072–2077. 10224003 10.1128/aem.65.5.2072-2077.1999 PMC91300

[mbt270304-bib-0013] Boscari, A. , G. Van de Sype , D. Le Rudulier , and K. Mandon . 2006. “Overexpression of BetS, a *Sinorhizobium meliloti* High‐Affinity Betaine Transporter, in Bacteroids From *Medicago sativa* Nodules Sustains Nitrogen Fixation During Early Salt Stress Adaptation.” MPMI 19: 896–903. 16903355 10.1094/MPMI-19-0896

[mbt270304-bib-0015] Bushby, H. V. A. , and K. C. Marshall . 1977. “Some Factors Affecting the Survival of Root‐Nodule Bacteria on Desiccation.” Soil Biology and Biochemistry 9: 143–147.

[mbt270304-bib-0017] Cardoso, P. , R. Freitas , and E. Figueira . 2015. “Salt Tolerance of Rhizobial Populations From Contrasting Environmental Conditions: Understanding the Implications of Climate Change.” Ecotoxicology 24: 143–152. 25318616 10.1007/s10646-014-1366-8

[mbt270304-bib-0018] Chakraborty, S. , and J. M. Harris . 2022. “At the Crossroads of Salinity and Rhizobium‐Legume Symbiosis.” Molecular Plant‐Microbe Interactions 35: 540–553. 35297650 10.1094/MPMI-09-21-0231-FI

[mbt270304-bib-0019] Checcucci, A. , G. C. DiCenzo , V. Ghini , et al. 2018. “Creation and Characterization of a Genomically Hybrid Strain in the Nitrogen‐Fixing Symbiotic Bacterium *Sinorhizobium meliloti* .” ACS Synthetic Biology 7: 2365–2378. 30223644 10.1021/acssynbio.8b00158

[mbt270304-bib-0020] Chien, C.‐T. , J. Maundu , J. Cavaness , L.‐M. Dandurand , and C. S. Orser . 1992. “Characterization of Salt‐Tolerant and Salt‐Sensitive Mutants of *Rhizobium leguminosarum* Biovar *Viciae* Strain C1204b.” FEMS Microbiology Letters 90: 135–140. 10.1016/0378-1097(92)90617-w 1537541

[mbt270304-bib-0021] Culligan, E. P. , R. D. Sleator , J. R. Marchesi , and C. Hill . 2012. “Functional Metagenomics Reveals Novel Salt Tolerance Loci From the Human Gut Microbiome.” ISME Journal 6: 1916–1925. 22534607 10.1038/ismej.2012.38 PMC3446828

[mbt270304-bib-0022] da Costa, M. S. , H. Santos , and E. A. Galinski . 1998. “An Overview of the Role and Diversity of Compatible Solutes in Bacteria and Archaea.” Advances in Biochemical Engineering/Biotechnology 61: 117–153. 9670799 10.1007/BFb0102291

[mbt270304-bib-0023] Darling, A. C. E. , B. Mau , F. R. Blattner , and N. T. Perna . 2004. “Mauve: Multiple Alignment of Conserved Genomic Sequence With Rearrangements.” Genome Research 14: 1394–1403. 15231754 10.1101/gr.2289704 PMC442156

[mbt270304-bib-0024] Daud, M. , H. Qiao , S. Xu , X. Hui , M. Adil , and Y. Lu . 2025. “Understanding Abiotic Stress in Alfalfa: Physiological and Molecular Perspectives on Salinity, Drought, and Heavy Metal Toxicity.” Frontiers in Plant Science 16: 1627599. 40822732 10.3389/fpls.2025.1627599 PMC12350362

[mbt270304-bib-0025] diCenzo, G. C. , and T. M. Finan . 2017. “The Divided Bacterial Genome: Structure, Function, and Evolution.” Microbiology and Molecular Biology Reviews 81: e00019‐17. 28794225 10.1128/MMBR.00019-17 PMC5584315

[mbt270304-bib-0026] Domínguez‐Ferreras, A. , R. Pérez‐Arnedo , A. Becker , J. Olivares , M. J. Soto , and J. Sanjuán . 2006. “Transcriptome Profiling Reveals the Importance of Plasmid pSymB for Osmoadaptation of *Sinorhizobium meliloti* .” Journal of Bacteriology 188: 7617–7625. 16916894 10.1128/JB.00719-06 PMC1636257

[mbt270304-bib-0027] Dunkley, E. J. , J. D. Chalmers , S. Cho , T. J. Finn , and W. M. Patrick . 2019. “Assessment of Phenotype Microarray Plates for Rapid and High‐Throughput Analysis of Collateral Sensitivity Networks.” PLoS One 14: e0219879. 31851668 10.1371/journal.pone.0219879 PMC6919586

[mbt270304-bib-0028] Elboutahiri, N. , I. Thami‐Alami , and S. M. Udupa . 2010. “Phenotypic and Genetic Diversity in *Sinorhizobium meliloti* and *S. medicae* From Drought and Salt Affected Regions of Morocco.” BMC Microbiology 10: 15. 20089174 10.1186/1471-2180-10-15 PMC2823721

[mbt270304-bib-0029] Elsheikh, E. A. E. , and M. Wood . 1995. “Nodulation and N2 Fixation by Soybean Inoculated With Salt‐Tolerant Rhizobia or Salt‐Sensitive Bradyrhizobia in Saline Soil.” Soil Biology and Biochemistry 27: 657–661.

[mbt270304-bib-0030] Fagorzi, C. , G. Bacci , R. Huang , et al. 2021. “Nonadditive Transcriptomic Signatures of Genotype‐By‐Genotype Interactions During the Initiation of Plant‐Rhizobium Symbiosis.” mSystems 6: e00974‐20. 33436514 10.1128/mSystems.00974-20 PMC7901481

[mbt270304-bib-0031] Fagorzi, C. , A. Ilie , F. Decorosi , et al. 2020. “Symbiotic and Non‐Symbiotic Members of the Genus *Ensifer* (Syn. *Sinorhizobium*) Are Separated Into Two Clades Based on Comparative Genomics and High‐Throughput Phenotyping.” Genome Biology and Evolution 12: 2521–2534. 33283865 10.1093/gbe/evaa221 PMC7719227

[mbt270304-bib-0122] Farhat, M. R. , L. Freschi , R. Calderon , et al. 2019. “GWAS for Quantitative Resistance Phenotypes in *Mycobacterium tuberculosis* Reveals Resistance Genes and Regulatory Regions.” Nature Communications 10, no. 1. 10.1038/s41467-019-10110-6. PMC6513847 31086182

[mbt270304-bib-0033] Fortune Business Insights . 2025. Alfalfa Pellets Market Size, Share & Industry Analysis, by Animal Type (Cattle, Horse, and Others), by Feed Type (Hay, Cubes, and Pellets) and Regional Forecast, 2025–2032. Fortune Business Insights.

[mbt270304-bib-0034] Galardini, M. , M. Brilli , G. Spini , et al. 2015. “Evolution of Intra‐Specific Regulatory Networks in a Multipartite Bacterial Genome.” PLoS Computational Biology 11: e1004478. 26340565 10.1371/journal.pcbi.1004478 PMC4560400

[mbt270304-bib-0035] Galardini, M. , A. Mengoni , M. Brilli , et al. 2011. “Exploring the Symbiotic Pangenome of the Nitrogen‐Fixing Bacterium *Sinorhizobium meliloti* .” BMC Genomics 12: 235. 21569405 10.1186/1471-2164-12-235 PMC3164228

[mbt270304-bib-0036] Galardini, M. , F. Pini , M. Bazzicalupo , E. G. Biondi , and A. Mengoni . 2013. “Replicon‐Dependent Bacterial Genome Evolution: The Case of *Sinorhizobium meliloti* .” Genome Biology and Evolution 5: 542–558. 23431003 10.1093/gbe/evt027 PMC3622305

[mbt270304-bib-0037] Geddes, B. A. , J. V. S. Kearsley , J. Huang , et al. 2021. “Minimal Gene Set From *Sinorhizobium* (*Ensifer*) *meliloti* pSymA Required for Efficient Symbiosis With Medicago.” Proceedings of the National Academy of Sciences of the United States of America 118: e2018015118. 33384333 10.1073/pnas.2018015118 PMC7814474

[mbt270304-bib-0038] Giaever, H. M. , O. B. Styrvold , I. Kaasen , and A. R. Strøm . 1988. “Biochemical and Genetic Characterization of Osmoregulatory Trehalose Synthesis in *Escherichia coli* .” Journal of Bacteriology 170: 2841–2849. 3131312 10.1128/jb.170.6.2841-2849.1988 PMC211211

[mbt270304-bib-0039] Gouffi, K. , N. Pica , V. Pichereau , and C. Blanco . 1999. “Disaccharides as a New Class of Nonaccumulated Osmoprotectants for *Sinorhizobium meliloti* .” Applied and Environmental Microbiology 65: 1491–1500. 10103242 10.1128/aem.65.4.1491-1500.1999 PMC91212

[mbt270304-bib-0040] Griffitts, J. S. , R. E. Carlyon , J. H. Erickson , et al. 2008. “A *Sinorhizobium meliloti* Osmosensory Two‐Component System Required for Cyclic Glucan Export and Symbiosis.” Molecular Microbiology 69: 479–490. 18630344 10.1111/j.1365-2958.2008.06304.x

[mbt270304-bib-0041] Haag, A. F. , M. F. F. Arnold , K. K. Myka , et al. 2012. “Molecular Insights Into Bacteroid Development During Rhizobium‐ Legume Symbiosis.” FEMS Microbiology Reviews 37: 364–383. 10.1111/1574-6976.12003 22998605

[mbt270304-bib-0042] Haque, M. M. , M. S. Biswas , M. K. Mosharaf , et al. 2022. “Halotolerant Biofilm‐Producing Rhizobacteria Mitigate Seawater‐Induced Salt Stress and Promote Growth of Tomato.” Scientific Reports 12: 5599. 35379908 10.1038/s41598-022-09519-9 PMC8980105

[mbt270304-bib-0124] Harrison, P. W. , R. P. J. Lower , N. K. D. Kim , and J. P. W. Young . 2010. “Introducing the Bacterial ‘Chromid’: Not a Chromosome, Not a Plasmid.” Trends in Microbiology 18, no. 4: 141–148. 10.1016/j.tim.2009.12.010. 20080407

[mbt270304-bib-0043] Hashem, F. M. , D. M. Swelim , L. D. Kuykendall , A. I. Mohamed , S. M. Abdel‐Wahab , and N. I. Hegazi . 1998. “Identification and Characterization of Salt‐ and Thermo‐Tolerant *Leucaena*‐Nodulating *Rhizobium* Strains.” Biology and Fertility of Soils 27: 335–341.

[mbt270304-bib-0044] Hawkins, J. P. , and I. J. Oresnik . 2022. “The Rhizobium‐Legume Symbiosis: Co‐Opting Successful Stress Management.” Frontiers in Plant Science 12: 796045. 35046982 10.3389/fpls.2021.796045 PMC8761673

[mbt270304-bib-0121] Holt, K. E. , H. Wertheim , R. N. Zadoks , et al. 2015. “Genomic Analysis of Diversity, Population Structure, Virulence, and Antimicrobial Resistance in Klebsiella pneumoniae, an Urgent Threat to Public Health.” Proceedings of the National Academy of Sciences 112, no. 27. 10.1073/pnas.1501049112. PMC4500264 26100894

[mbt270304-bib-0123] Hwang, W. , J. H. Yong , K. B. Min , et al. 2021. “Genome‐Wide Association Study of Signature Genetic Alterations Among *Pseudomonas aeruginosa* Cystic Fibrosis Isolates.” PLoS Pathogens 17, no. 6: e1009681. 10.1371/journal.ppat.1009681. 34161396 PMC8274868

[mbt270304-bib-0045] Ingram‐Smith, C. , and K. J. Miller . 1998. “Effects of Ionic and Osmotic Strength on the Glucosyltransferase of *Rhizobium meliloti* Responsible for Cyclic β‐(1,2)‐Glucan Biosynthesis.” Applied and Environmental Microbiology 64: 1290–1297. 16349538 10.1128/aem.64.4.1290-1297.1998 PMC106143

[mbt270304-bib-0046] Islam, M. M. , R. Bhattacharya , B. Sarkar , et al. 2022. “Different Soil Salinity Imparts Clear Alteration in Rhizospheric Bacterial Community Dynamics in Rice and Peanut.” Archives of Microbiology 204: 36. 10.1007/s00203-021-02695-8 34927211

[mbt270304-bib-0047] Johnson, D. A. , S. G. Tetu , K. Phillippy , J. Chen , Q. Ren , and I. T. Paulsen . 2008. “High‐Throughput Phenotypic Characterization of *Pseudomonas aeruginosa* Membrane Transport Genes.” PLoS Genetics 4: e1000211. 18833300 10.1371/journal.pgen.1000211 PMC2542419

[mbt270304-bib-0048] Jones, K. M. , H. Kobayashi , B. W. Davies , M. E. Taga , and G. C. Walker . 2007. “How Rhizobial Symbionts Invade Plants: The *Sinorhizobium—Medicago* Model.” Nature Reviews Microbiology 5: 619–633. 17632573 10.1038/nrmicro1705 PMC2766523

[mbt270304-bib-0049] Kazmierczak, T. , M. Nagymihály , F. Lamouche , et al. 2017. “Specific Host‐Responsive Associations Between *Medicago truncatula* Accessions and *Sinorhizobium* Strains.” Molecular Plant‐Microbe Interactions : MPMI 30: 399–409. 28437159 10.1094/MPMI-01-17-0009-R

[mbt270304-bib-0050] Kearsley, J. V. S. , B. A. Geddes , G. C. diCenzo , M. Zamani , and T. M. Finan . 2025. “A Minimized Symbiotic Gene Set From the 1.68 Mb pSymB Chromid of *Sinorhizobium meliloti* Reveals Auxiliary Symbiotic Loci.” BMC Biology 23: 204. 40629387 10.1186/s12915-025-02298-5 PMC12239276

[mbt270304-bib-0051] Koo, B. J. , D. C. Adriano , N. S. Bolan , and C. D. Barton . 2005. “Root Exudates and Microorganisms.” In Encyclopedia of Soils in the Environment, edited by D. Hillel , 421–428. Elsevier.

[mbt270304-bib-0052] Kreft, Ł. , A. Botzki , F. Coppens , K. Vandepoele , and M. Van Bel . 2017. “PhyD3: A Phylogenetic Tree Viewer With Extended phyloXML Support for Functional Genomics Data Visualization.” Bioinformatics 33: 2946–2947. 28525531 10.1093/bioinformatics/btx324

[mbt270304-bib-0053] Laganenka, L. , R. Colin , and V. Sourjik . 2016. “Chemotaxis Towards Autoinducer 2 Mediates Autoaggregation in *Escherichia coli* .” Nature Communications 7: 12984. 10.1038/ncomms12984 PMC5056481 27687245

[mbt270304-bib-0055] Lees, J. A. , M. Vehkala , N. Välimäki , et al. 2016. “Sequence Element Enrichment Analysis to Determine the Genetic Basis of Bacterial Phenotypes.” Nature Communications 7: 12797. 10.1038/ncomms12797 PMC5028413 27633831

[mbt270304-bib-0056] Lefort, V. , R. Desper , and O. Gascuel . 2015. “FastME 2.0: A Comprehensive, Accurate, and Fast Distance‐Based Phylogeny Inference Program.” Molecular Biology and Evolution 32: 2798–2800. 26130081 10.1093/molbev/msv150 PMC4576710

[mbt270304-bib-0057] Letunic, I. , and P. Bork . 2016. “Interactive Tree of Life (iTOL) v3: An Online Tool for the Display and Annotation of Phylogenetic and Other Trees.” Nucleic Acids Research 44: W242–W245. 27095192 10.1093/nar/gkw290 PMC4987883

[mbt270304-bib-0058] Li, X. , A. Wang , W. Wan , et al. 2021. “High Salinity Inhibits Soil Bacterial Community Mediating Nitrogen Cycling.” Applied and Environmental Microbiology 87: e0136621. 34406835 10.1128/AEM.01366-21 PMC8516042

[mbt270304-bib-0059] Liu, X. , Y. Luo , O. A. Mohamed , D. Liu , and G. Wei . 2014. “Global Transcriptome Analysis of *Mesorhizobium alhagi* CCNWXJ12‐2 Under Salt Stress.” BMC Microbiology 14: 1. 10.1186/s12866-014-0319-y PMC4302635 25539655

[mbt270304-bib-0060] Lloret, J. , L. Bolanos , M. M. Lucas , et al. 1995. “Ionic Stress and Osmotic Pressure Induce Different Alterations in the Lipopolysaccharide of a *Rhizobium meliloti* Strain.” Applied and Environmental Microbiology 61: 3701–3704. 16535151 10.1128/aem.61.10.3701-3704.1995 PMC1388713

[mbt270304-bib-0061] López‐Leal, G. , M. L. Tabche , S. Castillo‐Ramírez , A. Mendoza‐Vargas , M. A. Ramírez‐Romero , and G. Dávila . 2014. “RNA‐Seq Analysis of the Multipartite Genome of *Rhizobium etli* CE3 Shows Different Replicon Contributions Under Heat and Saline Shock.” BMC Genomics 15: 770. 25201548 10.1186/1471-2164-15-770 PMC4167512

[mbt270304-bib-0062] Maximiano, M. R. , E. Megías , I. R. Santos , et al. 2021. “Proteome Responses of *Rhizobium Tropici* CIAT 899 Upon Apigenin and Salt Stress Induction.” Applied Soil Ecology 159: 103815.

[mbt270304-bib-0065] Meier‐Kolthoff, J. P. , and M. Göker . 2019. “TYGS Is an Automated High‐Throughput Platform for State‐Of‐The‐Art Genome‐Based Taxonomy.” Nature Communications 10: 2182. 10.1038/s41467-019-10210-3 PMC6522516 31097708

[mbt270304-bib-0066] Mikheenko, A. , A. Prjibelski , V. Saveliev , D. Antipov , and A. Gurevich . 2018. “Versatile Genome Assembly Evaluation With QUAST‐LG.” Bioinformatics 34: i142–i150. 29949969 10.1093/bioinformatics/bty266 PMC6022658

[mbt270304-bib-0067] Miller, A. H. , F. Marks , L. Chan , H. Botella , D. Schnappinger , and S. Ehrt . 2024. “Interruption of Mycothiol Synthesis and Intracellular Redox Status Impact Iron‐Regulated Reporter Activation in *Mycobacterium smegmatis* .” Microbiology Spectrum 12: e0048724. 38860795 10.1128/spectrum.00487-24 PMC11218476

[mbt270304-bib-0068] Miller‐Williams, M. , P. C. Loewen , and I. J. Oresnik . 2006. “Isolation of Salt‐Sensitive Mutants of *Sinorhizobium meliloti* Strain Rm1021.” Microbiology 152: 2049–2059. 16804180 10.1099/mic.0.28937-0

[mbt270304-bib-0069] Mohammad, R. M. , M. Akhavan‐Kharazian , W. F. Campbell , and M. D. Rumbaugh . 1991. “Identification of Salt‐ and Drought‐Tolerant *Rhizobium meliloti* L. Strains.” Plant and Soil 134: 271–276.

[mbt270304-bib-0070] Muntyan, V. S. , and M. L. Roumiantseva . 2022. “Molecular Phylogenetic Analysis of Salt‐Tolerance‐Related Genes in Root‐Nodule Bacteria Species *Sinorhizobium meliloti* .” Agronomy 12: 1968.

[mbt270304-bib-0071] Nachshon, U. 2018. “Cropland Soil Salinization and Associated Hydrology: Trends, Processes and Examples.” Water 10: 1030.

[mbt270304-bib-0072] Nadeem, M. , J. Li , M. Yahya , et al. 2019. “Grain Legumes and Fear of Salt Stress: Focus on Mechanisms and Management Strategies.” International Journal of Molecular Sciences 20: 799. 30781763 10.3390/ijms20040799 PMC6412900

[mbt270304-bib-0073] Nogales, J. , R. Campos , H. BenAbdelkhalek , J. Olivares , C. Lluch , and J. Sanjuan . 2002. “ *Rhizobium tropici* Genes Involved in Free‐Living Salt Tolerance Are Required for the Establishment of Efficient Nitrogen‐Fixing Symbiosis With *Phaseolus vulgaris* .” Molecular Plant‐Microbe Interactions 15: 225–232. 11952125 10.1094/MPMI.2002.15.3.225

[mbt270304-bib-0074] Ondov, B. D. , T. J. Treangen , P. Melsted , et al. 2016. “Mash: Fast Genome and Metagenome Distance Estimation Using MinHash.” Genome Biology 17: 132. 27323842 10.1186/s13059-016-0997-x PMC4915045

[mbt270304-bib-0075] Page, A. J. , C. A. Cummins , M. Hunt , et al. 2015. “Roary: Rapid Large‐Scale Prokaryote Pan Genome Analysis.” Bioinformatics 31: 3691–3693. 26198102 10.1093/bioinformatics/btv421 PMC4817141

[mbt270304-bib-0076] Pagès, H. , P. Aboyoun , R. Gentleman , and S. DebRoy . 2017. “Biostrings: Efficient Manipulation of Biological Strings Version 2.58.0 From Bioconductor.”

[mbt270304-bib-0077] Perrin, E. , M. Fondi , I. Maida , et al. 2015. “Genomes Analysis and Bacteria Identification: The Use of Overlapping Genes as Molecular Markers.” Journal of Microbiological Methods 117: 108–112. 26235543 10.1016/j.mimet.2015.07.025

[mbt270304-bib-0078] Pini, F. , A. K. East , C. Appia‐Ayme , et al. 2017. “Bacterial Biosensors for In Vivo Spatiotemporal Mapping of Root Secretion.” Plant Physiology 174: 1289–1306. 28495892 10.1104/pp.16.01302 PMC5490882

[mbt270304-bib-0079] Power, R. A. , J. Parkhill , and T. De Oliveira . 2016. “Microbial Genome‐Wide Association Studies: Lessons From Human GWAS.” Nature Reviews Genetics 18: 41–50. 10.1038/nrg.2016.132 27840430

[mbt270304-bib-0080] Primo, E. , P. Bogino , S. Cossovich , E. Foresto , F. Nievas , and W. Giordano . 2020. “Exopolysaccharide II Is Relevant for the Survival of *Sinorhizobium meliloti* Under Water Deficiency and Salinity Stress.” Molecules 25: 4876. 33105680 10.3390/molecules25214876 PMC7659973

[mbt270304-bib-0081] Qadir, M. , E. Quillérou , V. Nangia , et al. 2014. “Economics of Salt‐Induced Land Degradation and Restoration.” Natural Resources Forum 38: 282–295.

[mbt270304-bib-0082] R Core Team . 2022. R: A Language and Environment for Statistical Computing. R Foundation for Statistical Computing.

[mbt270304-bib-0083] Ramachandran, V. K. , A. K. East , R. Karunakaran , J. A. Downie , and P. S. Poole . 2011. “Adaptation of *Rhizobium leguminosarum* to Pea, Alfalfa and Sugar Beet Rhizospheres Investigated by Comparative Transcriptomics.” Genome Biology 12: R106. 22018401 10.1186/gb-2011-12-10-r106 PMC3333776

[mbt270304-bib-0084] Rath, K. M. , N. Fierer , D. V. Murphy , and J. Rousk . 2019. “Linking Bacterial Community Composition to Soil Salinity Along Environmental Gradients.” ISME Journal 13: 836–846. 30446737 10.1038/s41396-018-0313-8 PMC6461869

[mbt270304-bib-0085] Reina‐Bueno, M. , M. Argandoña , J. J. Nieto , et al. 2012. “Role of Trehalose in Heat and Desiccation Tolerance in the Soil Bacterium *Rhizobium etli* .” BMC Microbiology 12: 207. 22985230 10.1186/1471-2180-12-207 PMC3518184

[mbt270304-bib-0086] Rengasamy, P. 2010. “Soil Processes Affecting Crop Production in Salt‐Affected Soils.” Functional Plant Biology 37: 613–620.

[mbt270304-bib-0087] Riley, A. B. , M. A. Grillo , B. Epstein , P. Tiffin , and K. D. Heath . 2023. “Discordant Population Structure Among Rhizobium Divided Genomes and Their Legume Hosts.” Molecular Ecology 32: 2646–2659. 36161739 10.1111/mec.16704

[mbt270304-bib-0088] Roumiantseva, M. L. , and V. S. Muntyan . 2015. “Root Nodule Bacteria *Sinorhizobium meliloti*: Tolerance to Salinity and Bacterial Genetic Determinants.” Microbiology (Reading) 84: 303–318. 26263687

[mbt270304-bib-0089] Seemann, T. 2014. “Prokka: Rapid Prokaryotic Genome Annotation.” Bioinformatics 30: 2068–2069. 24642063 10.1093/bioinformatics/btu153

[mbt270304-bib-0090] Shamseldin, A. , J. Nyalwidhe , and D. Werner . 2006. “A Proteomic Approach Towards the Analysis of Salt Tolerance in *Rhizobium etli* and *Sinorhizobium meliloti* Strains.” Current Microbiology 52: 333–339. 16604415 10.1007/s00284-005-6472-7

[mbt270304-bib-0091] Singh, A. 2021. “Soil Salinization Management for Sustainable Development: A Review.” Journal of Environmental Management 277: 111383. 33035935 10.1016/j.jenvman.2020.111383

[mbt270304-bib-0092] Stamatakis, A. 2014. “RAxML Version 8: A Tool for Phylogenetic Analysis and Post‐Analysis of Large Phylogenies.” Bioinformatics 30: 1312–1313. 24451623 10.1093/bioinformatics/btu033 PMC3998144

[mbt270304-bib-0093] Sugawara, M. , B. Epstein , B. D. Badgley , et al. 2013. “Comparative Genomics of the Core and Accessory Genomes of 48 *Sinorhizobium* Strains Comprising Five Genospecies.” Genome Biology 14: R17. 23425606 10.1186/gb-2013-14-2-r17 PMC4053727

[mbt270304-bib-0094] Szymańska, S. , L. Borruso , L. Brusetti , P. Hulisz , B. Furtado , and K. Hrynkiewicz . 2018. “Bacterial Microbiome of Root‐Associated Endophytes of *Salicornia europaea* in Correspondence to Different Levels of Salinity.” Environmental Science and Pollution Research 25: 25420–25431. 29951760 10.1007/s11356-018-2530-0 PMC6133108

[mbt270304-bib-0095] Talebi, M. B. , M. Bahar , G. Saeidi , A. Mengoni , and M. Bazzicalupo . 2008. “Diversity of *Sinorhizobium* Strains Nodulating *Medicago sativa* From Different Iranian Regions.” FEMS Microbiology Letters 288: 40–46. 18783438 10.1111/j.1574-6968.2008.01329.x

[mbt270304-bib-0096] Tanthanuch, W. , P. Tittabutr , S. Mohammed , et al. 2010. “Identification of Salt‐Tolerant *Sinorhizobium* sp. Strain BL3 Membrane Proteins Based on Proteomics.” Microbes and Environments 25: 275–280. 21576882 10.1264/jsme2.me09185

[mbt270304-bib-0097] Thrall, P. H. , L. M. Broadhurst , M. S. Hoque , and D. J. Bagnall . 2009. “Diversity and Salt Tolerance of Native Acacia Rhizobia Isolated From Saline and Non‐Saline Soils.” Austral Ecology 34: 950–963.

[mbt270304-bib-0098] Tomaz, A. , P. Palma , S. Fialho , et al. 2020. “Risk Assessment of Irrigation‐Related Soil Salinization and Sodification in Mediterranean Areas.” Water (Basel) 12: 3569.

[mbt270304-bib-0099] Toro, N. , P. J. Villadas , M. D. Molina‐Sánchez , et al. 2017. “The Underlying Process of Early Ecological and Genetic Differentiation in a Facultative Mutualistic *Sinorhizobium meliloti* Population.” Scientific Reports 7: 675. 28386109 10.1038/s41598-017-00730-7 PMC5429615

[mbt270304-bib-0100] Uffelmann, E. , Q. Q. Huang , N. S. Munung , et al. 2021. “Genome‐Wide Association Studies.” Nature Reviews Methods Primers 1: 59.

[mbt270304-bib-0101] Unni, S. , and K. K. Rao . 2001. “Protein and Lipopolysaccharide Profiles of a Salt‐Sensitive *Rhizobium* sp. and Its Exopolysaccharide‐Deficient Mutant.” Soil Biology & Biochemistry 33: 111–115.

[mbt270304-bib-0102] Viti, C. , A. Bellabarba , M. Daghio , et al. 2021. “Alfalfa for a Sustainable Ovine Farming System: Proposed Research for a New Feeding Strategy Based on Alfalfa and Ecological Leftovers in Drought Conditions.” Sustainability 13: 3880.

[mbt270304-bib-0103] Vriezen, J. A. C. , F. J. de Bruijn , and K. Nüsslein . 2006. “Desiccation Responses and Survival of *Sinorhizobium meliloti* USDA 1021 in Relation to Growth Phase, Temperature, Chloride and Sulfate Availability.” Letters in Applied Microbiology 42: 172–178. 16441384 10.1111/j.1472-765X.2005.01808.x

[mbt270304-bib-0104] Vriezen, J. A. C. , F. J. De Bruijn , and K. Nüsslein . 2007. “Responses of Rhizobia to Desiccation in Relation to Osmotic Stress, Oxygen, and Temperature.” Applied and Environmental Microbiology 73: 3451–3459. 17400779 10.1128/AEM.02991-06 PMC1932662

[mbt270304-bib-0105] Vriezen, J. A. C. , F. J. De Bruijn , and K. Nüsslein . 2013. “Identification and Characterization of a NaCL‐Responsive Genetic Locus Involved in Survival During Desiccation in *Sinorhizobium meliloti* .” Applied and Environmental Microbiology 79: 5693–5700. 23851090 10.1128/AEM.01037-13 PMC3754180

[mbt270304-bib-0125] Wagner, M. , J. Döhlemann , D. Geisel , et al. 2024. “Engineering a Sinorhizobium meliloti Chassis with Monopartite, Single Replicon Genome Configuration.” ACS Synthetic Biology 13, no. 8: 2515–2532. 10.1021/acssynbio.4c00281. 39109796

[mbt270304-bib-0106] Wang, Y. , W. Ren , Y. Li , et al. 2019. “Nontargeted Metabolomic Analysis to Unravel the Impact of di (2‐Ethylhexyl) Phthalate Stress on Root Exudates of Alfalfa ( *Medicago sativa* ).” Science of the Total Environment 646: 212–219. 30053665 10.1016/j.scitotenv.2018.07.247

[mbt270304-bib-0107] Wei, W. , J. Jiang , X. Li , L. Wang , and S. S. Yang . 2004. “Isolation of Salt‐Sensitive Mutants From *Sinorhizobium meliloti* and Characterization of Genes Involved in Salt Tolerance.” Letters in Applied Microbiology 39: 278–283. 15287875 10.1111/j.1472-765X.2004.01577.x

[mbt270304-bib-0108] Wick, R. R. , L. M. Judd , C. L. Gorrie , and K. E. Holt . 2017. “Unicycler: Resolving Bacterial Genome Assemblies From Short and Long Sequencing Reads.” PLoS Computational Biology 13: e1005595. 28594827 10.1371/journal.pcbi.1005595 PMC5481147

[mbt270304-bib-0109] Yang, Q. , X. Wang , M. Han , et al. 2025. “Bacterial Genome‐Wide Association Studies: Exploring the Genetic Variation Underlying Bacterial Phenotypes.” Applied and Environmental Microbiology 91: e02512‐24. 40377303 10.1128/aem.02512-24 PMC12175497

[mbt270304-bib-0110] Yuan, Y. , C. Brunel , M. van Kleunen , J. Li , and Z. Jin . 2019. “Salinity‐Induced Changes in the Rhizosphere Microbiome Improve Salt Tolerance of *Hibiscus hamabo* .” Plant and Soil 443: 525–537.

[mbt270304-bib-0111] Yukun, G. , C. Jianghui , R. Genzeng , et al. 2021. “Changes in the Root‐Associated Bacteria of Sorghum Are Driven by the Combined Effects of Salt and Sorghum Development.” Environmental Microbiomes 16: 14. 10.1186/s40793-021-00383-0 PMC8356455 34380546

[mbt270304-bib-0112] Zahran, H. H. 1999. “Rhizobium ‐Legume Symbiosis and Nitrogen Fixation Under Severe Conditions and in an Arid Climate.” Microbiology and Molecular Biology Reviews 63: 968–989. 10585971 10.1128/mmbr.63.4.968-989.1999 PMC98982

[mbt270304-bib-0113] Zhang, K. , Y. Shi , X. Cui , et al. 2019. “Salinity Is a Key Determinant for Soil Microbial Communities in a Desert Ecosystem.” mSystems 4: e00225‐18. 30801023 10.1128/mSystems.00225-18 PMC6372838

[mbt270304-bib-0114] Zhang, Y. , Y. Gu , R. Wu , et al. 2022. “Exploring the Relationship Between the Signal Molecule AI‐2 and the Biofilm Formation of *Lactobacillus sanfranciscensis* .” LWT ‐ Food Science and Technology 154: 112704.

[mbt270304-bib-0116] Zhu, Z. , Y. Zhang , J. Li , and H. Dong . 2021. “Insight Into Quorum Sensing and Microbial Community of an Anammox Consortium in Response to Salt Stress: From ‘*Candaditus* Brocadia’ to ‘*Candaditus* Scalindua’.” Science of the Total Environment 796: 148979. 34274671 10.1016/j.scitotenv.2021.148979

[mbt270304-bib-0117] Zou, N. , P. J. Dart , and N. E. Marcar . 1995. “Interaction of Salinity and Rhizobial Strain on Growth and N2‐Fixation by *Acacia ampliceps* .” Soil Biology & Biochemistry 27: 409–413.

