# Supplementary material for: Genomic and Phenotypic Bases of Salt Tolerance in Sinorhizobium meliloti : Candidate Traits for Bioinoculant Development Addressing Saline Soils
Source: Microb Biotechnol. 2026 Jan 29;19(1):e70304. doi: 10.1111/1751-7915.70304 (PMC12855168; doi:10.1111/1751-7915.70304)
Supplement: Supplementary file 2 — Appendix S1: mbt270304‐sup‐0002‐supinfo.docx. [file MBT2-19-e70304-s010.docx]

**SUPPLEMENTARY FIGURES**

**Figure S1. “Phylogram**”: Tree inferred with FastME from GBDP distances calculated from genome sequences. The branch lengths were scaled in terms of GBDP distance formula *d_5_*. The numbers above branches are GBDP pseudo-bootstrap support values > 60 % from 100 replications, with an average branch support of 28.2 %. Algerian strains were highlighted in bold.


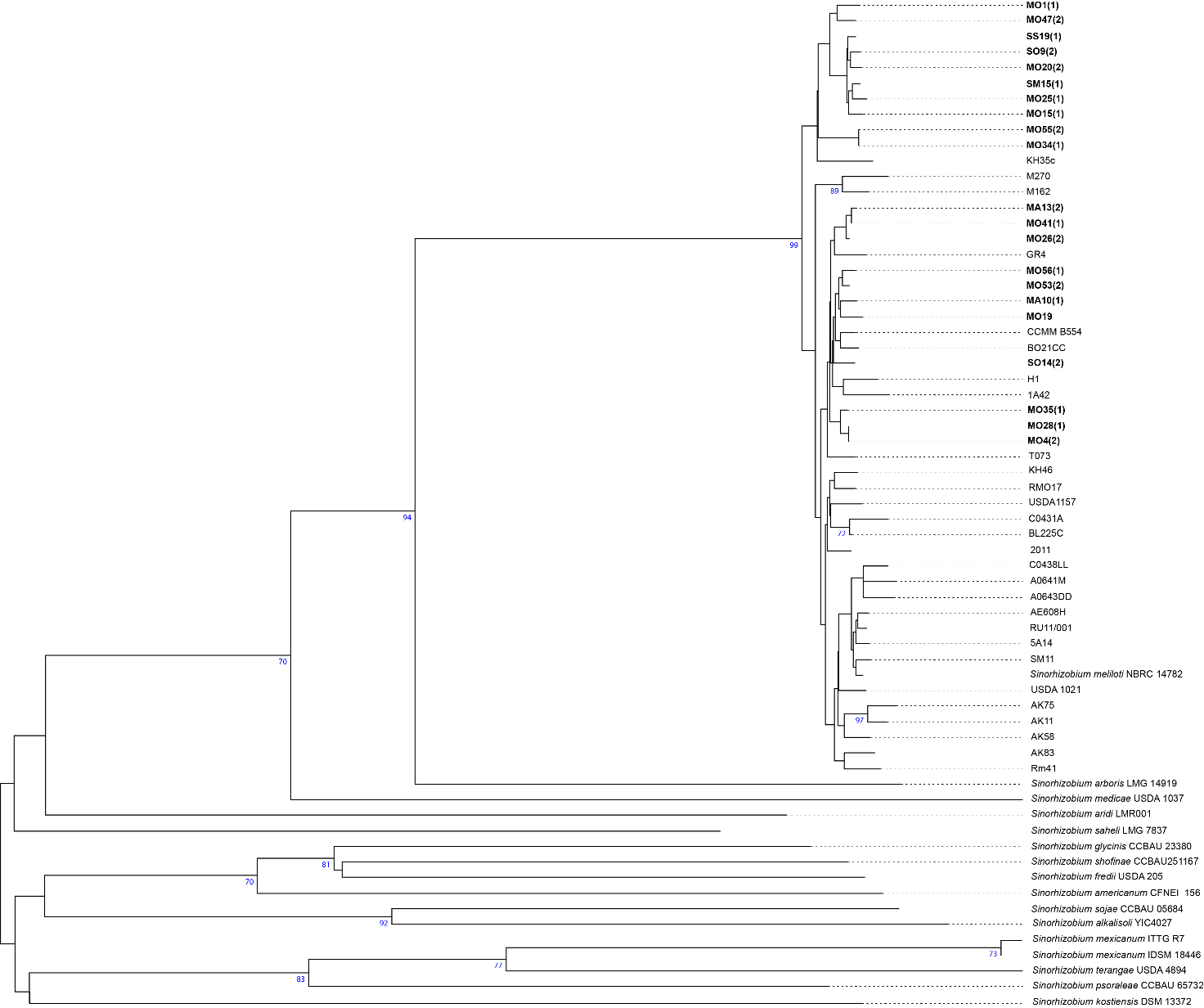


**Figure S2. *Sinorhizobium meliloti* strains with high salt resistance at 400 mM and 600 mM NaCl.** A) Venn diagram between the groups characterized by a higher AOU at 400 mM (groups A and B) and 600 mM NaCl (Groups A, B and C). B) Venn diagram with the top AOU groups at 400 mM (groups A) and 600 mM NaCl (Groups A and B). Algerian strains were highlighted in bold.


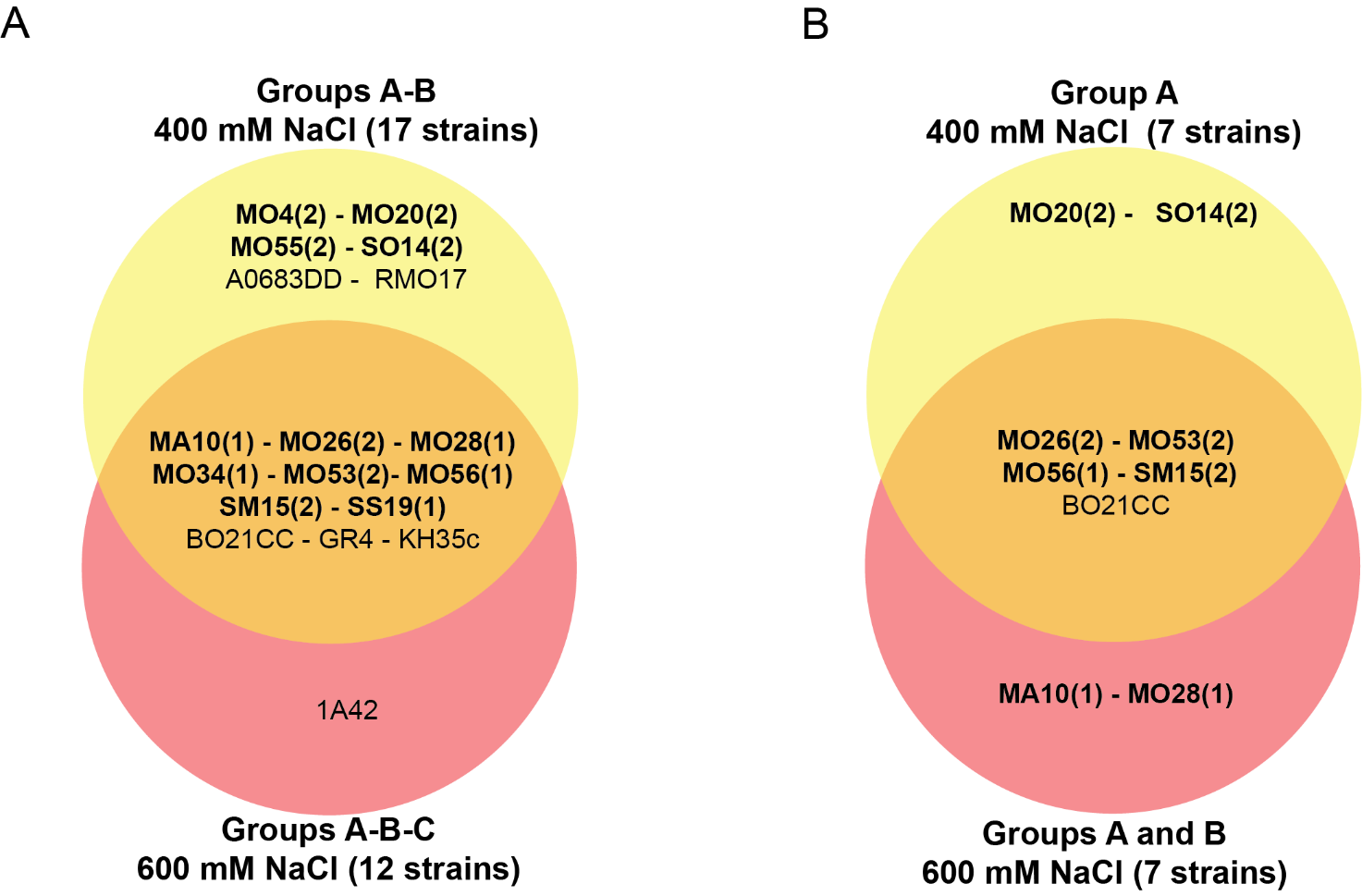


**Figure S3. Functions associated with the salt-resistant phenotype.** Frequency of candidate functions of gene hits (A) and regulatory regions (B) identified by *best* *k*-mers in the most salt-resistant strains. The frequency of candidate functions reported as COG annotations (rows) in each strain (columns) is represented by grayscale shades. In the upset plots, (C) the number of shared functions of gene hits, and (D) shared functions of regulatory regions, in each and different combinations of strains are reported.


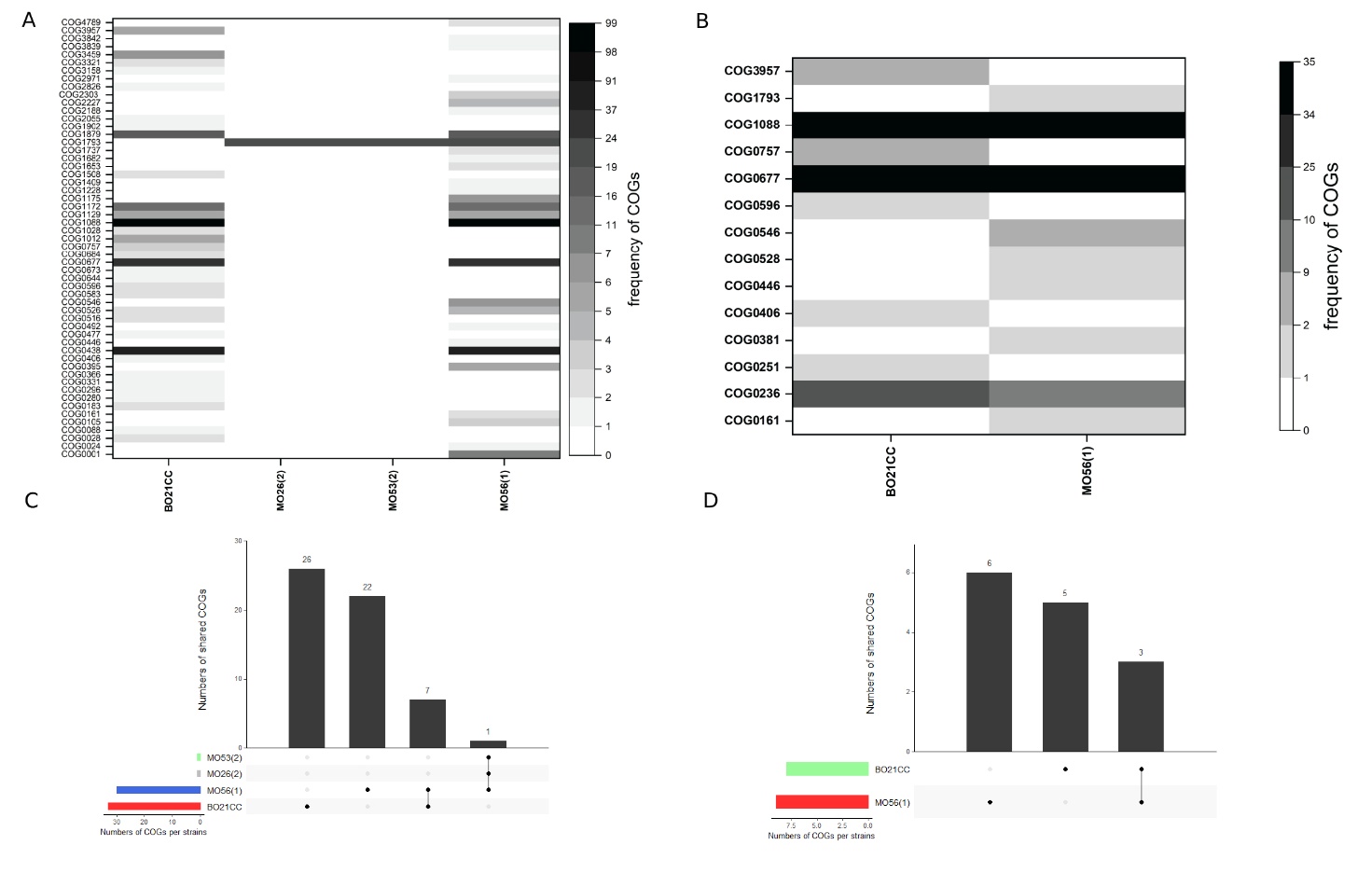


**Figure S4. Distribution of scaffolds hit by *best* *k*-mers among different replicons in *Sinorhizobium meliloti* MO56(1) and BO21CC through the alignment with the genome of *S. meliloti* 2011.** Distribution of *k*-mers-cointaining scaffolds of MO56(1) (A&C) and BO21CC (B & D) mapped on the genome of 2011 (RefSeq assembly GCF_000346065.1) referring to both gene hits (A-B) and regulatory region hits (C-D). Data are reported as percentage on the total number of considered scaffolds in each dataset. Scaffold reported as Unknown could not be aligned on the reference genome.


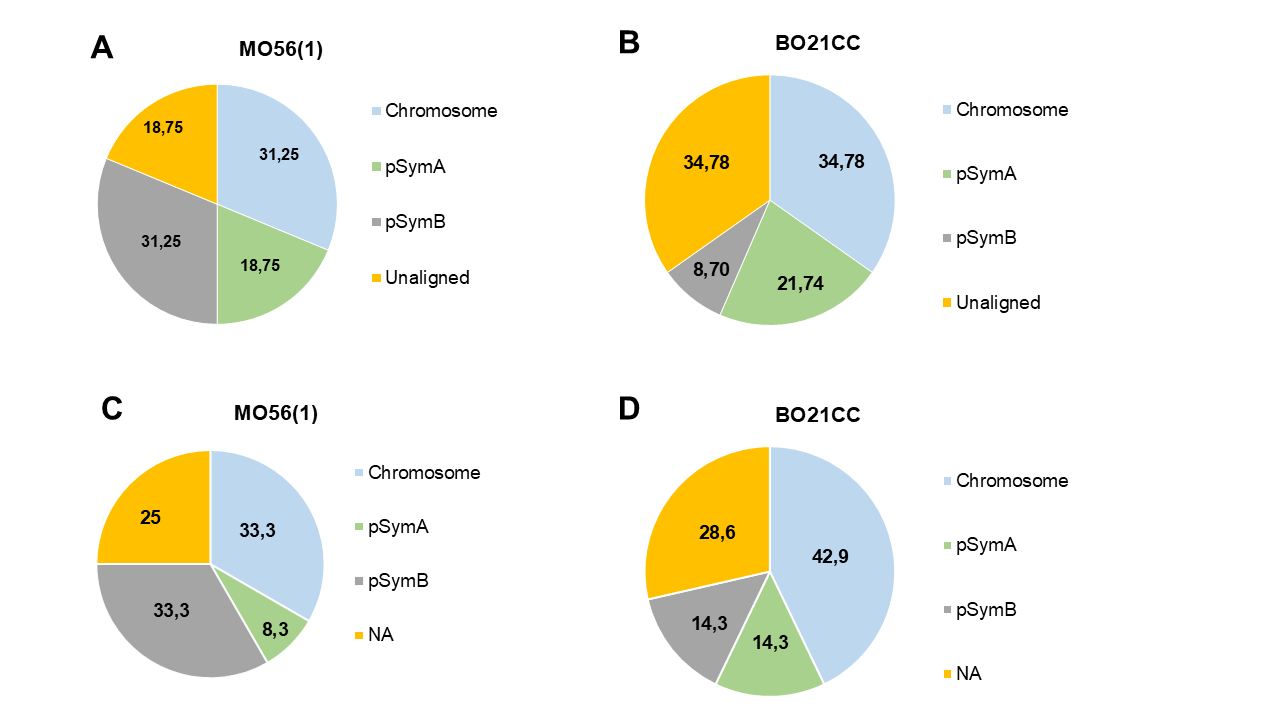


**Figure S5. Metabolic activities on carboxylic acid, amino acid and other sources of *S. meliloti* strains BO21CC, MO56(1) and RU11/001 at 0 mM and 300 mM NaCl.** Carboxylic acid (A), amino acid (C) and others (B) were used as carbon sources. Other sources refer to alcohols, ester & fatty acids, polymers, amine & amide. The metabolic activities were expressed and reported as area of the kinetic curves for each condition. Hierarchical clustering with complete linkage of *S. meliloti* strains was reported**.**

**
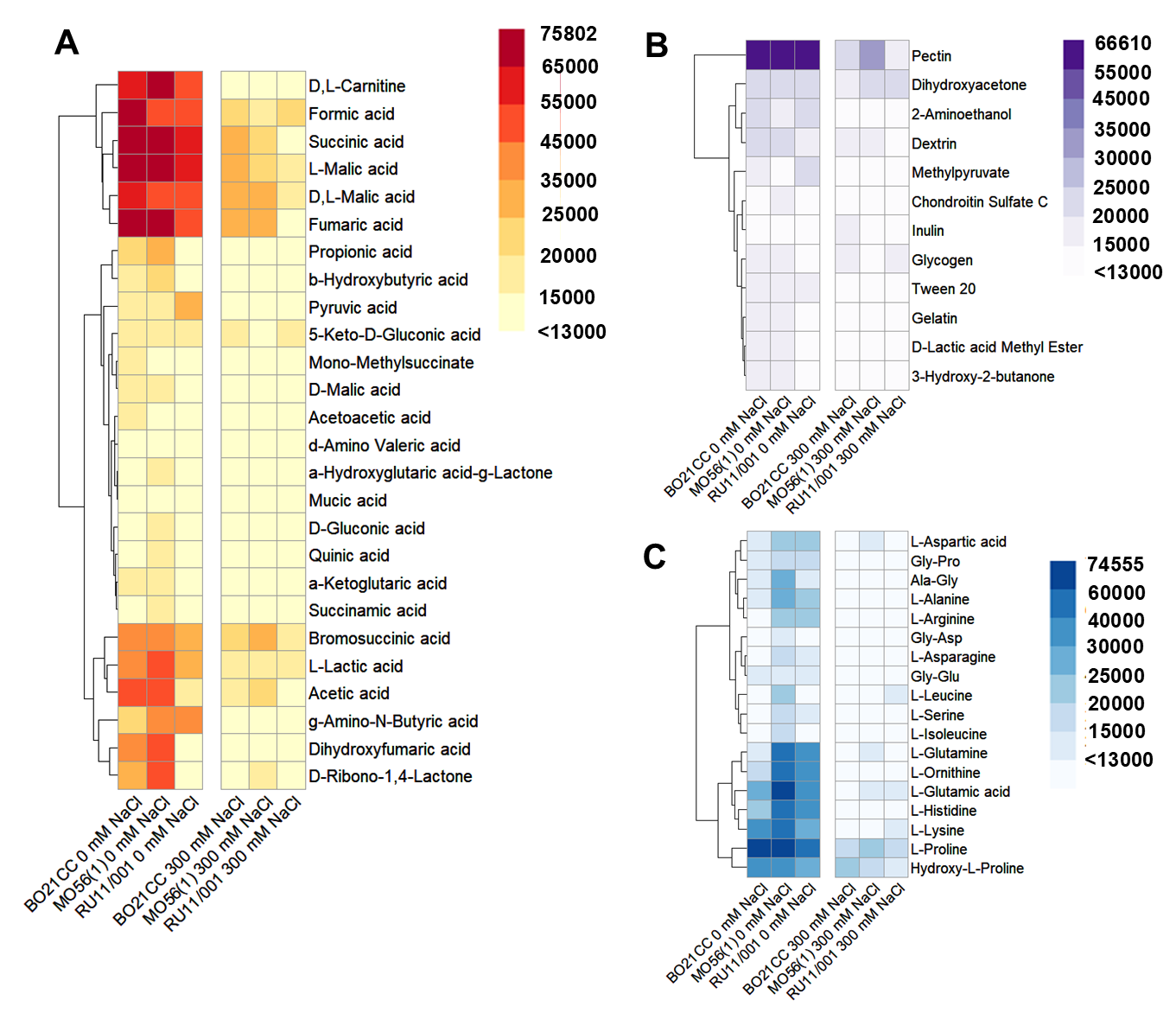
**

**SUPPLEMENTARY TABLES**

**Table S1. List of the Algerian *Sinorhizobium meliloti* strains used in this work.**

| **Strain name** | **Geographical site** | **BioProject ID** | **BioSample accession** | **Soil salinity level** | **Source of isolation** |
| --- | --- | --- | --- | --- | --- |
| MA10(1) | Fleuris (W. Oran) | PRJNA853716 | SAMN29401383 | Moderately saline | *M. sativa* cv. unspecified |
| MA13(2) | Fleuris (W. Oran) | PRJNA853716 | SAMN29401382 | Moderately saline | *M. sativa* cv. unspecified |
| MO1(1) | Fleuris (W. Oran) | PRJNA853716 | SAMN29401381 | Moderately saline | *M. sativa* cv. unspecified |
| MO15(1) | Fleuris (W. Oran) | PRJNA853716 | SAMN29401379 | Moderately saline | *M. sativa* cv. unspecified |
| MO19 | Fleuris (W. Oran) | PRJNA853716 | SAMN29401378 | Moderately saline | *M. sativa* cv. unspecified |
| MO20(2) | Fleuris (W. Oran) | PRJNA853716 | SAMN29401377 | Moderately saline | *M. sativa* cv. unspecified |
| MO25(1) | Fleuris (W. Oran) | PRJNA853716 | SAMN29401376 | Moderately saline | *M. sativa* cv. unspecified |
| MO26(2) | Fleuris (W. Oran) | PRJNA853716 | SAMN29401375 | Moderately saline | *M. sativa* cv. unspecified |
| MO28(1) | Fleuris (W. Oran) | PRJNA853716 | SAMN29401374 | Moderately saline | *M. sativa* cv. unspecified |
| MO34(1) | Fleuris (W. Oran) | PRJNA853716 | SAMN29401373 | Moderately saline | *M. sativa* cv. unspecified |
| MO35(1) | Fleuris (W. Oran) | PRJNA853716 | SAMN29401372 | Moderately saline | *M. sativa* cv. unspecified |
| MO4(2) | Fleuris (W. Oran) | PRJNA853716 | SAMN29401380 | Moderately saline | *M. sativa* cv. unspecified |
| MO41(1) | Fleuris (W. Oran) | PRJNA853716 | SAMN29401371 | Moderately saline | *M. sativa* cv. unspecified |
| MO47(2) | Fleuris (W. Oran) | PRJNA853716 | SAMN29401370 | Moderately saline | *M. sativa* cv. unspecified |
| MO53(2) | Fleuris (W. Oran) | PRJNA853716 | SAMN29401369 | Moderately saline | *M. sativa* cv. unspecified |
| MO55(2) | Fleuris (W. Oran) | PRJNA853716 | SAMN29401368 | Moderately saline | *M. sativa* cv. unspecified |
| MO56(1) | Fleuris (W. Oran) | PRJNA853716 | SAMN29401367 | Moderately saline | *M. sativa* cv. unspecified |
| SM15(1) | El Malah (W. d'Ain Témouchent) | PRJNA853716 | SAMN29401366 | Moderately saline | *M. sativa* cv. Seriver |
| SO14(2) | Oued Sebbah (W. d'Ain Témouchent)) | PRJNA853716 | SAMN29401364 | Saline | *M. sativa* cv. Seriver |
| SO9(2) | Oued Sebbah (W. d'Ain Témouchent) | PRJNA853716 | SAMN29401365 | Saline | *M. sativa* cv. Seriver |
| SS19(1) | Es Senia (Oran) | PRJNA853716 | SAMN29401363 | Not saline | *M. sativa* cv. unspecified |

**Table S2. Genomes assembly summary statistics of Algerian *Sinorhizobium meliloti* isolates**

| **Strains** | **Biosample accession** | **Genome accession** | **Genome coverage** | **Total length (>= 0 bp)** | **Contigs (>= 0 bp)** | **Contigs N50** | **Contigs L50** | **GC (%)** | **CDS** | **rRNA** | **tRNA** | **tmRNA** |
| --- | --- | --- | --- | --- | --- | --- | --- | --- | --- | --- | --- | --- |
| MA10(1) | SAMN29401383 | JAMZCS000000000 | 167x | 6463707 | 153 | 153932 | 12 | 62.19 | 6000 | 5S, 16S, 23S | 56 | 1 |
| MA13(2) | SAMN29401382 | JAMZCR000000000 | 210x | 6714708 | 91 | 215711 | 10 | 62.22 | 6270 | 5S, 16S, 23S | 57 | 1 |
| MO1(1) | SAMN29401381 | JAMZCQ000000000 | 154x | 6472345 | 63 | 330603 | 7 | 62.3 | 6017 | 5S, 16S, 23S | 56 | 1 |
| MO4(2) | SAMN29401380 | JAMZCP000000000 | 180x | 6795388 | 90 | 319638 | 8 | 62.14 | 6351 | 5S, 16S, 23S | 57 | 1 |
| MO15(1) | SAMN29401379 | JAMZCO000000000 | 166x | 6650060 | 141 | 192101 | 10 | 62.13 | 6176 | 5S, 16S, 23S | 55 | 1 |
| MO19 | SAMN29401378 | JAMZCN000000000 | 293x | 6818496 | 140 | 177694 | 11 | 62.12 | 6396 | 5S, 16S, 23S | 55 | 1 |
| MO20(2) | SAMN29401377 | JAMZCM000000000 | 172x | 6556148 | 97 | 323028 | 8 | 62.23 | 6084 | 5S, 16S, 23S | 56 | 1 |
| MO25(1) | SAMN29401376 | JAMZCL000000000 | 223x | 6768153 | 151 | 254130 | 10 | 62.11 | 6305 | 5S, 16S, 23S | 59 | 1 |
| MO26(2) | SAMN29401375 | JAMZCK000000000 | 242x | 6584239 | 116 | 163856 | 15 | 62.2 | 6126 | 5S, 16S, 23S | 55 | 1 |
| MO28(1) | SAMN29401374 | JAMZCJ000000000 | 163x | 6795718 | 96 | 215950 | 10 | 62.14 | 6348 | 5S, 16S, 23S | 58 | 1 |
| MO34(1) | SAMN29401373 | JAMZCI000000000 | 221x | 6527643 | 99 | 264086 | 10 | 62.21 | 6106 | 5S, 16S, 23S | 55 | 1 |
| MO35(1) | SAMN29401372 | JAMZCH000000000 | 348x | 6565157 | 104 | 294365 | 9 | 62.23 | 6155 | 5S, 16S, 23S | 58 | 1 |
| MO41(1) | SAMN29401371 | JAMZCG000000000 | 198x | 6596457 | 100 | 183338 | 12 | 62.25 | 6141 | 5S, 16S, 23S | 56 | 1 |
| MO47(2) | SAMN29401370 | JAMZCF000000000 | 197x | 6707328 | 98 | 393212 | 6 | 62.18 | 6293 | 5S, 16S, 23S | 55 | 1 |
| MO53(2) | SAMN29401369 | JAMZCE000000000 | 210x | 6454551 | 110 | 175359 | 12 | 62.31 | 5978 | 5S, 16S, 23S | 57 | 1 |
| MO55(2) | SAMN29401368 | JAMZCD000000000 | 197x | 6519993 | 124 | 263830 | 9 | 62.21 | 6097 | 5S, 16S, 23S | 52 | 1 |
| MO56(1) | SAMN29401367 | JAMZCC000000000 | 187x | 6627008 | 148 | 153862 | 12 | 62.13 | 6187 | 5S, 16S, 23S | 55 | 1 |
| SM15(1) | SAMN29401366 | JAMZCB000000000 | 253x | 6653622 | 98 | 231080 | 9 | 62.16 | 6200 | 5S, 16S, 23S | 56 | 1 |
| SO9(2) | SAMN29401365 | JAMZCA000000000 | 203x | 6724991 | 83 | 279763 | 9 | 62.19 | 6290 | 5S, 16S, 23S | 54 | 1 |
| SO14(2) | SAMN29401364 | JAMZBZ000000000 | 207x | 6620114 | 121 | 215734 | 11 | 62.24 | 6153 | 5S, 16S, 23S | 57 | 1 |
| SS19(1) | SAMN29401363 | JAMZBY000000000 | 197x | 6650993 | 126 | 263672 | 8 | 62.19 | 6199 | 5S, 16S, 23S | 57 | 1 |

**Table S3**. List of analyzed and sequenced *S. meliloti* strains.

| **Strain name** | **Origin** | **Source of isolation** | **BioProject ID** | **Assembly** | **Reference** |
| --- | --- | --- | --- | --- | --- |
| 1A42 | Iran | *M. sativa*, nodules | PRJNA167584 | GCA_000287435.1 | Talebi-Bedaf et al., 2008 |
| 2011 | Australia | SU47 derivative | PRJNA187276 | GCA_000346065.1 | Meade and Signer 1977 |
| 5A14 | Iran | *M. sativa*, nodules | PRJNA167593 | GCA_000287415.1 | Talebi-Bedaf et al., 2008 |
| A0641M | Italy | *M. sativa* cv. Oneida, nodules | PRJNA167594 | GCA_000287455.1 | Carelli et al., 2000 |
| A0643DD | Italy | *M. sativa* cv. Oneida, nodules | PRJNA167595 | GCA_000287575.1 | Carelli et al., 2000 |
| AE608H | Italy | *M. sativa* cv. Estival, nodules | PRJNA167596 | GCA_000287555.1 | Carelli et al., 2000 |
| AK11 | Kazakhstan | *M. falcata,* nodules | PRJNA167597 | GCA_000287535.1 | Galardini et al., 2013 |
| AK58 | Kazakhstan | *M. falcata,* nodules | PRJNA71315 | GCA_000473425.1 | Giuntini et al., 2005 |
| AK75 | Kazakhstan | *M. lupulina*, nodules | PRJNA167598 | AKZU00000000 | Galardini et al,., 2013b |
| AK83 | Kazakhstan | *M. falcata,* nodules | PRJNA41993 | GCA_000147795.3 | Giuntini et al., 2005 |
| BL225C | Italy | *M. sativa* cv. Lodi, nodules | PRJNA42477 | GCA_000147775.3 | Giuntini et al., 2005 |
| BO21CC | Italy | *M. sativa* cv. Oneida, nodules | PRJNA74719 | GCA_000473405.1 | Giuntini et al., 2005 |
| C0431A | Italy | *M. sativa* cv. Oneida, nodules | PRJNA167599 | GCA_000287375.1 | Carelli et al., 2000 |
| C0438LL | Italy | *M. sativa* cv. Oneida, nodules | PRJNA167600 | GCA_000287495.1 | Carelli et al., 2000 |
| CCMM-B554 | Morocco | *M. arborea*, nodules | PRJNA369312 | GCA_002215195.1 | Kazmierczak et al., 2017 |
| GR4 | Spain | Agricultural field | PRJNA175860 | GCA_000320385.2 | Martinez-Abarca et al., 2013 |
| H1 | Italy | *M. sativa*, leaves | PRJNA167601 | GCA_000287475.1 | Galardini et al., 2013b |
| KH35c | France | *M. truncatula,* nodules | PRJNA388336 | GCA_002197105.1 | Sugawara et al., 2013 |
| KH46 |  |  | PRJNA388336 | GCA_002197465.1 | Nelson et al., 2018 |
| M162 | Syria | *Medicago truncatula,* nodules | PRJNA388336 | GCA_002197125.1 | Sugawara et al., 2013 |
| M270 | Jordan | *M. truncatula,* nodules | PRJNA388336 | GCA_002197085.1 | Sugawara et al., 2013 |
| Rm41 | Hungary | *Melilotus/Medicago,* nodules | PRJEB436 | GCA_000304415.1 | Szende and Ordogh, 1960 |
| RMO17 | Spain | *M. orbicularis,* nodules | PRJNA244712 | GCA_000747295.1 | Villadas et al.,1995 |
| RU11/001 | Germany | *M. sativa,* nodules | PRJEB4559 | GCA_001050915.2 | Krupski et al., 1985 |
| SM11 | Germany | *M. sativa,* nodules | PRJNA41117 | GCA_000218265.1 | Stiens et al., 2007 |
| T073 | Tunisia | *M. truncatula,* nodules | PRJNA388336 | GCA_002197145.1 | Sugawara et al., 2013 |
| USDA1021 | USA | *M. sativa,* nodules | PRJNA388336 | GCA_002197445.1 | Sugawara et al., 2013 |
| USDA1157 | USA, California | *M. truncatula,* nodules | PRJNA388336 | GCA_002197025.1 | Nelson et al., 2018 |

**Table S4**. **One-way ANOVA outputs on normalized metabolic activities of strains at different NaCl concentrations (200 mM, 400 mM, 600 mM, 700 mM, 800 mM, 900 mM and 1M).** The degrees of freedom (Df), the sum of squares (Sum Sq), the mean of the sum of squares (Mean Sq), the F value and the *P* value of the F statistic (Pr(>F)) are reported for each dataset.

| **NaCl concentration** | | **Df** | **Sum Sq** | | **Mean Sq** | | | **F value** | **Pr(>F)** | |
| --- | --- | --- | --- | --- | --- | --- | --- | --- | --- | --- |
| **200 mM** | 48  98 | | | 0.22413  0.03325 | | 0.004669  0.000339 | 13.76 | | | <2e-16 *** |
| **400 mM** | 48  98 | | | 0.9122  0.0627 | | 0.01901  0.00064 | 29.71 | | | <2e-16 *** |
| **600 mM** | 48  98 | | | 2.6708  0.0391 | | 0.05564  0.00040 | 139.6 | | | <2e-16 *** |
| **700 mM** | 48  98 | | | 0.5607  0.02 | | 0.011682  0.000204 | 57.31 | | | <2e-16 *** |
| **800 mM** | 48  98 | | | 0.05467  0.00338 | | 0.0011389  0.0000345 | 32.99 | | | <2e-16 *** |
| **900 mM** | 48  98 | | | 0.023055  0.004917 | | 0.0004803  0.0000502 | 9.572 | | | <2e-16 *** |
| **1 M** | 48  98 | | | 0.01558  0.003767 | | 0.0003246  0.0000384 | 8.444 | | | <2e-16 *** |

Signif. codes: 0 ‘***’ 0.001 ‘**’ 0.01 ‘*’ 0.05 ‘.’ 0.1 ‘’ 1

**Table S5 Identification of significantly *k*-mers by association analysis with PhenotypeSeeker.** The number of total k-mers associated with the salt-resistant phenotype (P value < 0.05) determined at three NaCl concentration (400 mM, 600 mM and 700 mM) and their range of P value are reported.

| Dataset | Total  *k*-mers  (*P* value < 0.05) | *P* value range |
| --- | --- | --- |
| 400 mM | 23753 | 4.4e^-09^ - 1.64e^-19^ |
| 600 mM | 31439 | 4.29e^-09^ - 1.07e^-14^ |

**Table S6. List of COG classes.**

| **COG ID** | **COG name** |
| --- | --- |
| J | Translation, ribosomal structure and biogenesis |
| A | RNA processing and modification |
| K | Transcription |
| L | Replication, recombination and repair |
| B | Chromatin structure and dynamics |
| D | Cell cycle control, cell division, chromosome partitioning |
| Y | Nuclear structure |
| V | Defense mechanisms |
| T | Signal transduction mechanisms |
| M | Cell wall/membrane/envelope biogenesis |
| N | Cell motility |
| Z | Cytoskeleton |
| W | Extracellular structures |
| U | Intracellular trafficking, secretion, and vesicular transport |
| O | Post-translational modification, protein turnover, chaperones |
| X | Mobilome: prophages, transposons |
| C | Energy production and conversion |
| G | Carbohydrate transport and metabolism |
| E | Amino acid transport and metabolism |
| F | Nucleotide transport and metabolism |
| H | Coenzyme transport and metabolism |
| I | Lipid transport and metabolism |
| P | Inorganic ion transport and metabolism |
| Q | Secondary metabolism biosynthesis, transport and catabolism |
| R | General function prediction only |
| S | Function unknown |

**Table S7. List of regulatory regions putatively involved in defining salt resistance at 600 mM NaCl.** COG description of supposed target orthologous genes of regulatory region hits identified by 146 top *k*-mers (P value =1.07e^-14^, P value = 1.11e^-14^ and P value = 1.01e^-13^) in the most salt-resistant strains at concentration of 600 mM (MO28(1), MO26(2), MO53(2), MO56(1) and BO21CC).

| **COG ID** | **COG classes** | **COG functional category** | **Prokka annotation / Product** | **Biological process** |
| --- | --- | --- | --- | --- |
| COG0161 | H | Adenosylmethionine-8-amino-7-oxononanoate aminotransferase | Putrescine - pyruvate aminotransferase  SpuC | Putrescine catabolism |
| COG0236 | IQ | Acyl carrier protein | Acyl carrier protein | Fatty acid and lipid biosynthesis |
| COG0251 | V | Enamine deaminase RidA, house cleaning of reactive enamine intermediates, YjgF/YER057c/UK114 family | RutC family protein | Unknown function |
| COG0381 | M | UDP-N-acetylglucosamine 2-epimerase | UDP-N-acetylglucosamine 2-epimerase | Cell wall modification |
| COG0406 | G | Broad specificity phosphatase PhoE | Acid phosphatase Gpm2 (or Fructose-1,6-bisphosphatase) | Carbohydrate metabolism  (gluconeogenesis) |
| COG0446 | I | NADPH-dependent 2,4-dienoyl-CoA reductase, sulfur reductase, or a related oxidoreductase | P-cumate 2,3-dioxygenase system, ferredoxin - NAD (+) reductase | Aromatic hydrocarbons catabolism |
| COG0528 | F | Uridylate kinase | Uridylate kinase | Pyrimidine nucleotide biosynthesis |
| COG0546 | C | Phosphoglycolate phosphatase, HAD superfamily | Phosphoglycolate phosphatase | Carbohydrate metabolism  (glycolate biosynthesis) |
| COG0596 | HR | Pimeloyl-ACP methyl ester carboxylesterase | 3-oxoadipate enol-lactonase 2 | Aromatic hydrocarbons catabolism  (beta-ketoadipate pathway) |
| COG0677 | M | UDP-N-acetyl-D-mannosaminuronate dehydrogenase | UDP-N-acetyl-D-glucosamine 6-dehydrogenase WbpA | Lipopolysaccharide biosynthesis  Cell wall modification |
| COG0757 | E | 3-dehydroquinate dehydratase | 3-dehydroquinate dehydratase AroQ | Aromatic amino acid biosynthesis |
| COG1088 | M | dTDP-D-glucose 4,6-dehydratase | UDP-glucose 4-epimerase | Carbohydrate metabolism  Galactose metabolic process |
| COG1793 | L | ATP-dependent DNA ligase | Multifunctional non-homologous end joining protein LigD | DNA recombination and repair |
| COG3957 | G | Phosphoketolase | Xylulose-5-phosphate phosphoketolase XpkA | Carbohydrate metabolism |

**Table S8. Gene Loci frequently identified by *k*-mers and the related numbers of similar sequences identified in the pangenome analysis.** In the column “ID_locus_tag” the gene loci is reported. In “Product”, the most frequently occurring functional annotation from the cluster is used. In “No. isolates” and “No. sequences”, the number of isolates represented in the genes cluster and the number of sequences in the cluster are reported, respectively. In the column “Putative Gene name” the most frequently occurring gene name from the sequences in the cluster is reported. If absence of gene name, a generic unique name starting with group_XXX is provided (column “non-unique gene name”).

| **Product** | **ID_locus tag** | **Putative gene name** | **Non-unique gene name** | **No. isolates** | **No. sequences** |
| --- | --- | --- | --- | --- | --- |
| Multifunctional non-homologous end joining protein LigD | MO26-2_05866 | ligD_5 |  | 49 | 49 |
| Multifunctional non-homologous end joining protein LigD | MO53-2_04847 | ligD_5 |  | 49 | 49 |
| Multifunctional non-homologous end joining protein LigD | MO56-1_04526 | ligD_5 |  | 49 | 49 |
| UDP-N-acetyl-D-glucosamine 6-dehydrogenase | BO21CC_04383 | wbpA_2 |  | 9 | 9 |
| UDP-N-acetyl-D-glucosamine 6-dehydrogenase | MO56-1_06151 | wbpA_2 |  | 9 | 9 |
| D-inositol-3-phosphate glycosyltransferase | BO21CC_04385 | mshA_17 |  | 9 | 9 |
| D-inositol-3-phosphate glycosyltransferase | MO56-1_06149 | mshA_17 |  | 9 | 9 |
| Autoinducer 2-binding protein LsrB | BO21CC_04339 | lsrB_4 |  | 49 | 49 |
| Autoinducer 2 import system permease protein LsrD | BO21CC_04340 | group_2255 | lsrD_3 | 49 | 49 |
| Autoinducer 2 import ATP-binding protein LsrA | BO21CC_04342 | lsrA_2 |  | 49 | 49 |
| Autoinducer 2-binding protein LsrB | MO56-1_04505 | lsrB_4 |  | 49 | 49 |
| Autoinducer 2 import system permease protein LsrD | MO56-1_04506 | group_2255 | lsrD_3 | 49 | 49 |
| Autoinducer 2 import ATP-binding protein LsrA | MO56-1_04508 |  | lsrA_2 | 49 | 49 |
| UDP-glucose 4-epimerase | BO21CC_04384 | galE1 |  | 9 | 9 |
| UDP-glucose 4-epimerase | MO56-1_06150 | galE1 |  | 9 | 9 |
| Malonyl CoA-acyl carrier protein transacylase | BO21CC_00713 | fabD |  | 49 | 49 |
| 50S ribosomal protein L4 | BO21CC_00937 | rplD |  | 49 | 49 |
| 1,4-alpha-glucan branching enzyme GlgB | BO21CC_02726 | glgB |  | 48 | 48 |
| Trehalose synthase / amylase TreS | BO21CC_03828 | treS |  | 48 | 48 |
| Electron transfer flavoprotein-ubiquinone oxidoreductase | BO21CC_05425 | group_10726 |  | 48 | 48 |
| Acetyl-CoA acetyltransferase | BO21CC_05673 | thlA |  | 33 | 33 |
| 3-oxoadipyl-CoA / 3-oxo-5,6-dehydrosuberyl-CoA thiolase | BO21CC_06139 | paaJ |  | 4 | 4 |
| Anhydromuropeptide permease | BO21CC_06220 | ampG_1 |  | 30 | 30 |
| HTH-type transcriptional regulator PgrR | BO21CC_06231 | group_4499 | pgrR_2 | 32 | 32 |
| putative oxidoreductase YjmC | BO21CC_06422 | yjmC_1 |  | 48 | 48 |
| Narbonolide / 10-deoxymethynolide synthase PikA2, modules 3 and 4 | BO21CC_06479 | pikAII |  | 2 | 2 |
| N-ethylmaleimide reductase | BO21CC_06482 | nemA_3 |  | 2 | 2 |
| Spermidine / putrescine import ATP-binding protein PotA | MO56-1_00208 | potA_2 |  | 49 | 49 |
| Methionine aminopeptidase | MO56-1_01298 | map_1 |  | 49 | 49 |
| Thioredoxin reductase | MO56-1_03145 | trxB_2 |  | 49 | 49 |
| Hydrogen cyanide synthase subunit HcnB | MO56-1_04118 | group_9644 | hcnB_1 | 3 | 3 |
| putative protein | MO56-1_04132 | group_18447 |  | 2 | 2 |
| Invasion protein InvA | MO56-1_04161 | invA |  | 3 | 3 |
| IS30 family transposase ISRle2 | BO21CC_05268 |  |  | 4 | 4 |

**Table S9. Unique gene loci of BO21CC and MO56(1) identified by *k*-mers.** In the column “ID_locus_tag” the gene loci is reported. In “Product”, the most frequently occurring functional annotation from the cluster is used. In “No. isolates” and “No. sequences”, the number of isolates represented in the genes cluster and the number of sequences in the cluster are reported, respectively. In the column “Putative Gene name” the most frequently occurring gene name from the sequences in the cluster is reported. If absence of gene name, a generic unique name starting with group_XXX is provided (column “non-unique gene name”).

| **ID_locus tag** | **Product** | **Putative gene name** | **Non-unique gene name** | **No. isolates** | **No. sequences** |
| --- | --- | --- | --- | --- | --- |
| BO21CC_01754 | Thiol-disulfide oxidoreductase ResA | group_17541 | resA | 1 | 1 |
| BO21CC_02524 | RNA polymerase sigma-54 factor 2 | group_17557 | rpoN2_2 | 1 | 1 |
| BO21CC_05174 | IS66 family transposase ISAzs18 | group_17597 |  | 1 | 1 |
| BO21CC_05182 | Phosphate acetyltransferase | pta_2 |  | 1 | 1 |
| BO21CC_05183 | Inosine-5'-monophosphate dehydrogenase | IMPDH_3 |  | 1 | 1 |
| BO21CC_05185 | Xylulose-5-phosphate phosphoketolase | xpkA_2 |  |  | 1 |
| BO21CC_05187 | Acid phosphatase | group_17610 | gpm2 | 1 | 1 |
| BO21CC_05188 | Cyclic beta-(1,2)-glucan synthase NdvB | group_17611 | ndvB_2 | 1 | 1 |
| BO21CC_06118 | Acetolactate synthase isozyme 3 large subunit | group_17691 | ilvI_2 | 1 | 1 |
| BO21CC_06121 | 2-formylbenzoate dehydrogenase | phdK |  | 1 | 1 |
| BO21CC_06124 | 4-carboxy-4-hydroxy-2-oxoadipate aldolase | group_17697 | ligK_2 | 1 | 1 |
| BO21CC_06127 | 3-dehydroquinate dehydratase | group_17700 | aroQ_2 | 1 | 1 |
| BO21CC_06131 | 3-dehydroquinate dehydratase | aroQ_3 |  | 1 | 1 |
| BO21CC_06133 | Hydrogen peroxide-inducible genes activator | group_17706 | oxyR_4 | 1 | 1 |
| BO21CC_06135 | Glucose--fructose oxidoreductase | group_17708 | gfo | 1 | 1 |
| BO21CC_06137 | 3-oxoacyl- reductase FabG | group_17710 | fabG_17 | 1 | 1 |
| BO21CC_06144 | 3-oxoadipate enol-lactonase 2 | group_17715 | catD_2 | 1 | 1 |
| BO21CC_06468 | Low affinity potassium transport system protein kup | kup_6 |  | 1 | 1 |
| MO56-1_04192 | Modification methylase Eco57IB | group_23256 |  | 1 | 1 |
| MO56-1_04193 | Phosphoglycolate phosphatase | gph_4 |  | 1 | 1 |
| MO56-1_04485 | Polysialic acid transport protein KpsM | group_23261 | kpsM_1 | 1 | 1 |
| MO56-1_04493 | Ubiquinone biosynthesis O-methyltransferase | COQ3_5 |  | 1 | 1 |
| MO56-1_04807 | Tyrosine recombinase XerC | group_23276 | xerC_3 | 1 | 1 |
| MO56-1_05834 | Putrescine--pyruvate aminotransferase | group_23298 | spuC_4 | 1 | 1 |
| MO56-1_05836 | Inner membrane ABC transporter permease protein YcjO | ycjO_5 |  | 1 | 1 |
| MO56-1_05837 | Trehalose transport system permease protein SugB | group_23301 | sugB_5 | 1 | 1 |
| MO56-1_05838 | glycerol-3-phosphate import ATP-binding protein UgpC | ugpC_15 |  | 1 | 1 |
| MO56-1_05847 | Nucleoside diphosphate kinase | ndk_2 |  | 1 | 1 |
| MO56-1_05849 | Glutamate-1-semialdehyde 2,1-aminomutase | group_23308 | hemL_1 | 1 | 1 |
| MO56-1_05850 | Fructose dehydrogenase large subunit | group_23309 | fdhL | 1 | 1 |
| MO56-1_05932 | Glucosamine kinase GspK | gspK_2 |  | 1 | 1 |
| MO56-1_05933 | HTH-type transcriptional repressor NagR | group_23314 | nagR_4 | 1 | 1 |
| MO56-1_05936 | D-xylulose reductase | group_23317 |  | 1 | 1 |
| MO56-1_05937 | Phosphoglycolate phosphatase | group_23318 | gph_5 | 1 | 1 |
| MO56-1_05938 | HTH-type transcriptional regulator MurR | group_23319 | murR_2 | 1 | 1 |
| MO56-1_05940 | Lactose transport system permease protein LacF | group_23321 | lacF_8 | 1 | 1 |
| MO56-1_05941 | L-arabinose transport system permease protein AraQ | group_23322 | araQ_5 | 1 | 1 |
| MO56-1_05942 | glycerol-3-phosphate-binding periplasmic protein UgpB | group_23323 | ugpB_3 | 1 | 1 |
| MO56-1_05943 | 3,5'-cyclic adenosine monophosphate phosphodiesterase CpdA | group_23324 | cpdA_4 | 1 | 1 |
| MO56-1_05948 | Thiol-disulfide oxidoreductase ResA | group_23328 | resA | 1 | 1 |

**Table S10. The maximum areas values of kinetic curves of negative controls in Phenotype Microarray analysis.** Values of area reported were obtained for all conditions tested on carbon sources of PM1 and PM2.

| **Plates** | **0 mM** | | | **3000 mM** | | |
| --- | --- | --- | --- | --- | --- | --- |
|  | **BO21CC** | **MO56(1)** | **RU11/001** | **BO21CC** | **MO56(1)** | **RU11/001** |
| **PM1** | 10240 | 10763 | 9393 | 12948 | 12225 | 11946 |
| **PM2** | 10732 | 12713 | 8694 | 12563 | 11239 | 12587 |

**Table S11. Report on PM1-2 carbon sources used by BO21CC and MO56(1) under 0 mM and 300 mM NaCl.** The total number of carbon sources tested with PM1 and PM2, and the percentage of sources used under 300 mM NaCl compared to 0 mM are reported.

| **Classes of sources** | **Total n° of sources** | **NaCl-R^+^** | | | | **NaCl-R^-^** | |
| --- | --- | --- | --- | --- | --- | --- | --- |
|  |  | **BO21CC** | | **MO56(1)** | | **RU11/001** | |
|  |  | **0 mM** | **300 mM** | **0 mM** | **300 mM** | **0 mM** | **300 mM** |
| Alcohols | 6 | 3 | 1 | 3 | 1 | 2 | 1 |
| Amine & Amidi | 8 | 0 | 0 | 0 | 0 | 0 | 0 |
| Amino acids | 30 | 12 | 3 | 18 | 6 | 15 | 5 |
| Carbohydrates | 70 | 53 | 42 | 57 | 43 | 51 | 38 |
| Carboxylic acids | 60 | 21 | 9 | 23 | 9 | 14 | 7 |
| Ester & Fatty acids | 5 | 2 | 0 | 2 | 0 | 2 | 0 |
| Polymers | 11 | 3 | 3 | 5 | 2 | 2 | 2 |
| Total of sources |  | 94 | 58 | 108 | 61 | 86 | 53 |

**SUPPLEMENTARY FILES**

**File S1. (file excel) DDH table**

**File S2. (file excel)** **The gene presence and absence matrix of *S. meliloti* pangenome.**

**File S3. (file excel) Grouping of mean values of normalized metabolic activity for each *S. meliloti* strain at 200 mM, 400 mM, 600 mM, 700 mM, 800 mM, 900 mM and 1 M NaCl.** Different letters indicate statistically significant groupings based on the Scott Knott tests (p-value < 0.05).

**File S4. (file excel) List of top *k*-mers** for a) 400 mM b) 600 mM.

**File S5. (file excel) Genes hits identified by the best *k*-mers at 600 mM dataset.**

**File S6. (file excel) regulatory region hits identified by the best *k*-mers for 600 mM dataset.**

**File S7. (file word)** **Metabolic activity values of strains NaCl-R+ (BO21CC and RU11/001) and NaCl-R- (RU11/001) in presence and absence of NaCl (0 mM and 300 mM NaCl) on unused sources with an area smaller than 13000 AOU on PM1 and PM2 plates.**

**File S8. (file word)** **Metabolic activity values of strains NaCl-R+ (BO21CC and RU11/001) and NaCl-R- (RU11/001) in presence and absence of NaCl (0 mM and 300 mM NaCl) on some carbon sources putatively involved in salt resistance.**
