# Supplementary figures and images for: Genomic and Phenotypic Bases of Salt Tolerance in Sinorhizobium meliloti : Candidate Traits for Bioinoculant Development Addressing Saline Soils
Source: Microb Biotechnol. 2026 Jan 29;19(1):e70304. doi: 10.1111/1751-7915.70304 (PMC12855168; doi:10.1111/1751-7915.70304)

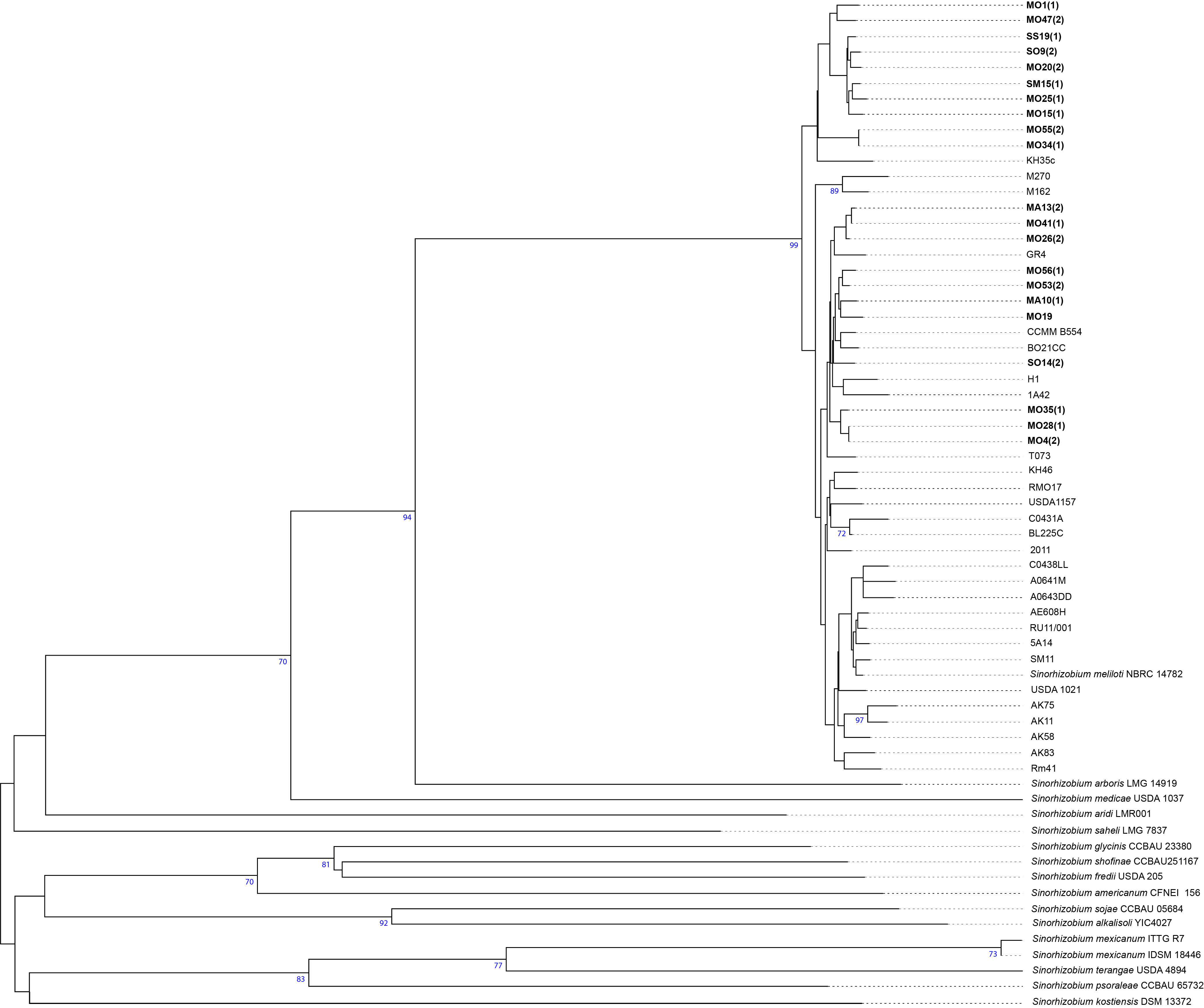

Supplement: Supplementary file 3 — Figure S1: ‘Phylogram’: Tree inferred with FastME from GBDP distances calculated from genome sequences. The branch lengths were scaled in terms of GBDP distance formula d 5 . The numbers above branches are GBDP pseudo‐bootstrap support values > 60% from 100 replications, with an average branch support of 28.2%. Algerian strains were highlighted in bold. [file MBT2-19-e70304-s007.png]

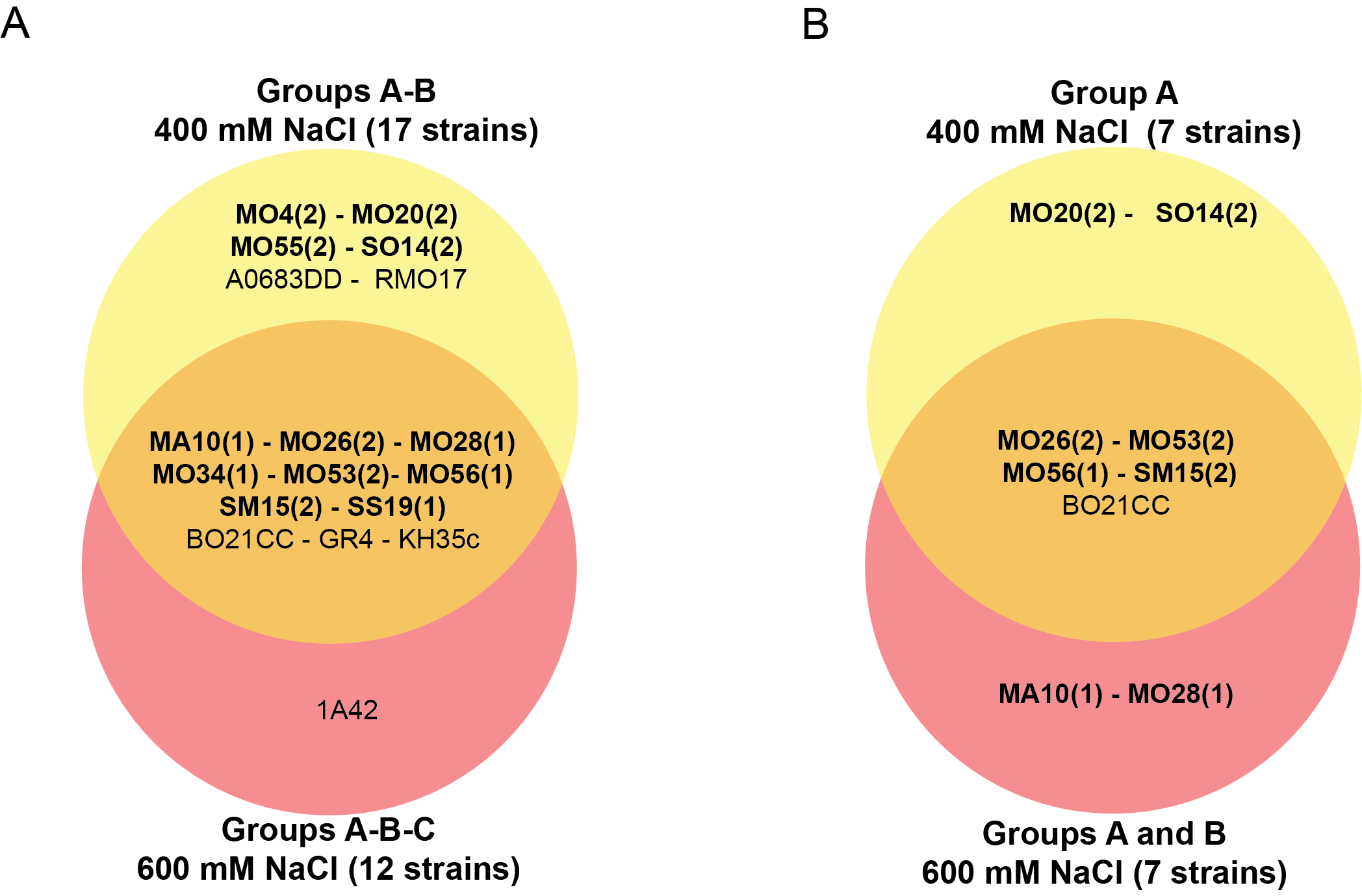

Supplement: Supplementary file 4 — Figure S2: mbt270304‐sup‐0004‐FigureS2.png. Sinorhizobium meliloti strains with high salt resistance at 400 and 600 mM NaCl. (A) Venn diagram between the groups characterised by a higher AOU at 400 mM (groups A and B) and 600 mM NaCl (Groups A, B and C). (B) Venn diagram with the top AOU groups at 400 mM (groups A) and 600 mM NaCl (Groups A and B). Algerian strains were highlighted in bold. [file MBT2-19-e70304-s002.png]

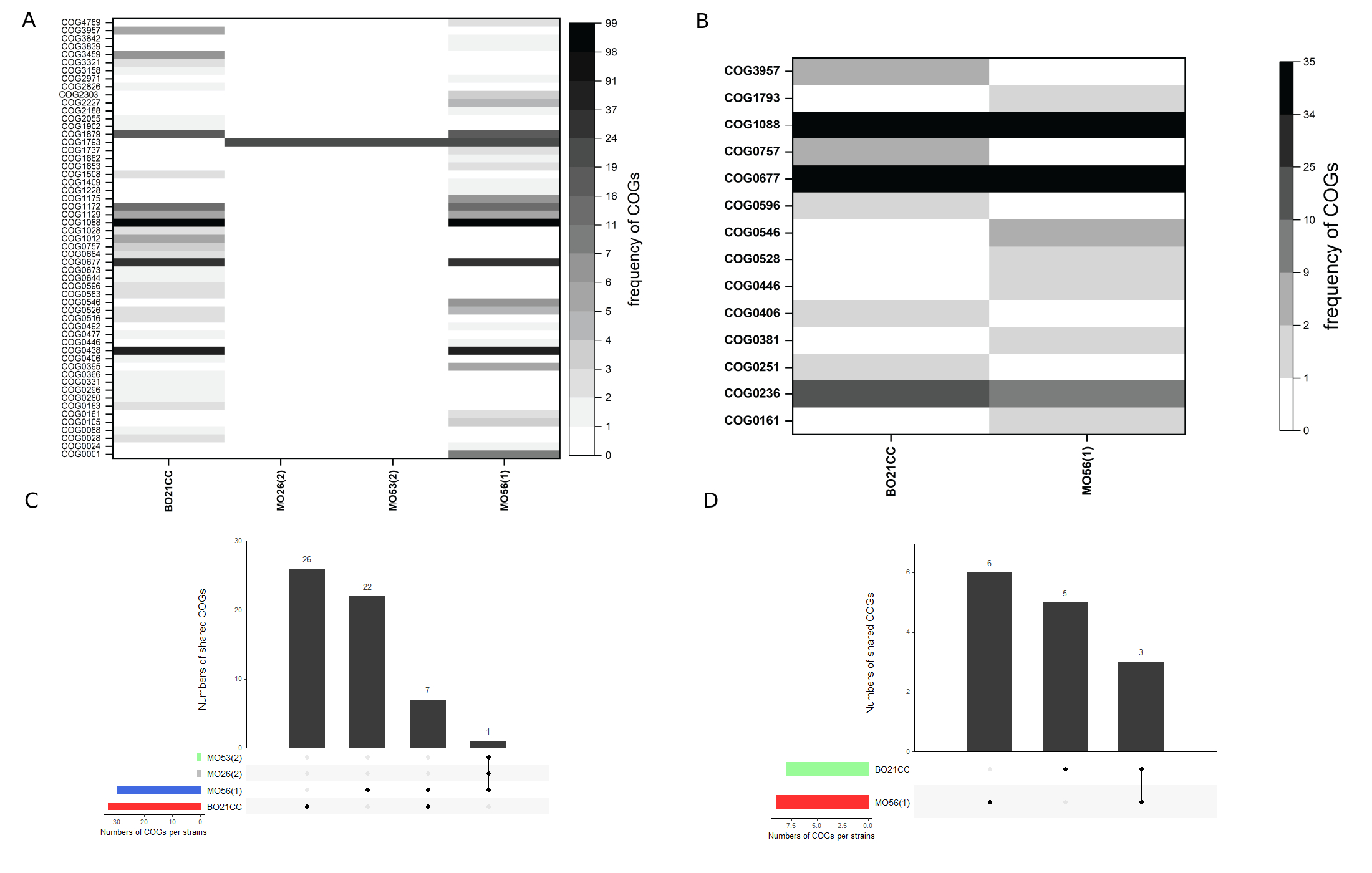

Supplement: Supplementary file 5 — Figure S3: Functions associated with the salt‐resistant phenotype. Frequency of candidate functions of gene hits (A) and regulatory regions (B) identified by best k‐mers in the most salt‐resistant strains. The frequency of candidate functions reported as COG annotations (rows) in each strain (columns) is represented by grayscale shades. In the upset plots, (C) the number of shared functions of gene hits and (D) shared functions of regulatory regions, in each and different combinations of strains are reported. [file MBT2-19-e70304-s005.jpg]

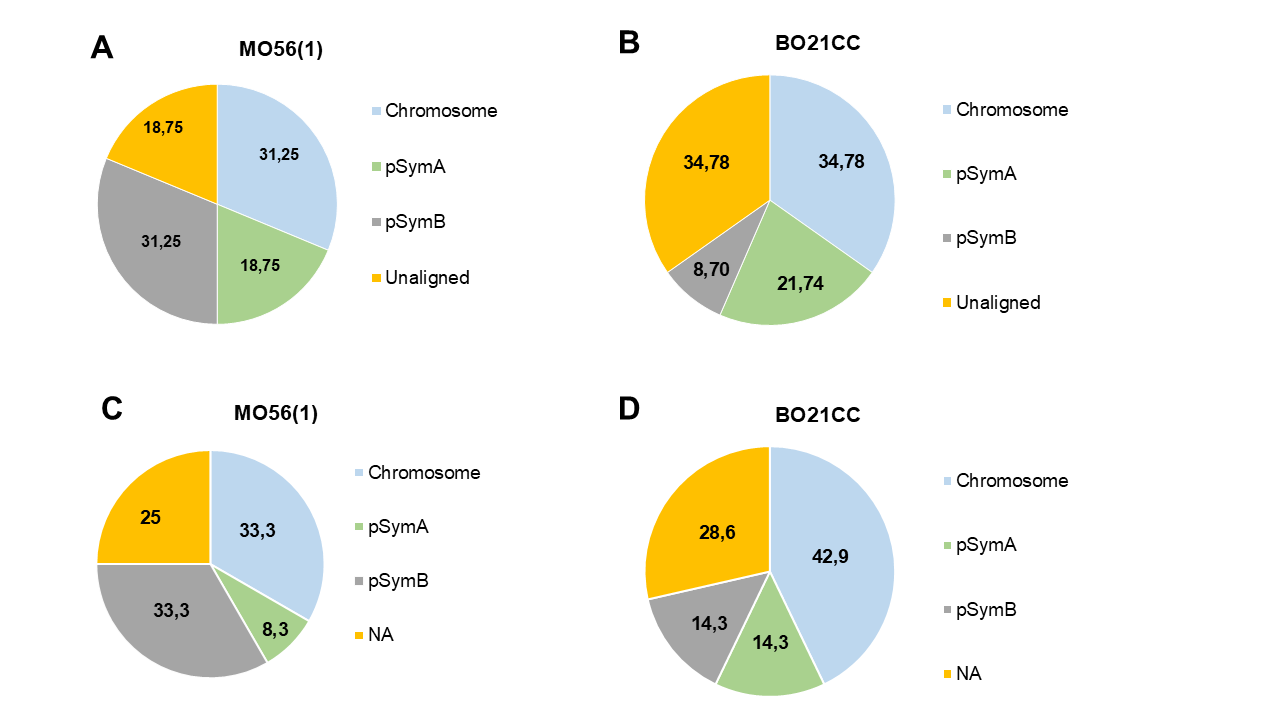

Supplement: Supplementary file 6 — Figure S4: Distribution of scaffolds hit by best k‐mers among different replicons in Sinorhizobium meliloti MO56(1) and BO21CC through the alignment with the genome of S. meliloti 2011. Distribution of k‐mers‐cointaining scaffolds of MO56(1) (A&C) and BO21CC (B & D) mapped on the genome of 2011 (RefSeq assembly GCF_000346065.1) referring to both gene hits (A, B) and regulatory region hits (C, D). Data are reported as percentage on the total number of considered scaffolds in each dataset. Scaffold reported as Unknown could not be aligned on the reference genome. [file MBT2-19-e70304-s006.png]

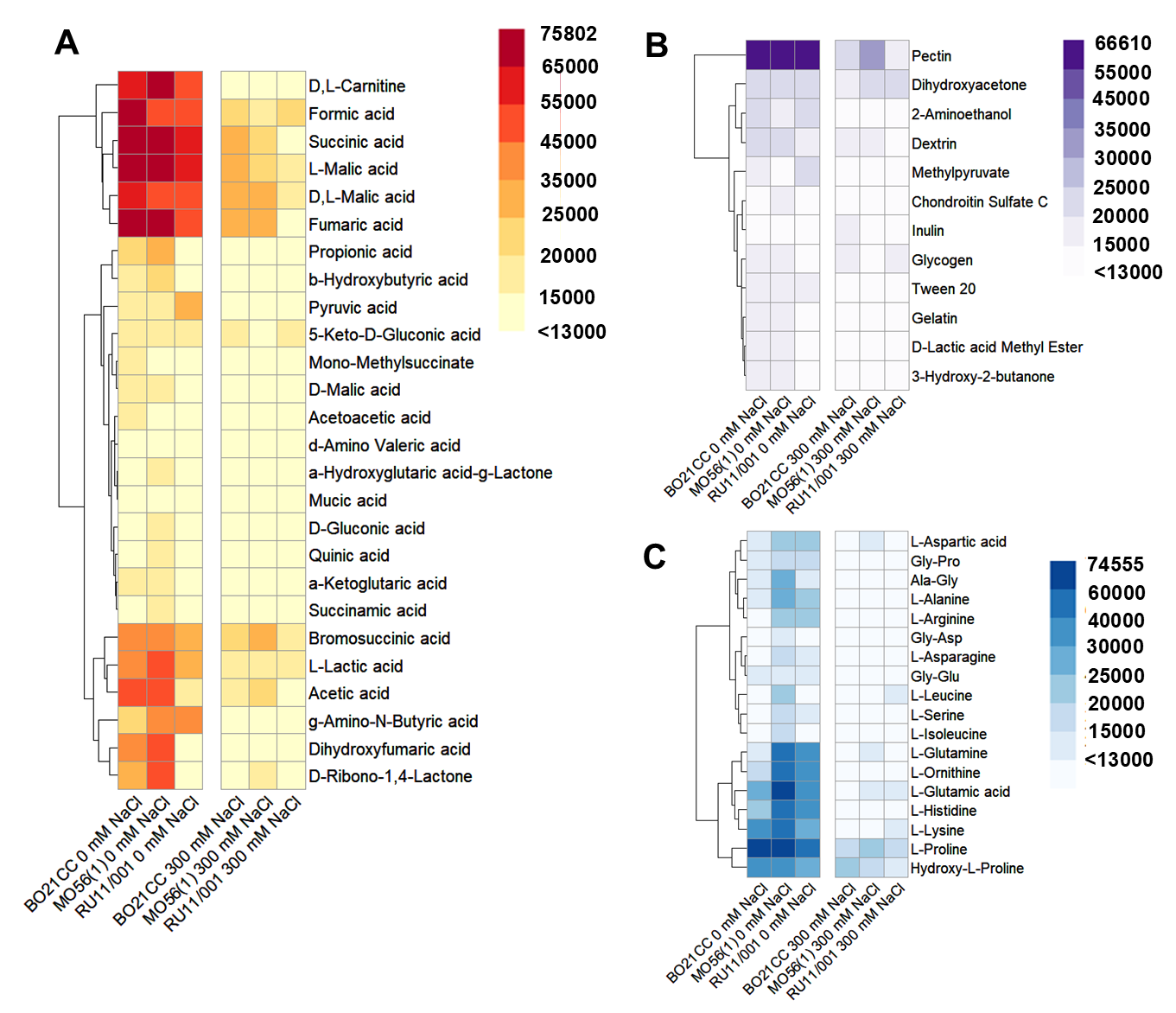

Supplement: Supplementary file 7 — Figure S5: Metabolic activities on carboxylic acid, amino acid and other sources of S. meliloti strains BO21CC, MO56(1) and RU11/001 at 0 and 300 mM NaCl. Carboxylic acid (A), amino acid (C) and others (B) were used as carbon sources. Other sources refer to alcohols, ester & fatty acids, polymers, amine & amide. The metabolic activities were expressed and reported as area of the kinetic curves for each condition. Hierarchical clustering with complete linkage of S. meliloti strains was reported. [file MBT2-19-e70304-s013.png]
