# Supplementary material for: Genomic and Phenotypic Bases of Salt Tolerance in Sinorhizobium meliloti : Candidate Traits for Bioinoculant Development Addressing Saline Soils
Source: Microb Biotechnol. 2026 Jan 29;19(1):e70304. doi: 10.1111/1751-7915.70304 (PMC12855168; doi:10.1111/1751-7915.70304)
Supplement: Supplementary file 13 — File S7: Metabolic activity values of strains NaCl‐R+ (BO21CC and RU11/001) and NaCl‐R‐ (RU11/001) in presence and absence of NaCl (0 mM and 300 mM NaCl) on unused sources with an area smaller than 13000 AOU on PM1 and PM2 plates. [file MBT2-19-e70304-s001.docx]

**File S7**

**Metabolic activity values of strains** **NaCl-R+ (BO21CC and RU11/001) and NaCl-R- (RU11/001) in presence and absence of NaCl (0 mM and 300 mM NaCl) on unused sources with an area smaller than 13000 AOU on PM1 and PM2 plates.**

**PM1:**

**m-Tartaric acid**

**
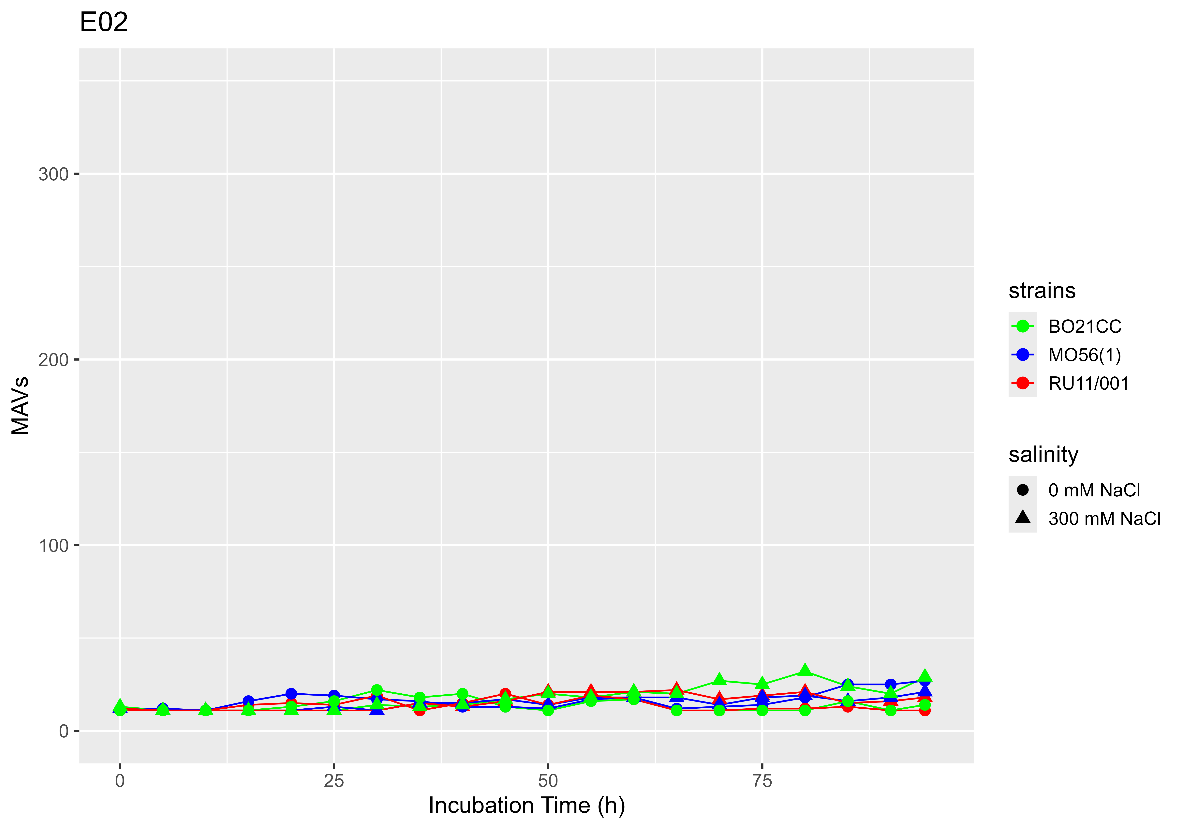
**

**D-Glucose-1-Phosphate**

**
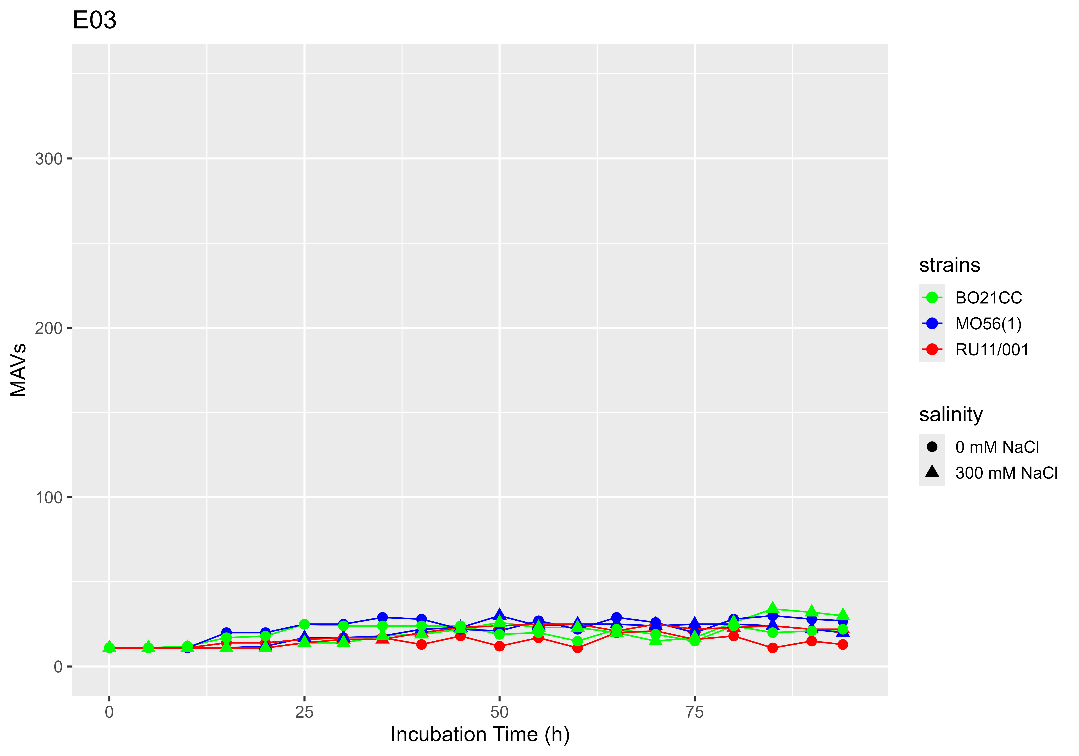
**

**
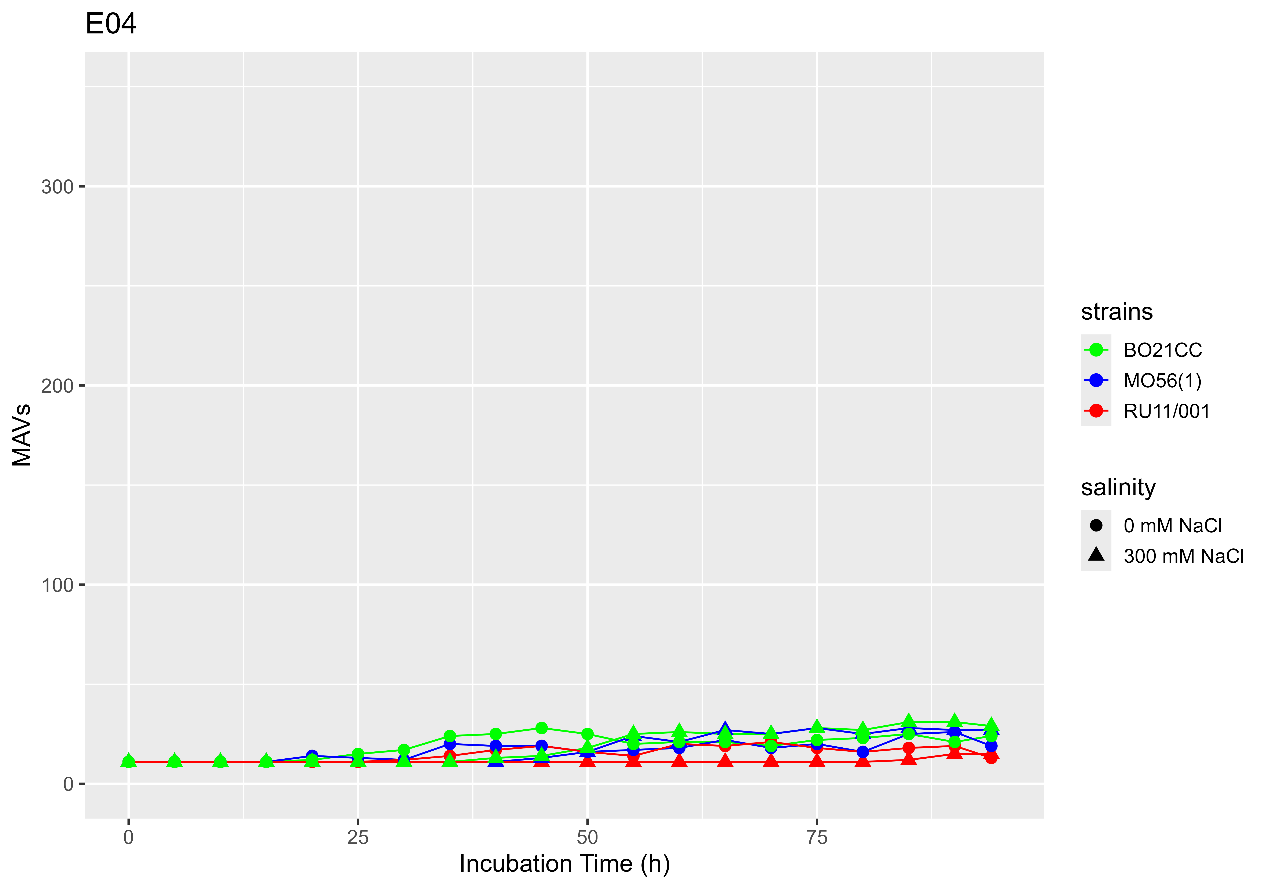
D-Fructose-6-Phosphate**

**Tween 80**

**
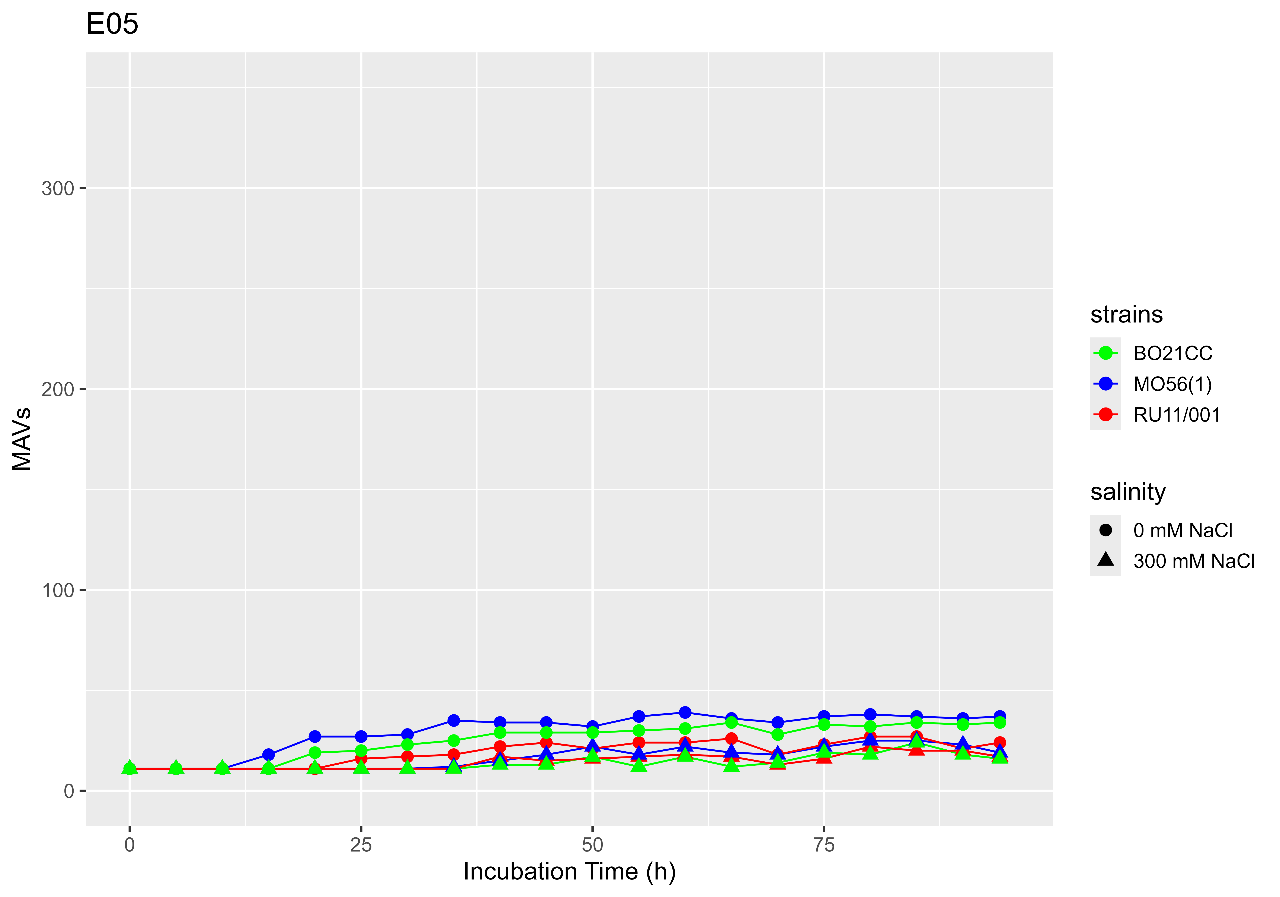
**


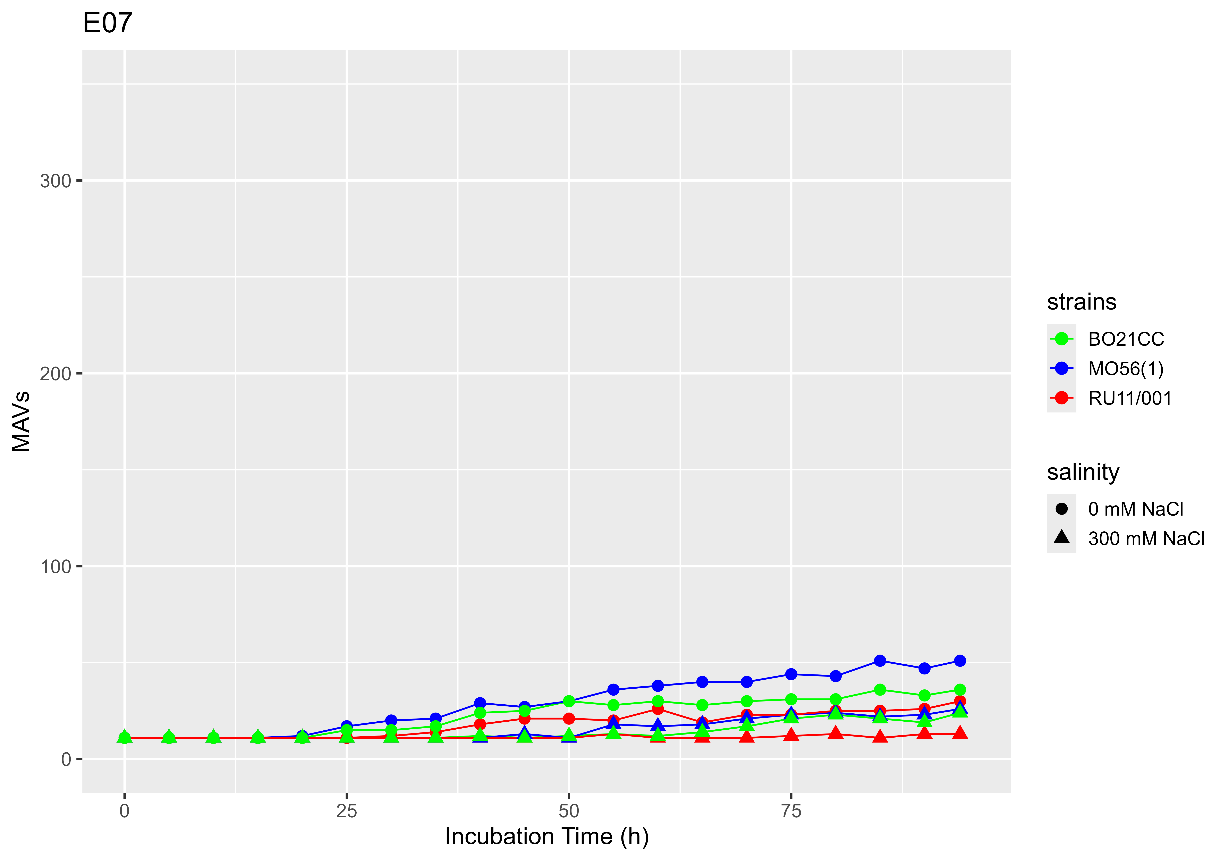
**α-Hydroxybutyric acid**

**2`-Deoxyadenosine**

**
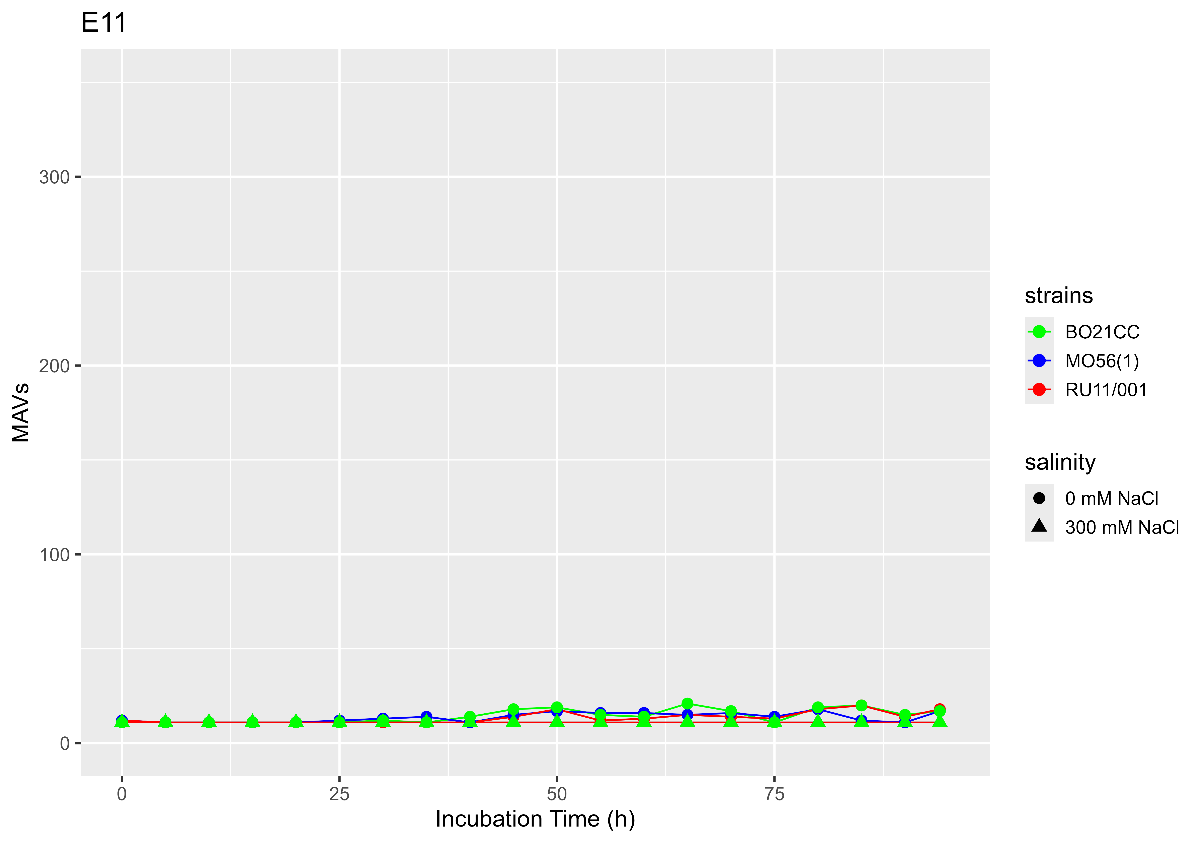
**

**Citric acid**

**
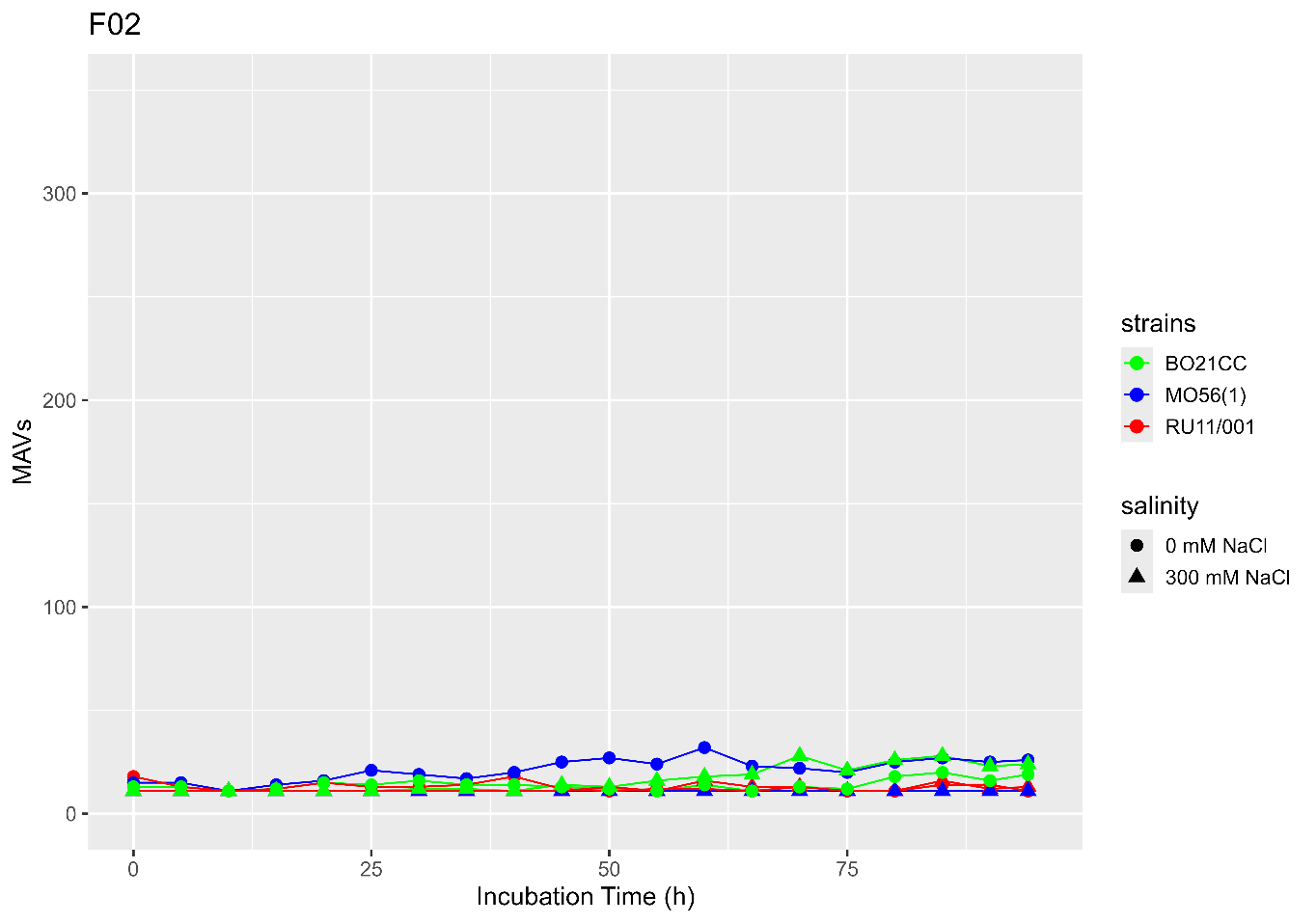
**

**
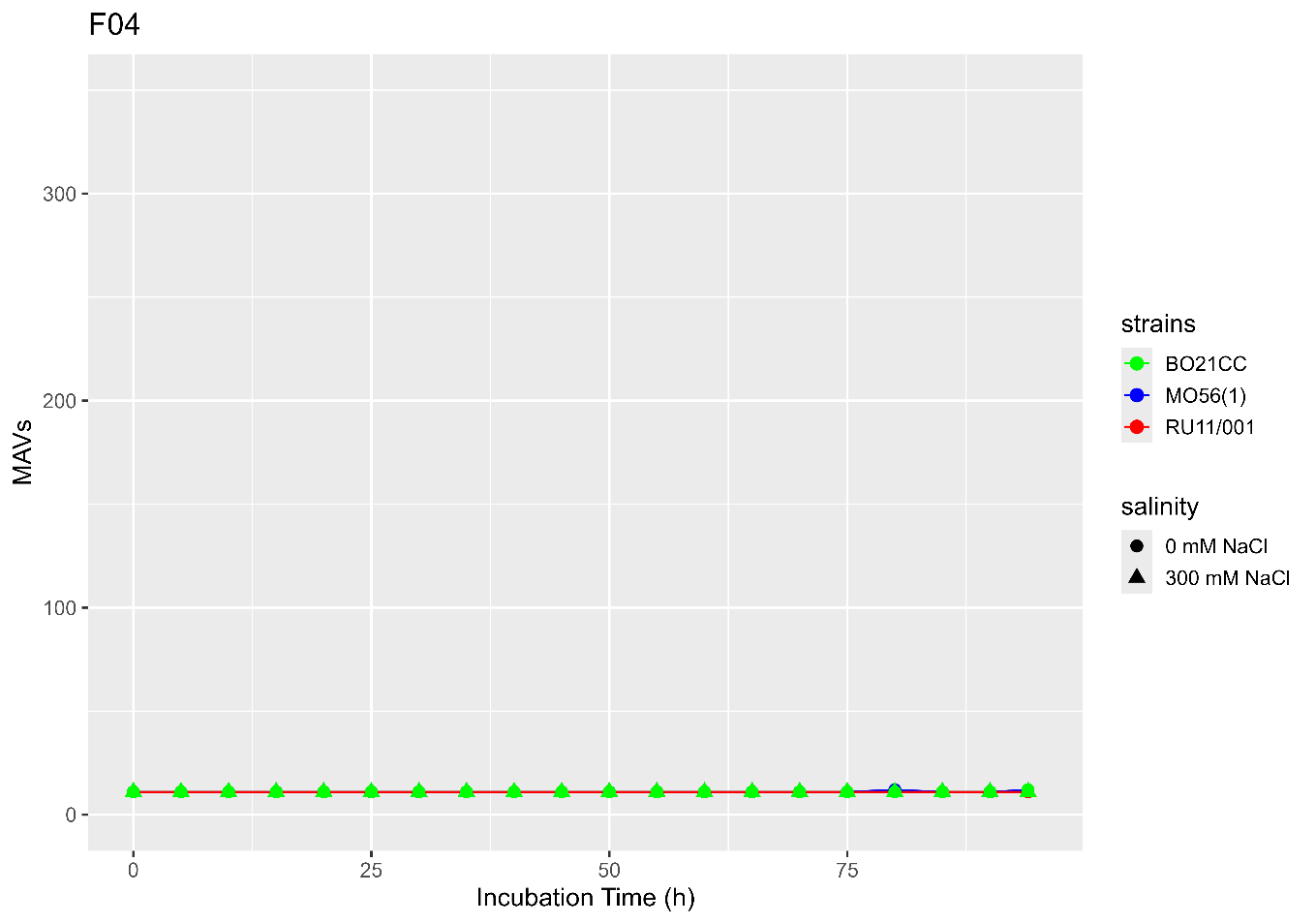
D-Threonine**

**
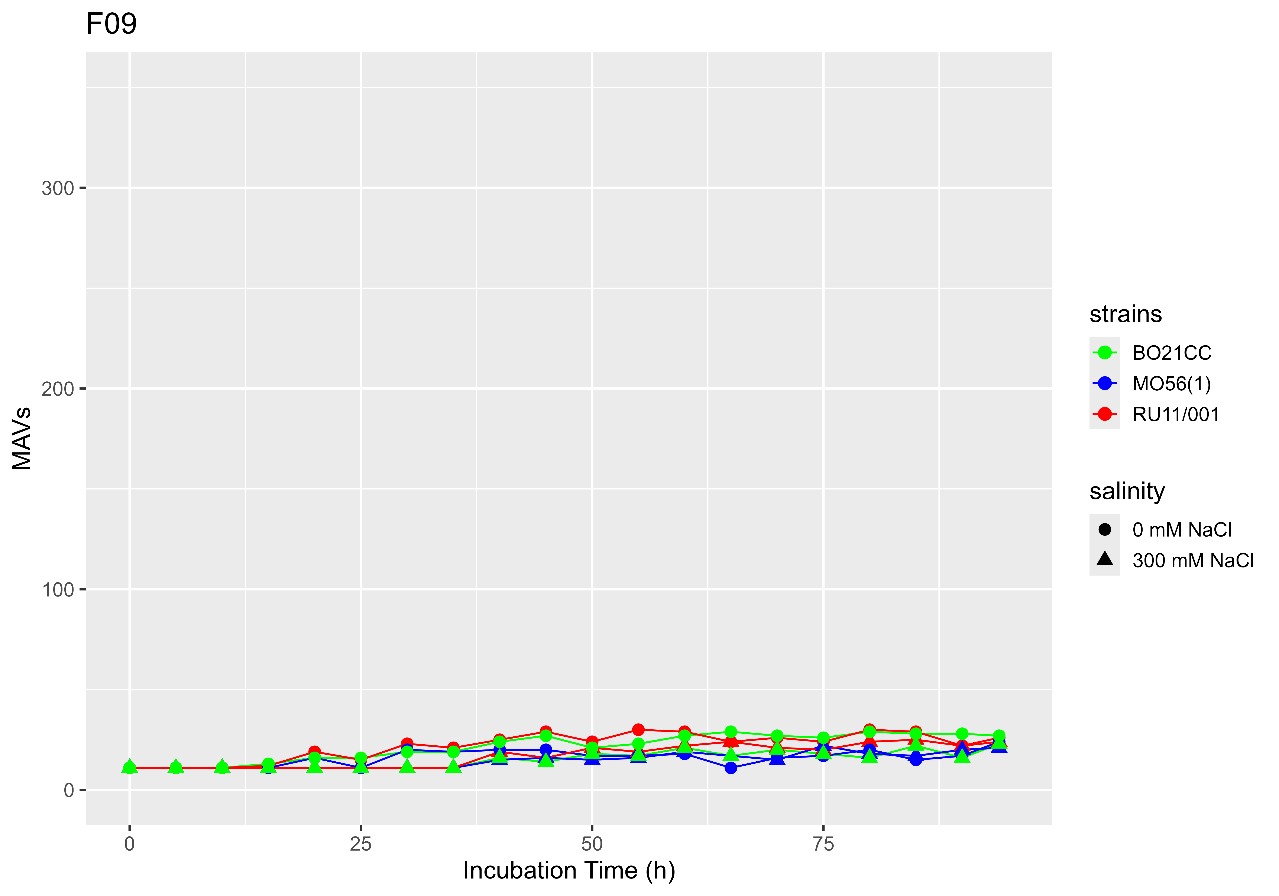
Glycolic acid**

**Glyoxylic acid**

**
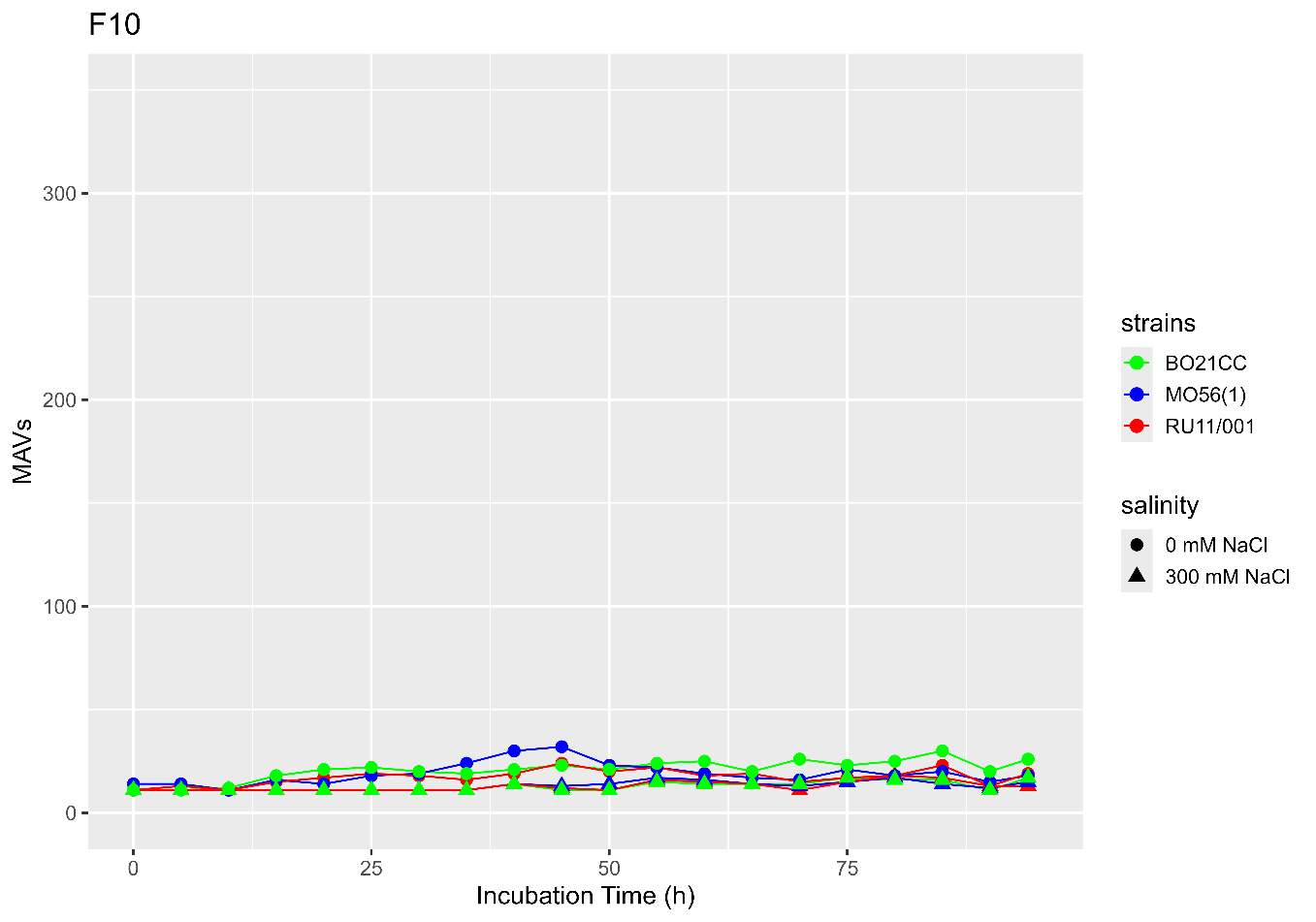
**

**Inosine**

**
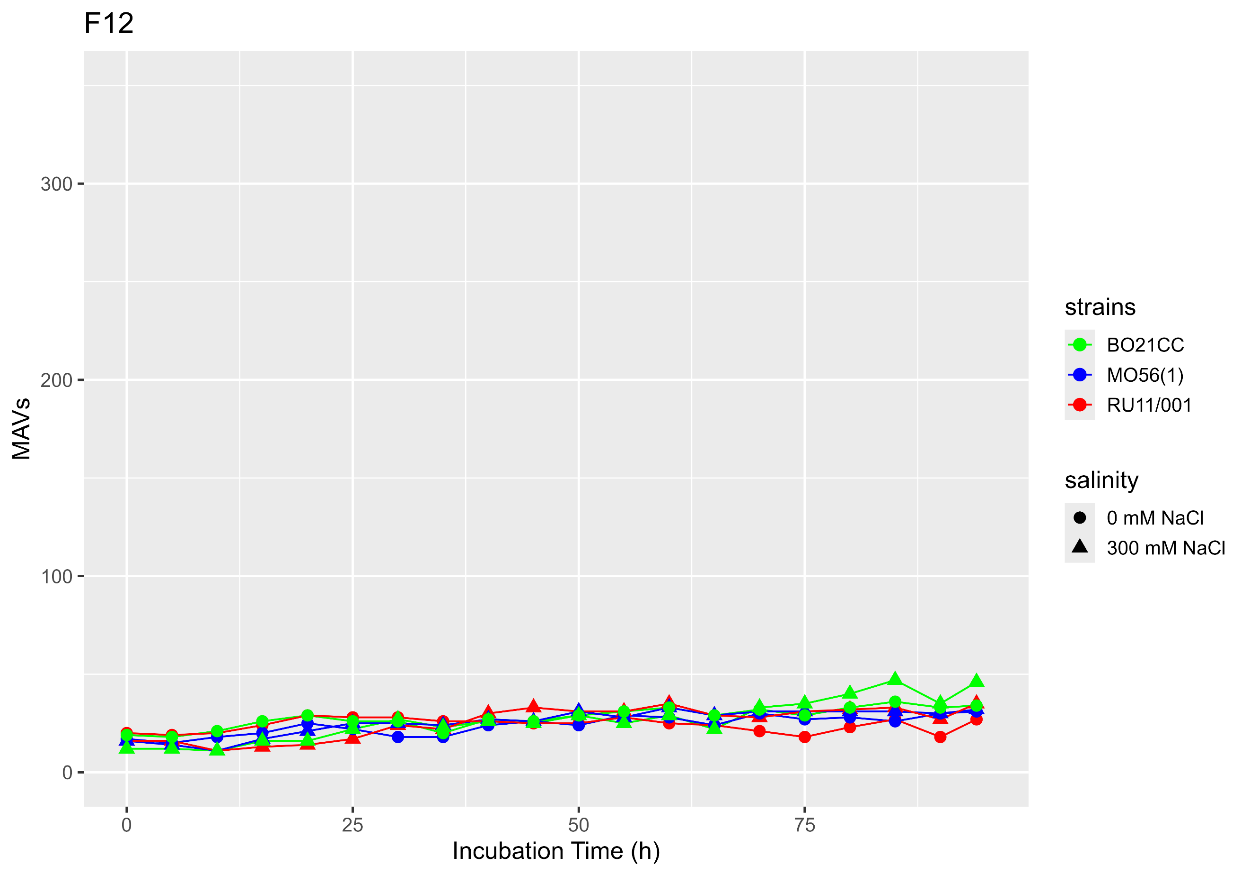
Inosine**

**Tricarballylic acid**

**
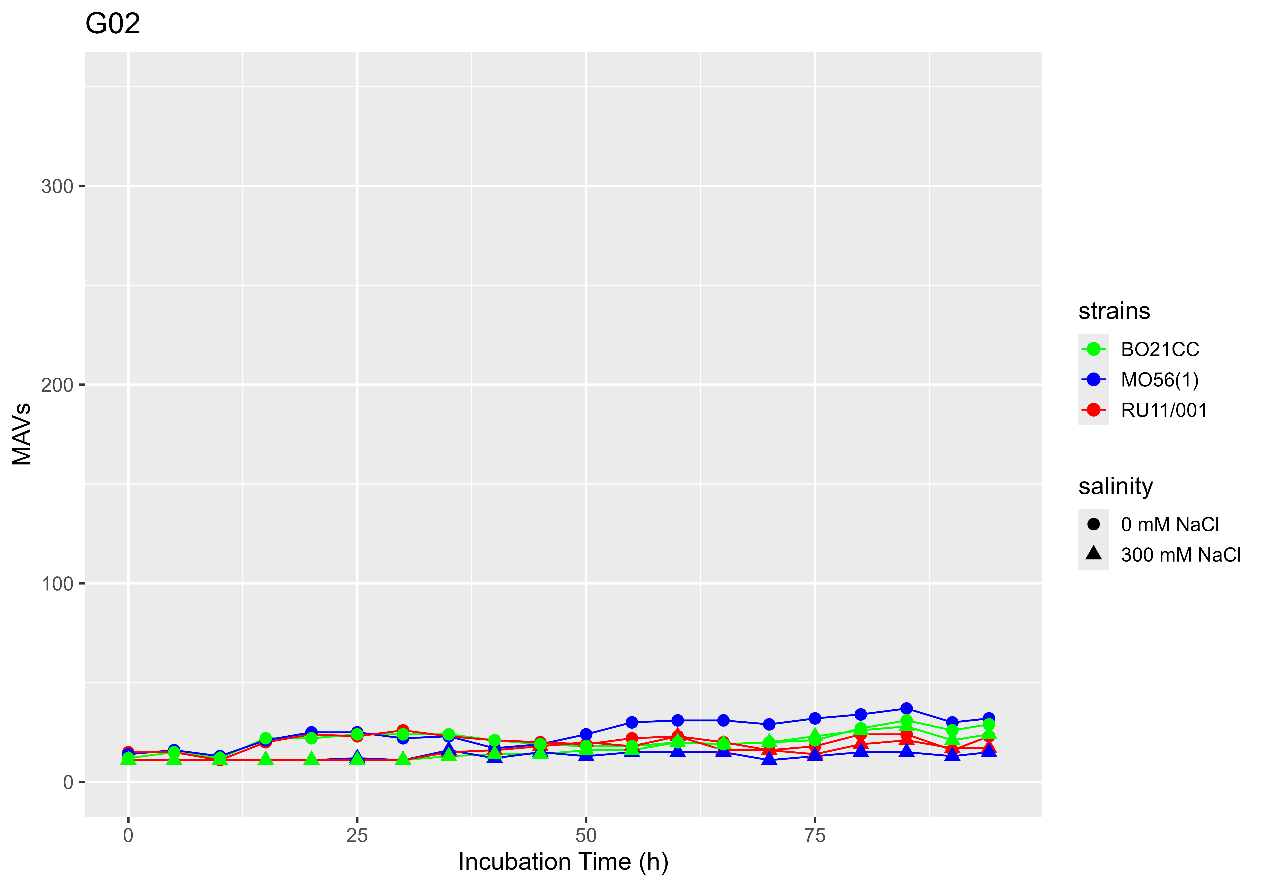
**

**L-Threonine**

**
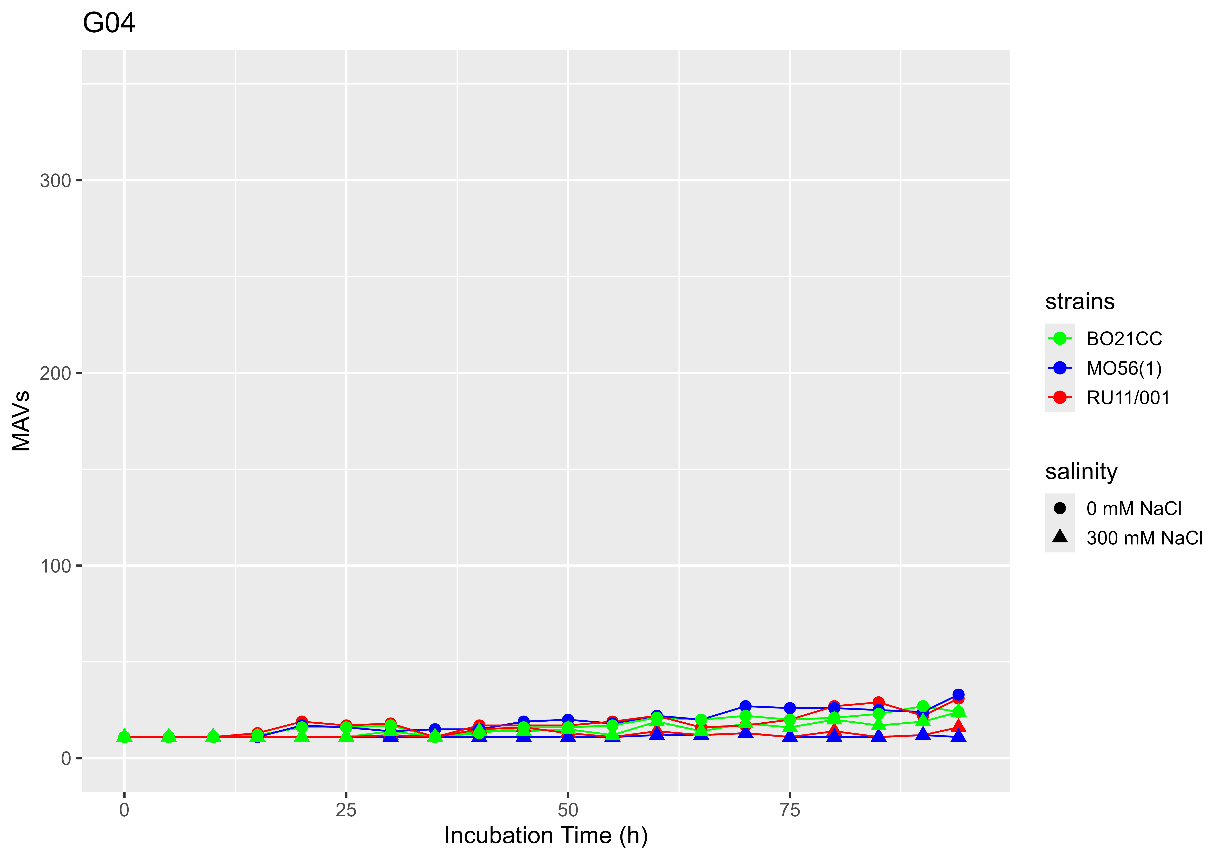
**

**N-Acetyl-D-Mannosamine**

**
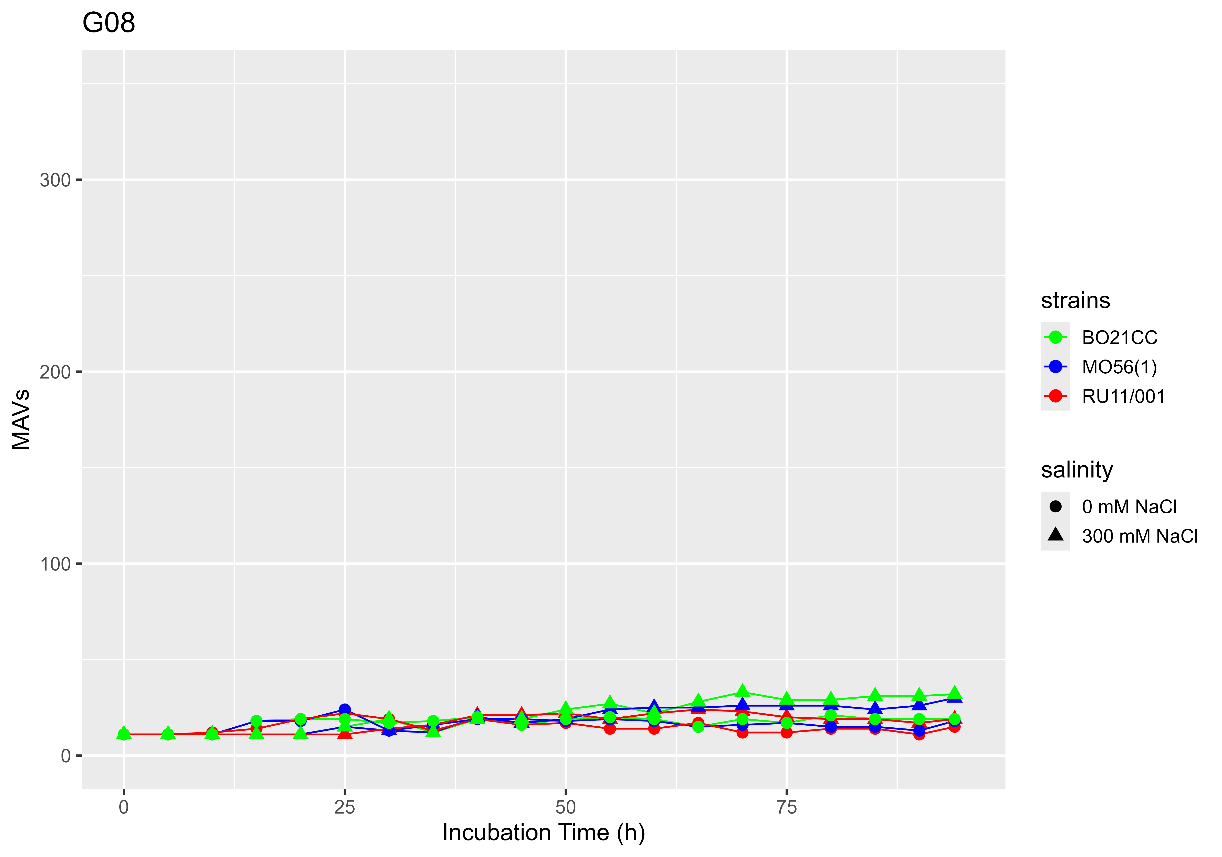
**

**
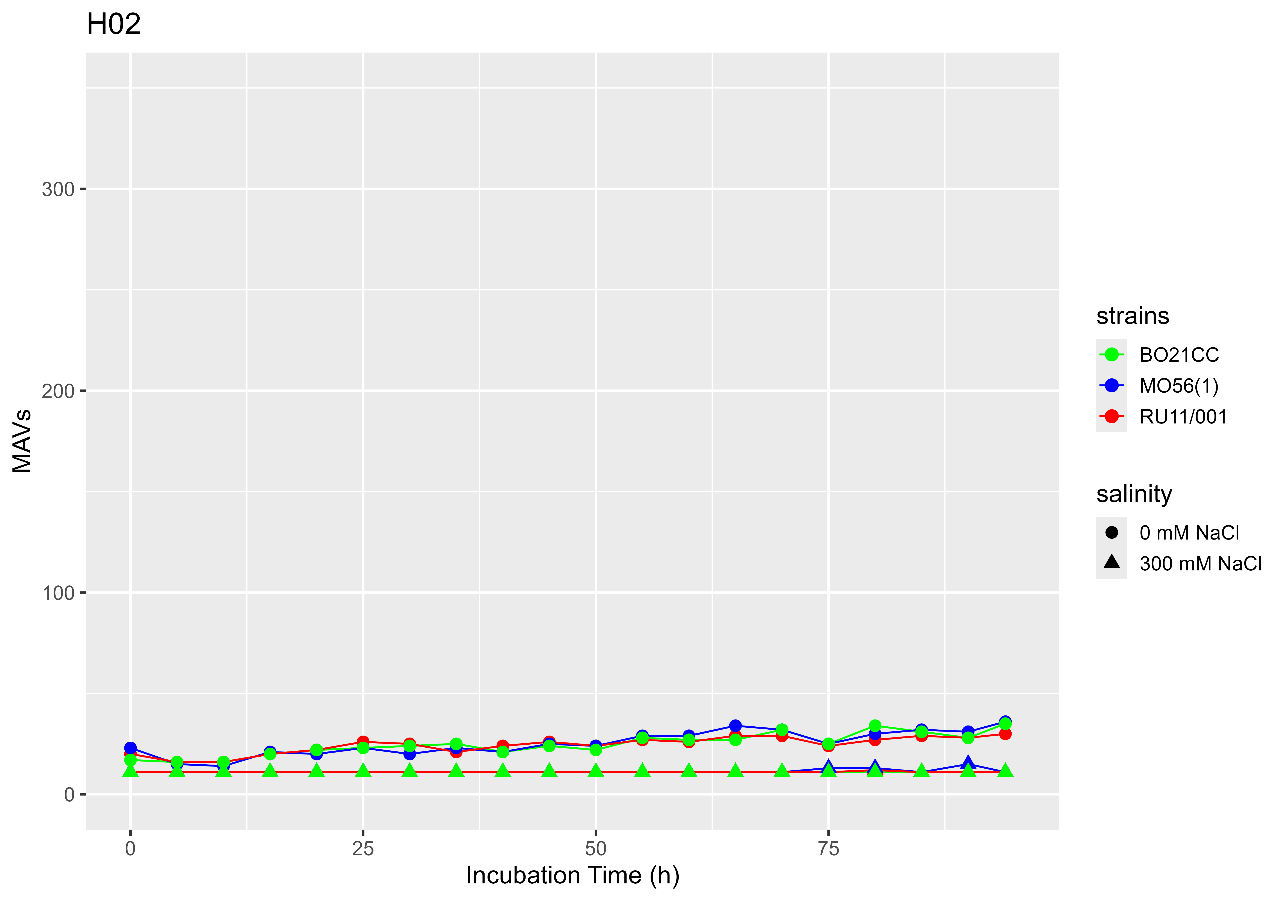
p-Hydroxyphenyl Acetic acid**

**
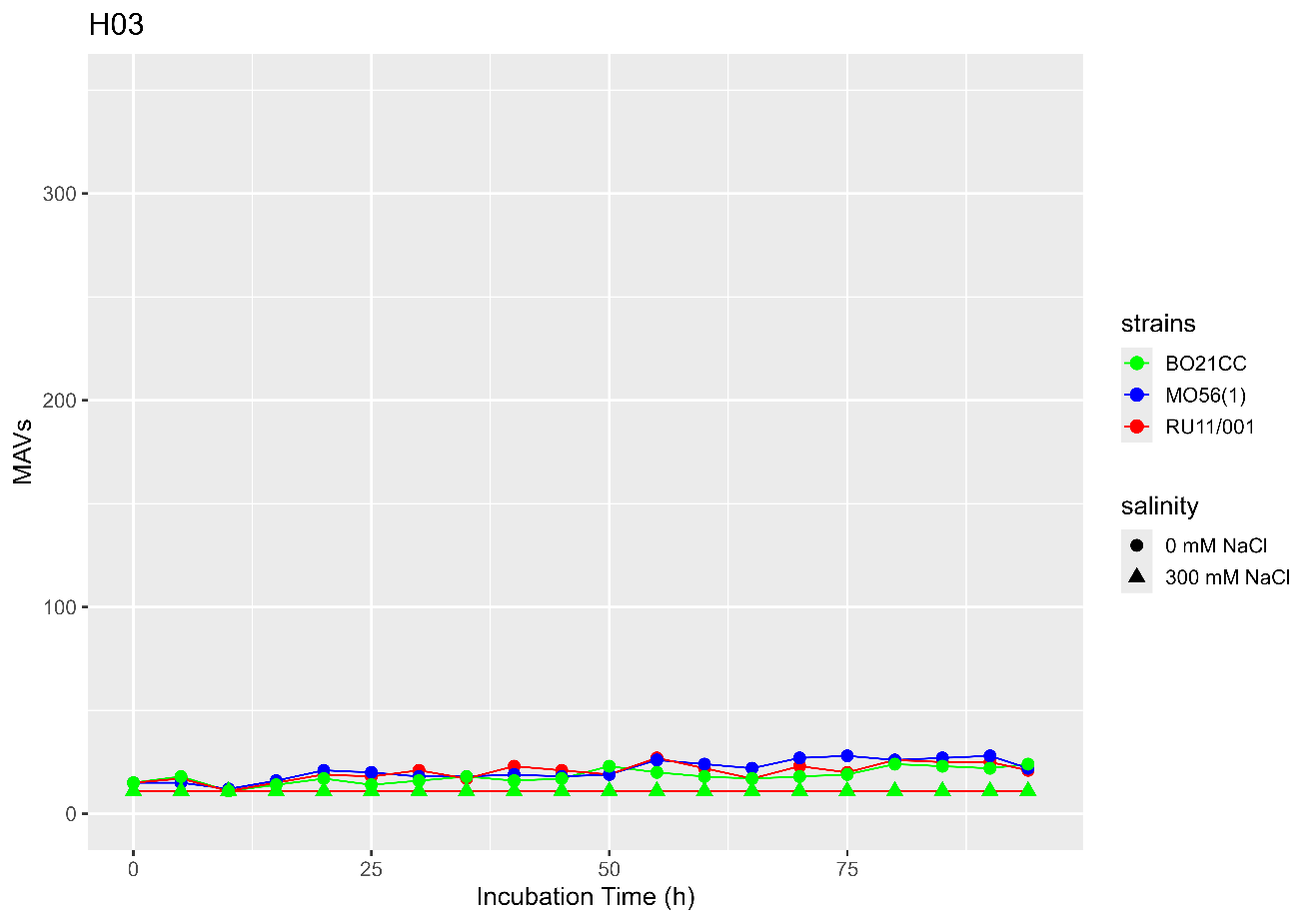
m-Hydroxyphenyl Acetic acid**

**
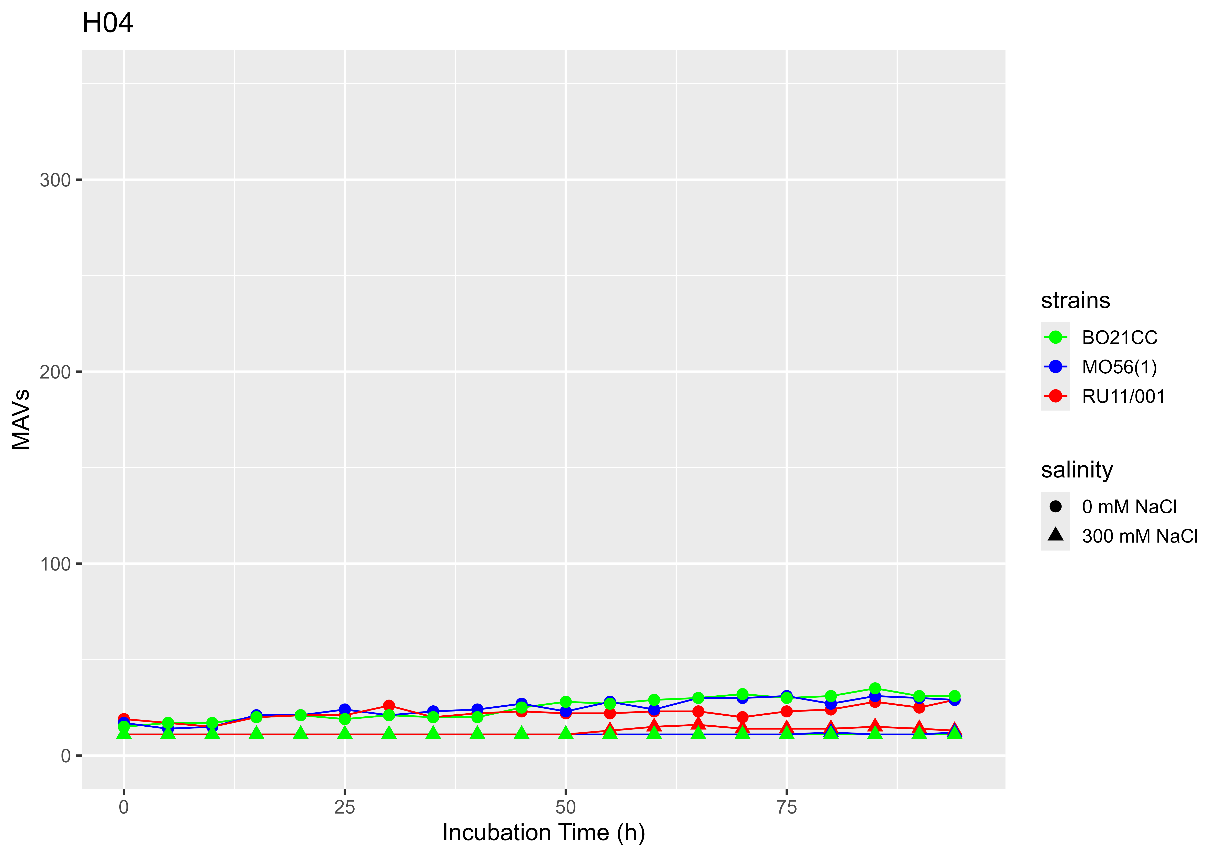
Tyramine**

**Glucuronamide**

**
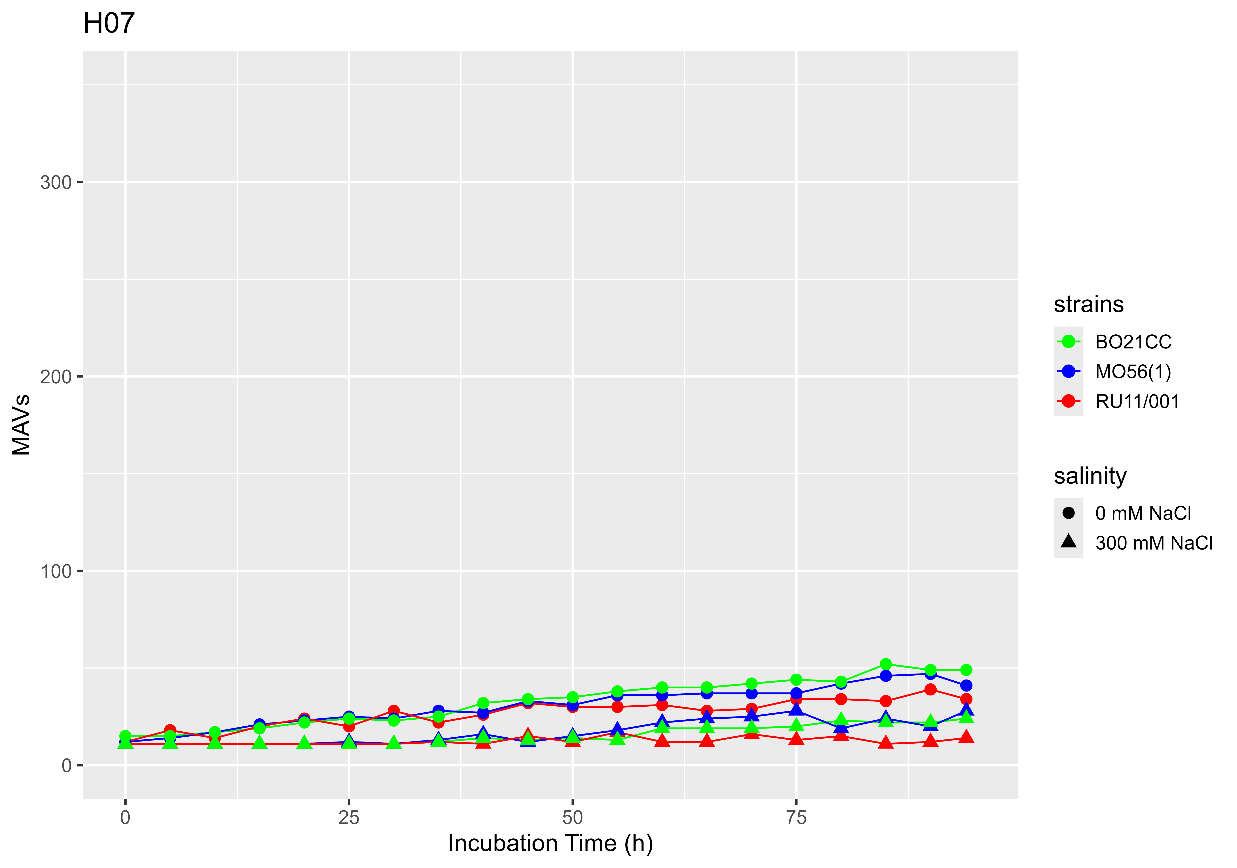
**

**
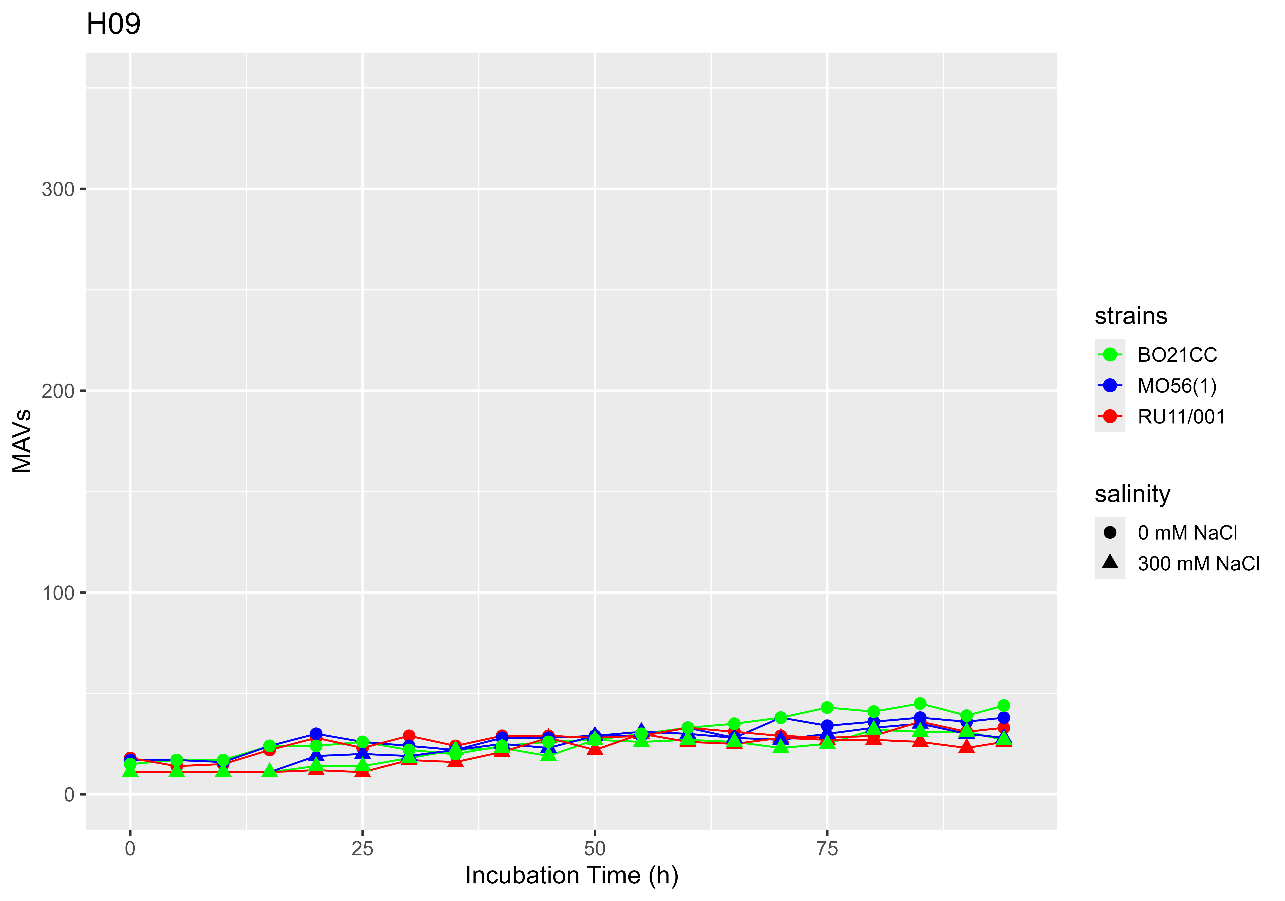
L-Galactonic acid-γ-Lactone**

**
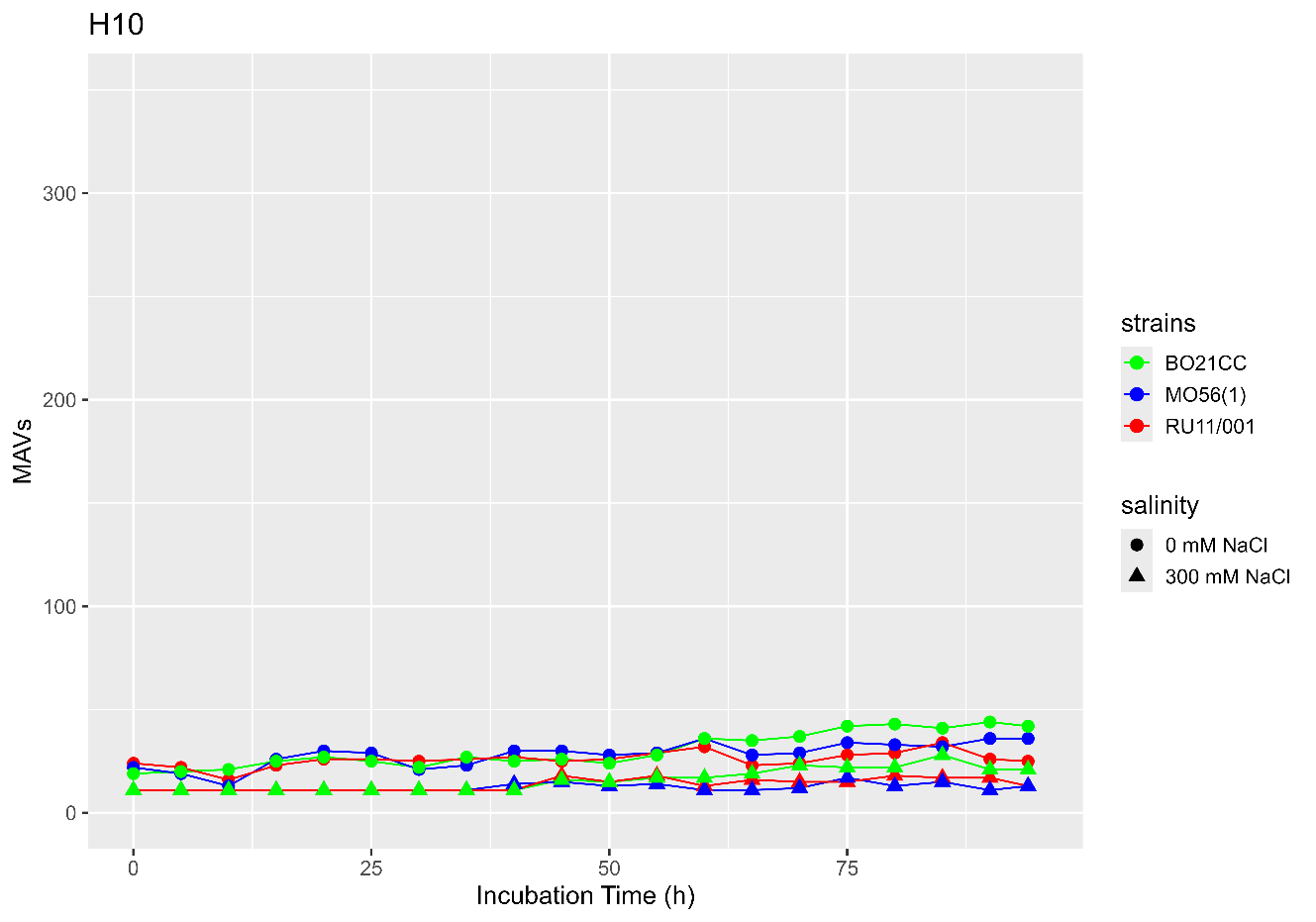
D-Galacturonic acid**

**Phenylethylamine**

**
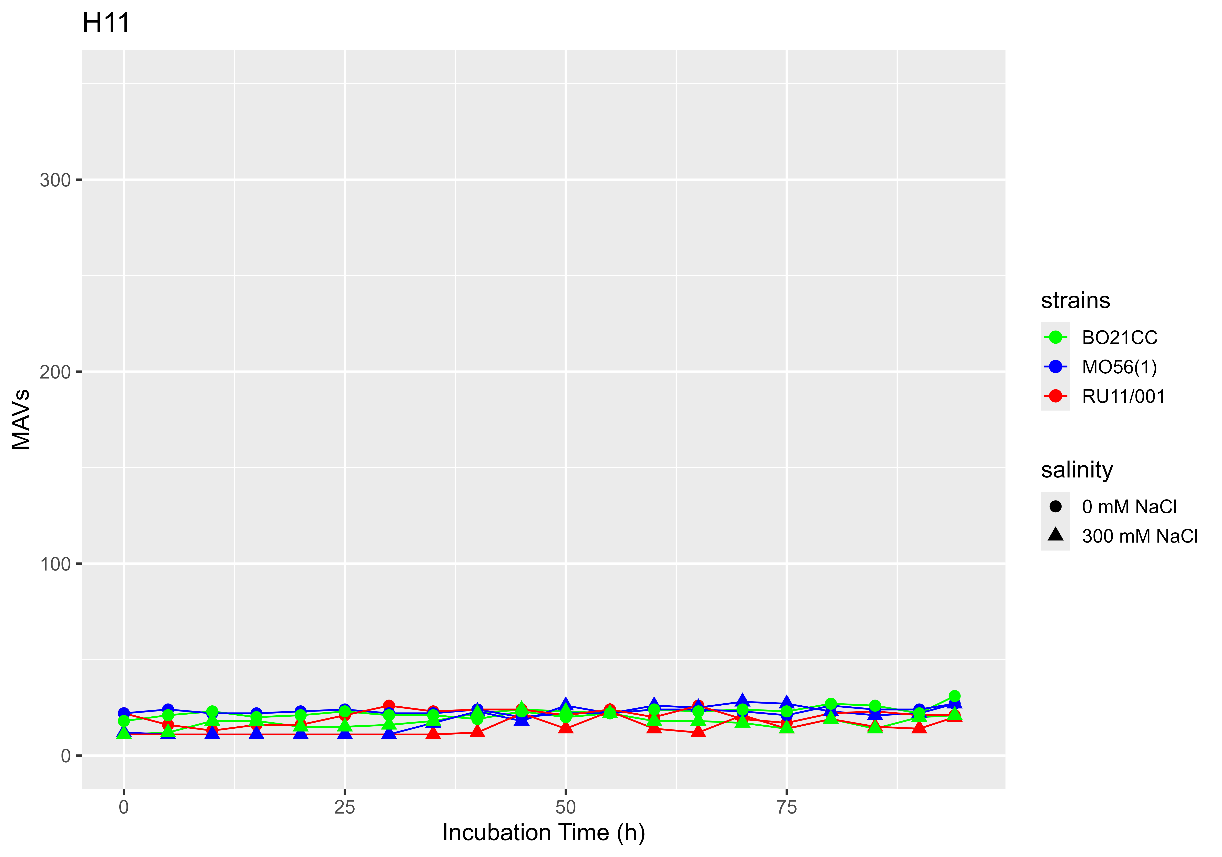
**

**PM2:**

**α-Cyclodextrin**

**
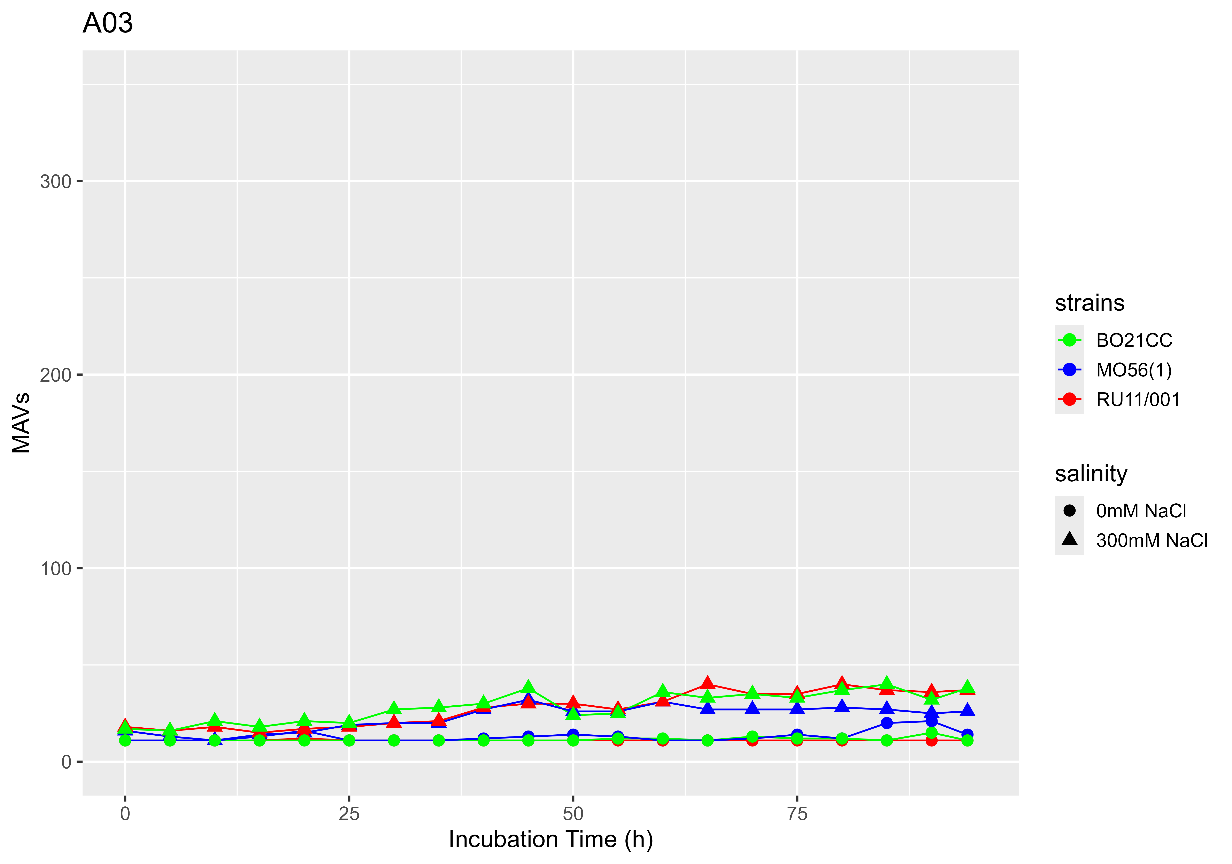
**

**
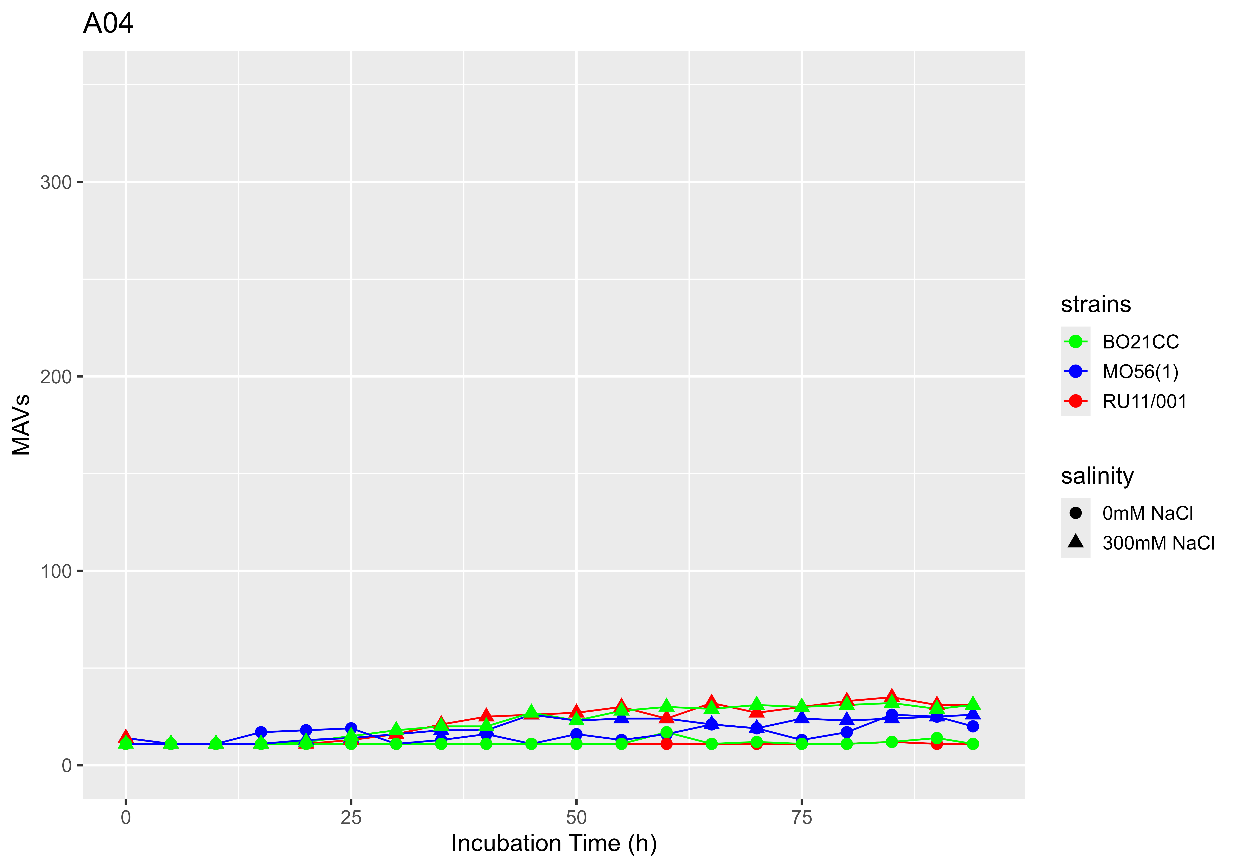
β-Cyclodextrin**

**γ-Cyclodextrin**

**
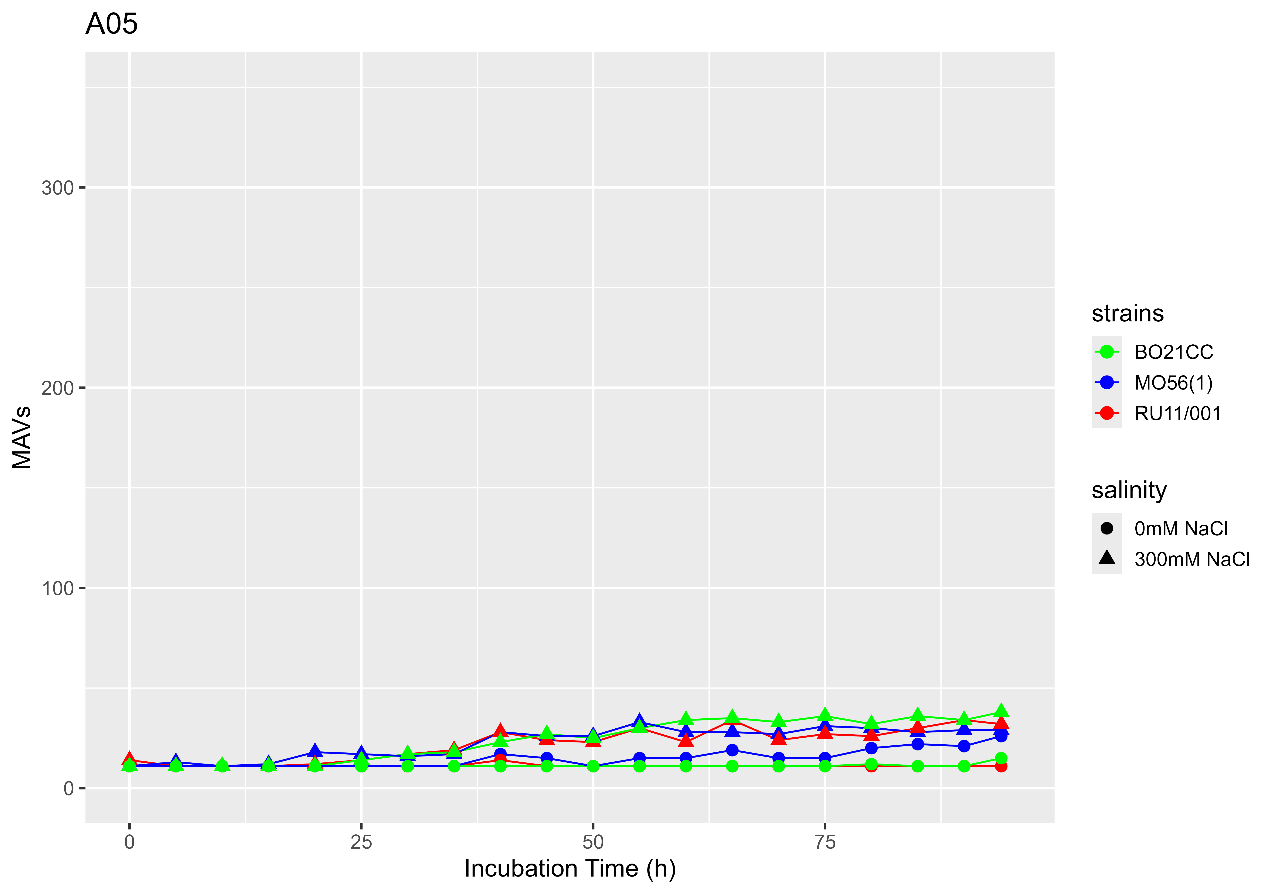
**

**
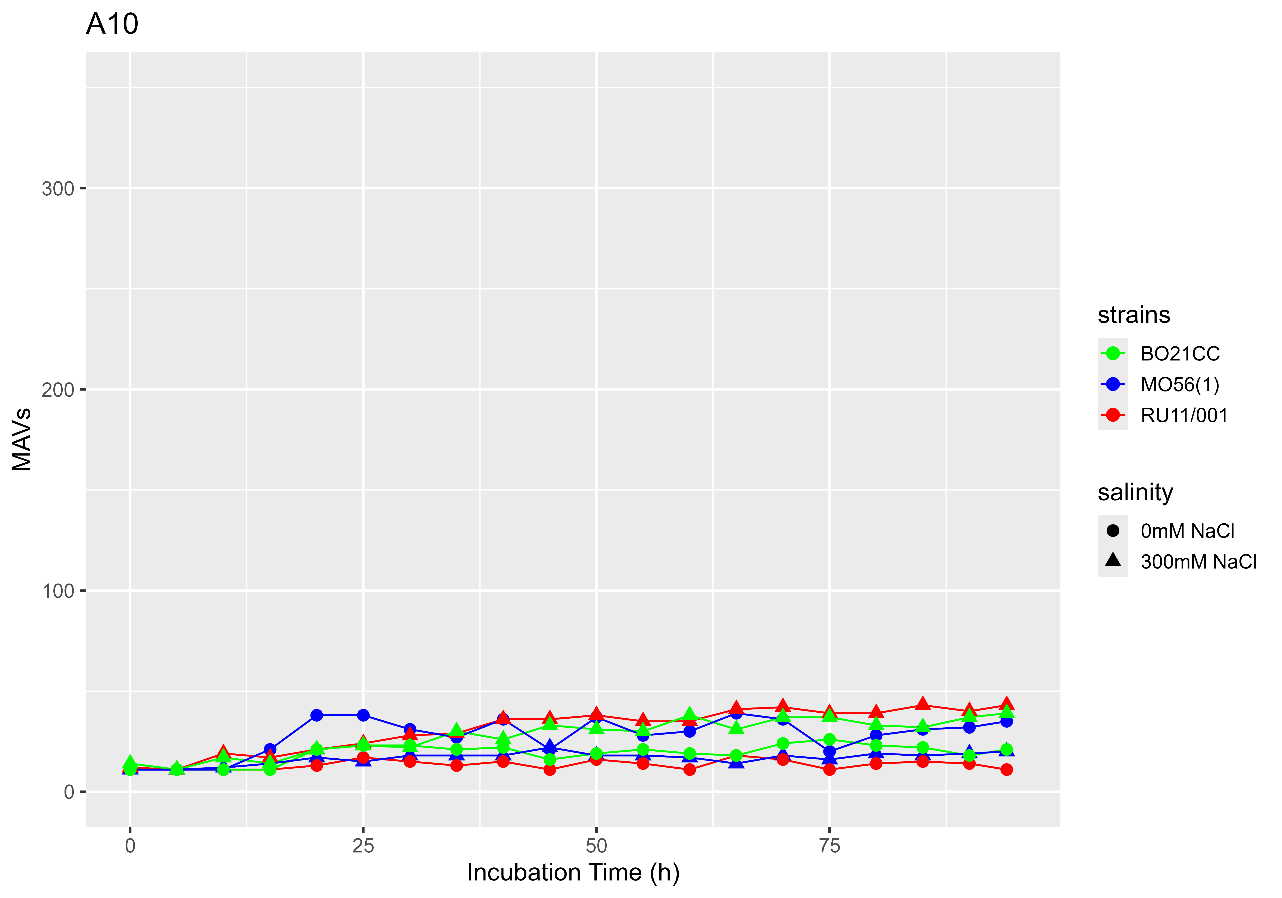
Laminarin**

**
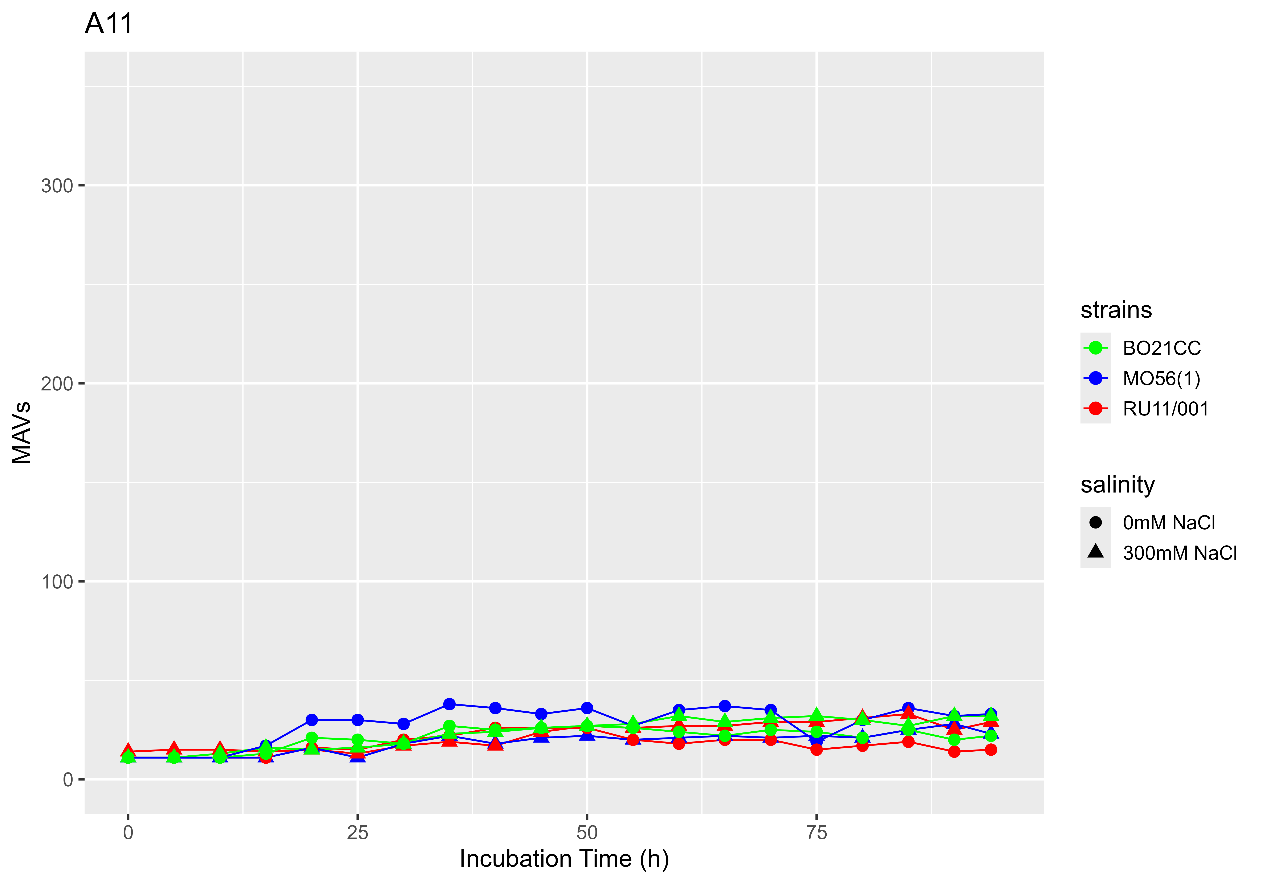
Mannan**

**N-Acetyl-Neuraminic acid**

**
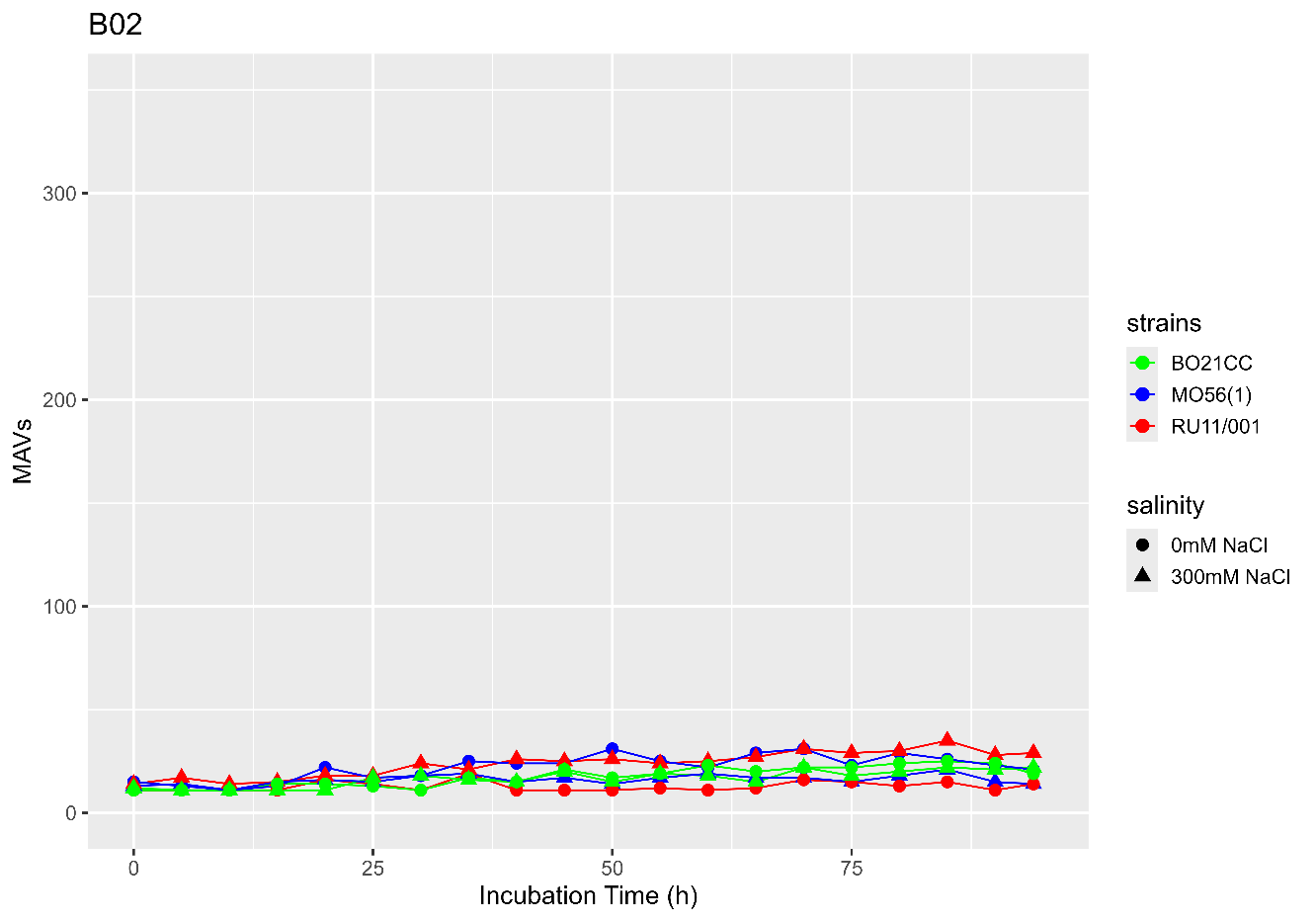
**

**Amygdalin**

**
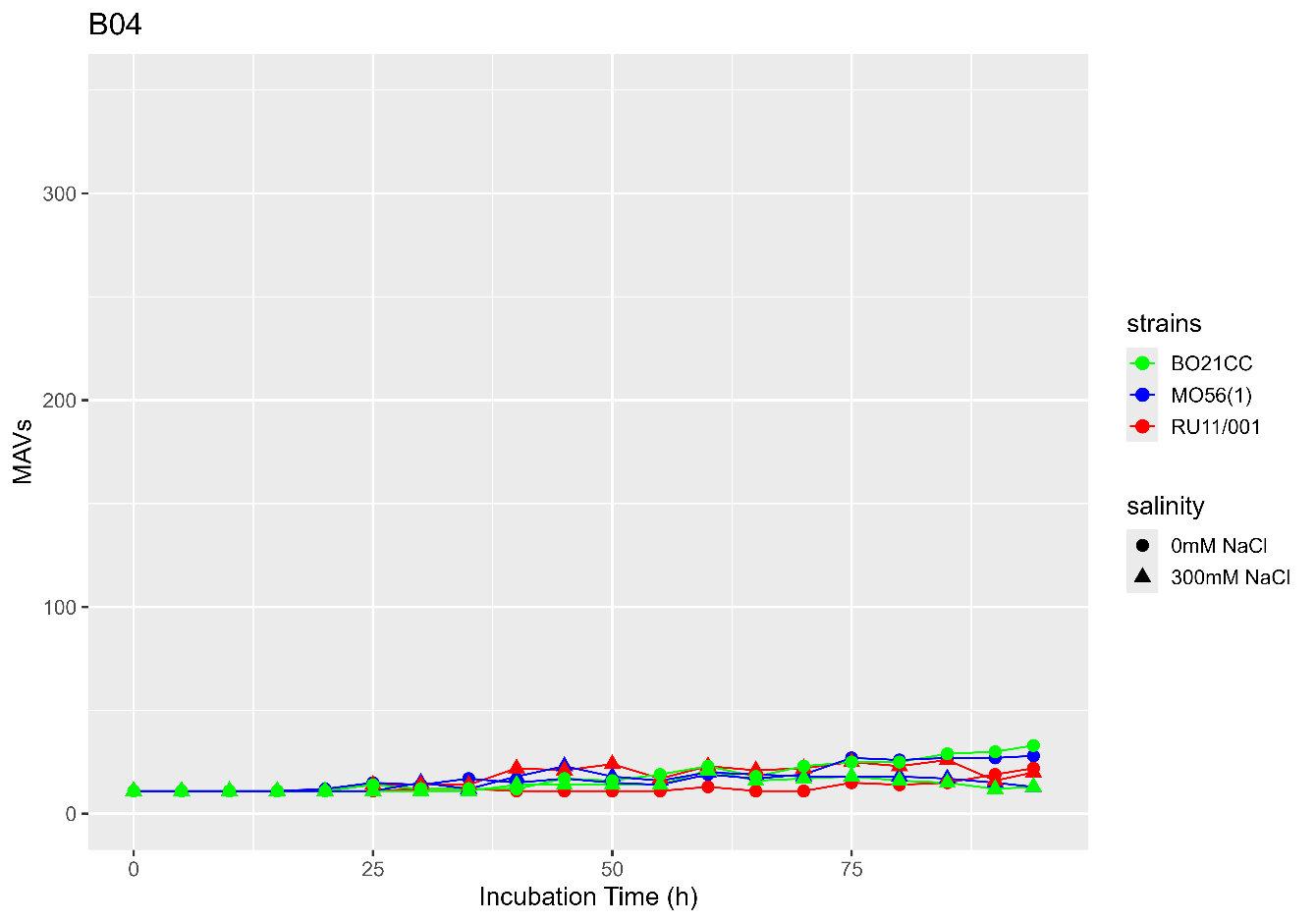
**

**
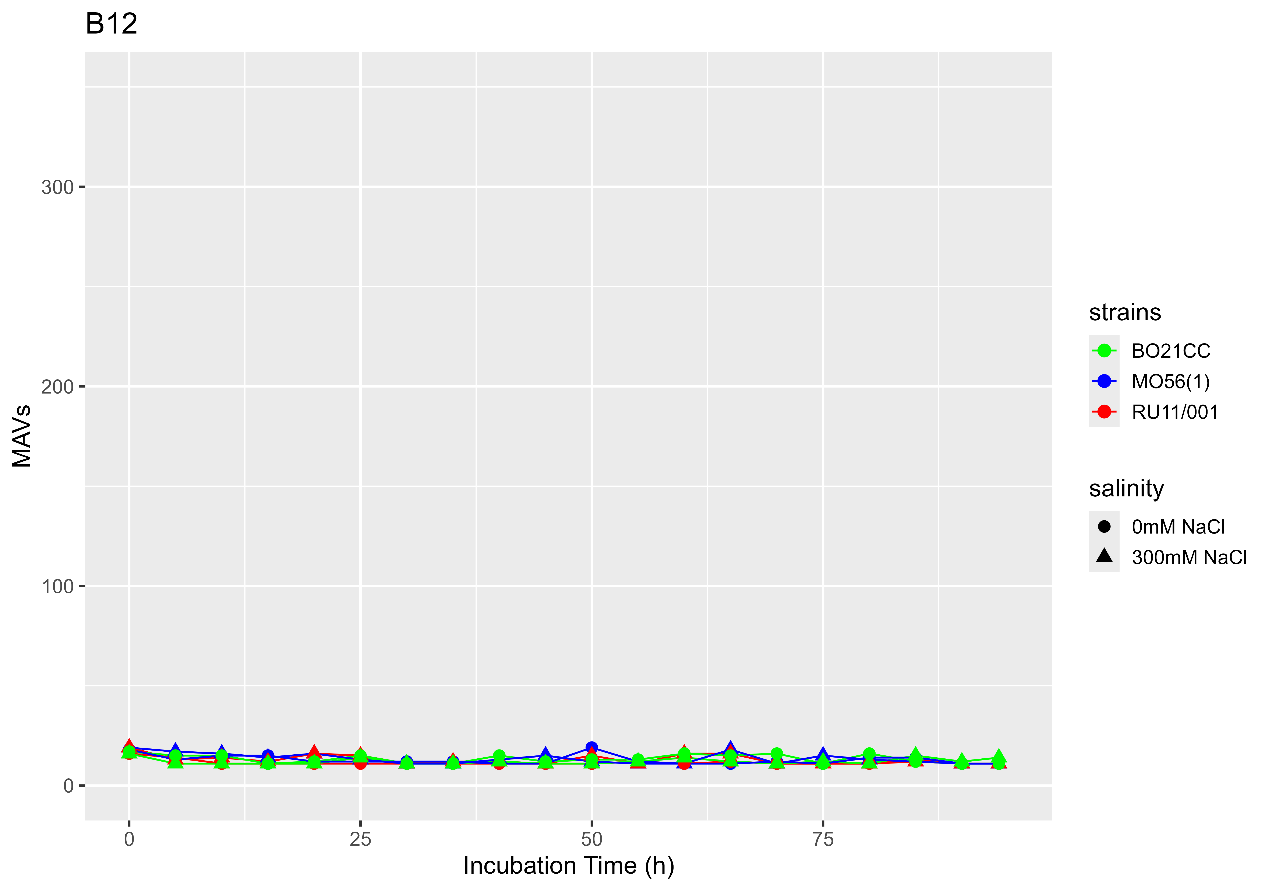
3-0-β-D-Galactopyranosyl-D-Arabinose**

**
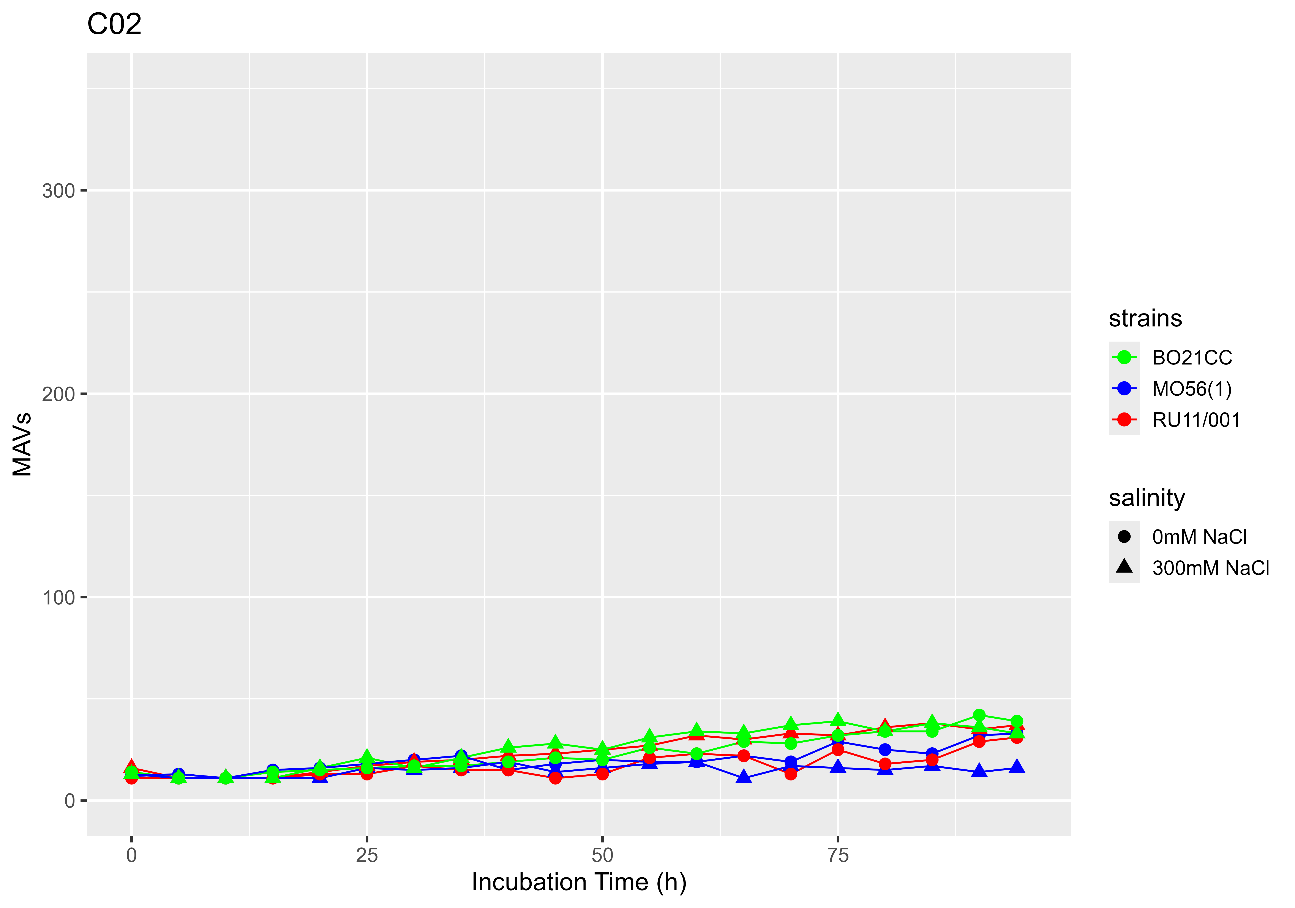
L-Glucose**

**
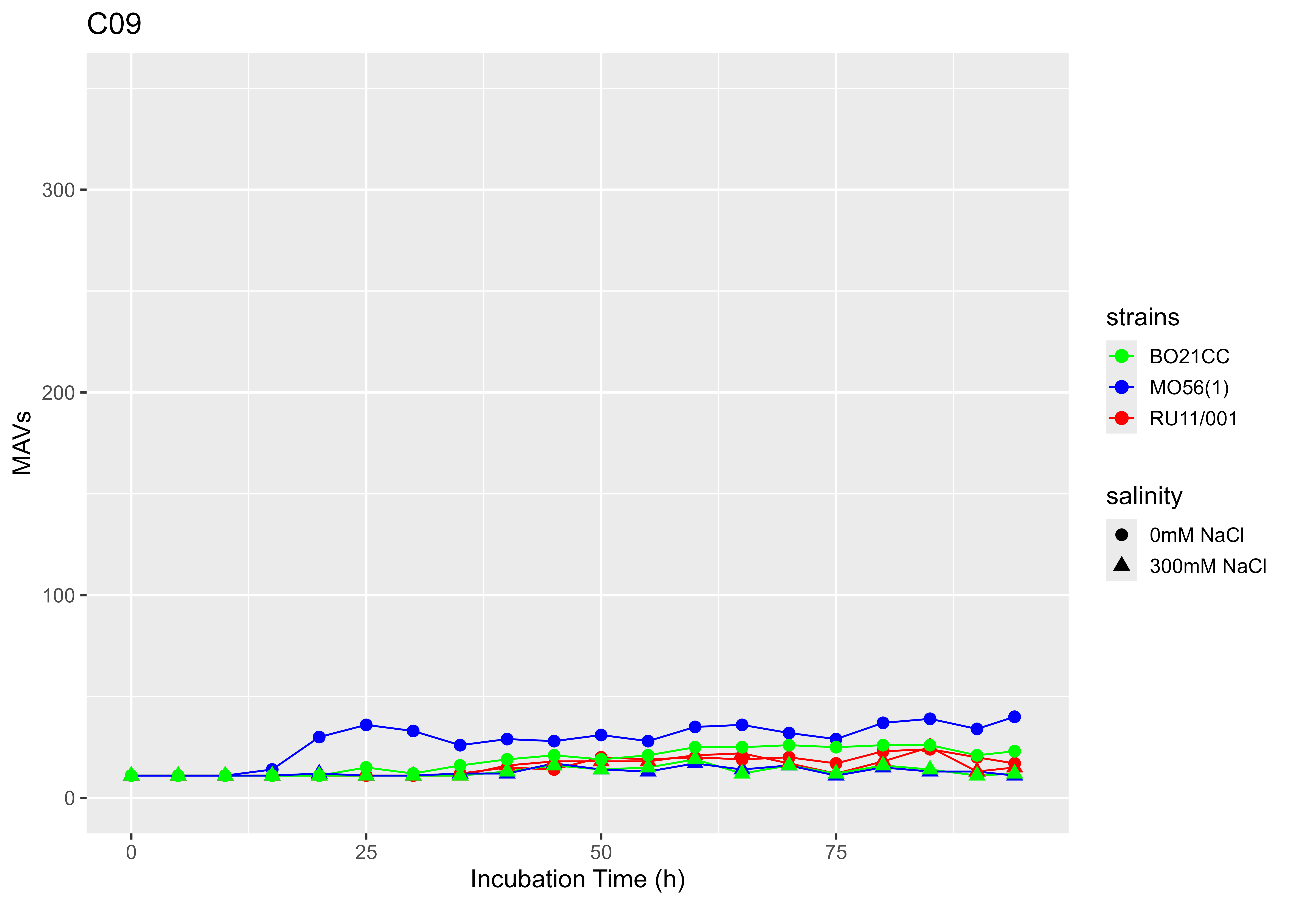
β-Methyl-D-Glucuronic acid**

**
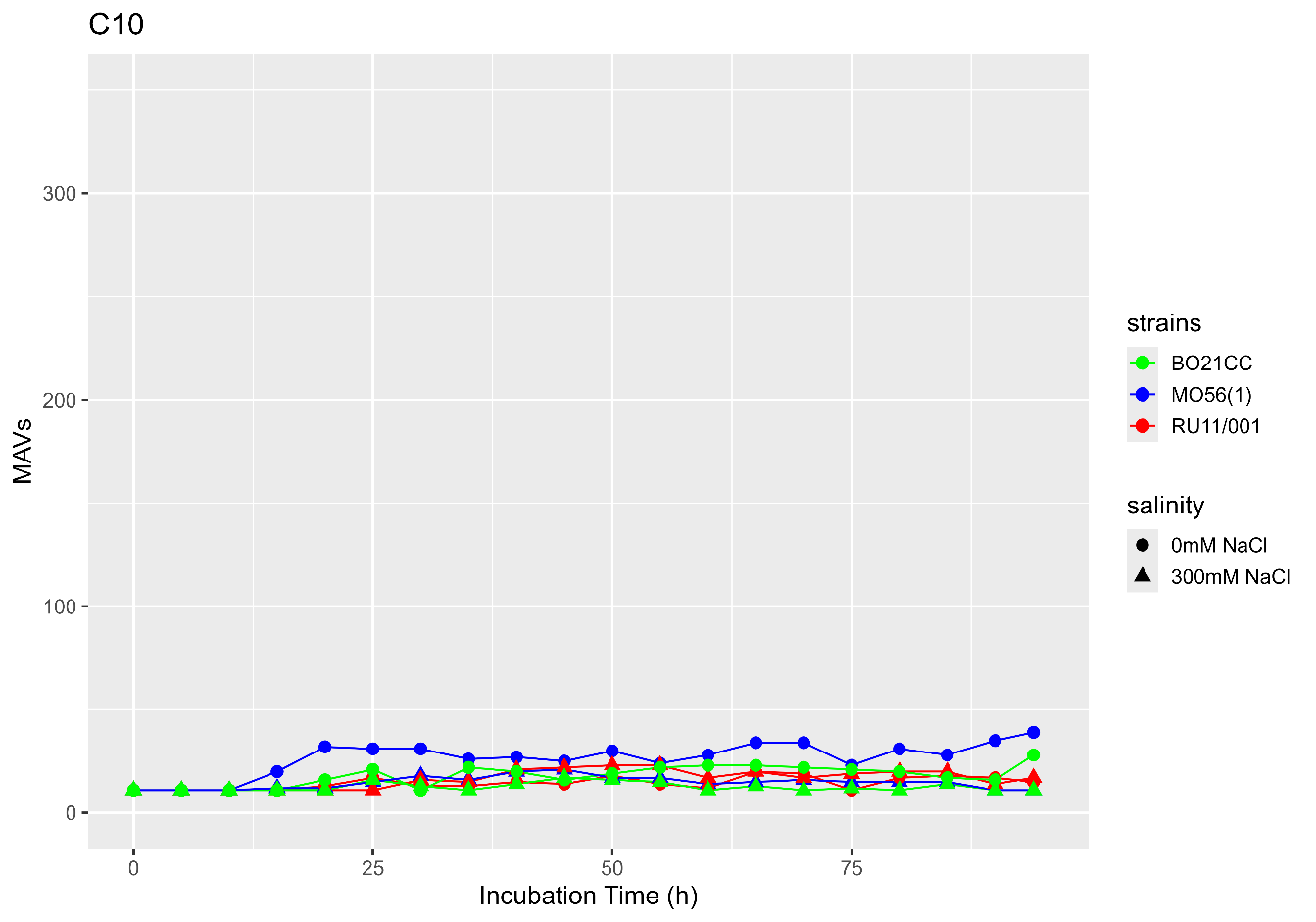
α-Methyl-D-Mannoside**

**
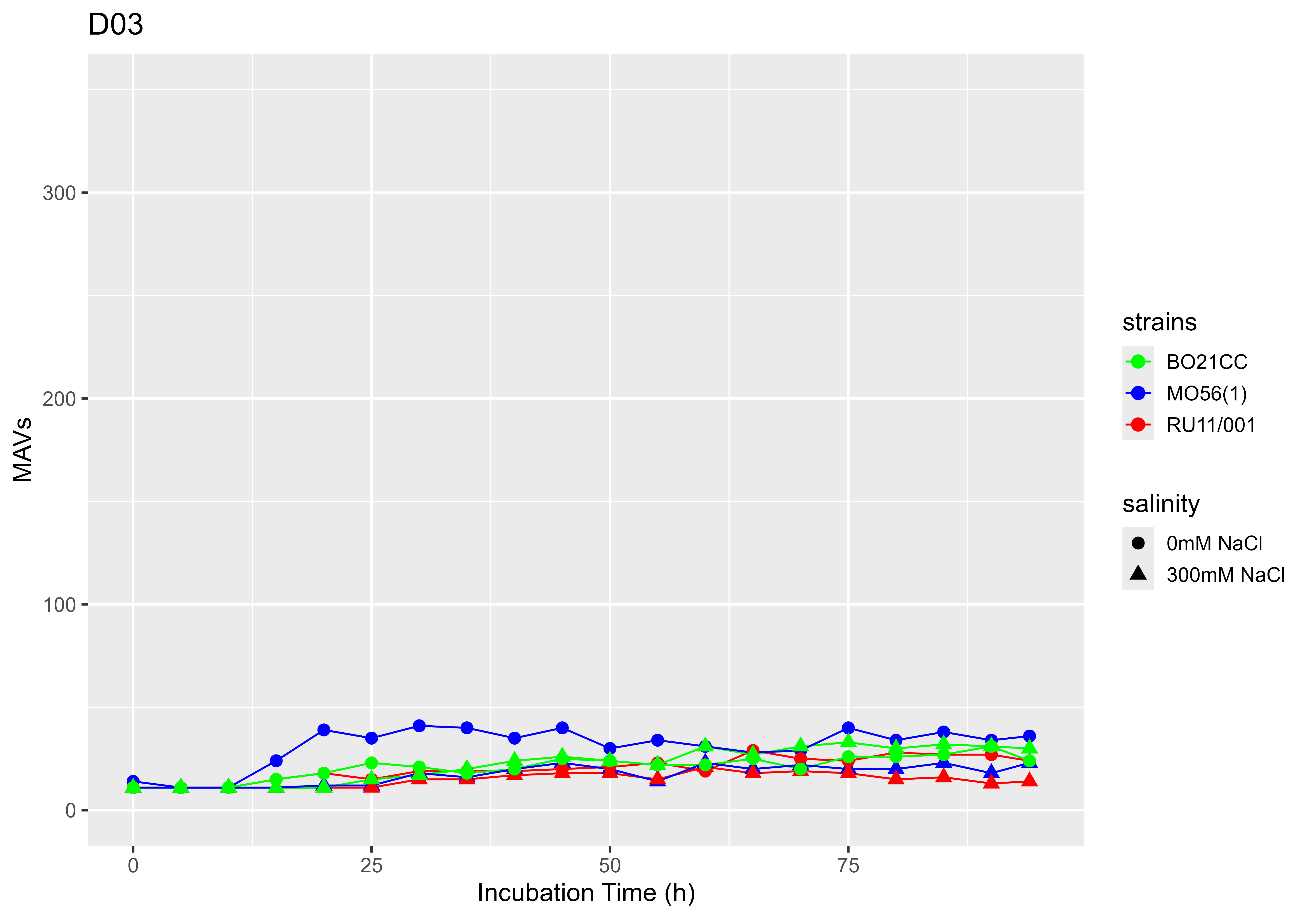
Sedoheptulosan**

**
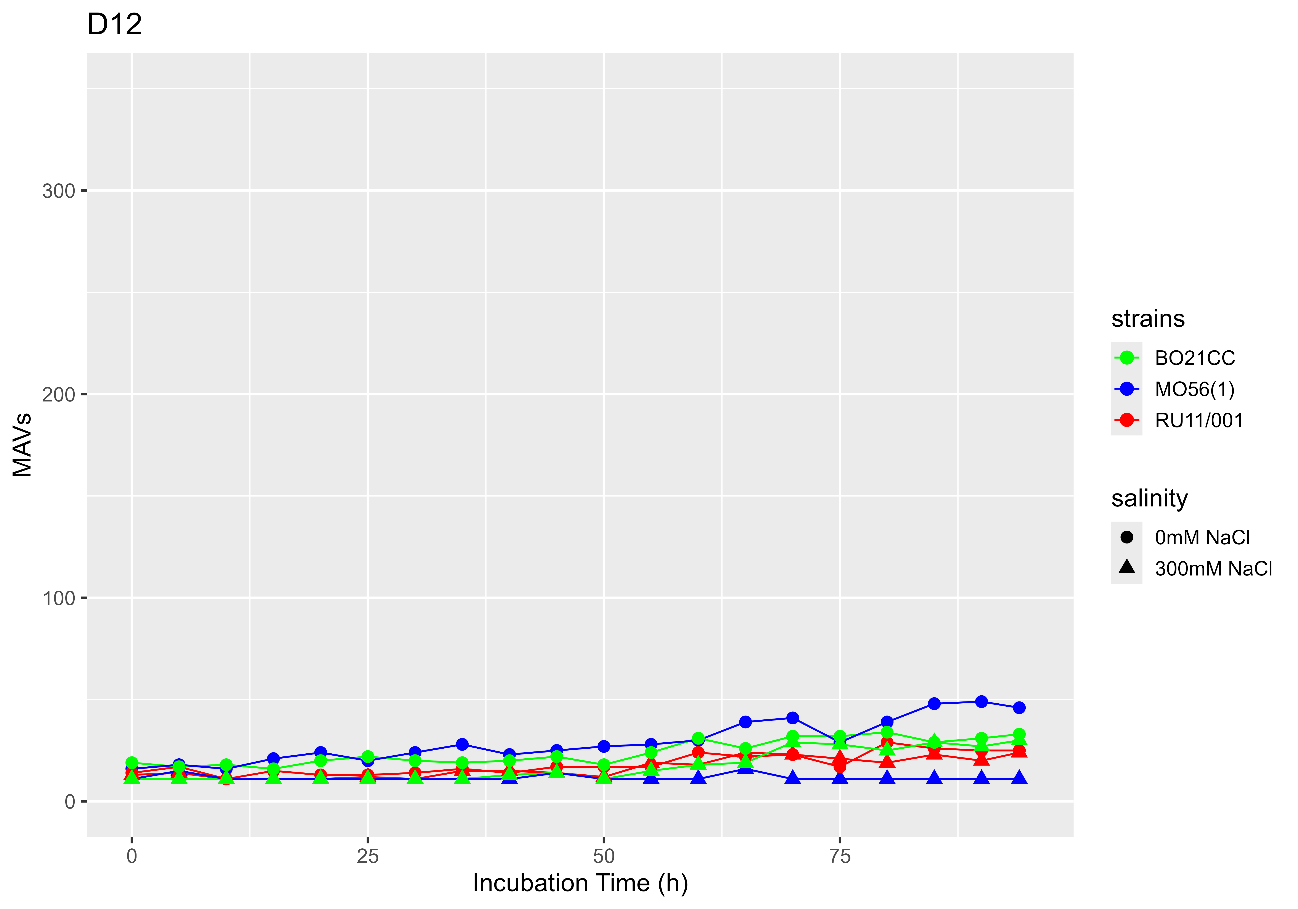
Butyric acid**

**
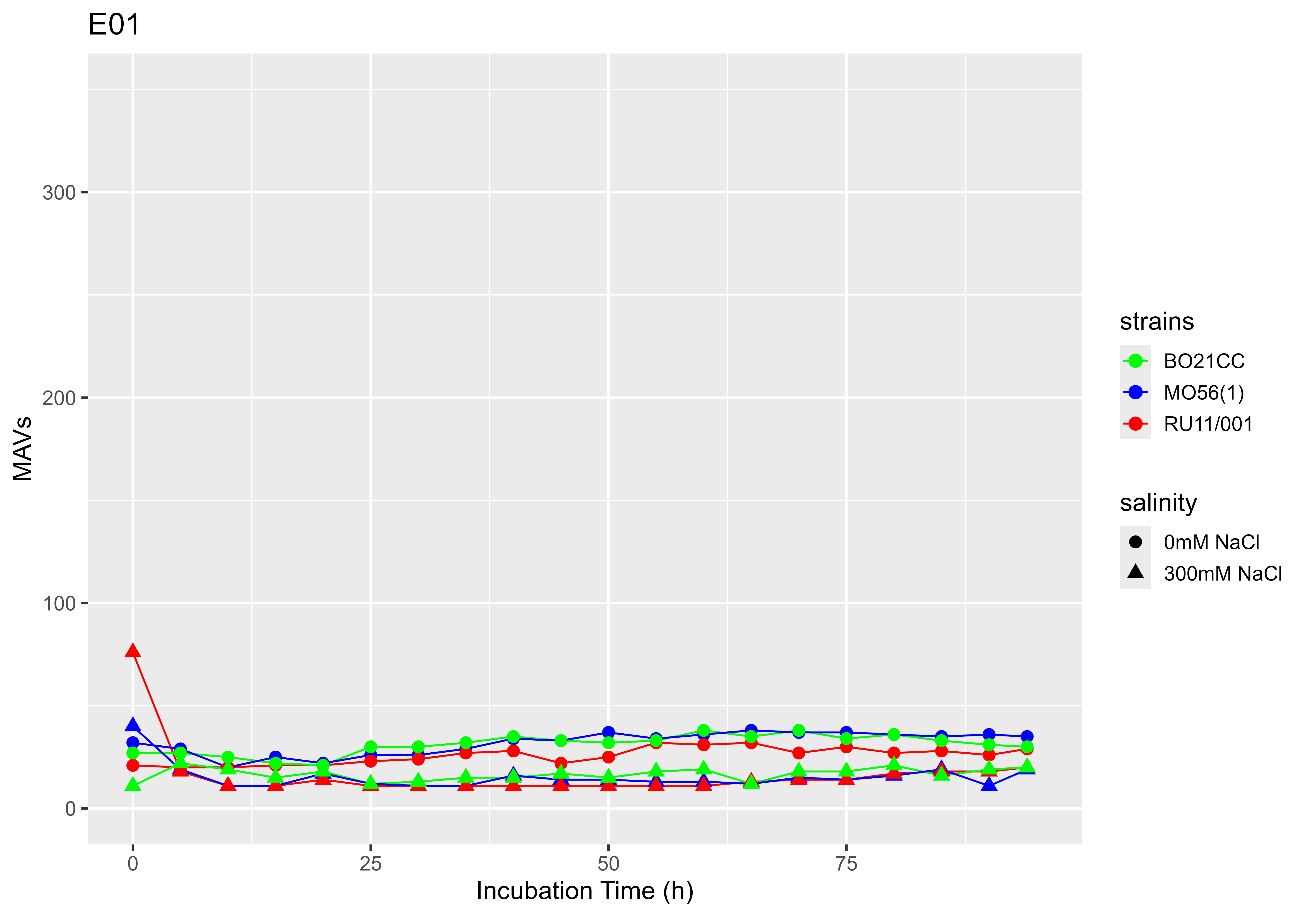
Capric acid**

**
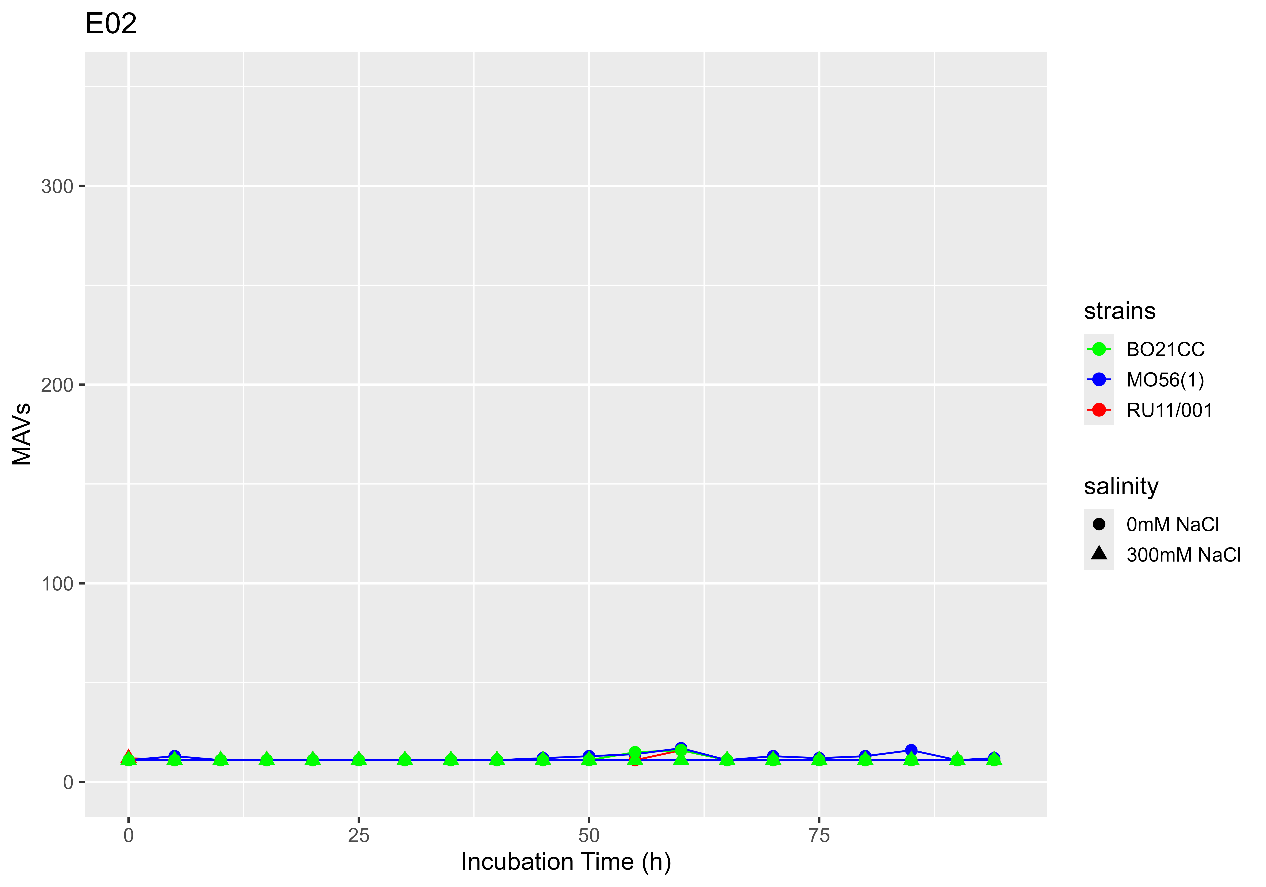
Caproic acid**

**
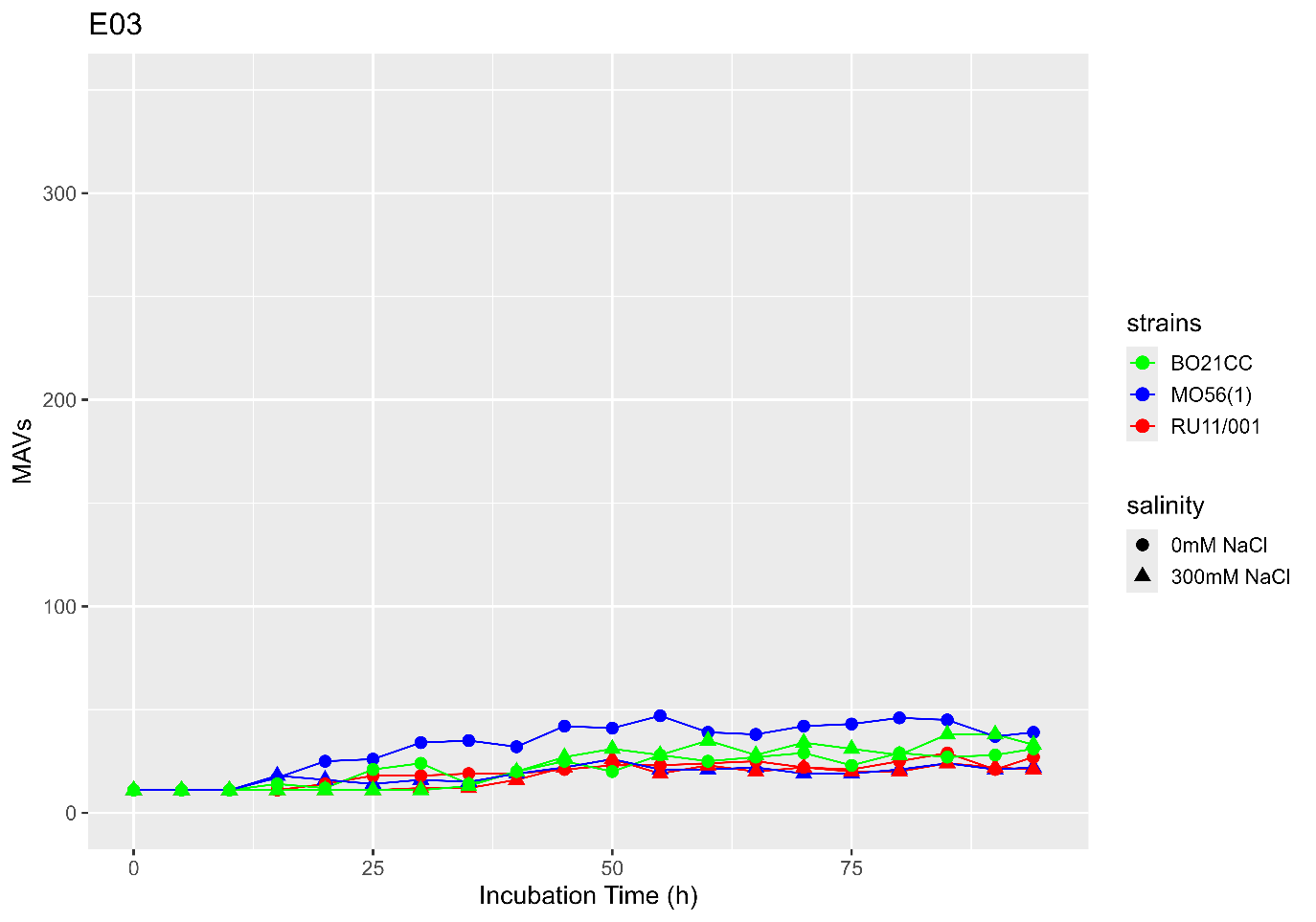
Citraconic acid**

**
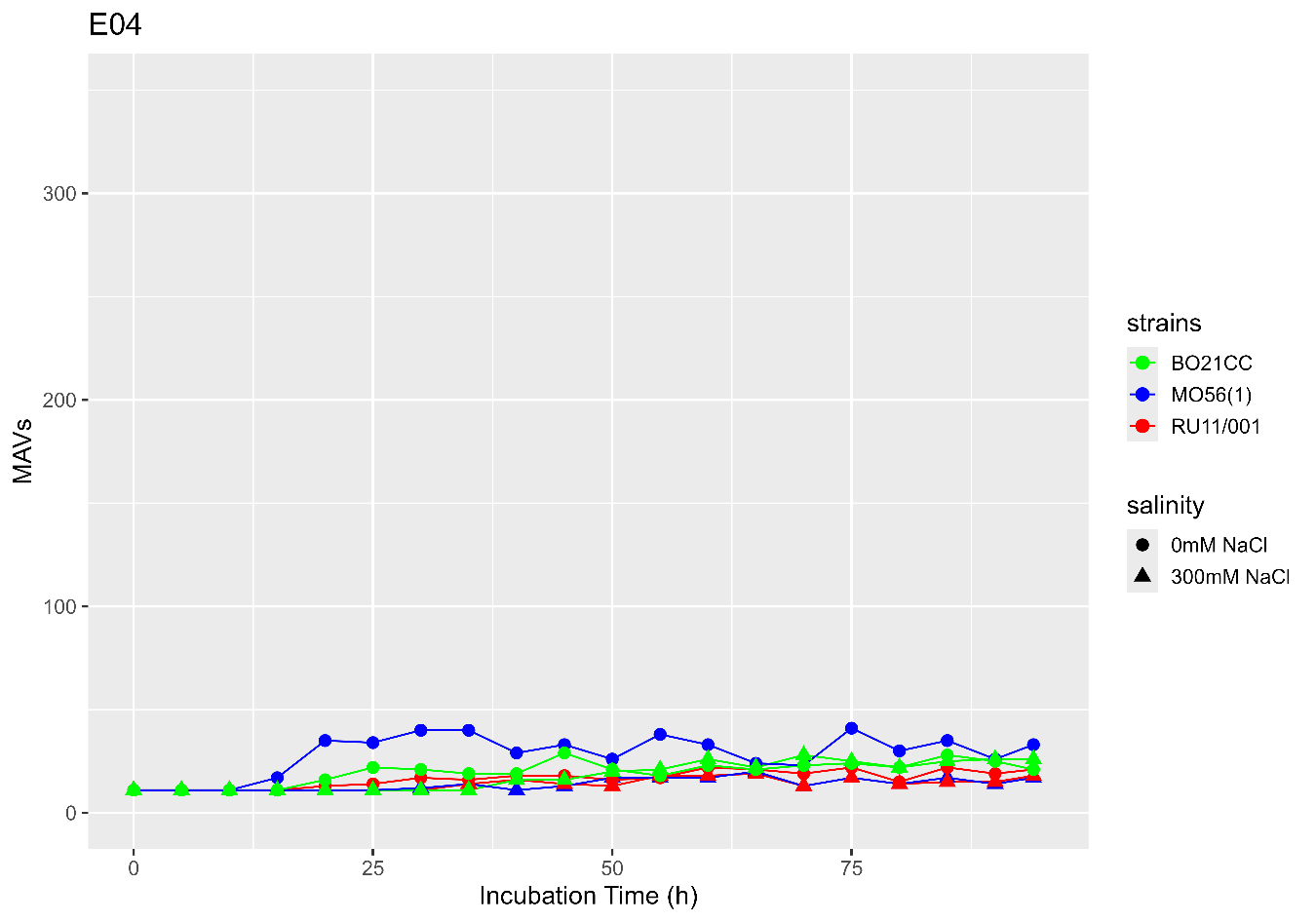
D,L-Citramalic acid**

**
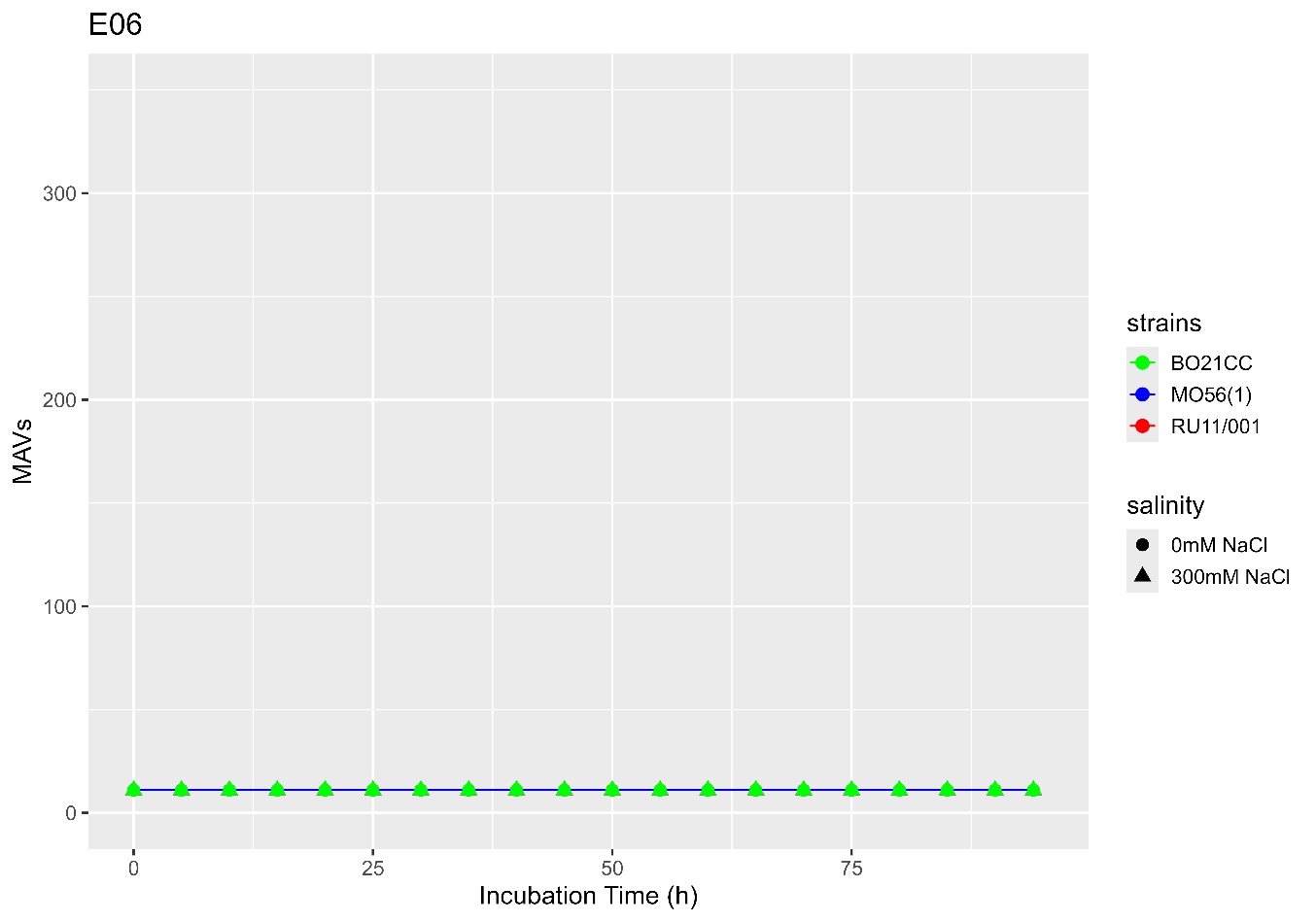
2-Hydroxybenzoic acid**

**4-Hydroxybenzoic acid**

**
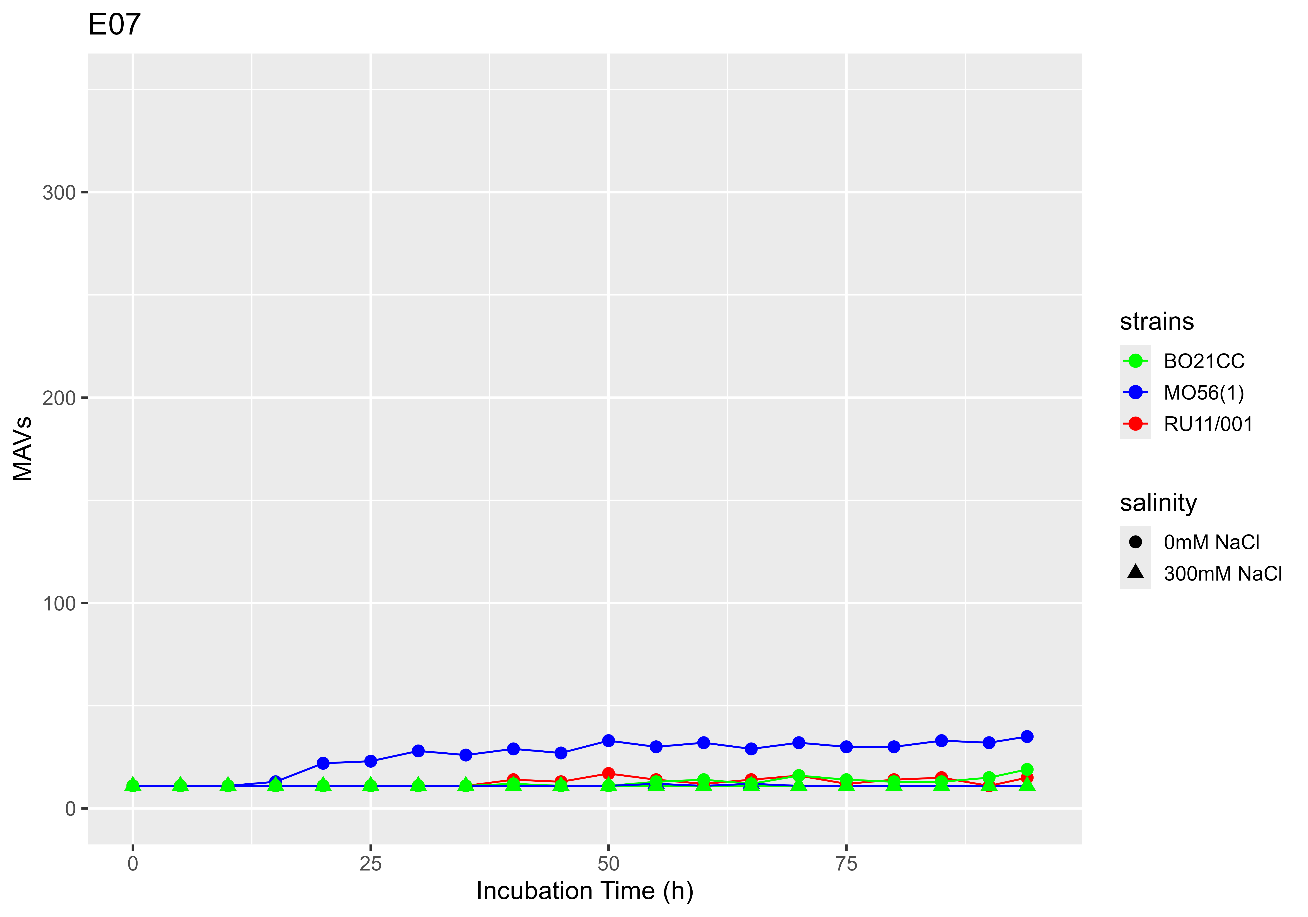
**

**
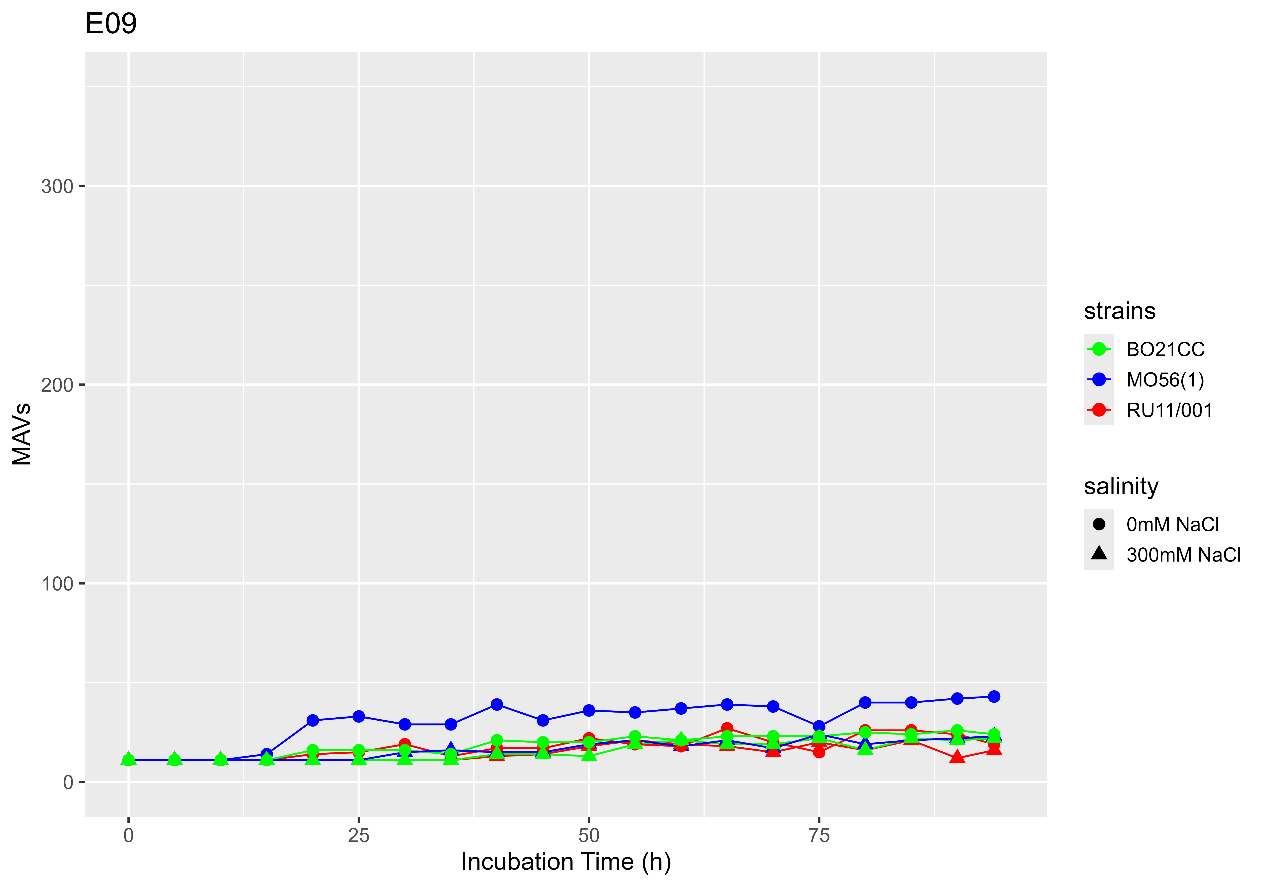
γ-Hydroxybutyric acid**

**β-Hydroxypyruvic acid**

**
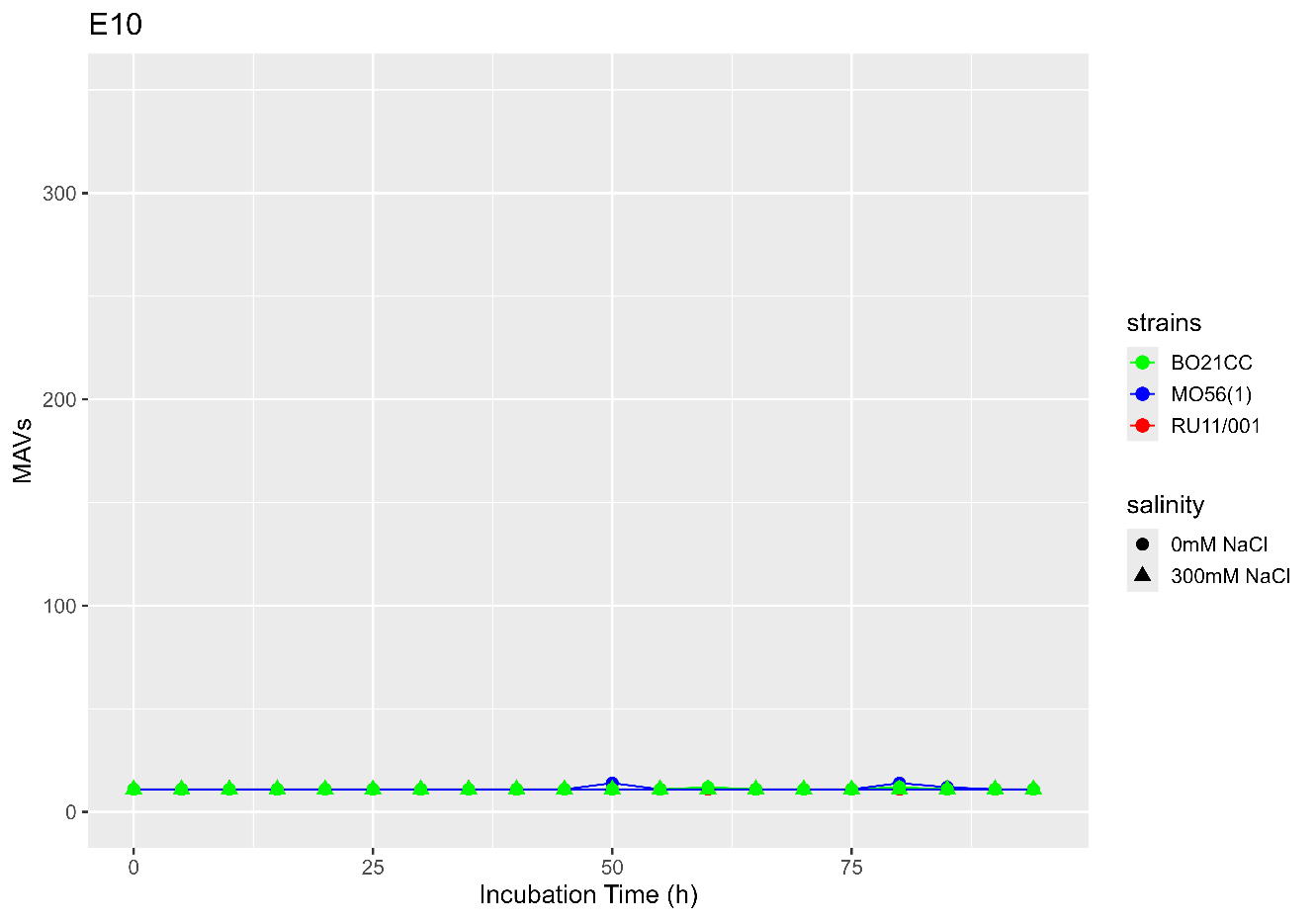
**

**
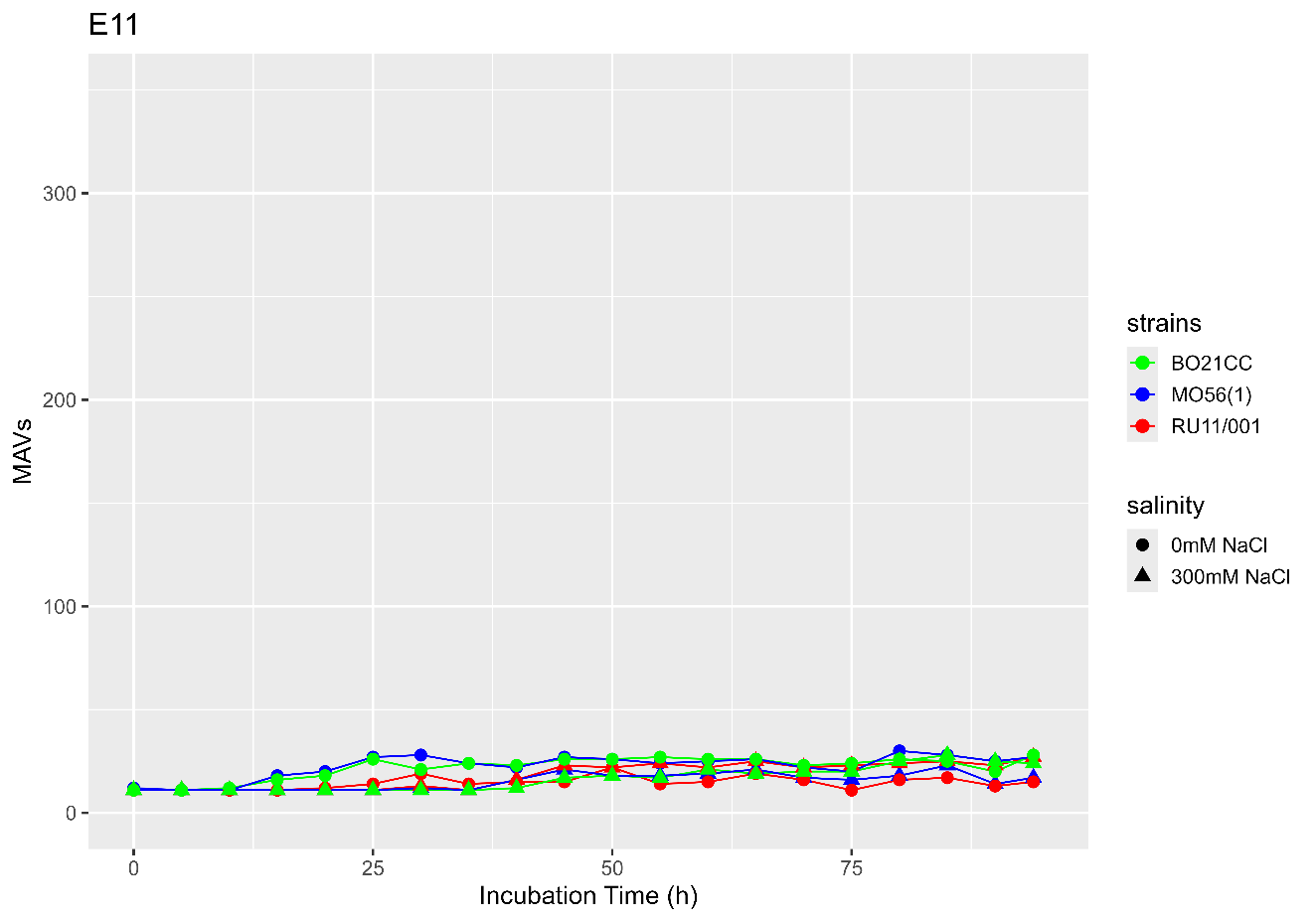
Itaconic acid**

**Malonic acid**

**
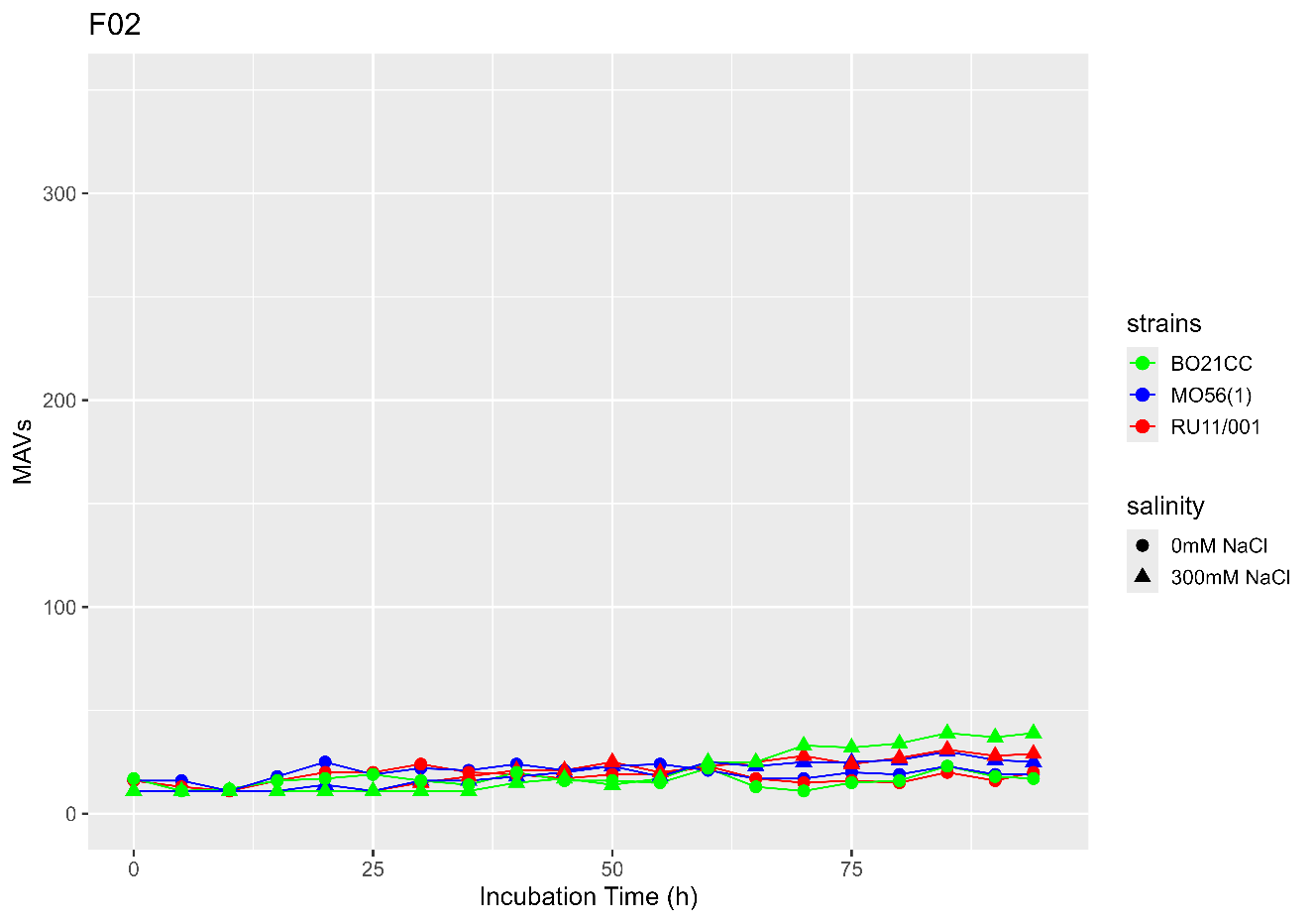
**

**
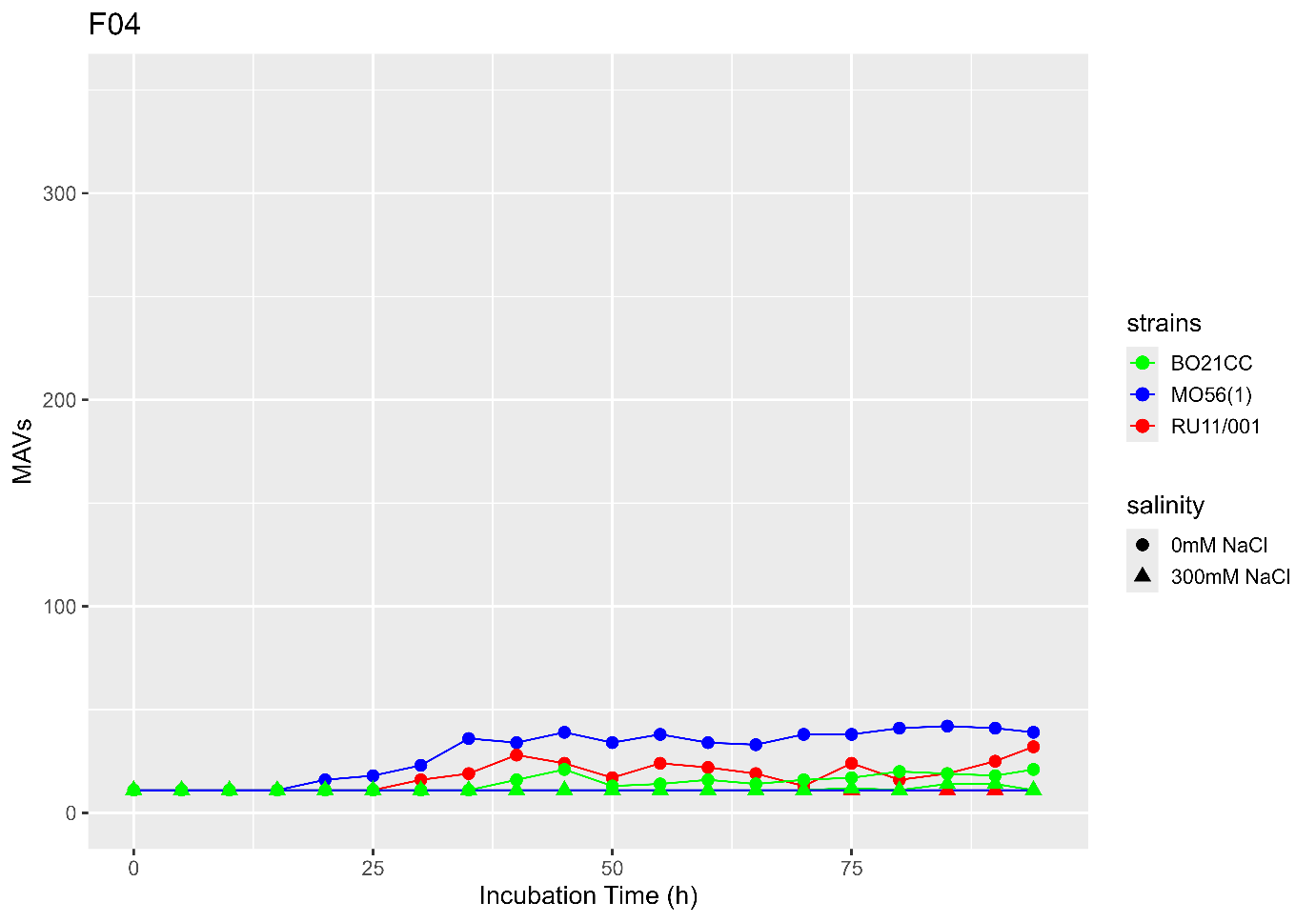
Oxalic acid**

**
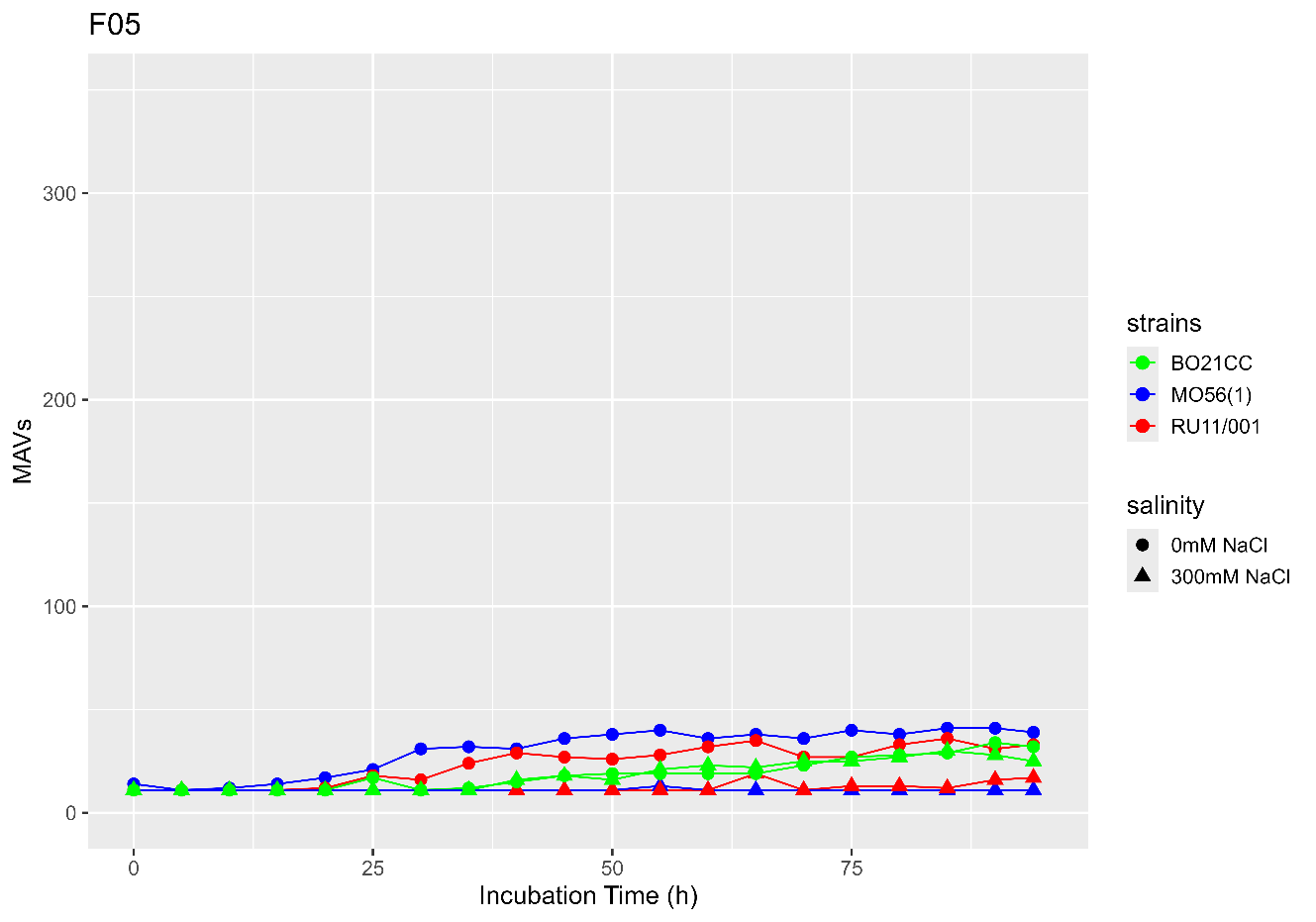
Oxalomalic acid**

**
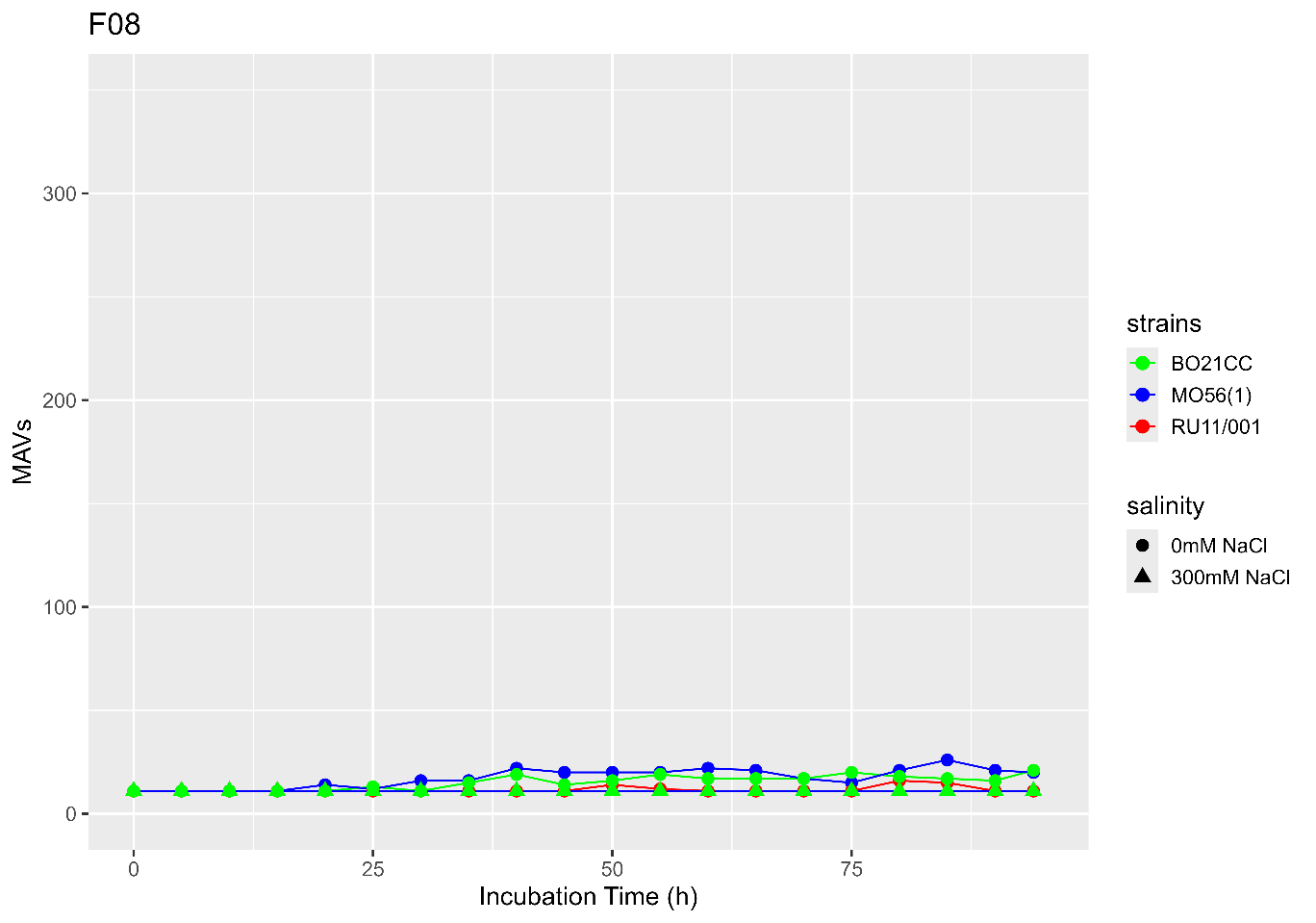
Sebacic acid**

**
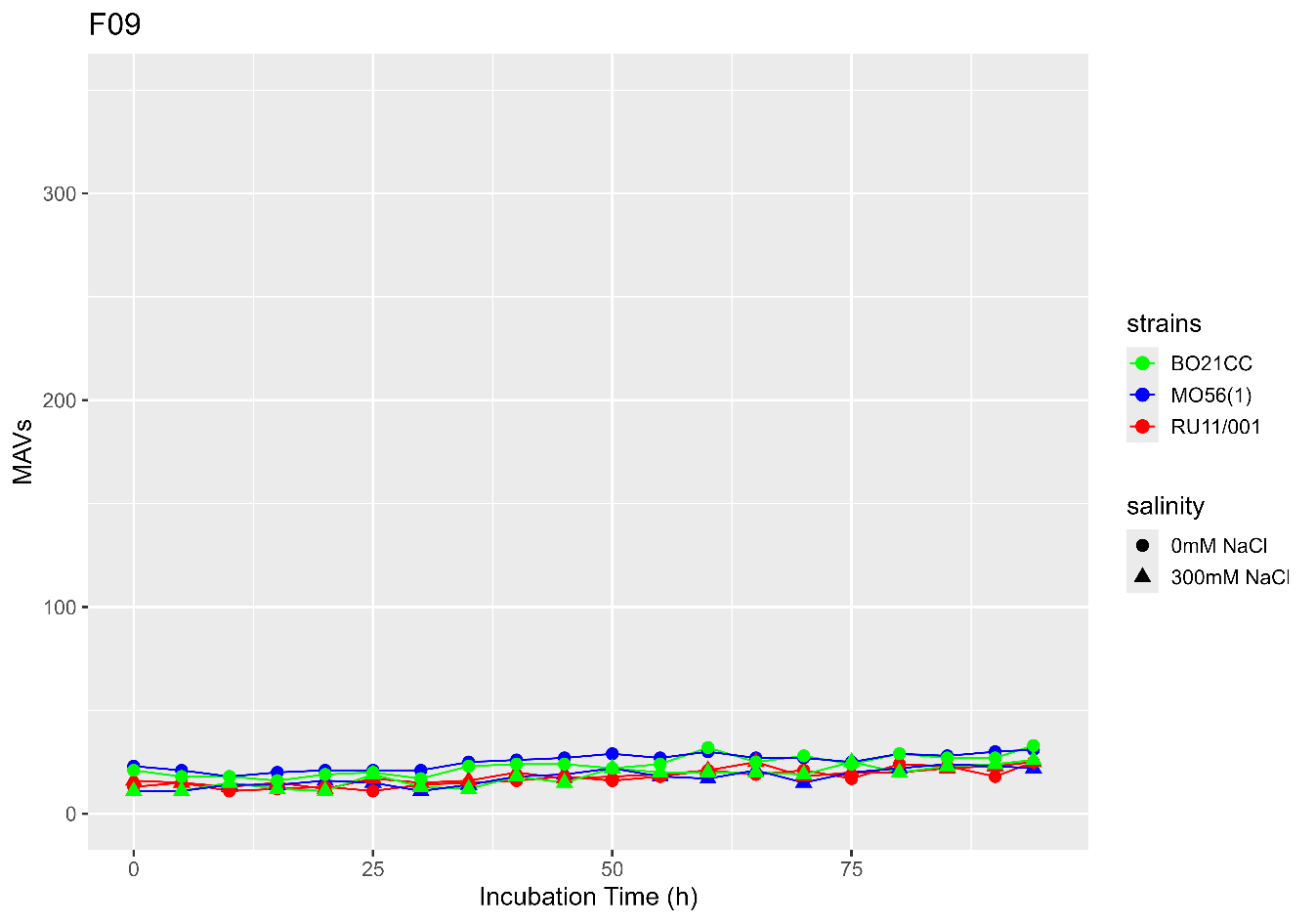
Sorbic acid**

**
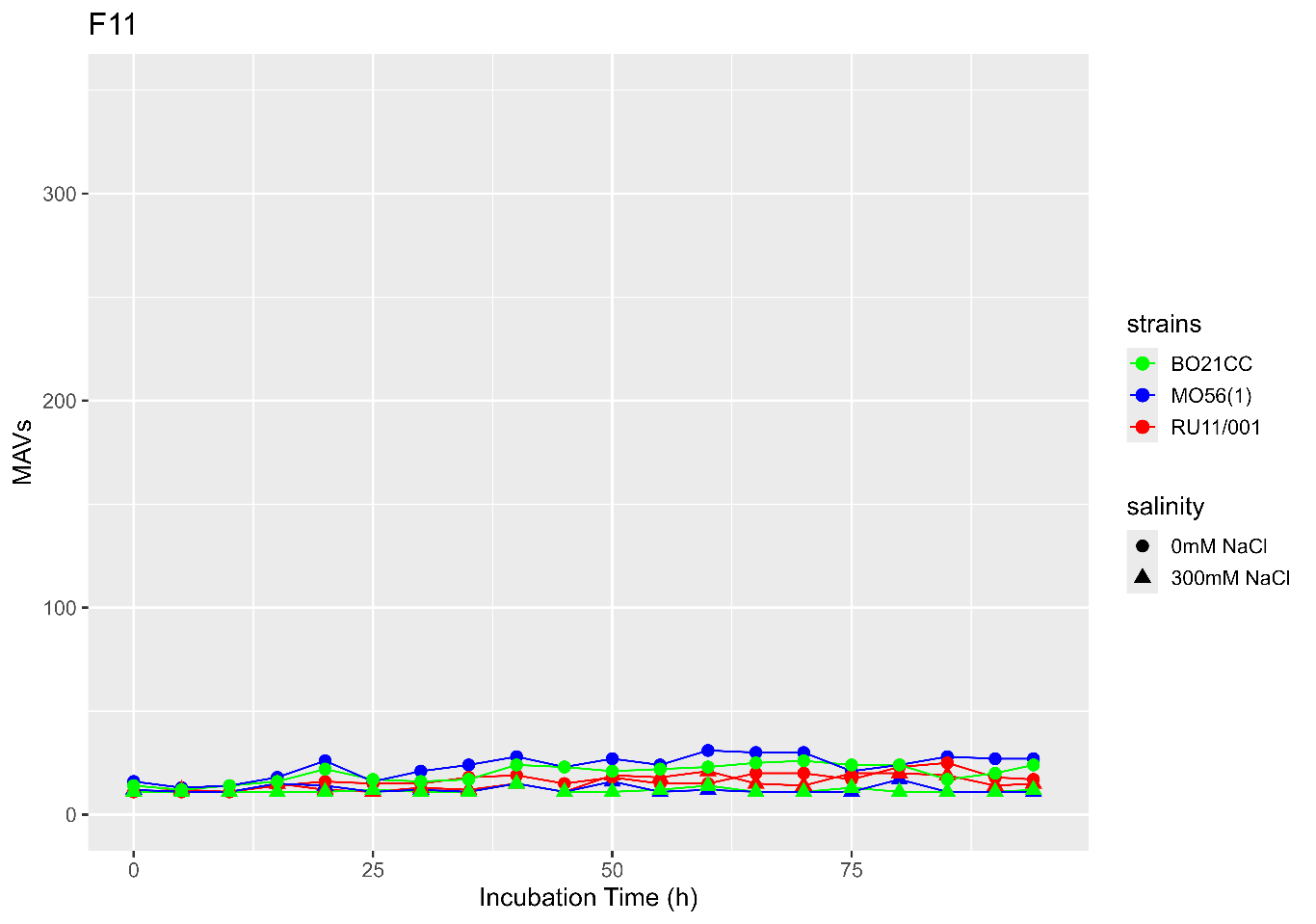
D-Tartaric acid**

**L-Tartaric acid**

**
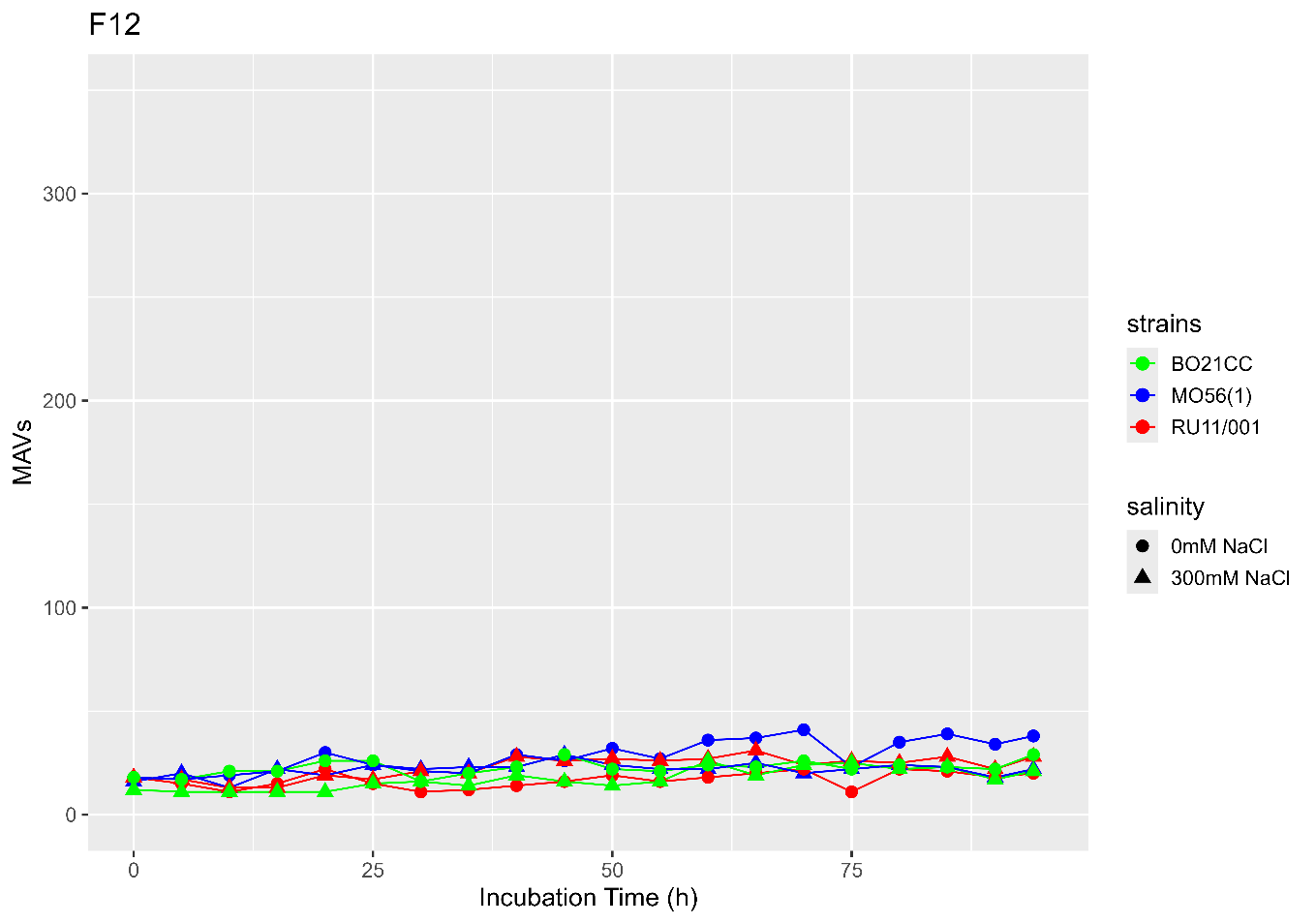
**

**
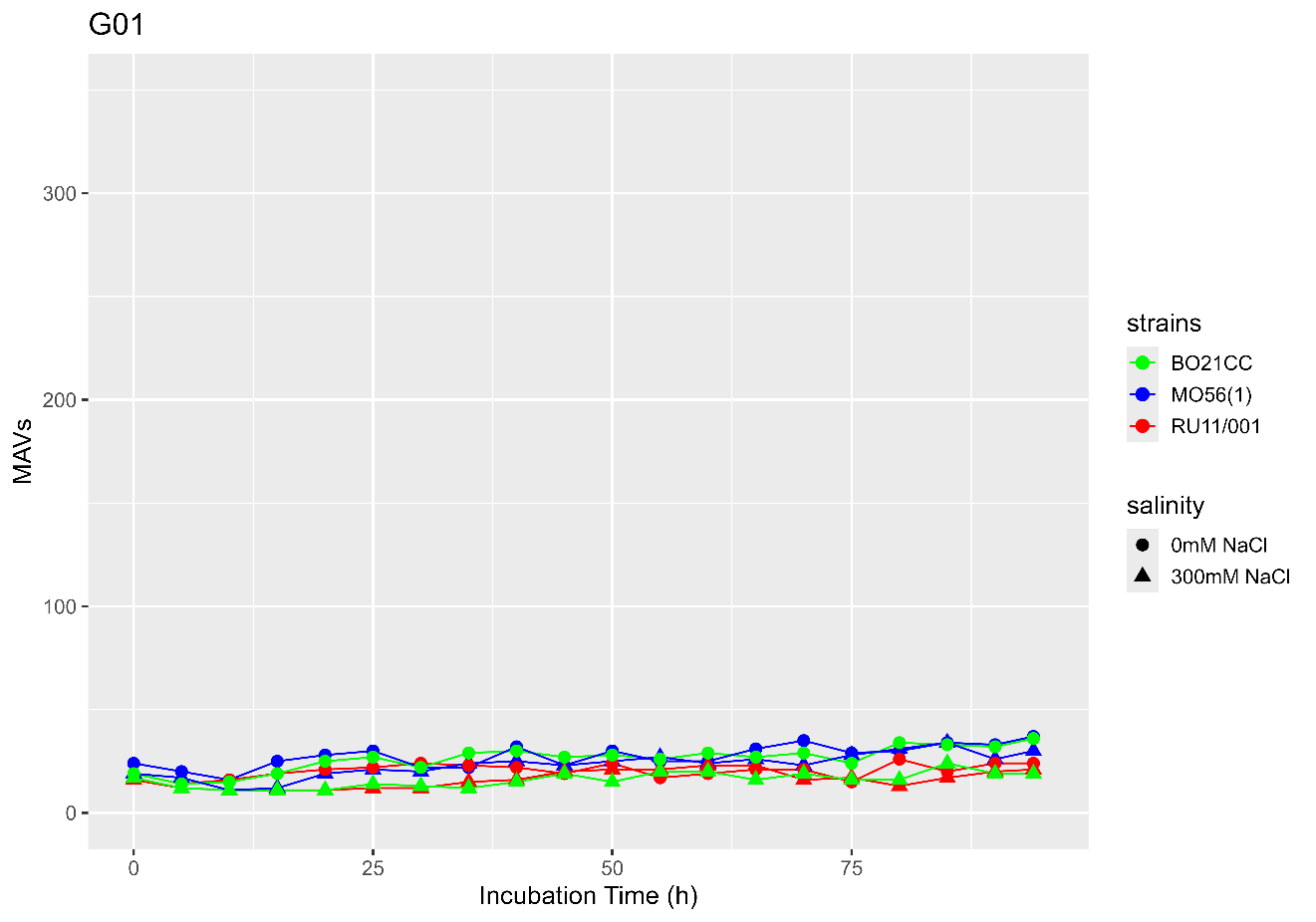
Acetamide**

**L-Alaninamide**

**
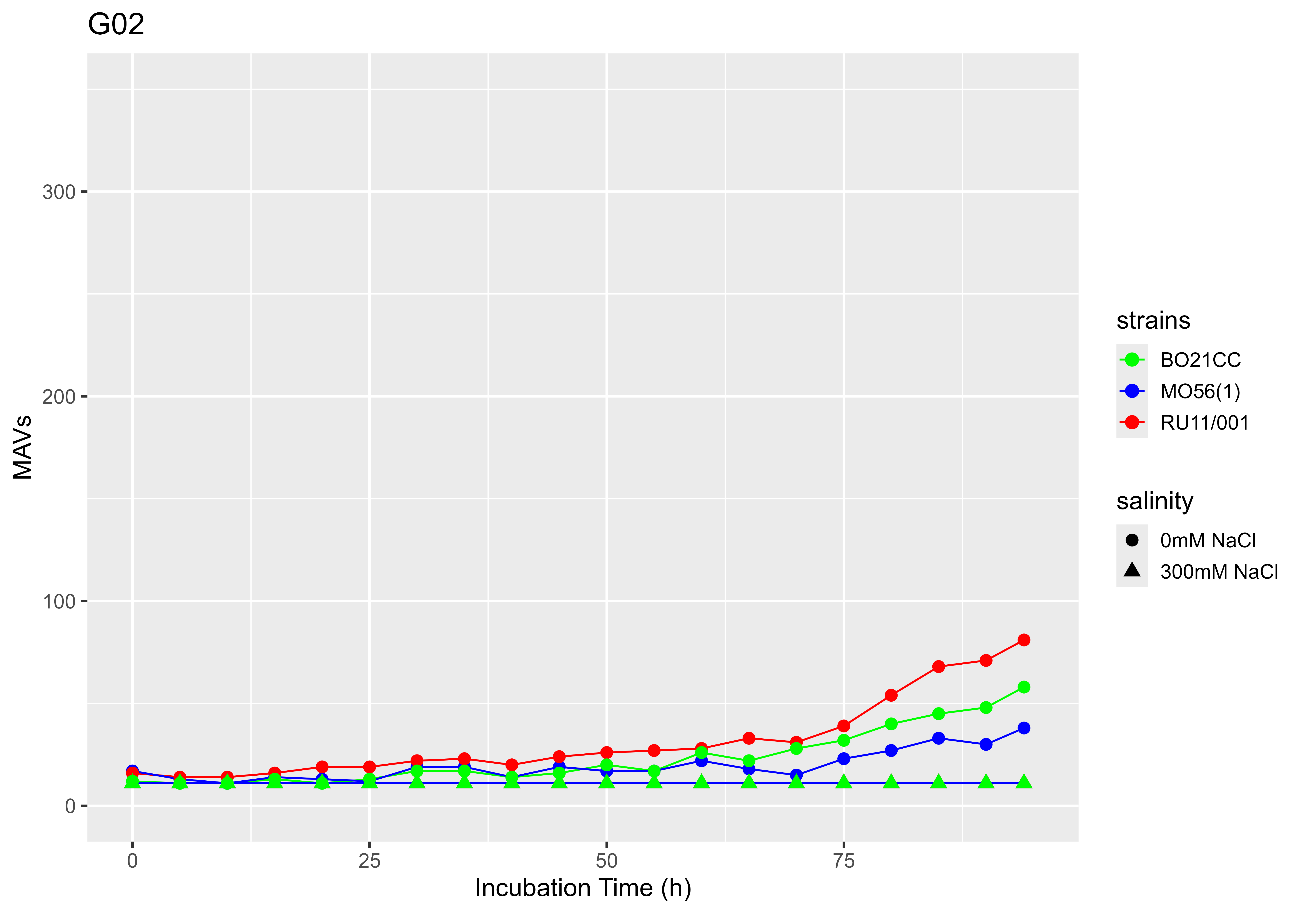
**

**
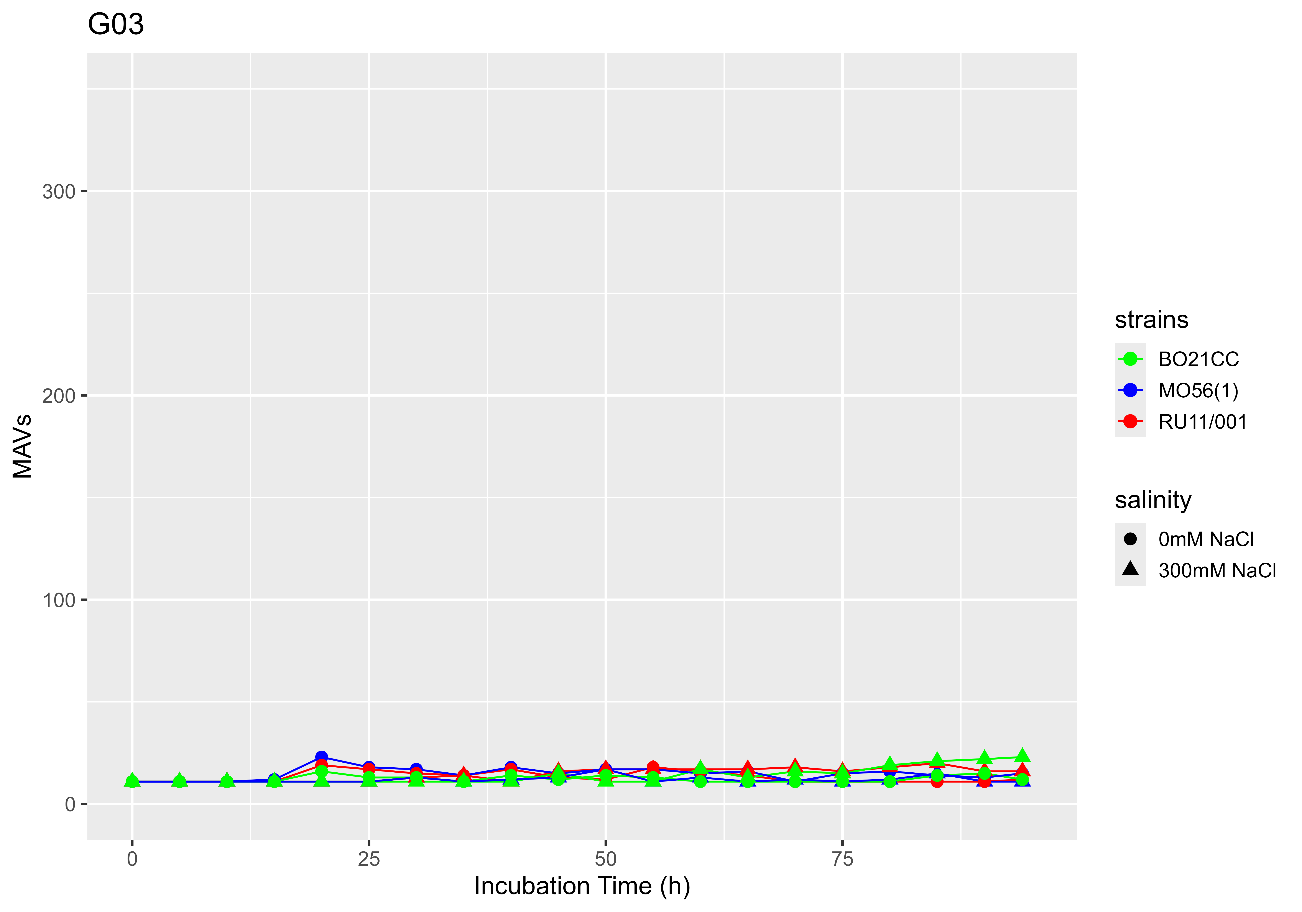
N-Acetyl-L-Glutamic acid**

**Glycine**

**
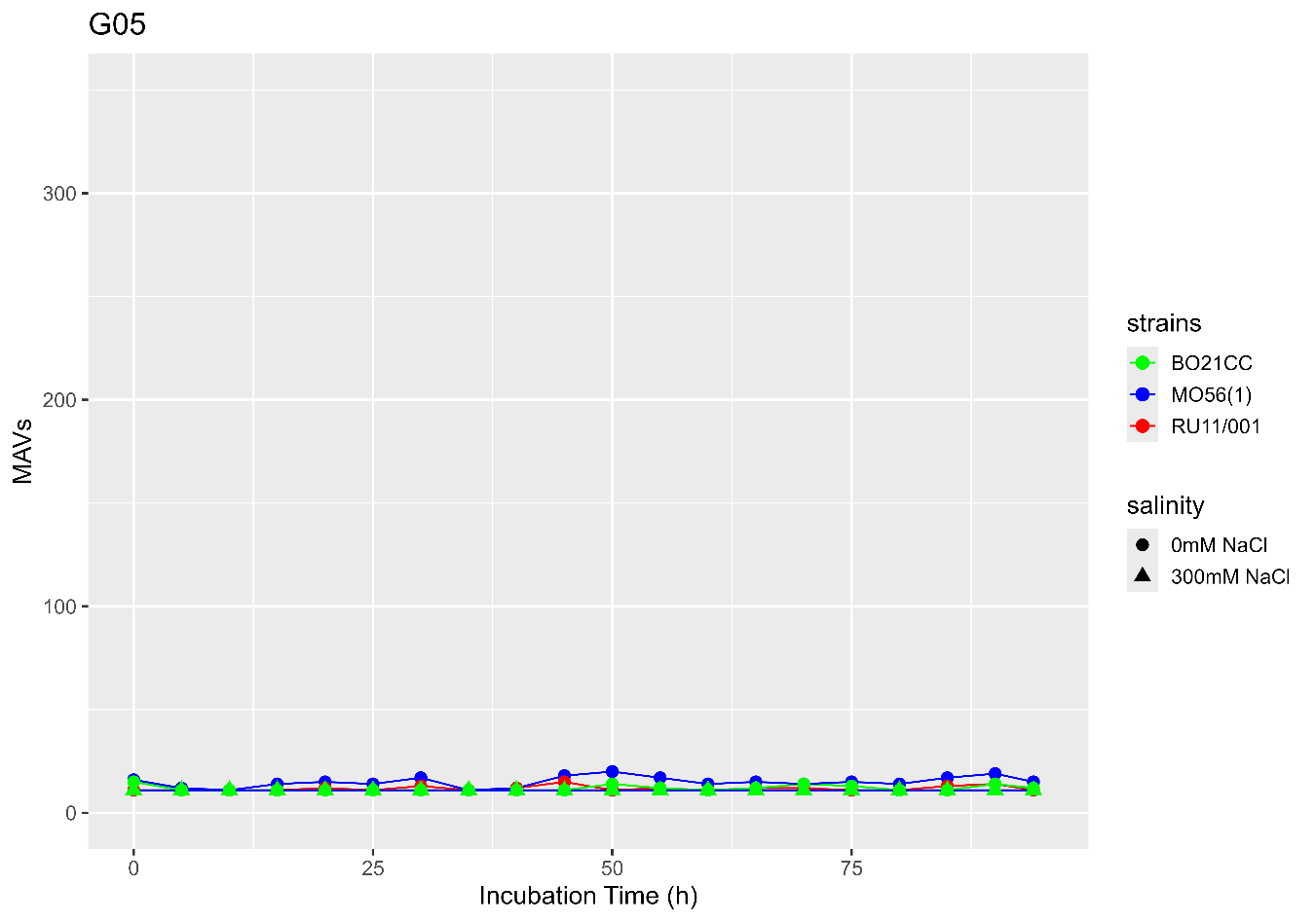
**

**
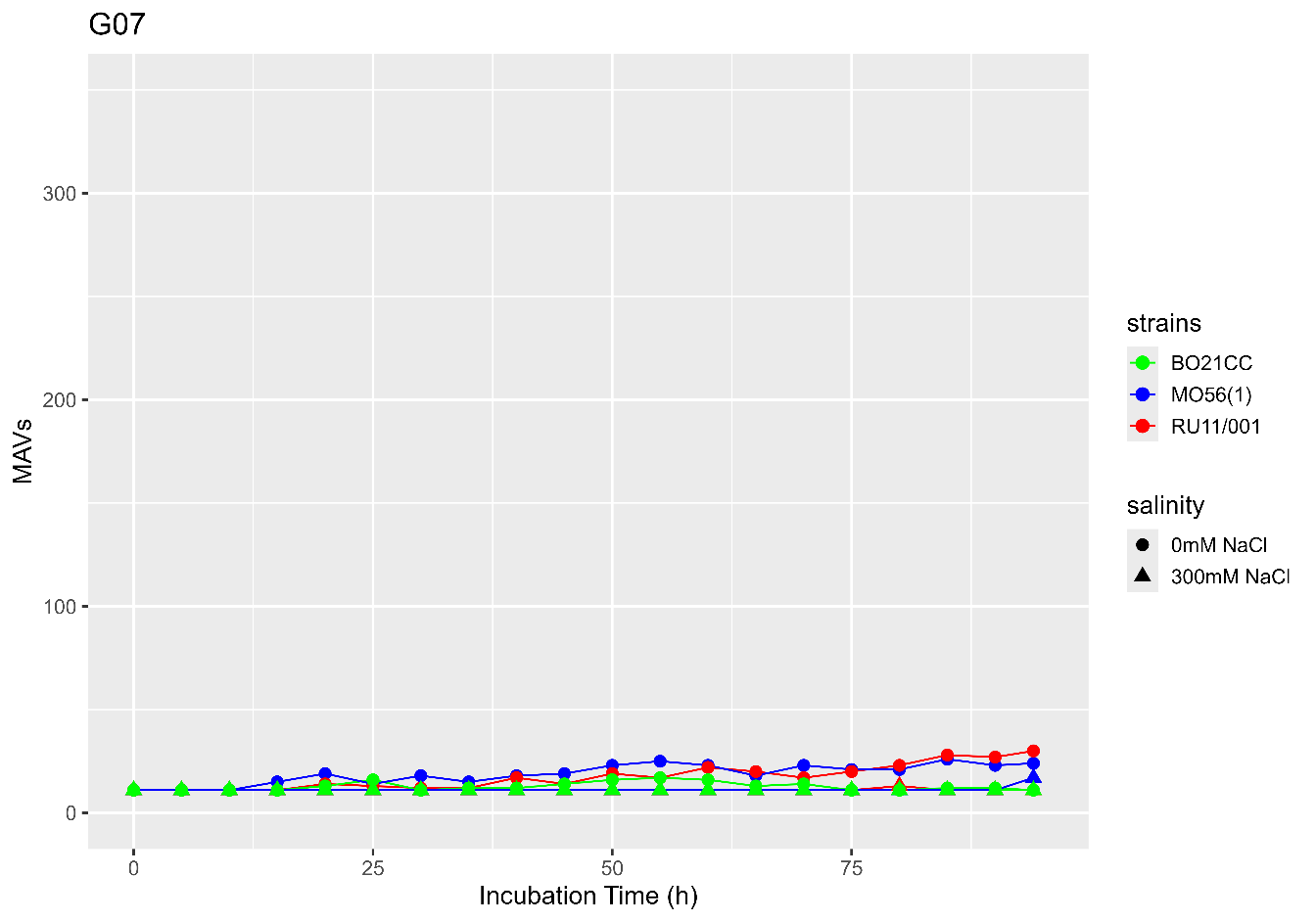
L-Homoserine**

**
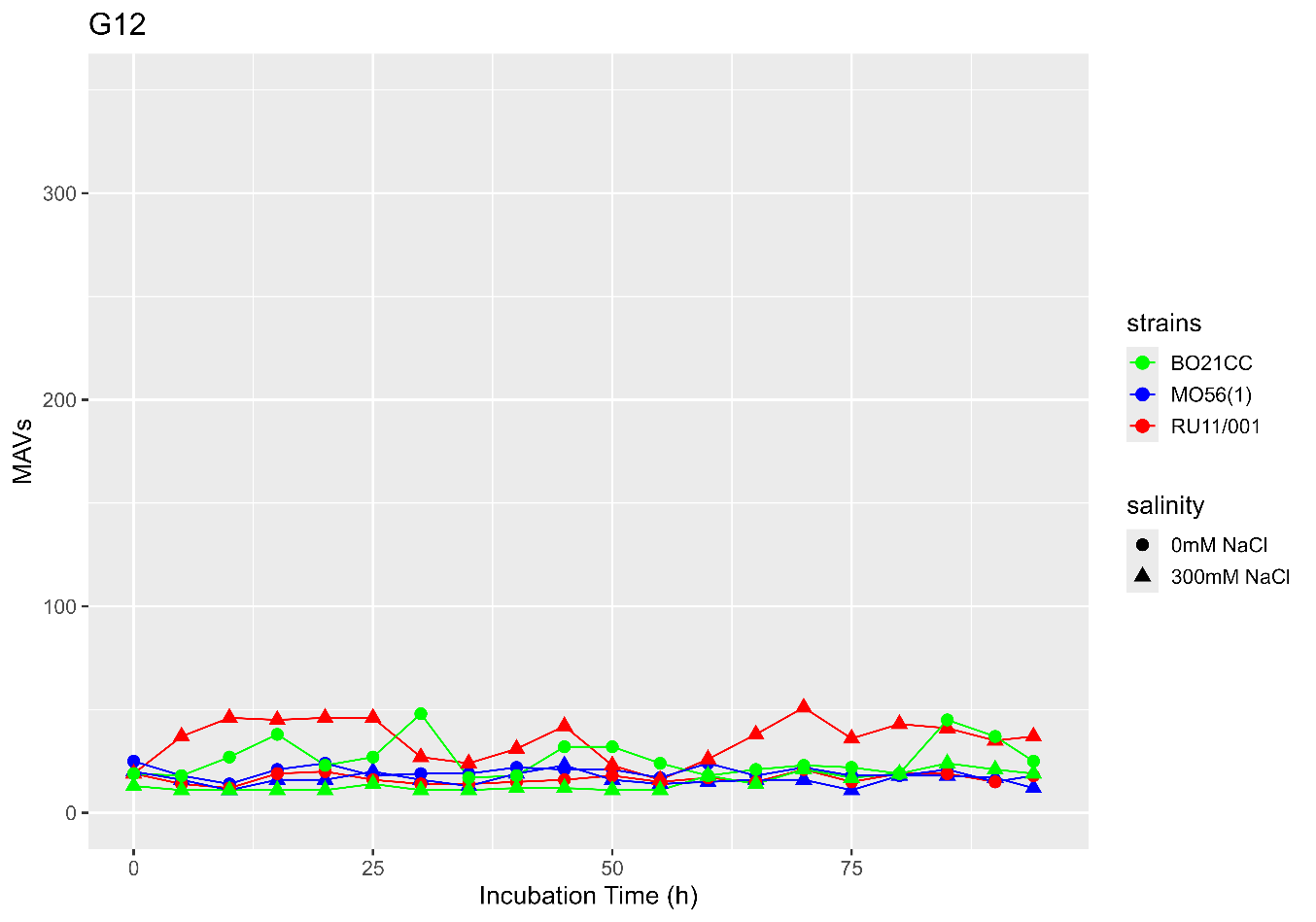
L-Methionine**

**
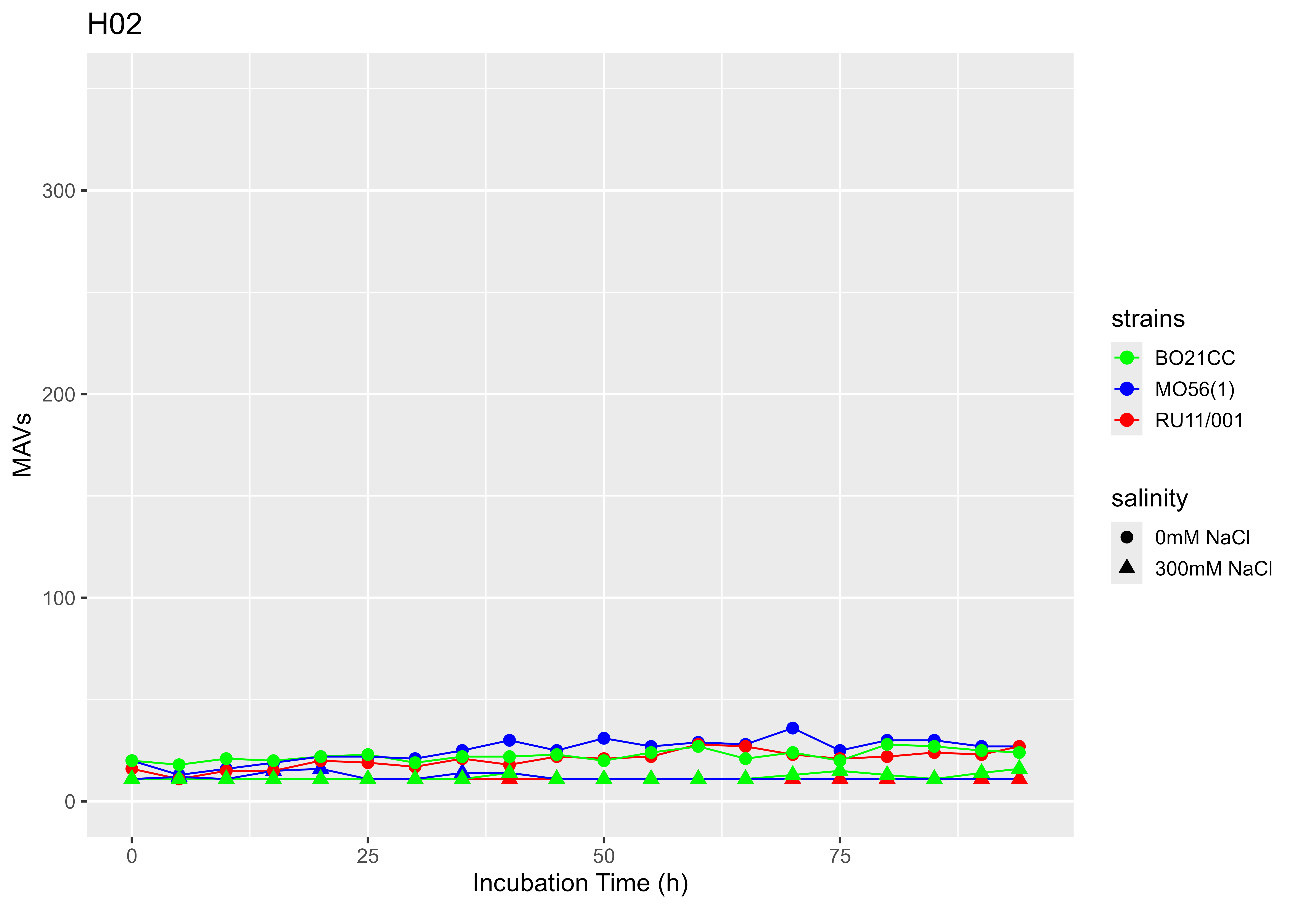
L-Phenylalanine**

**L-Pyroglutamic acid**

**
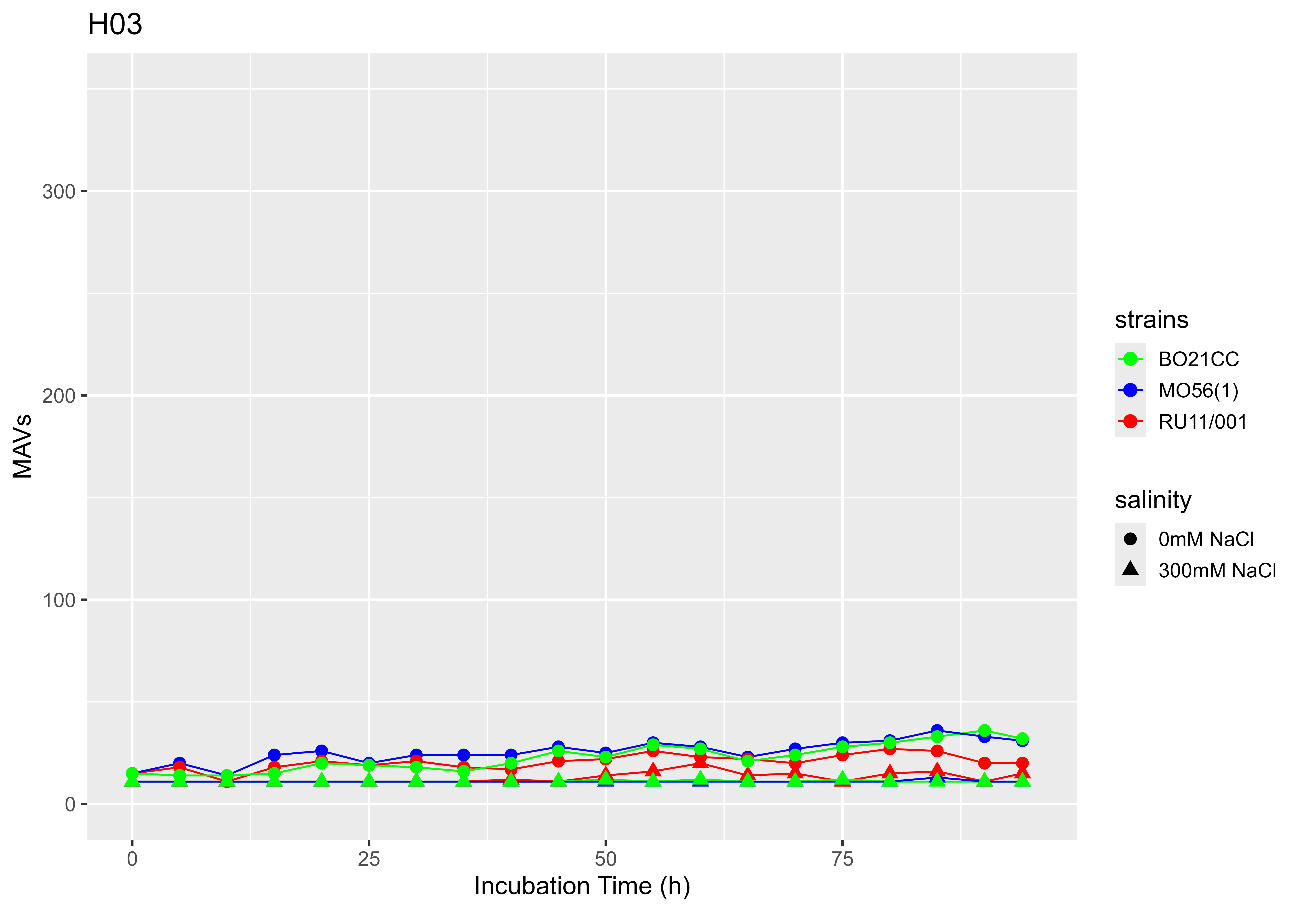
**

**
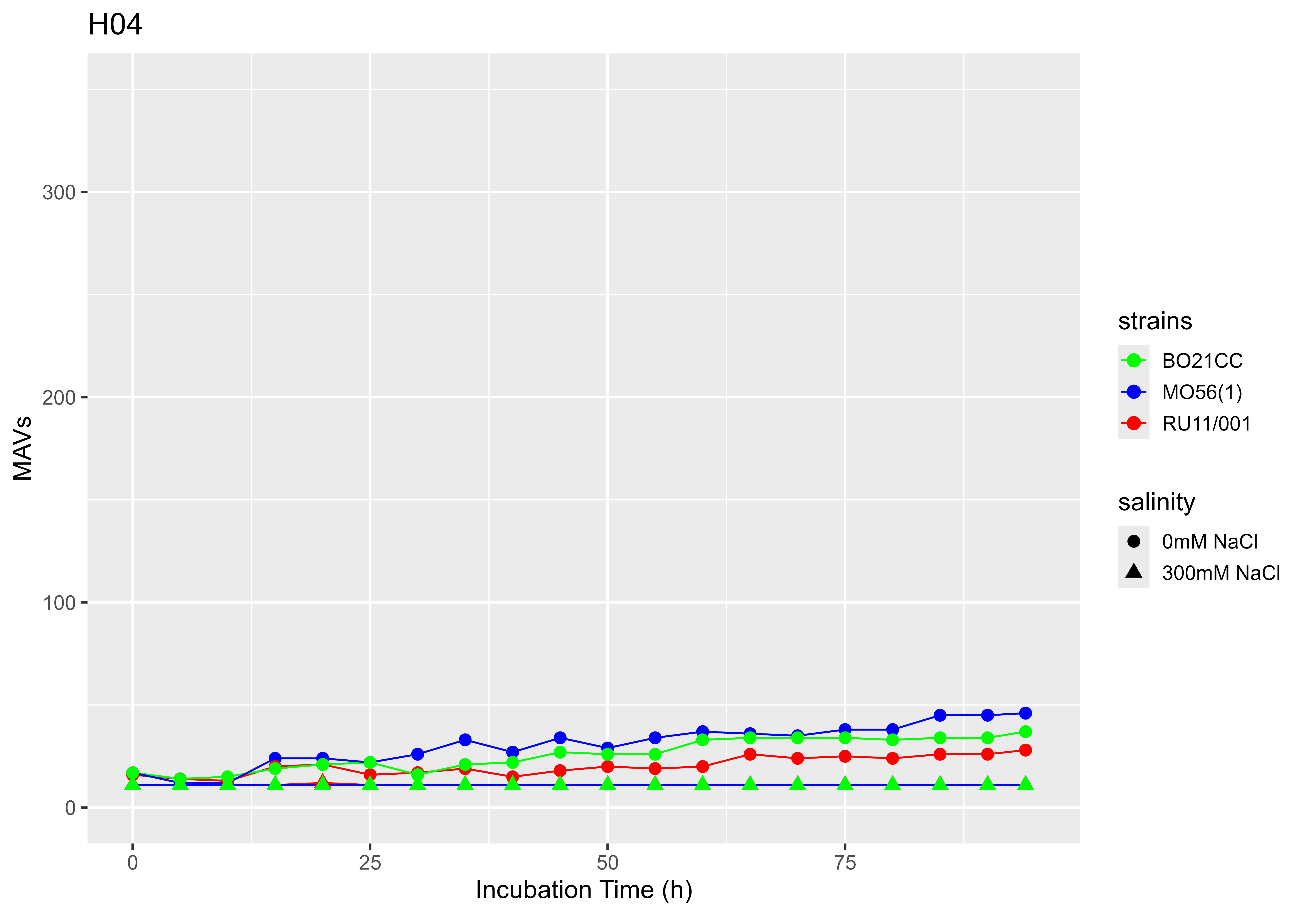
L-Valine**

**sec-Butylamine**

**
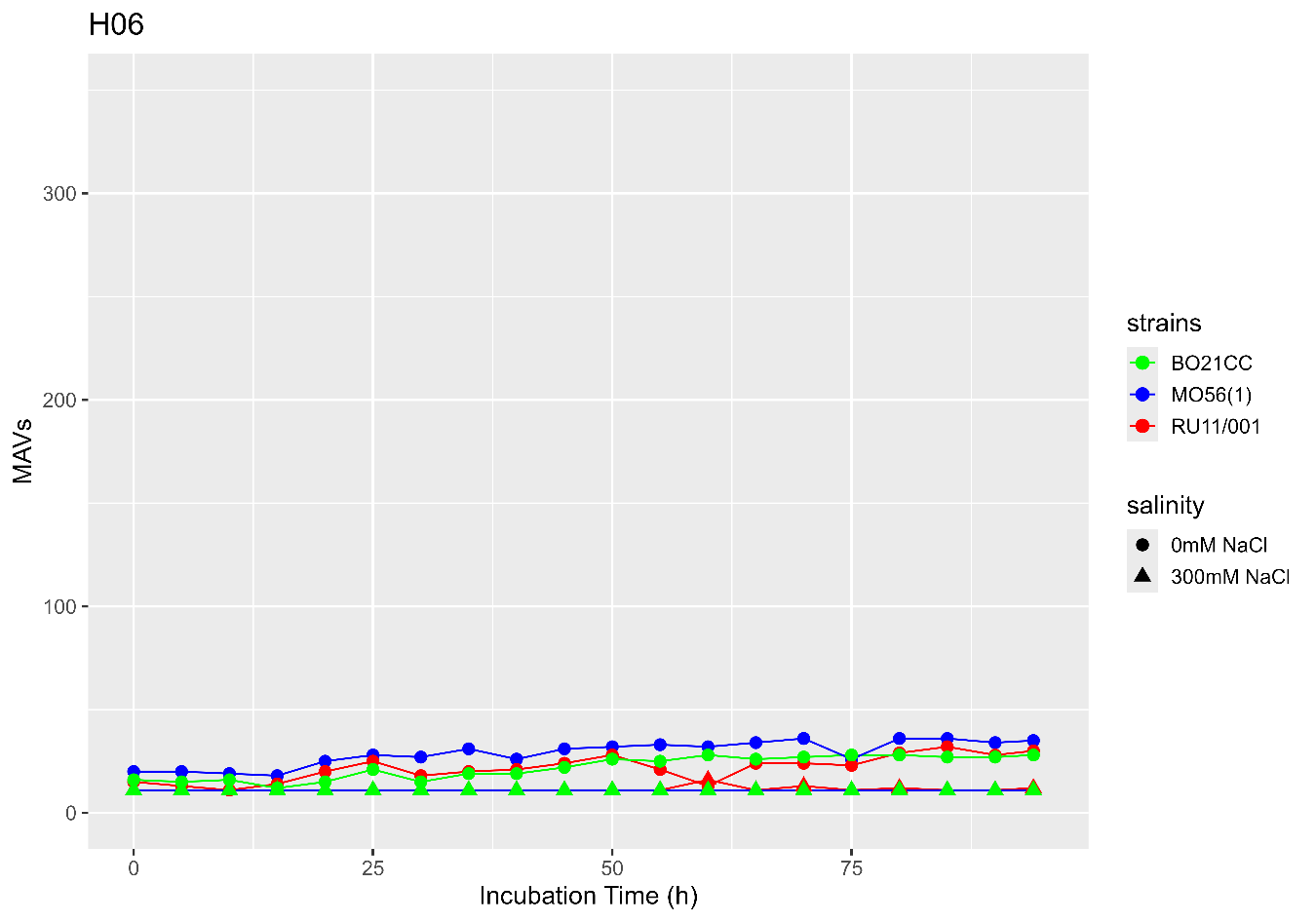
**

**
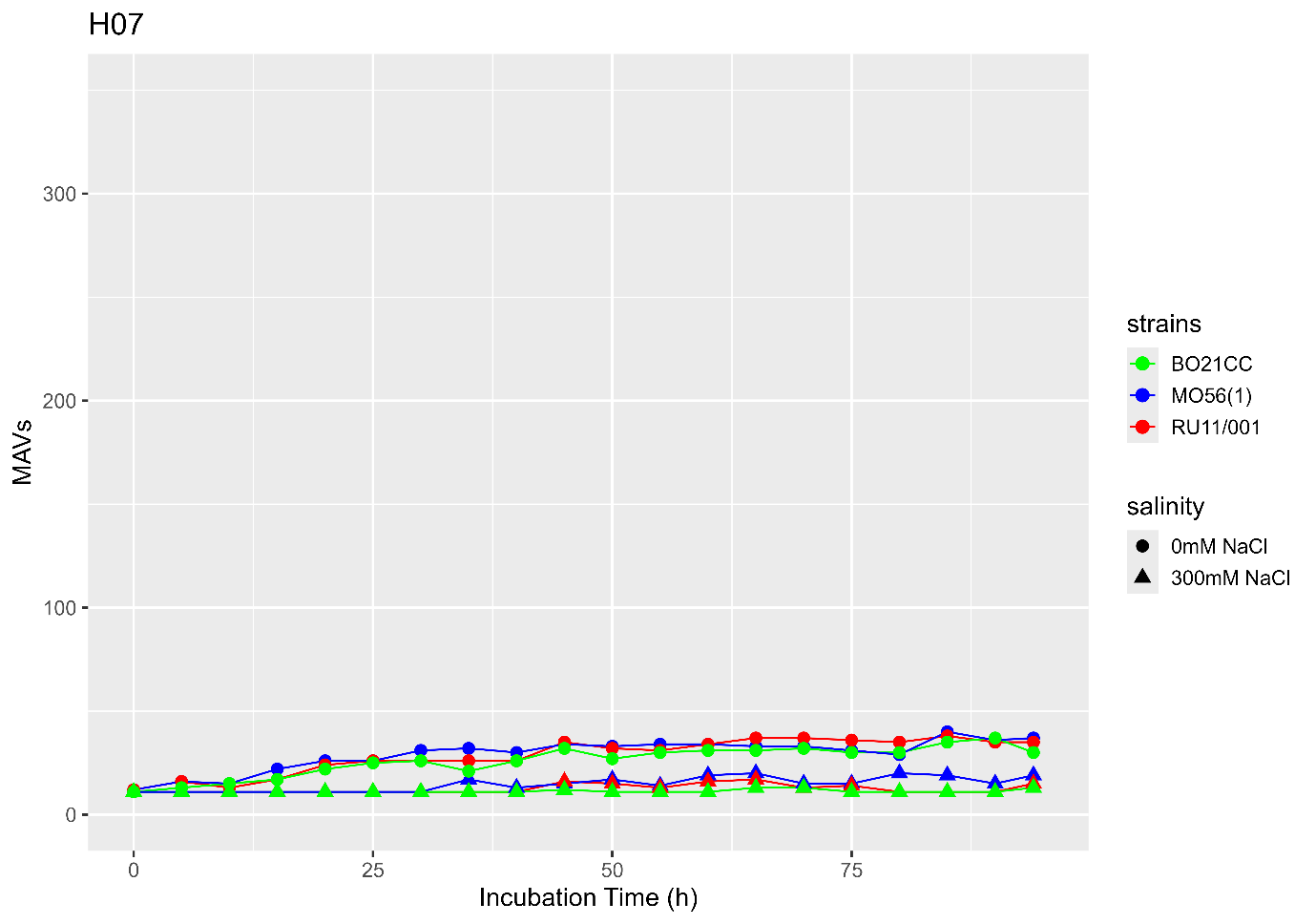
D,L-Octopamine**

**
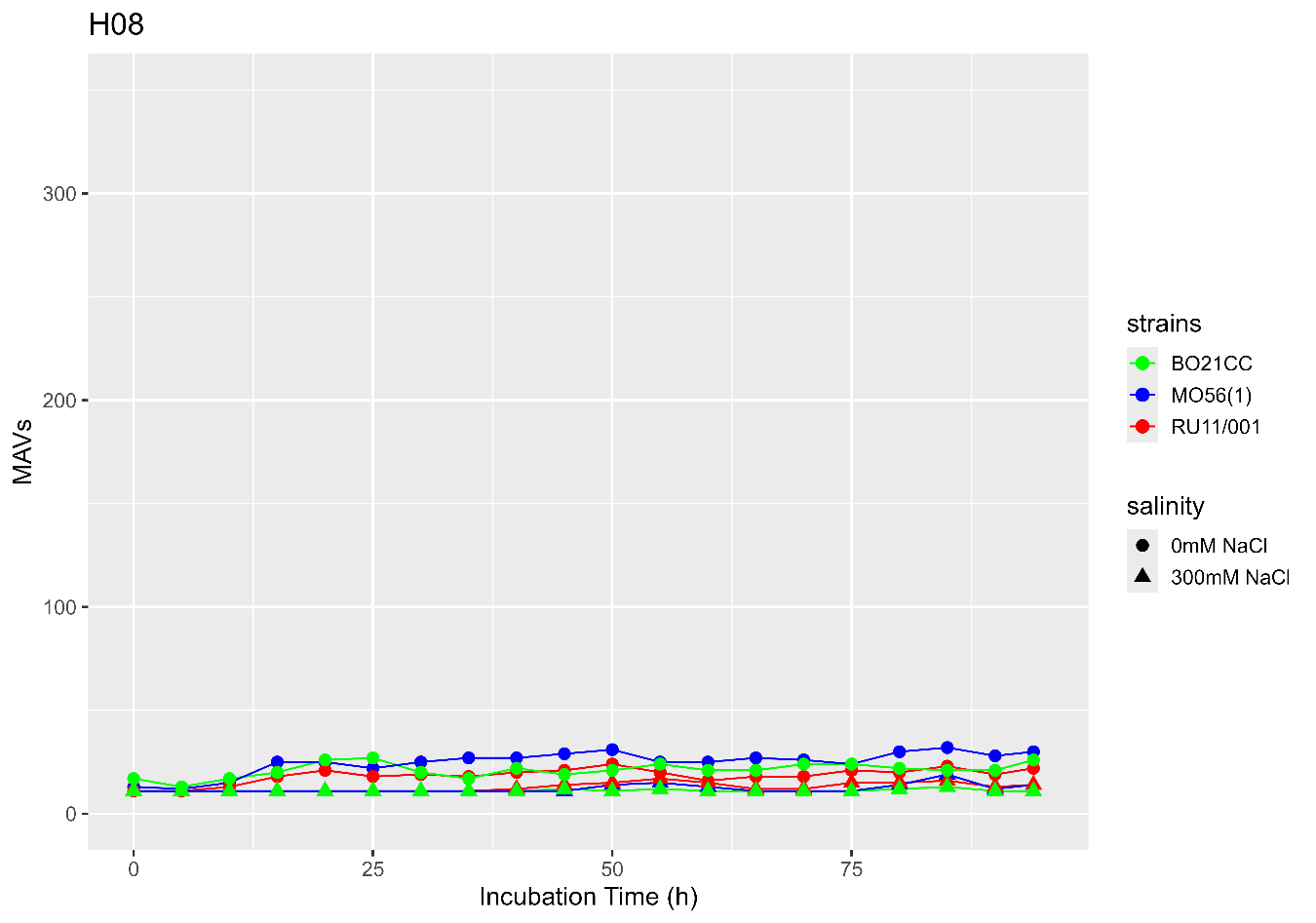
Putrescine**

**2,3-Butanediol**

**
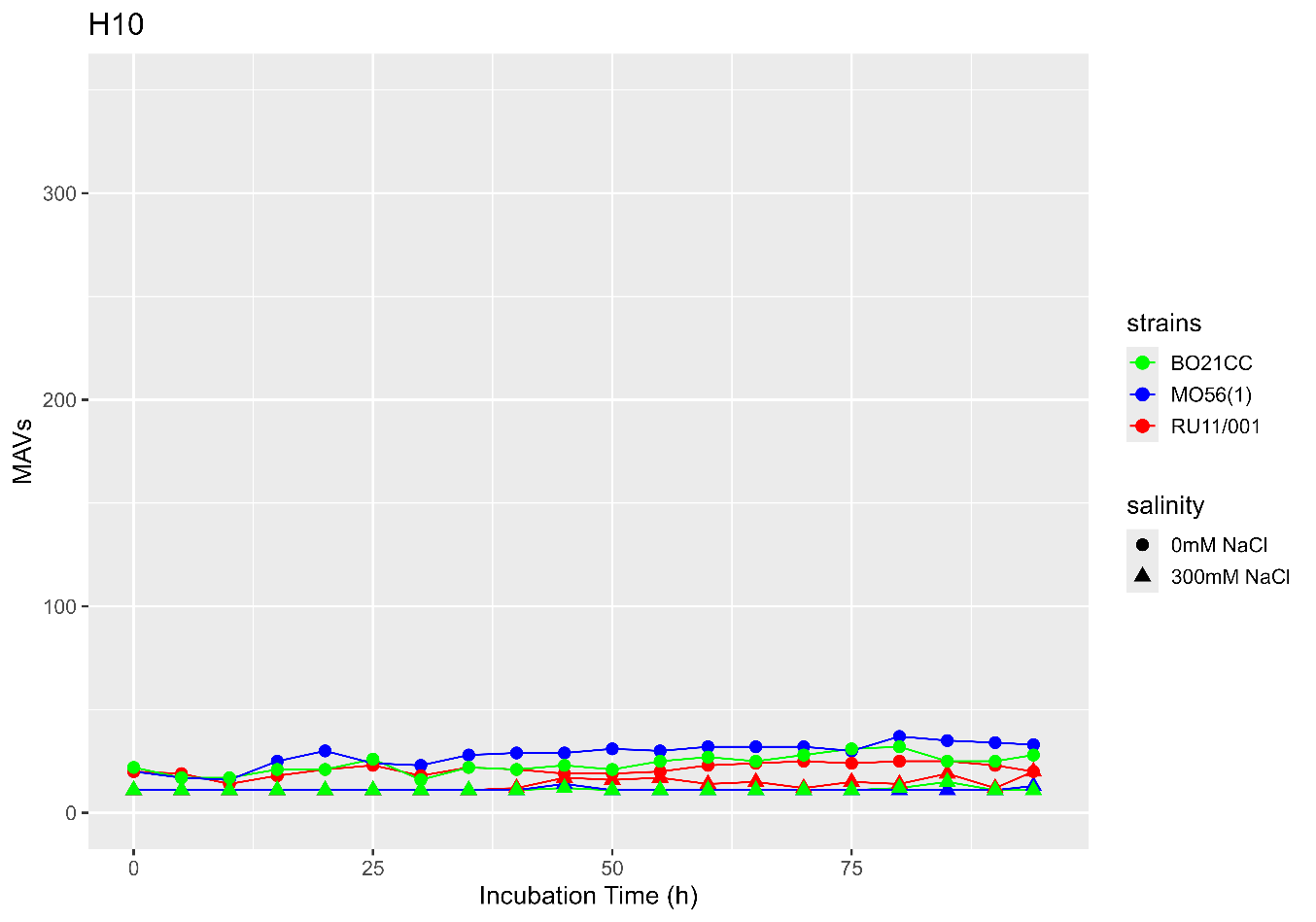
**

**2,3-Butanone**

**
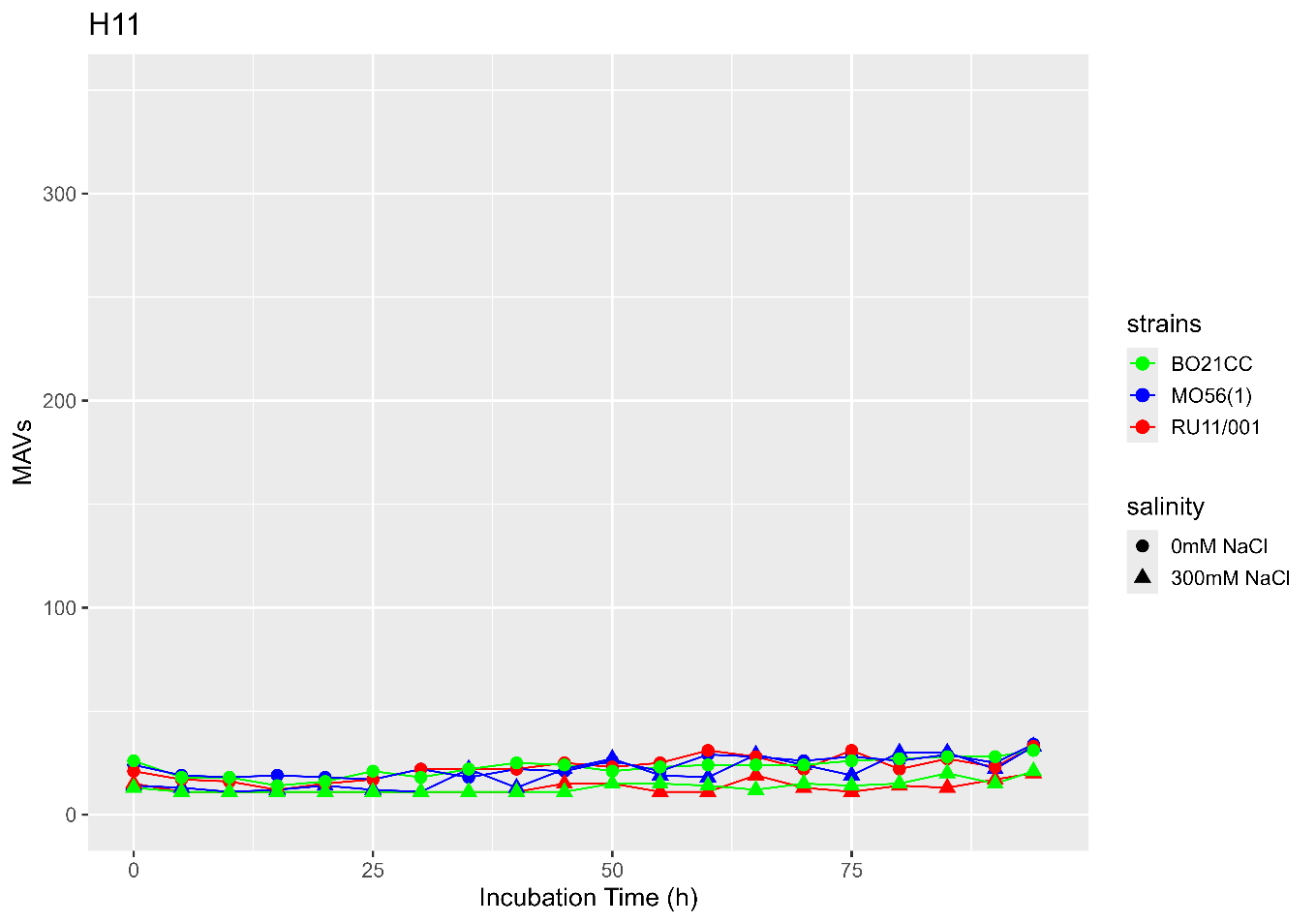
**
