# Supplementary material for: Genomic and Phenotypic Bases of Salt Tolerance in Sinorhizobium meliloti : Candidate Traits for Bioinoculant Development Addressing Saline Soils
Source: Microb Biotechnol. 2026 Jan 29;19(1):e70304. doi: 10.1111/1751-7915.70304 (PMC12855168; doi:10.1111/1751-7915.70304)
Supplement: Supplementary file 14 — File S8: Metabolic activity values of strains NaCl‐R+ (BO21CC and RU11/001) and NaCl‐R‐ (RU11/001) in presence and absence of NaCl (0 mM and 300 mM NaCl) on some carbon sources putatively involved in salt resistance. [file MBT2-19-e70304-s012.docx]

**File S8**

**Metabolic activity values of strains** **NaCl-R+ (BO21CC and RU11/001) and NaCl-R- (RU11/001) in presence and absence of NaCl (0 mM and 300 mM NaCl) on some carbon sources putatively involved in salt resistance.**

**
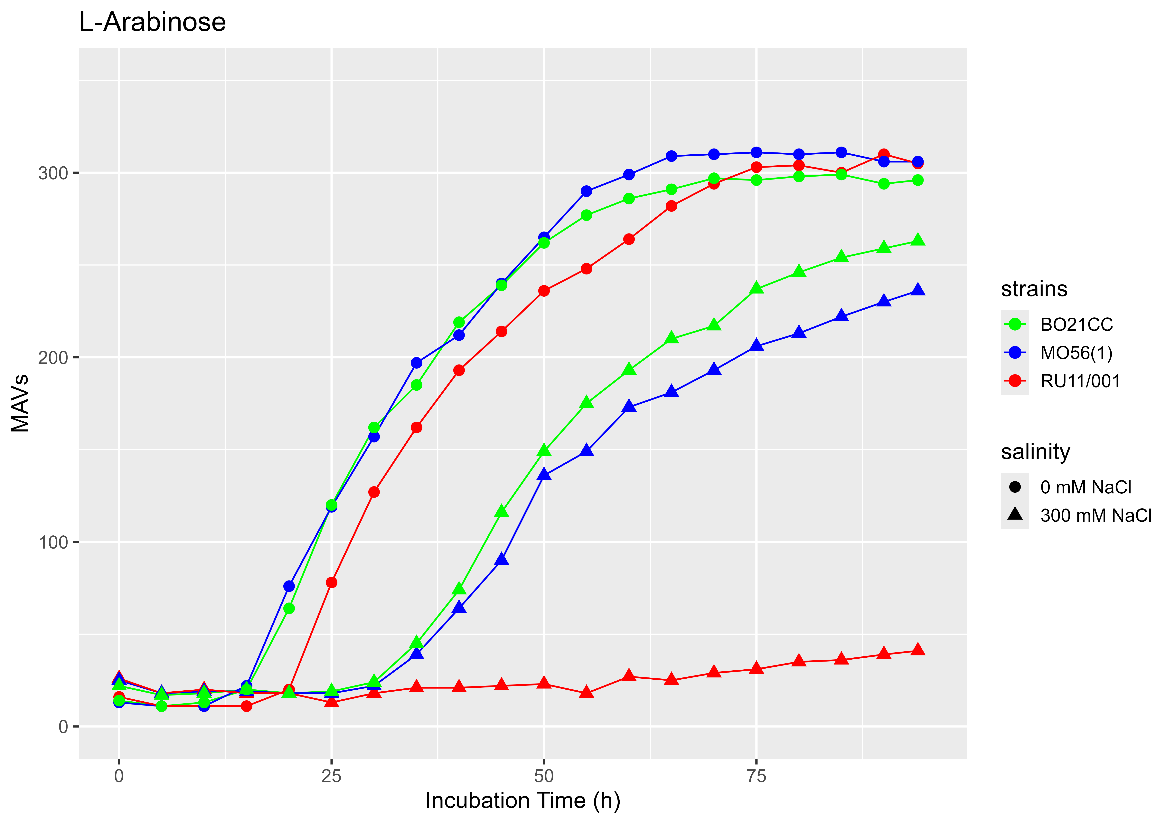
L-Arabinose**

**D-galactose**

**
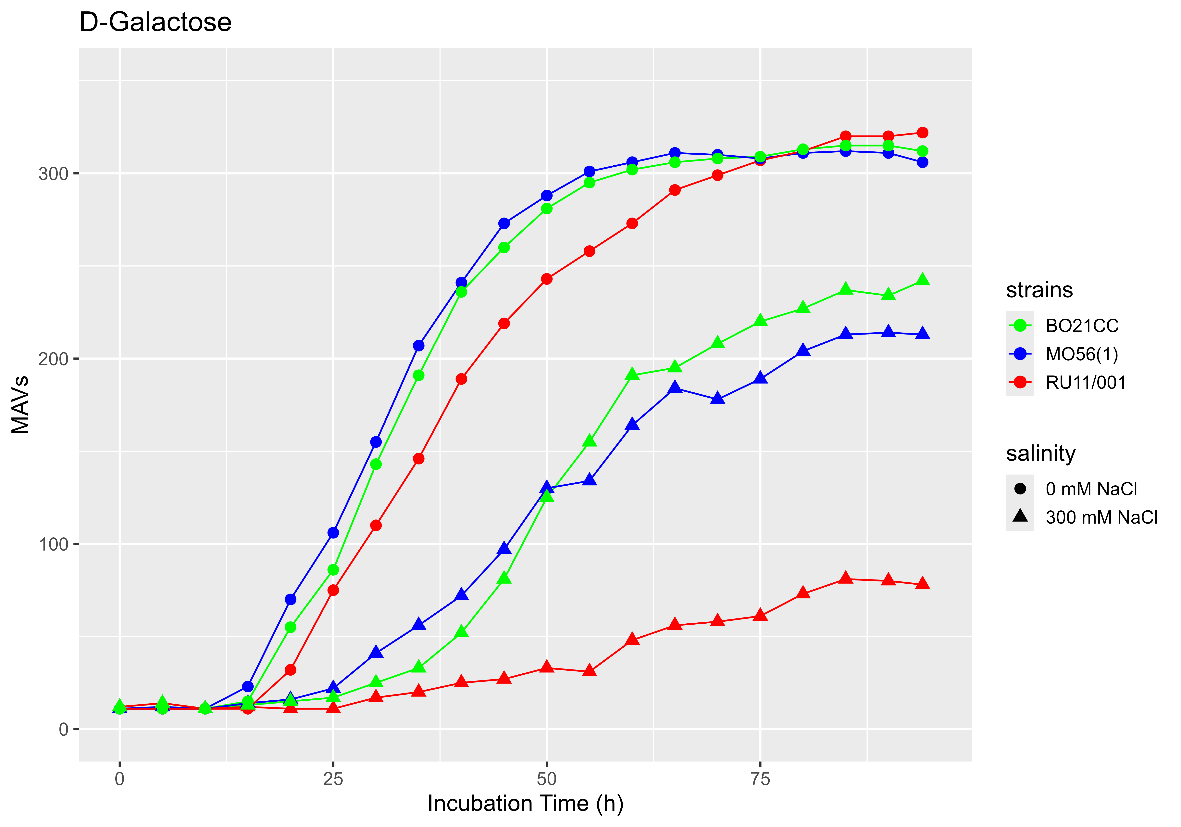
**

**
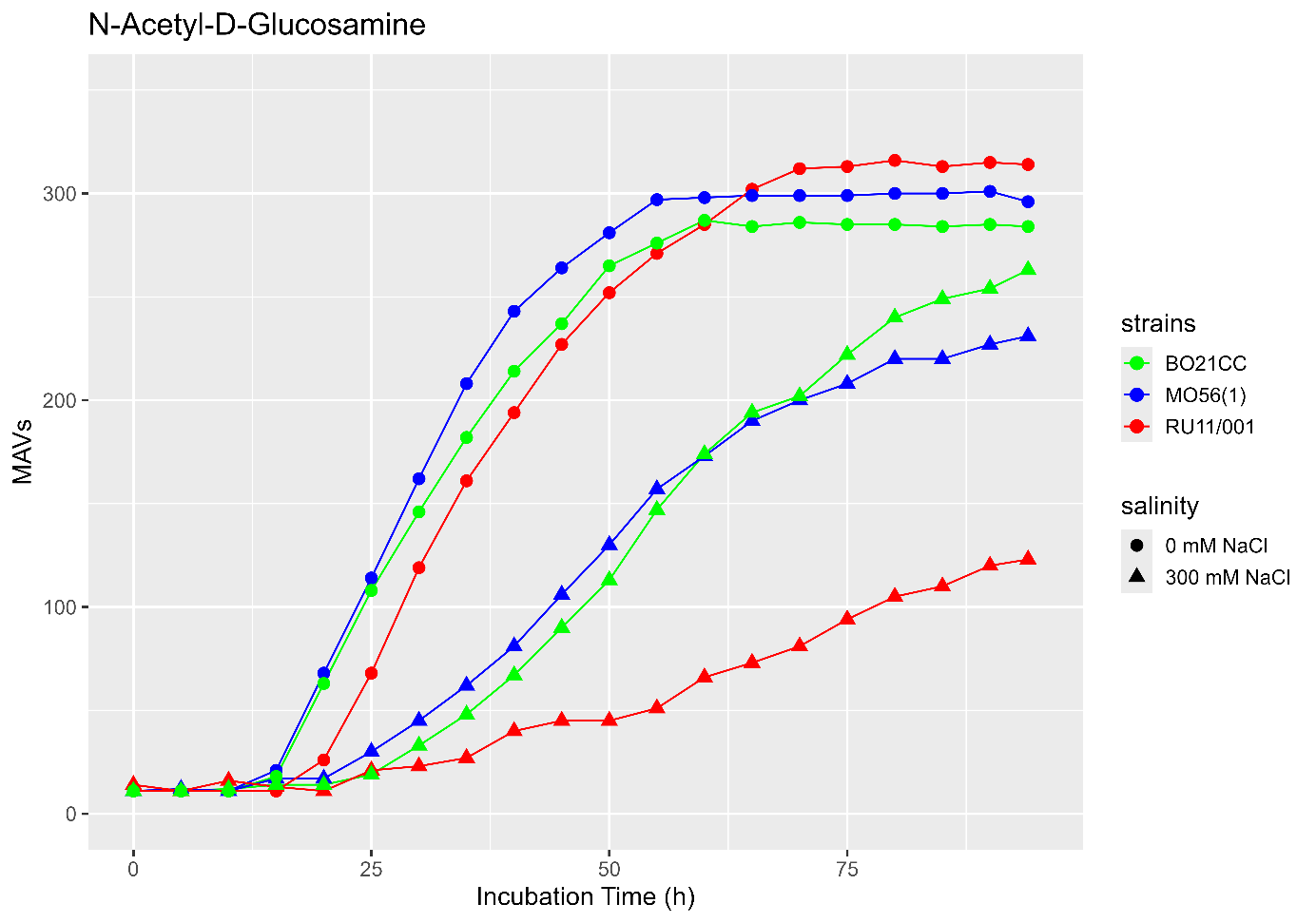
N-acetyl-D-glucosamine**

**Gentiobiose**

**
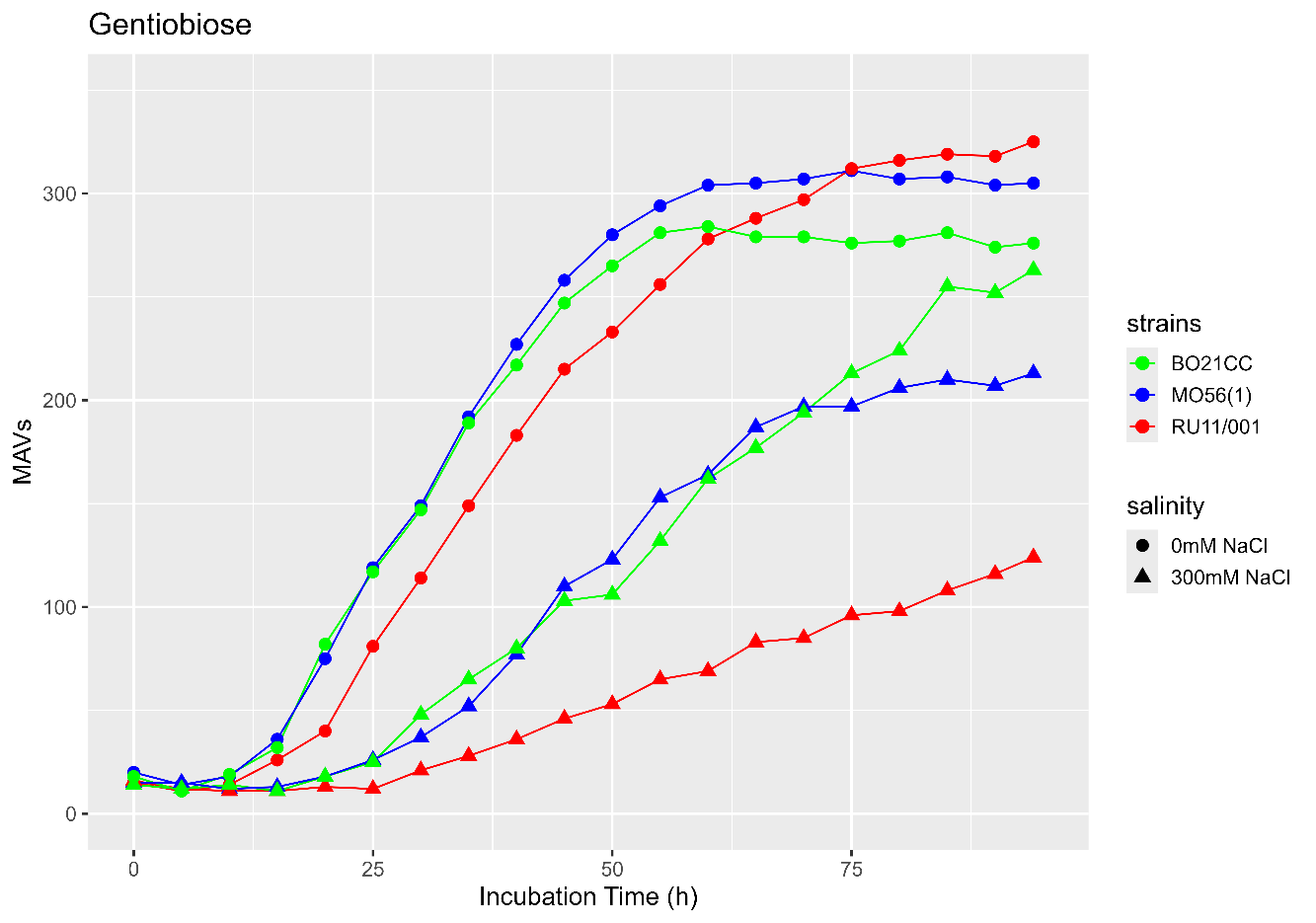
**

**
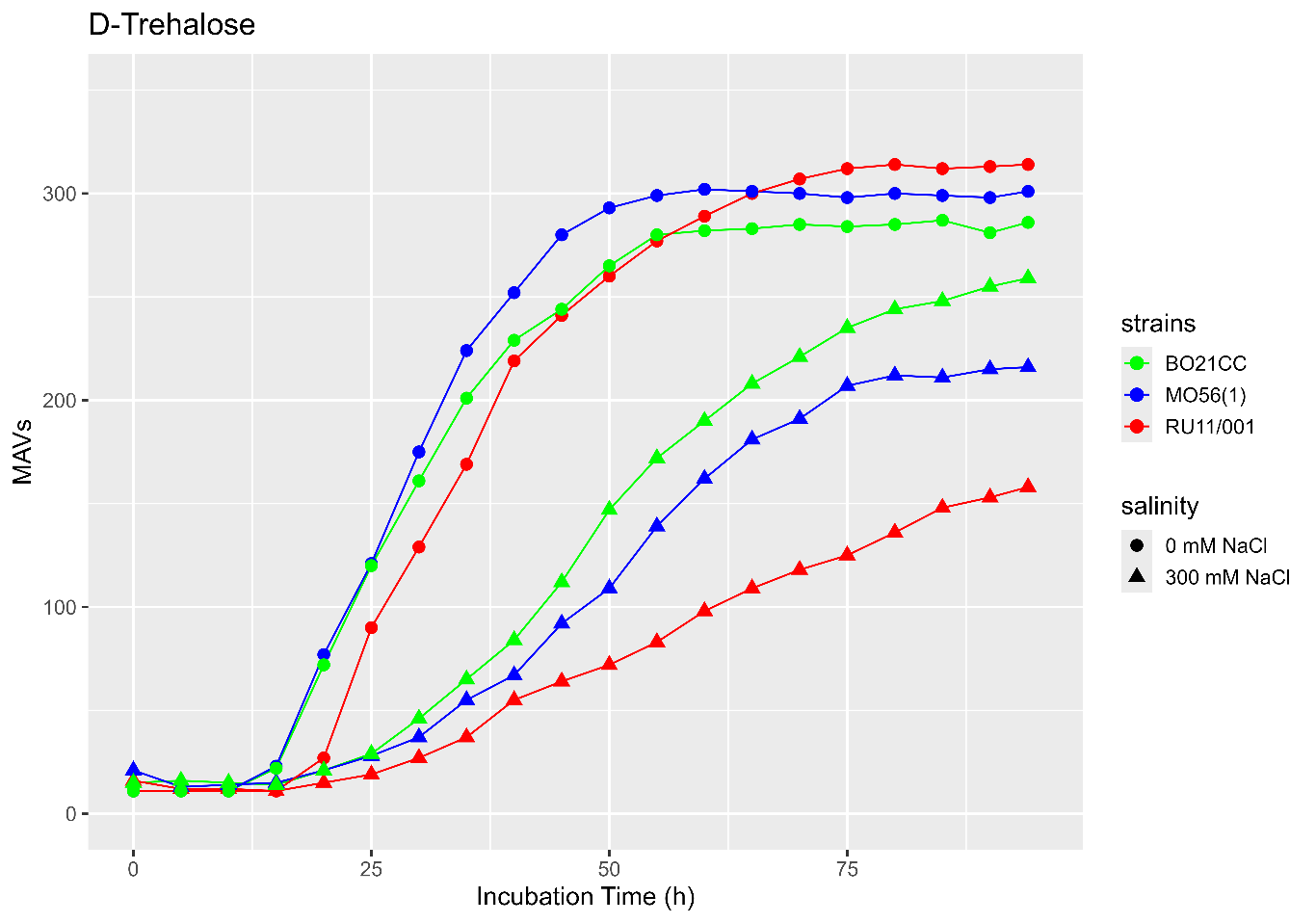
D-trehalose**

**D-mannose**

**
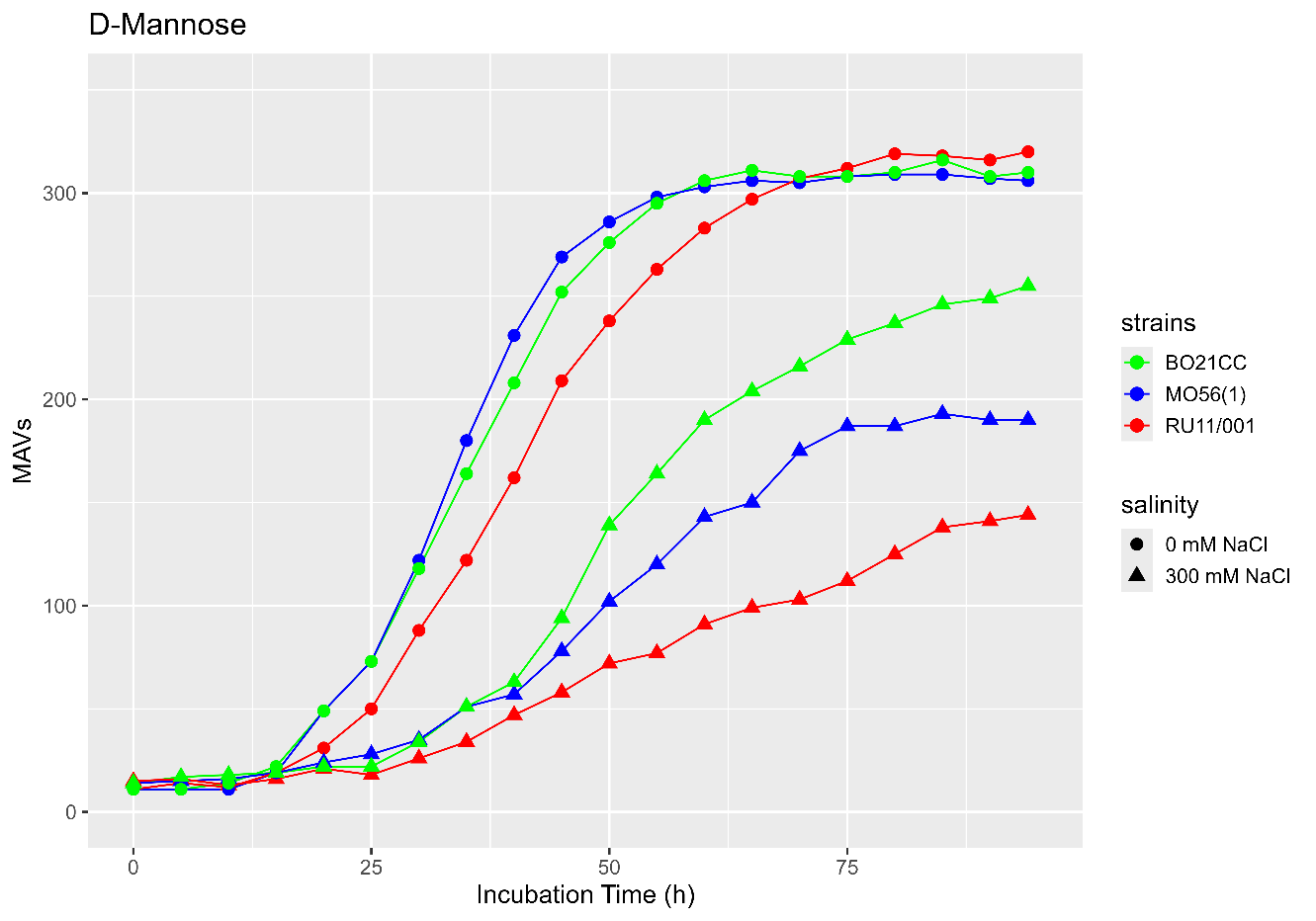
**

**
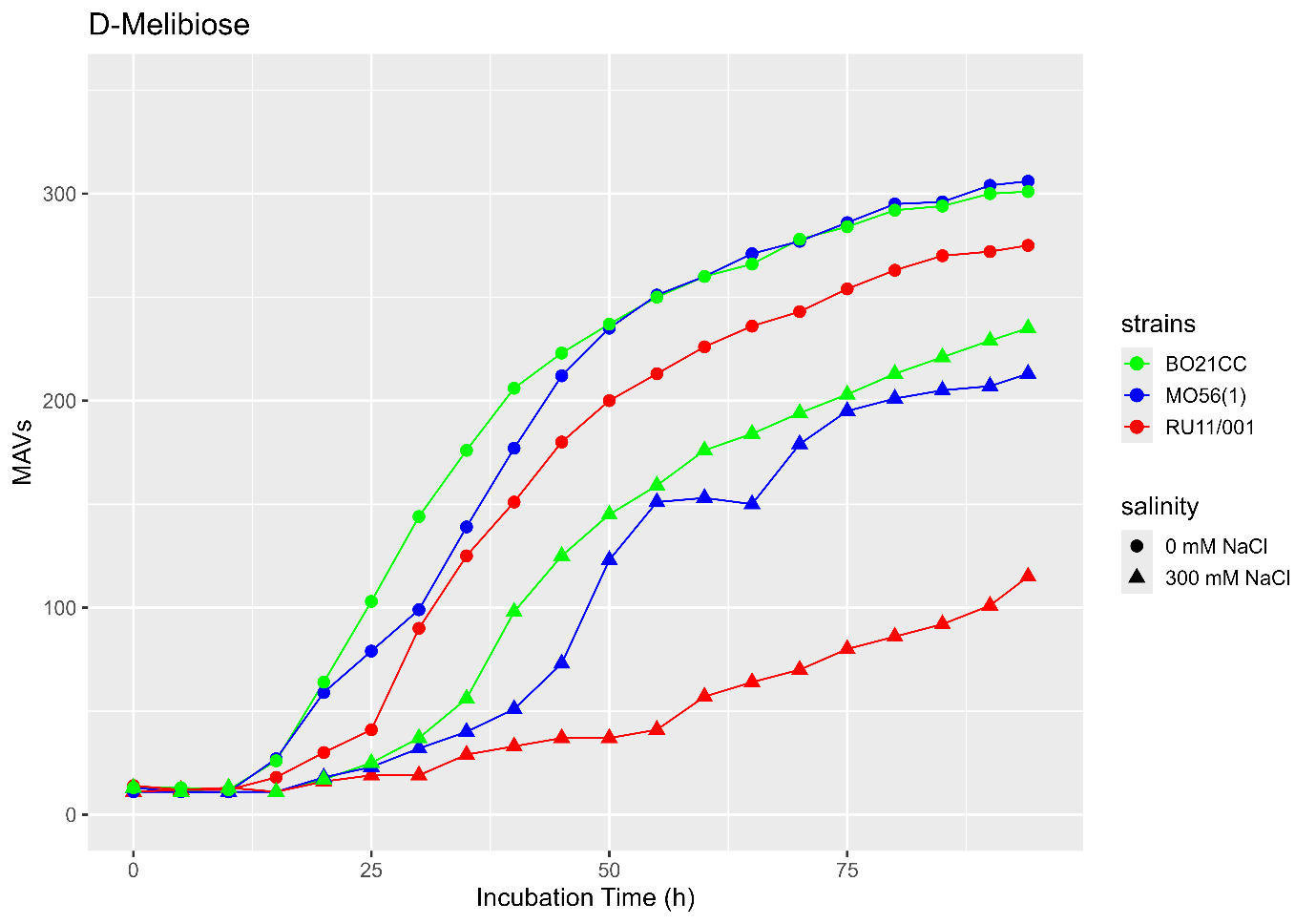
D-melibiose**

**Maltotriose**

**
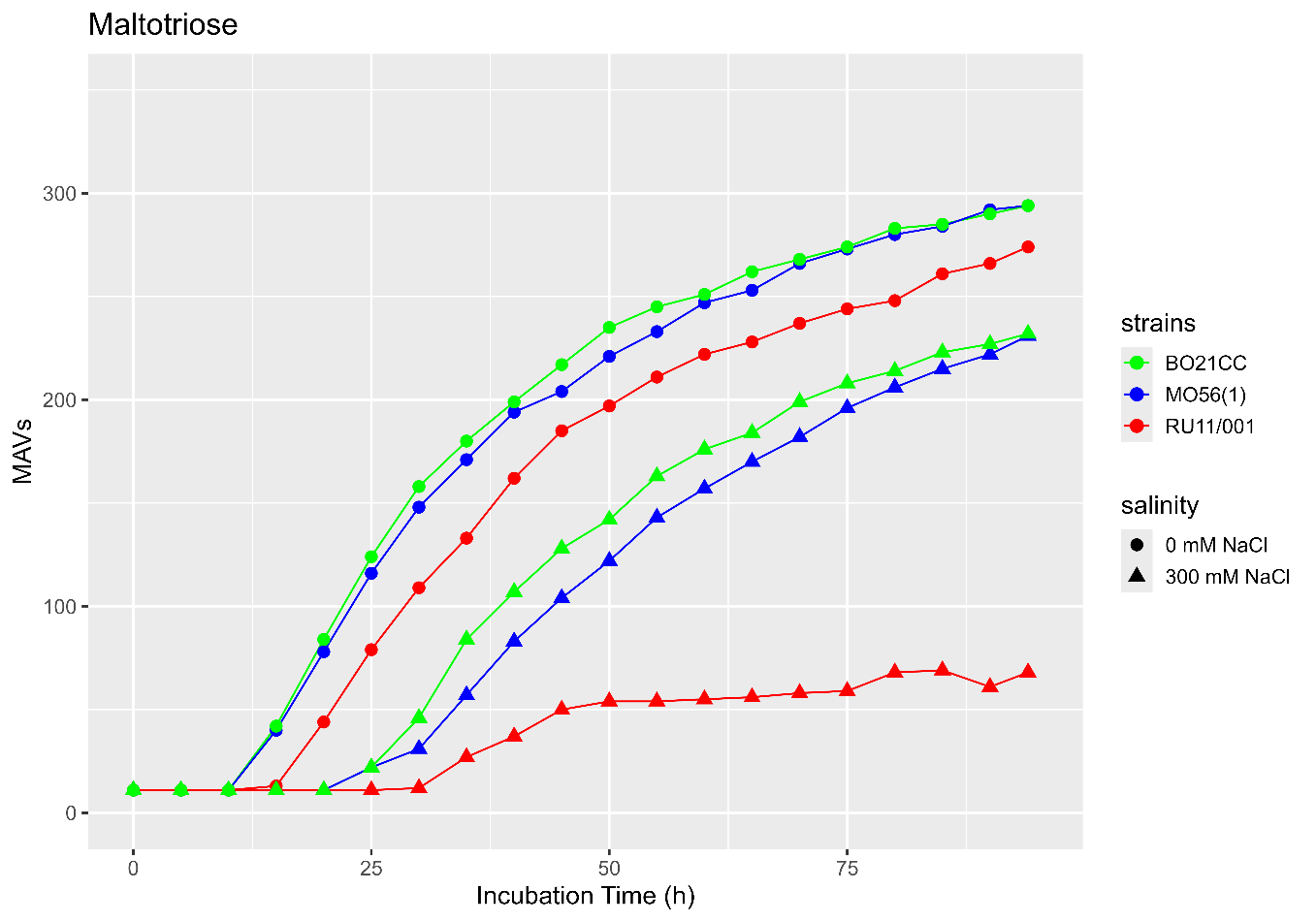
**

**Turanose**

**
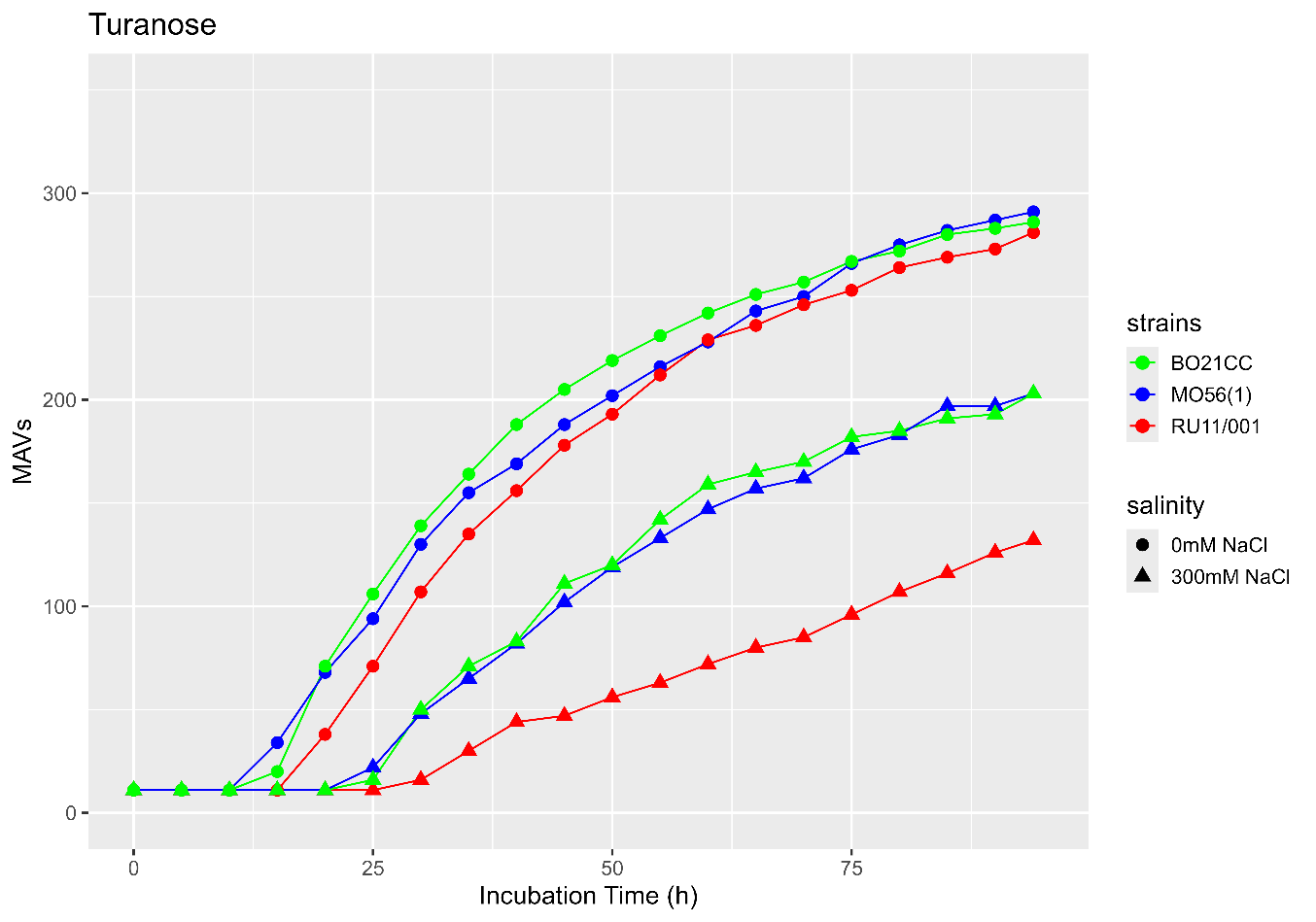
**

**α-D-glucose**

**
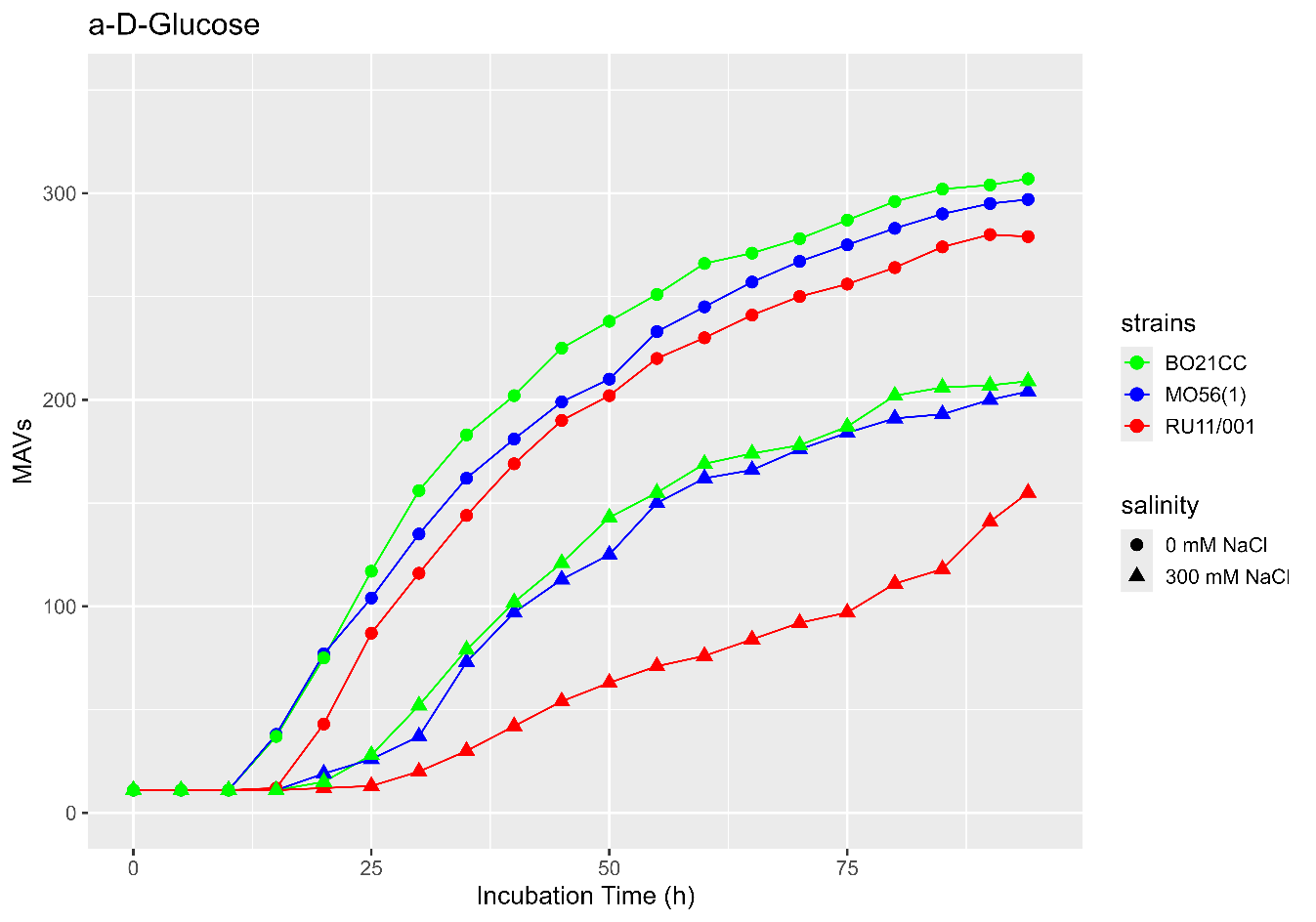
**

**Sucrose**

**
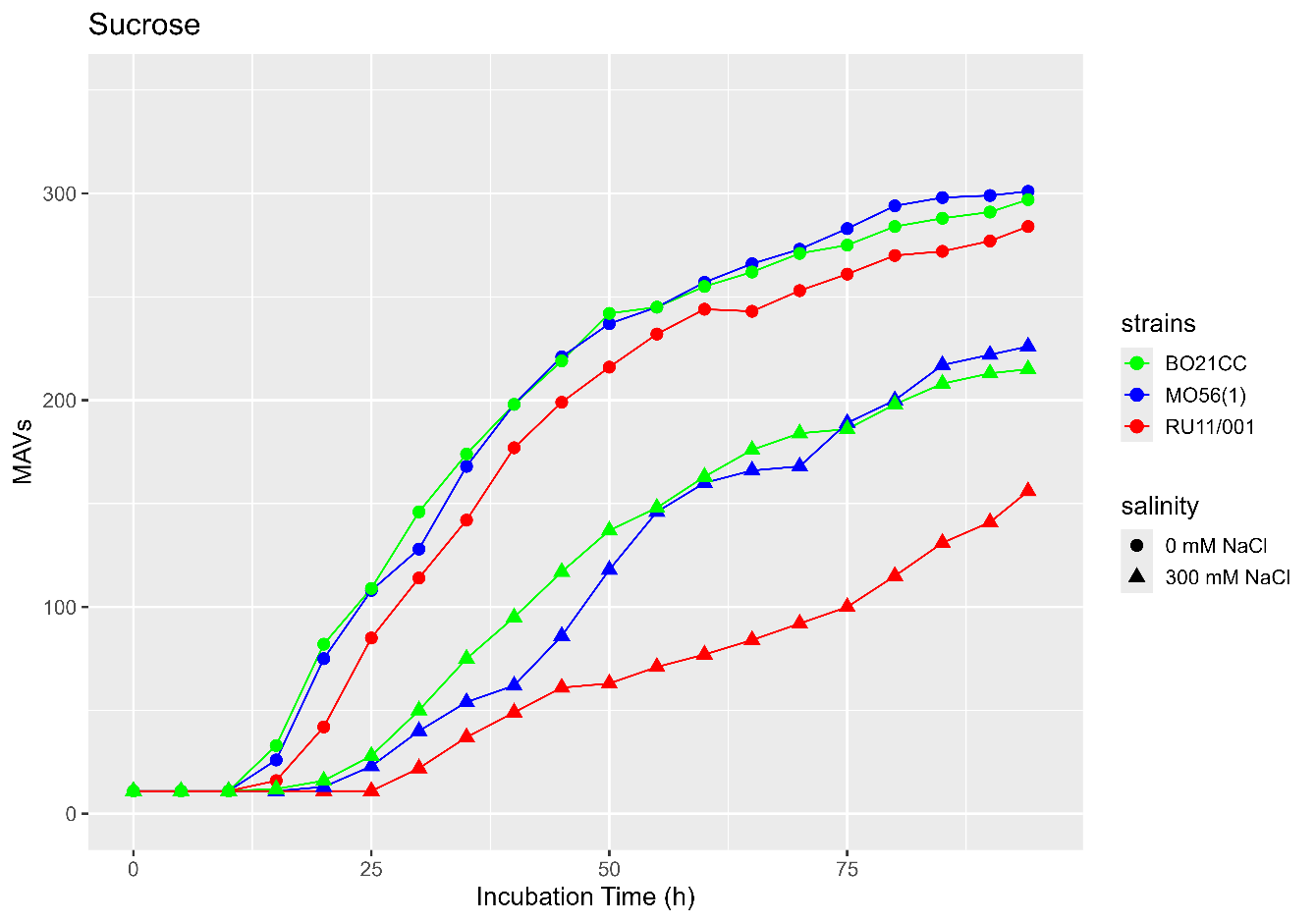
**

**
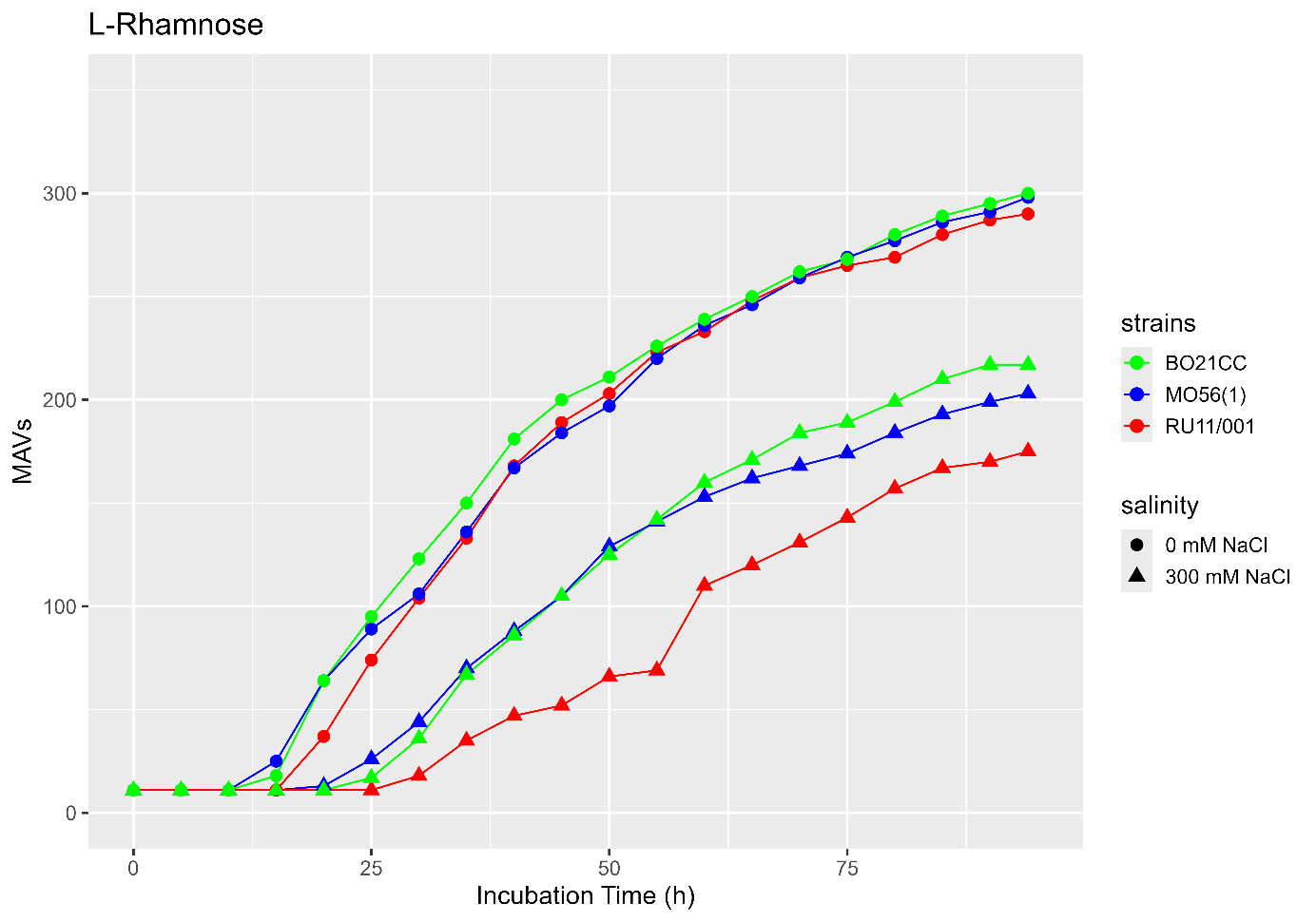
L-rhamnose**

**
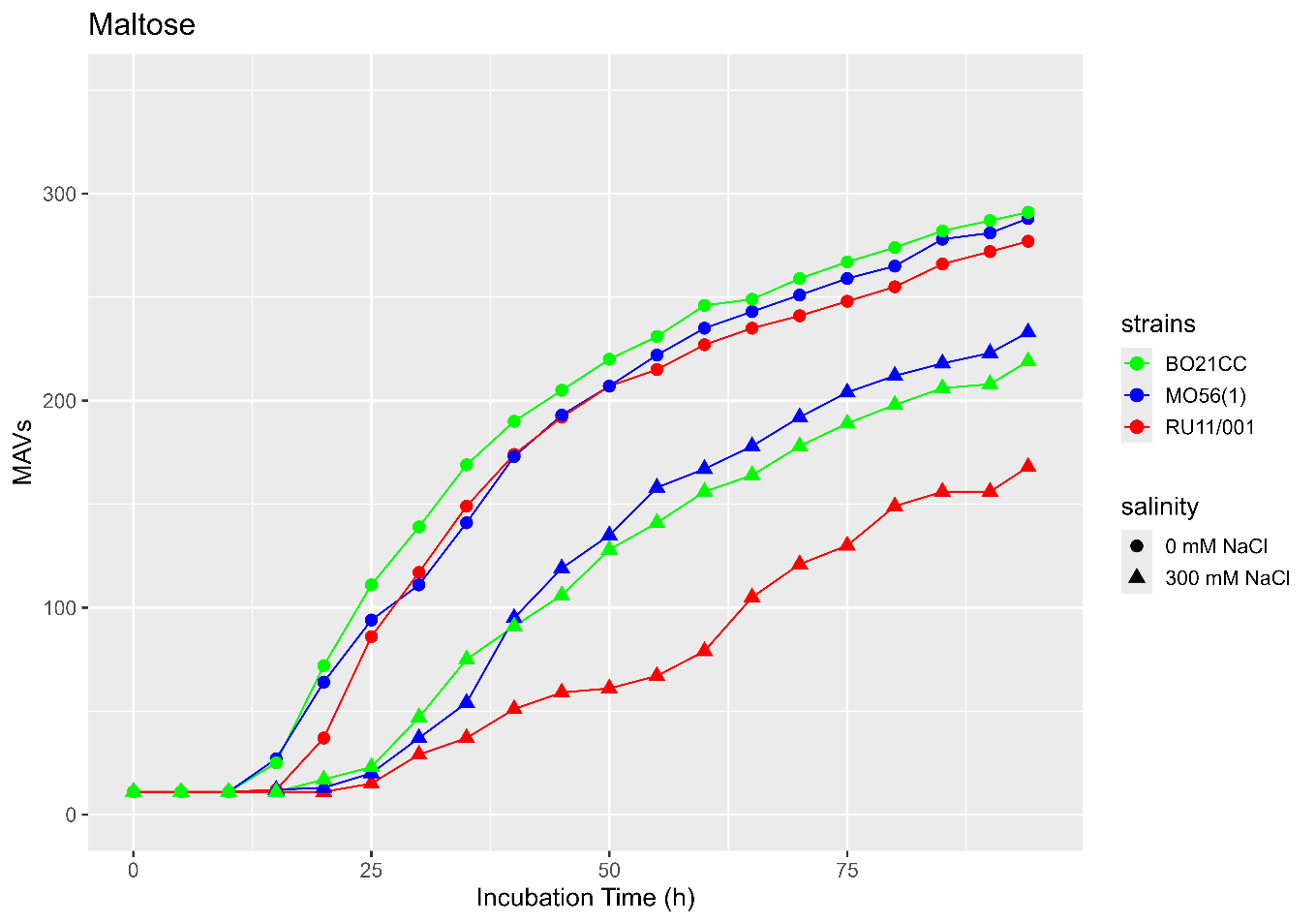
Maltose**

**L-fucose**

**
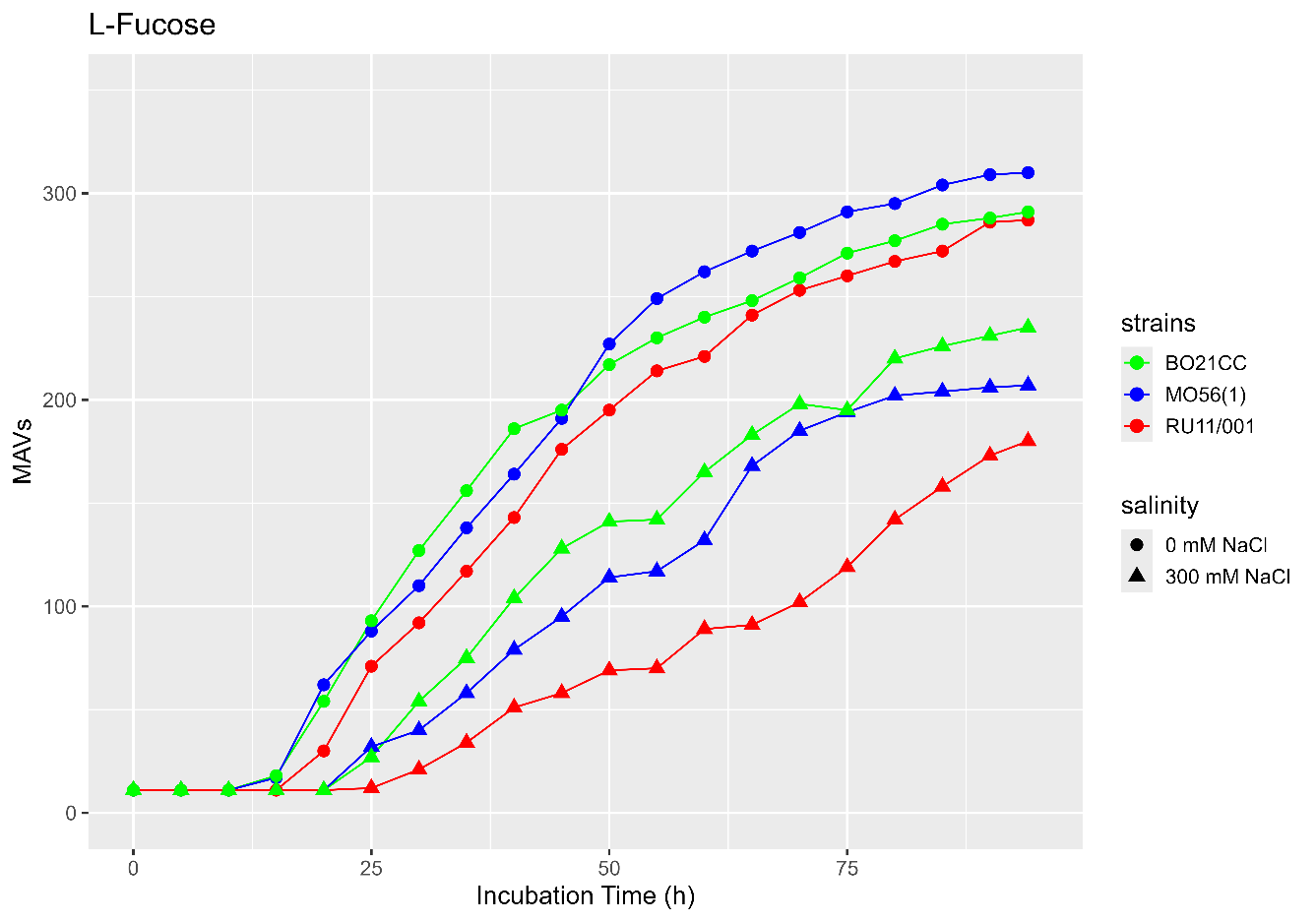
**

**D-cellobiose**


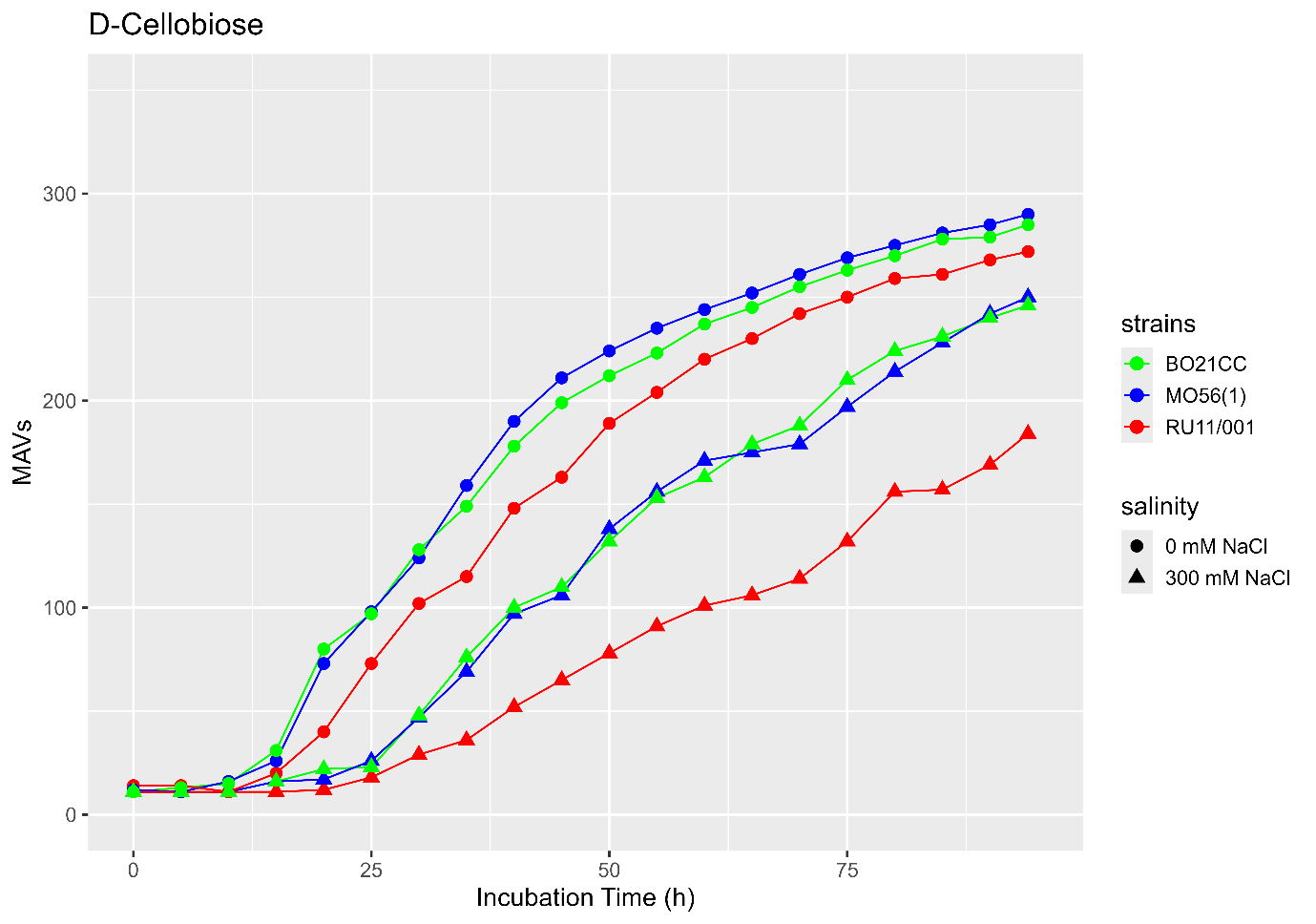


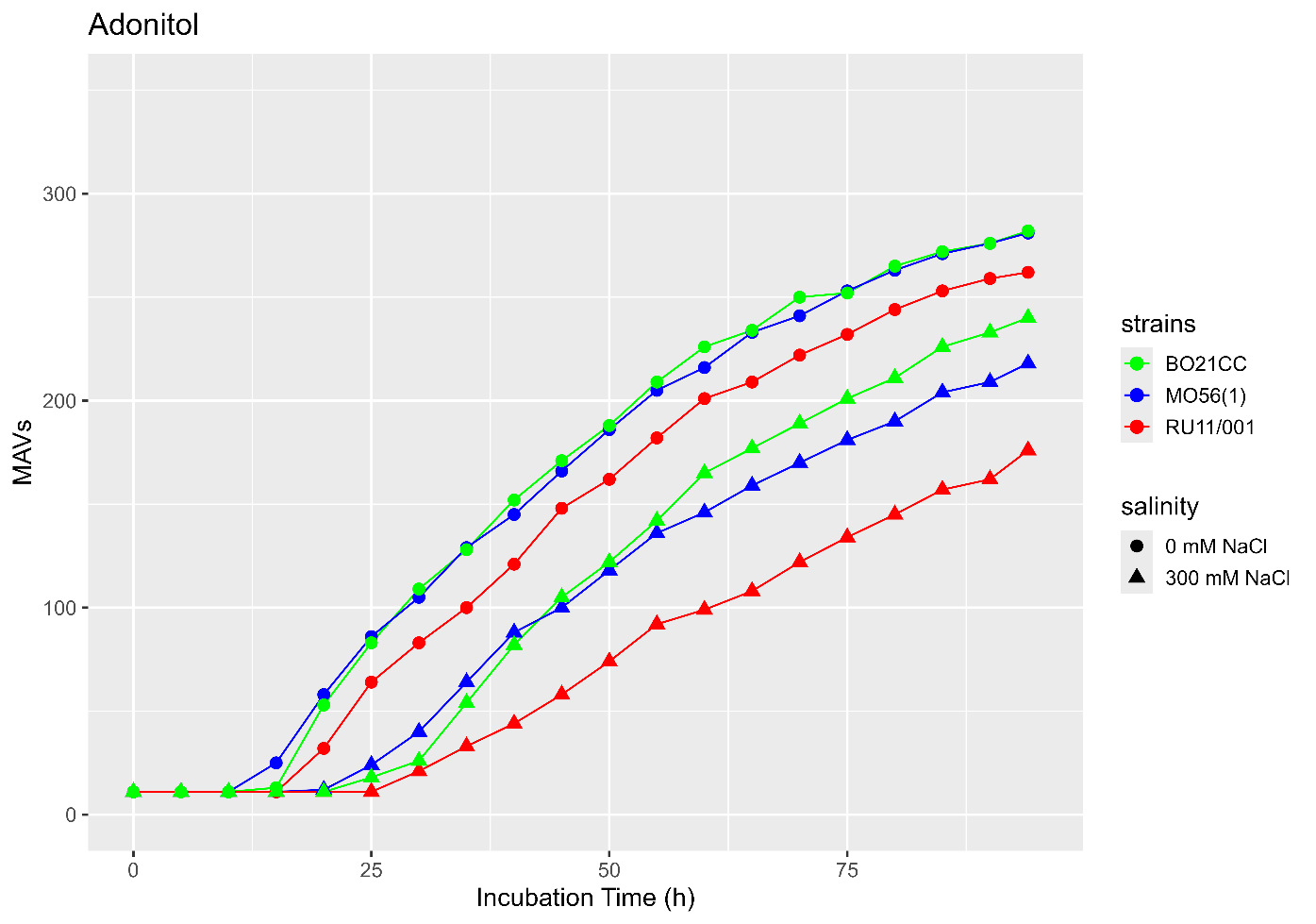
**Adonitol**

**α-methyl D-galactoside**


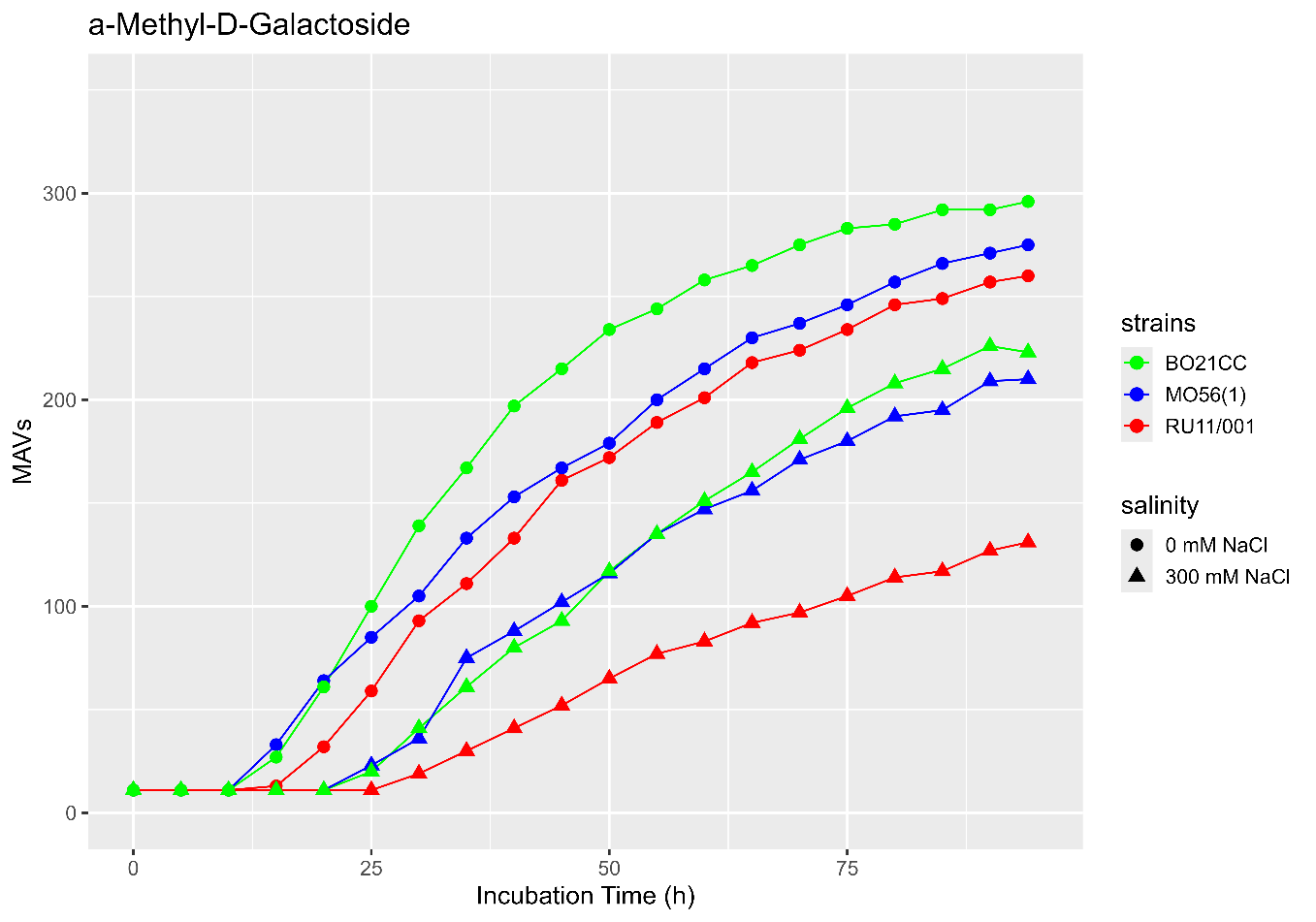


**β-methyl-D-glucoside**


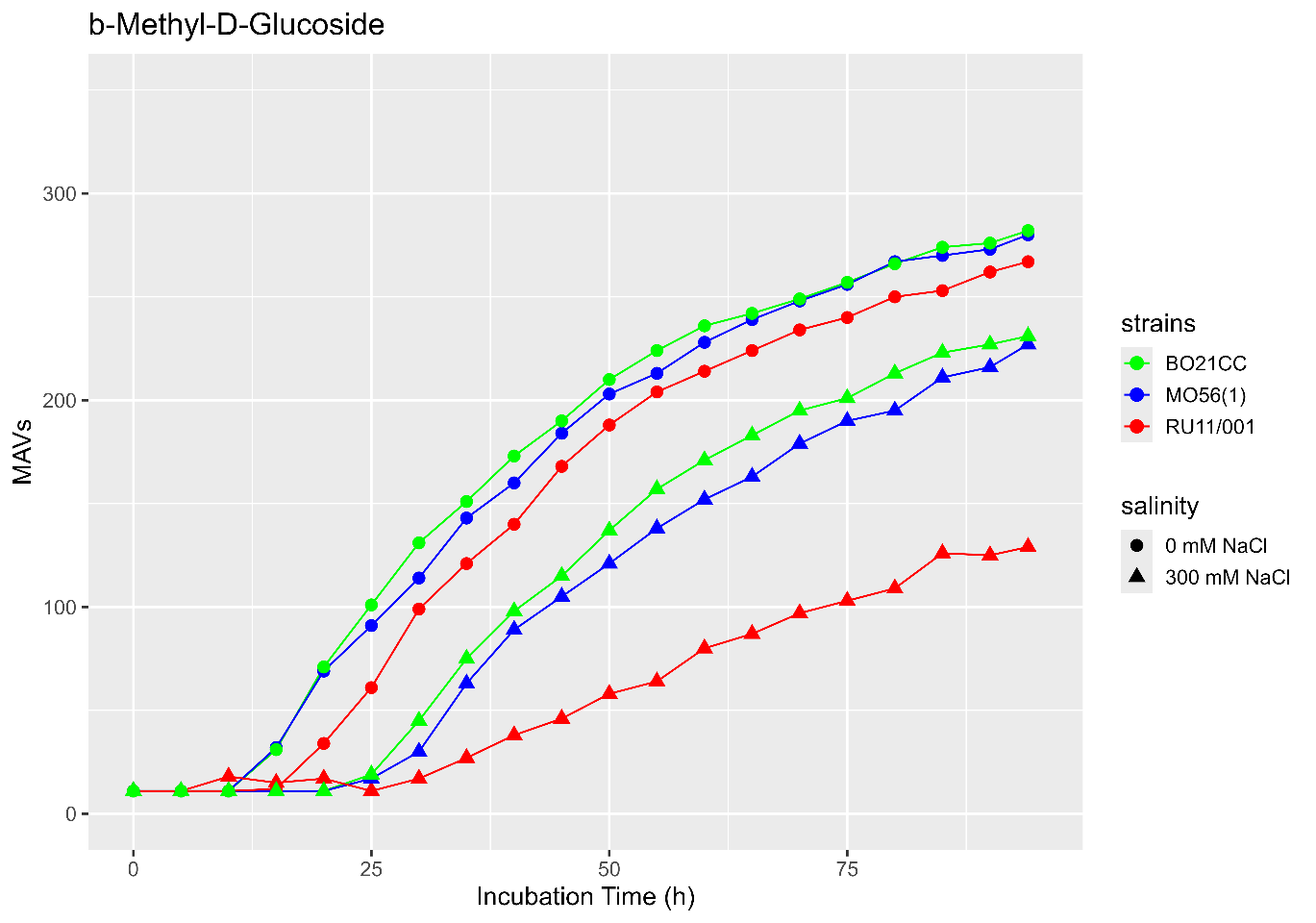


**M-inositol**


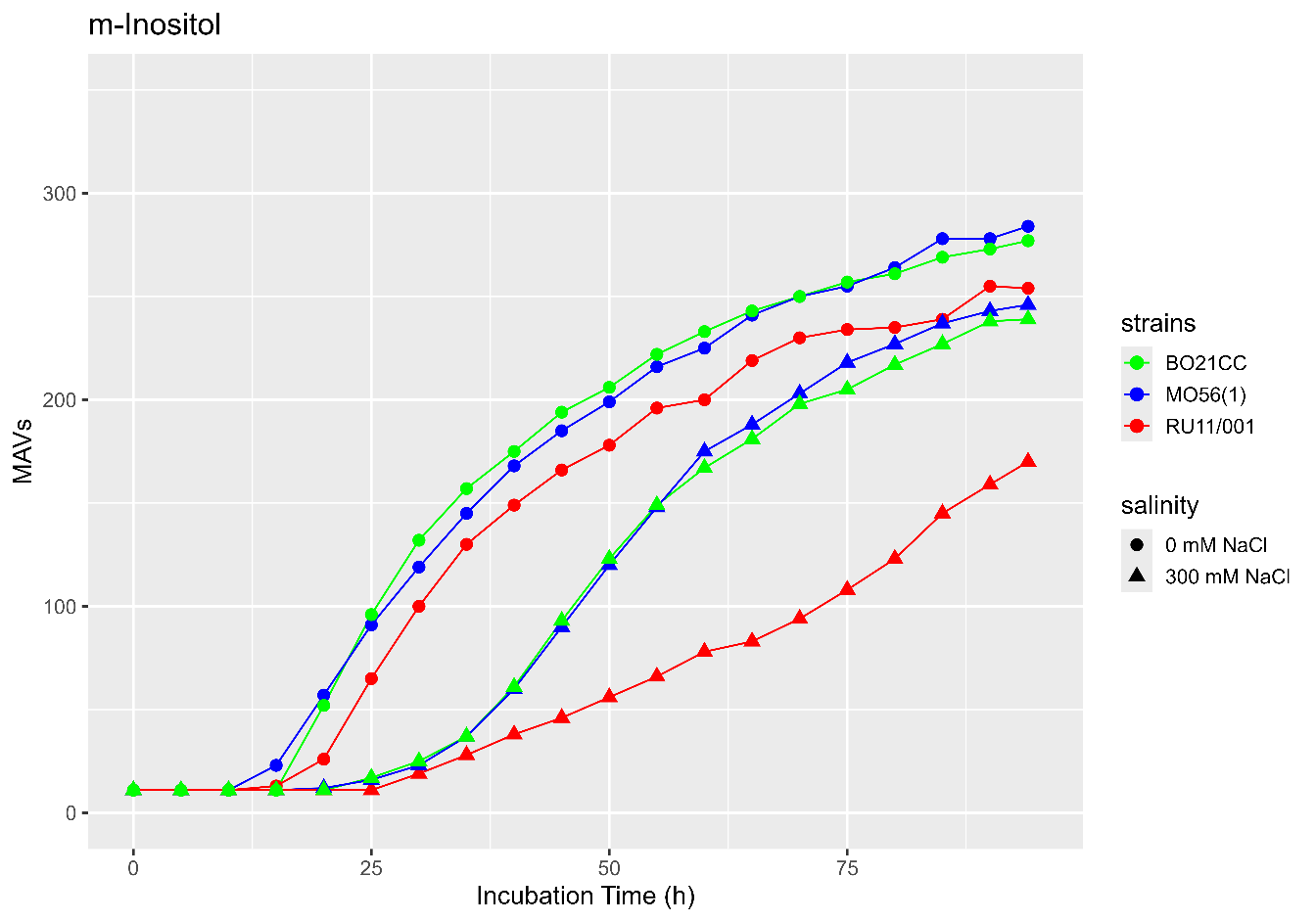


**Glycerol**

**
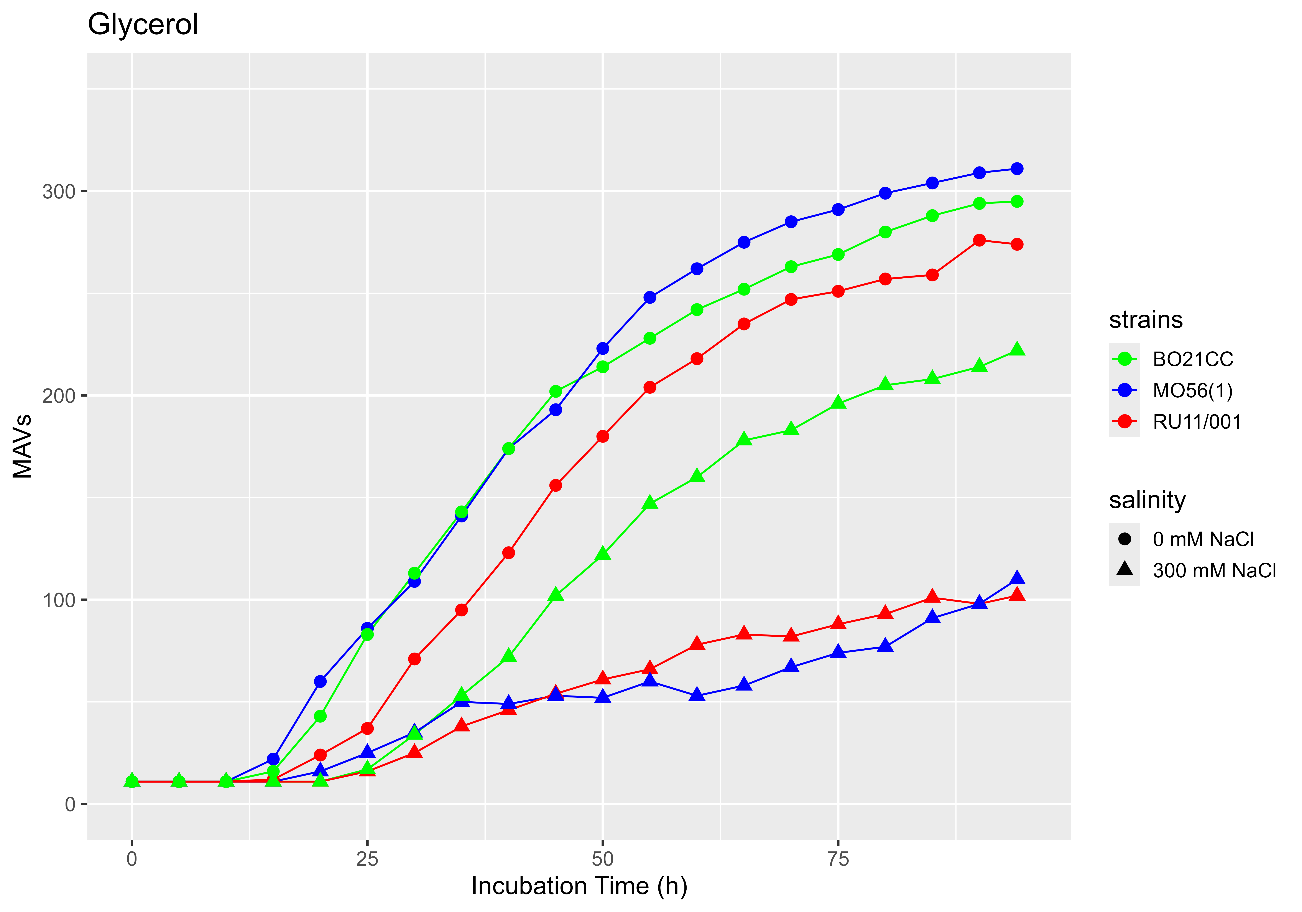
**

**D-arabinose**

**
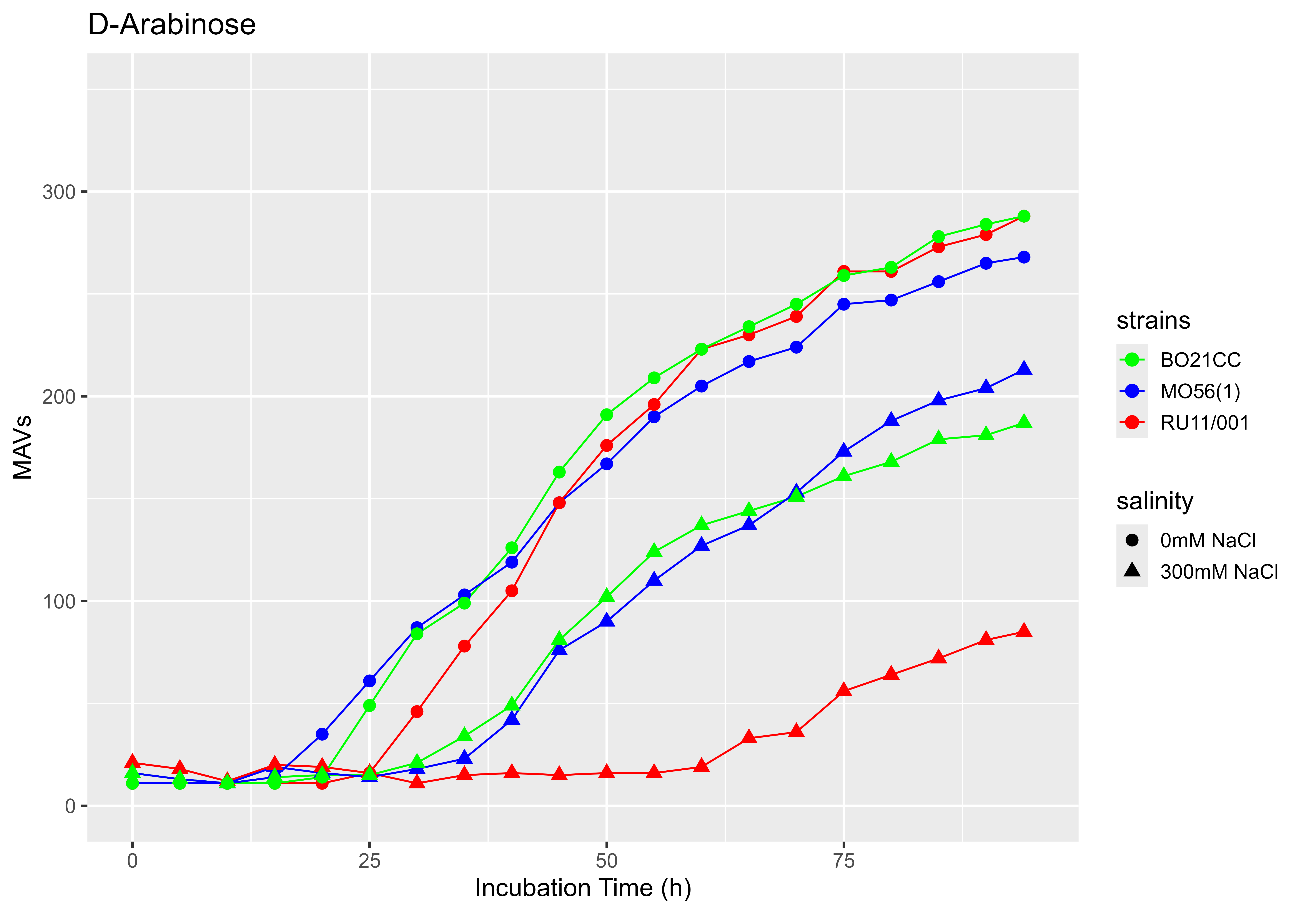
**

**
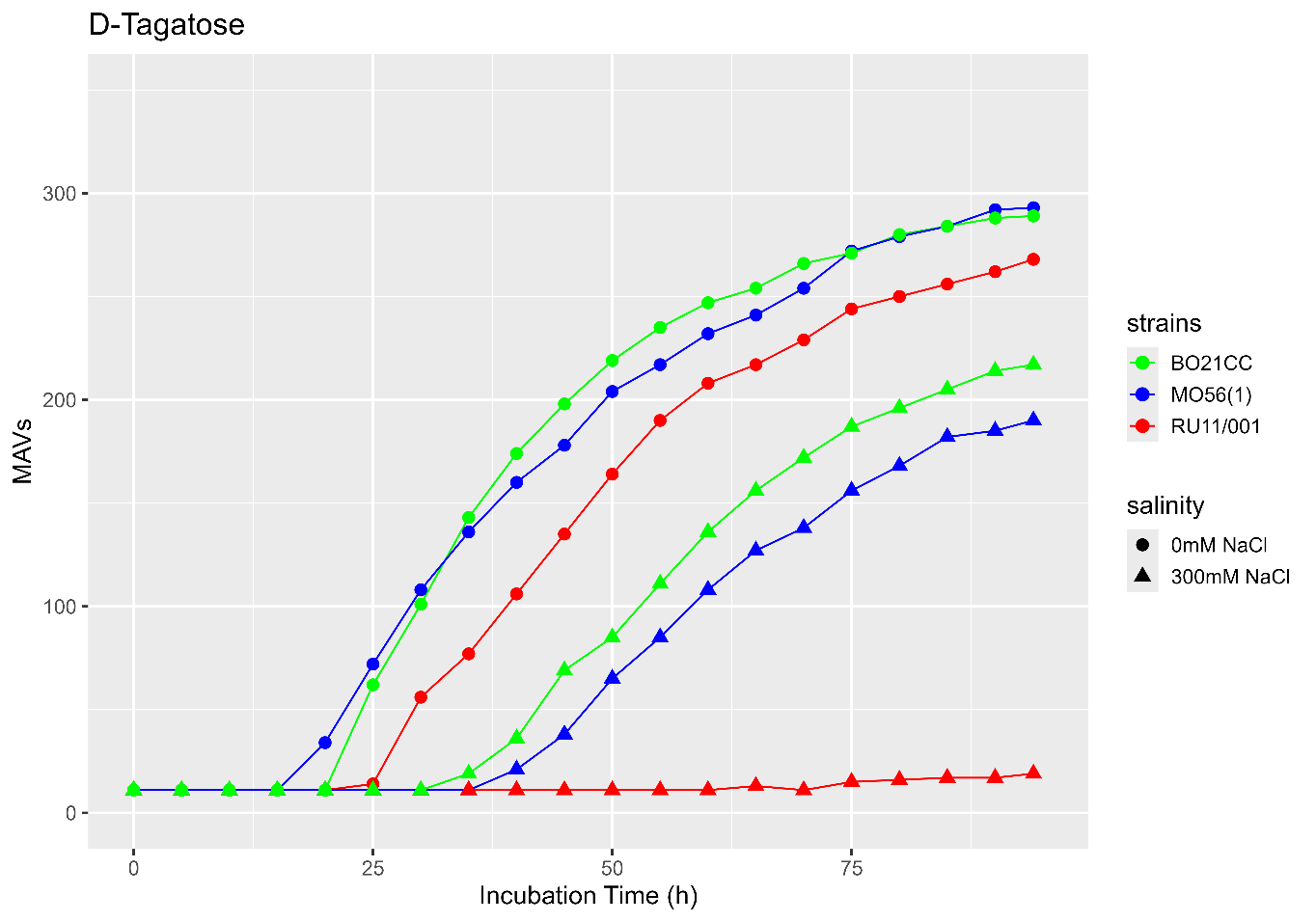
D-tagatose**

**D-fructose**

**
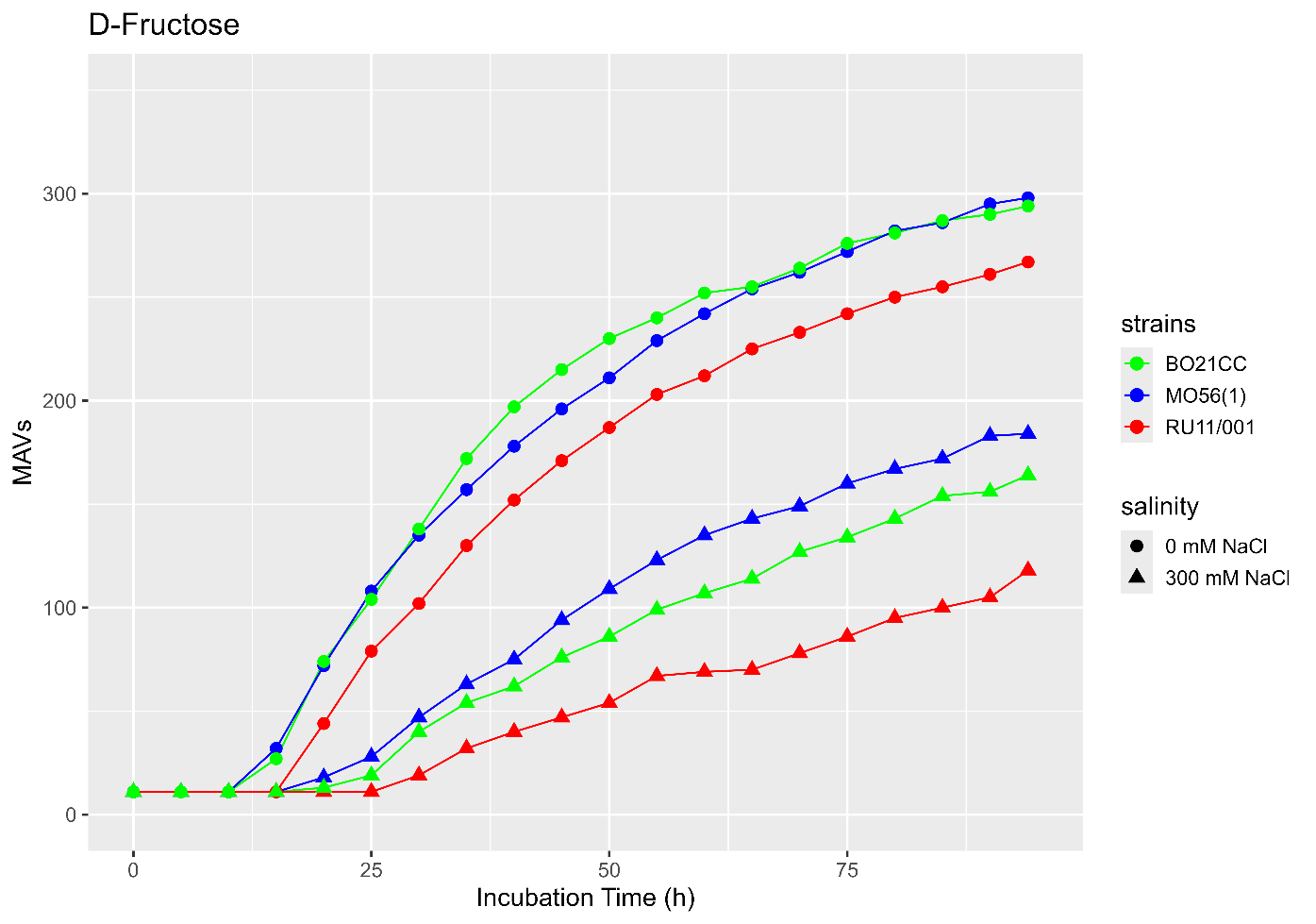
**

**a-D-lactose**


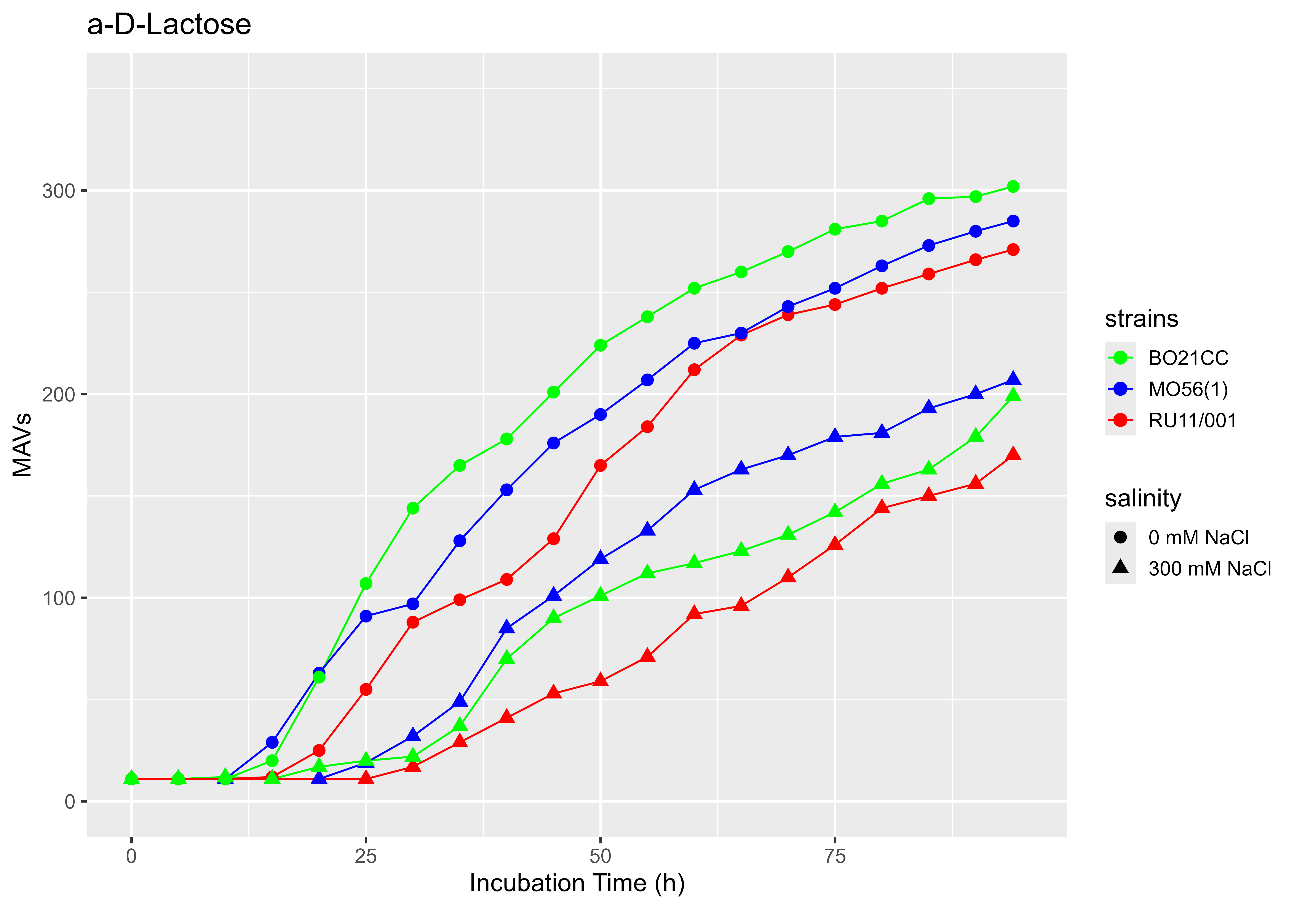


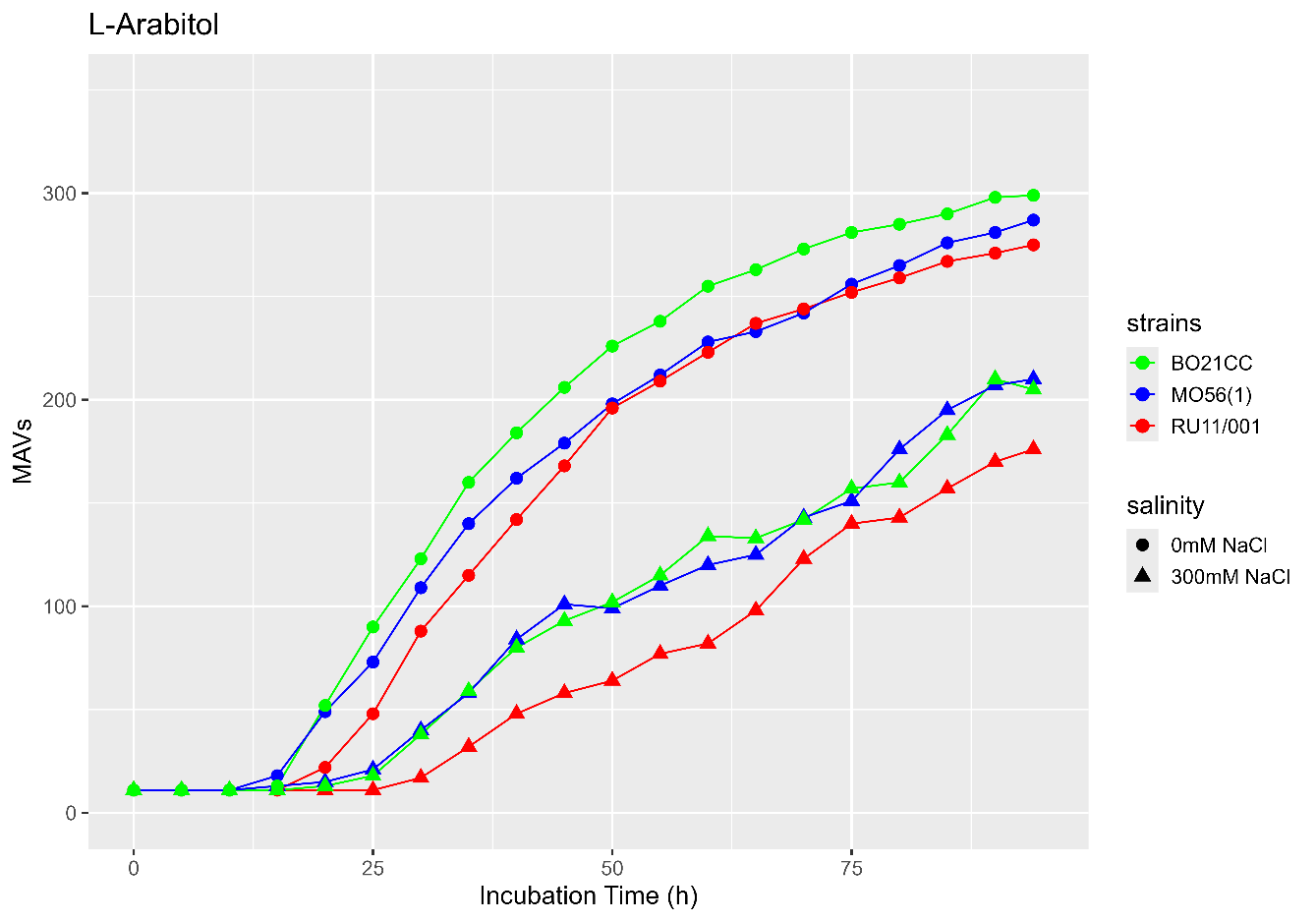
**L-arabitol**

**3-methylglucose**


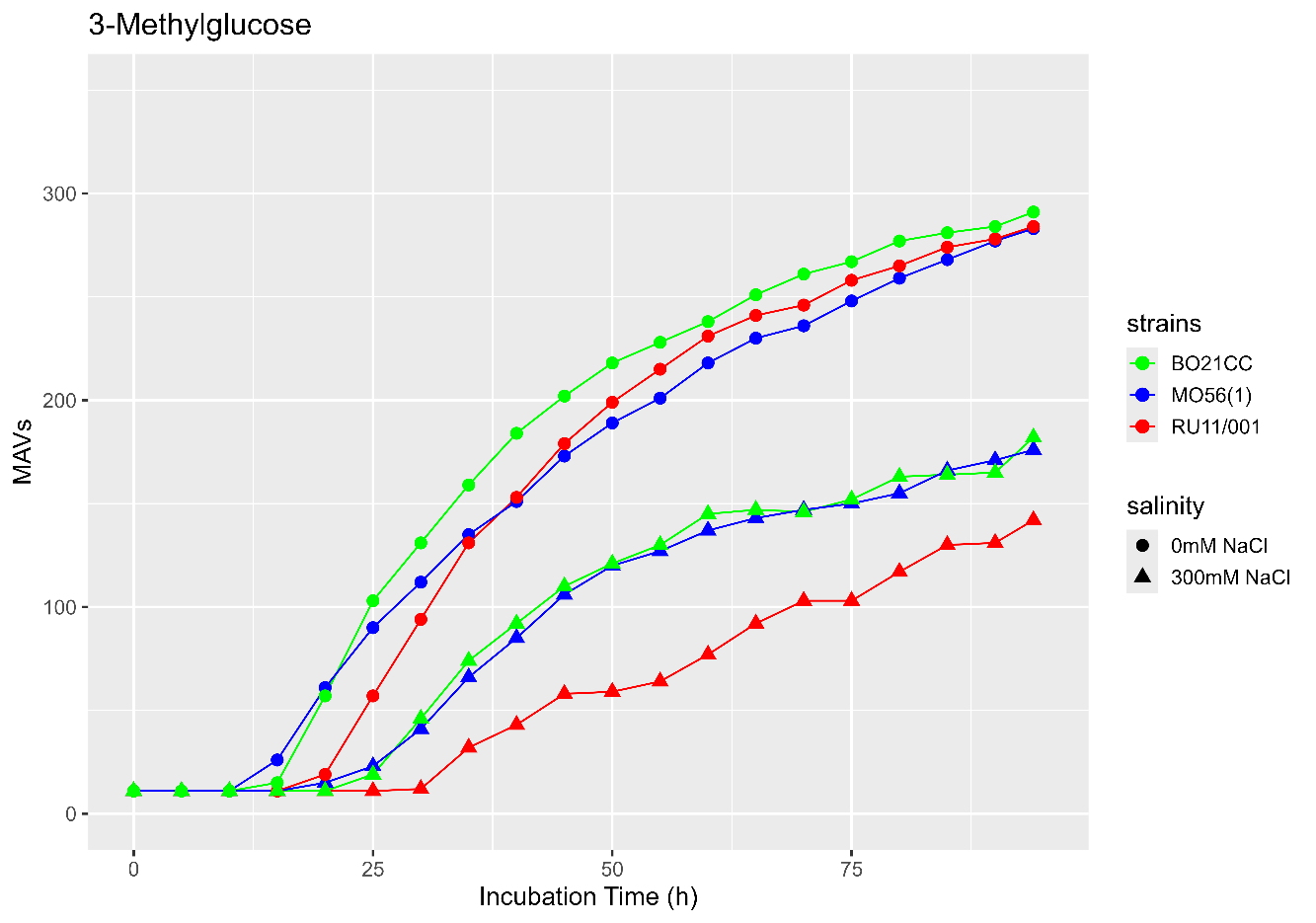


**Melibionic acid**


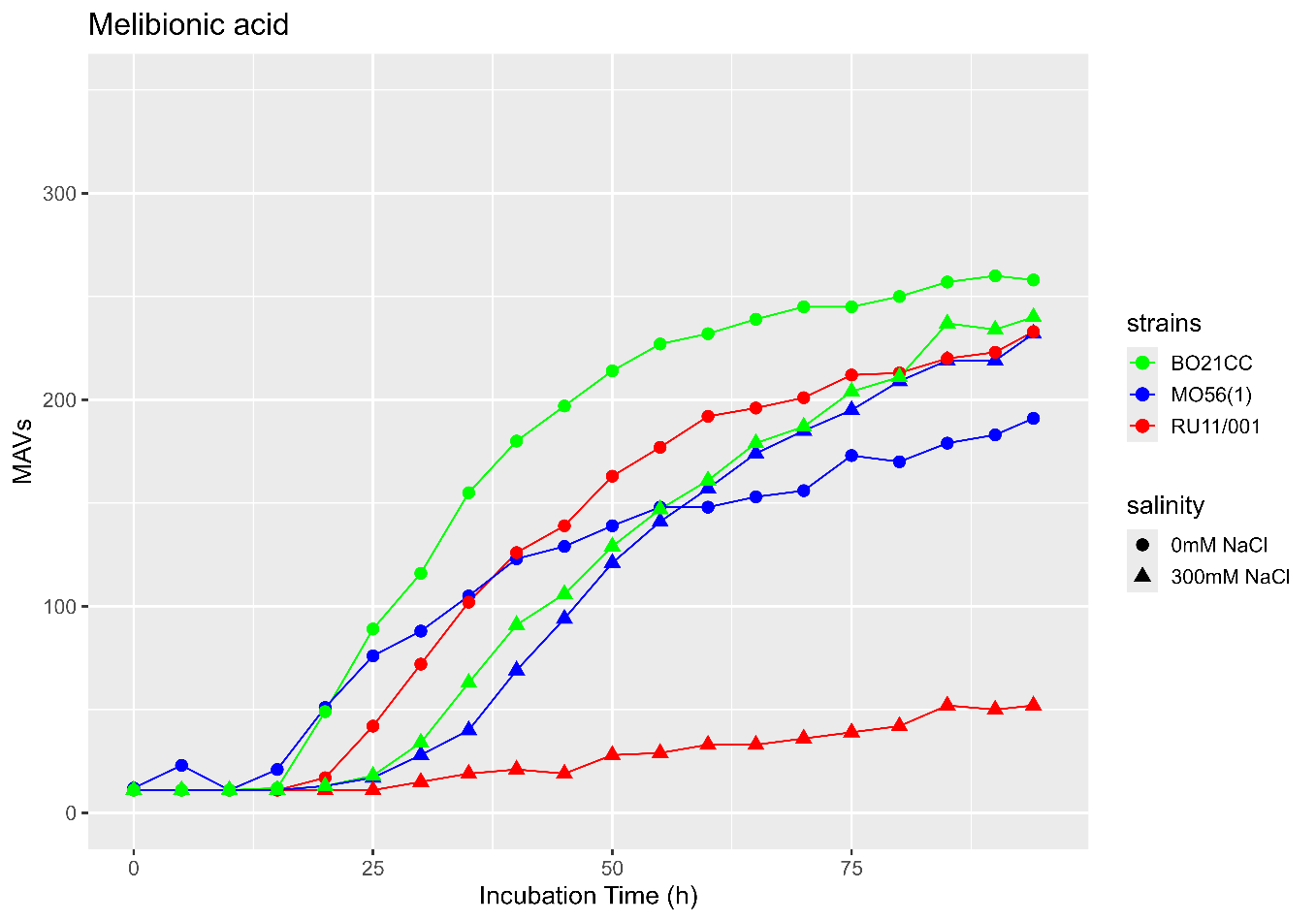


**D-fucose
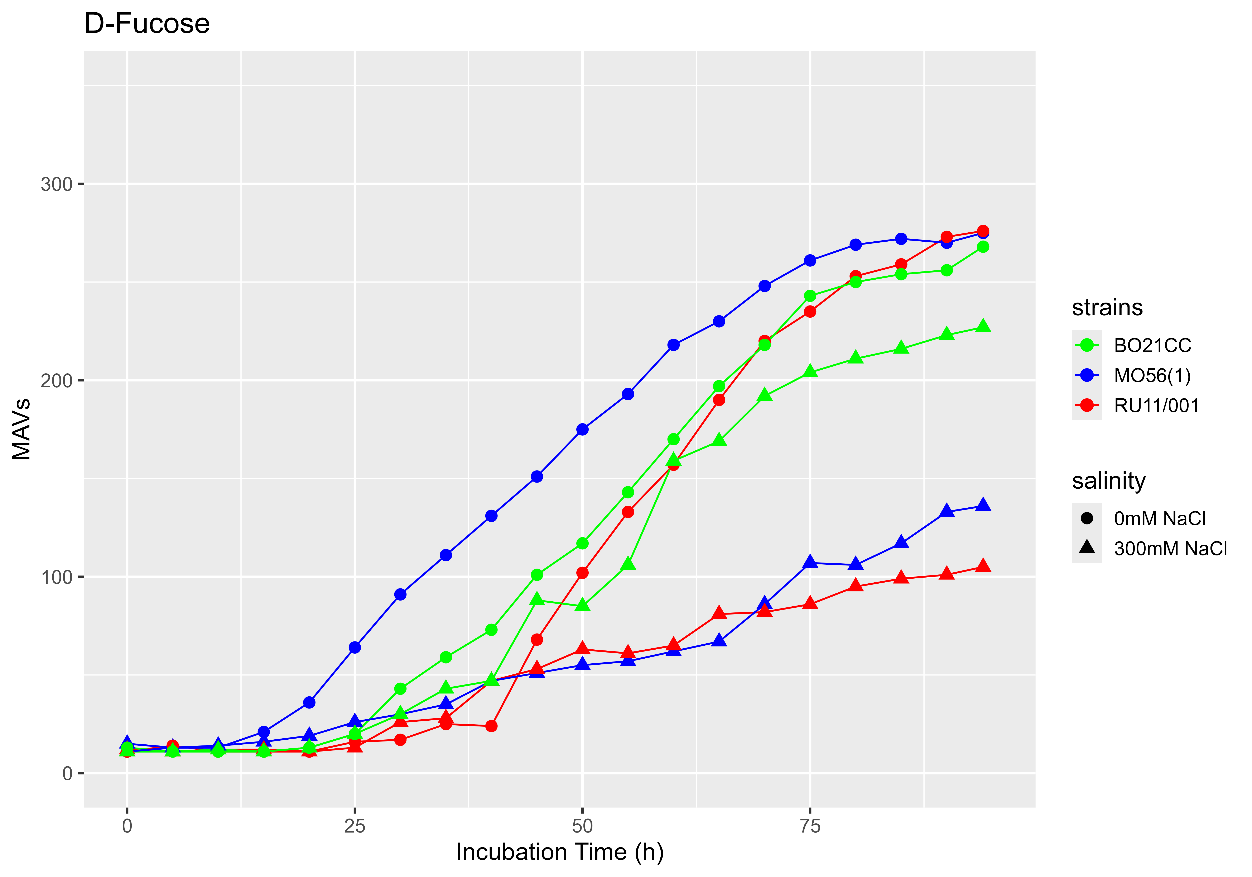
**
